# Pharmacological Mechanism of Shen Huang Chong Ji for Treating Alzheimer's Disease Based on Network Pharmacology and Experimental Validation

**DOI:** 10.1155/2022/9243348

**Published:** 2022-05-24

**Authors:** Lei Tang, Jing Liu, Xiaozhuo Xu, Juan Zhao, Xu Han

**Affiliations:** ^1^Renal Division, Affiliated Hospital of Nanjing University of Chinese Medicine, Nanjing, China; ^2^First Clinic Medical School, Nanjing University of Chinese Medicine, Nanjing, China; ^3^Key Laboratory for Metabolic Diseases, Chinese Medicine, Nanjing University of Chinese Medicine, Nanjing, China; ^4^Geriatric Department, Affiliated Hospital of Nanjing University of Chinese Medicine, Nanjing, China

## Abstract

The traditional Chinese medicine (TCM) formula, Sheng Huang Chong Ji (SHCJ) is largely applied for treating Alzheimer's disease (AD), but not much is known regarding its active compounds, molecular targets, and mechanism of action. The current study aimed to predict the potential molecular mechanism of SHCJ against AD based on network pharmacology combined with in vitro validation. Using public databases, SHCJ's active compounds, their potential targets, and AD-related genes were screened, while Cytoscape Version 3.7.2 was used to build protein–protein interaction (PPI) and compound-disease-target (C-D-T) networks. Analysis of enriched Kyoto Encyclopedia of Genes and Genomes (KEGG) pathways and Gene Ontology (GO) terms was then carried out in *R* 4.0.2, including associated packages. Subsequently, molecular docking analysis was performed with AutoDock Vina 1.1.2, with intro experiments involving SH-SY5Y cells used to further investigate the mechanism of SHCJ against AD. Finally, a total of 56 active compounds of SHCJ and 192 SHCJ-AD-related targets were identified. Quercetin was identified as the top potential candidate agent. HSP90AA1, AKT1, and MAPK1 represent potential therapeutic targets. The PI3K-Akt signaling pathway potentially represents a core one mediating the effects of SHCJ against AD. Additionally, molecular docking analysis indicated that quercetin could combine well with AKT1 and multiple apoptosis-related target genes. During cell experiments, a significant increase in cell viability along with a decrease in A*β*_25-35_-induced apoptosis was observed after treatment with SHCJ. Furthermore, SHCJ significantly increased the phosphorylation of PI3K and Akt while reversing A*β*_25-35_-induced apoptosis-related protein expression downregulation.

## 1. Introduction

The elderly population around the world is being increasingly affected by Alzheimer's disease (AD), with memory loss and a progressive decline in the cognitive function being two main characteristics of this neurodegenerative disease. In 2020, an estimated 59 million people were affected by AD, according to the World Alzheimer´s Report, and with the ageing population increasing and the lack of effective interventions, there is no doubt that the number is projected to triple by 2050 [1]. At present, the prevention and clinical intervention of AD, as a major global challenge, has become a common concern in modern society. Hence, the development of effective clinical therapy is of great significance for delaying disease progression, ameliorating cognitive functions, thereby improving patients' quality of life.

Traditional Chinese medicine (TCM) has been reported to be effective in treating cardiovascular diseases, neurological diseases, respiratory diseases, and tumours [2–5]. In addition, various herbs and compounds, including Rhizome *Anemarrhenae* and *Astragalus*, have been demonstrated to have definite effects on neurodegenerative diseases [6, 7], suggesting that Chinese herbs hold great potential for treating AD.

Shen Huang Chong Ji (SHCJ), combined with clinical experience, is made up of Ren Shen Gu Ben Wan, and its information is recorded in a TCM book under the name of Yi Fang Kao. SHCJ, which is composed of Ginseng Radix etRhizoma, Polygonati Rhizoma, Epimedii Folium, Rehmanniae Radix Praeparata, and Polygoni Multiflori Radix Praeparata at a ratio of 2 : 2:1 *Astragalus* 1:1, has achieved curative effects since its development. SHCJ is known to improve serum nitric oxide (NO) levels, reduce serum superoxide dismutase (SOD) levels, scavenge free radicals, and enhance endogenous antioxidant capacity in ageing patients [8]. In addition, SHCJ reduces the expression levels of p16 mRNA transcript for p16 [9], which is believed to regulate human cell ageing at the genetic level, with its mechanism involving delaying cellular senescence by inhibiting the proliferation of senescent cells. However, the active compounds and their potential mechanisms in SHCJ against AD have not been well elucidated.

Network pharmacology, based on principles of systems biology, basically analyses biological networks to identify specific signal nodes that would allow the design or identification of multitarget drugs. So far, this method has proved to be useful in clarifying the multitarget effect of TCM in several diseases [10, 11]. Molecular docking is a method used to virtually screen the best experimental results and docking control measures, and this method is conducive to large-scale prospective research [12, 13]. Combining molecular docking with network pharmacology to study traditional Chinese medicine provides new means through which active TCM compounds can be screened and explored. This combined approach, along with in vitro cell experiments, was therefore applied in the current study to explore the active compounds in SHCJ, their potential targets, and the possible underlying mechanism for the observed effects against AD.

**Figure 1:**
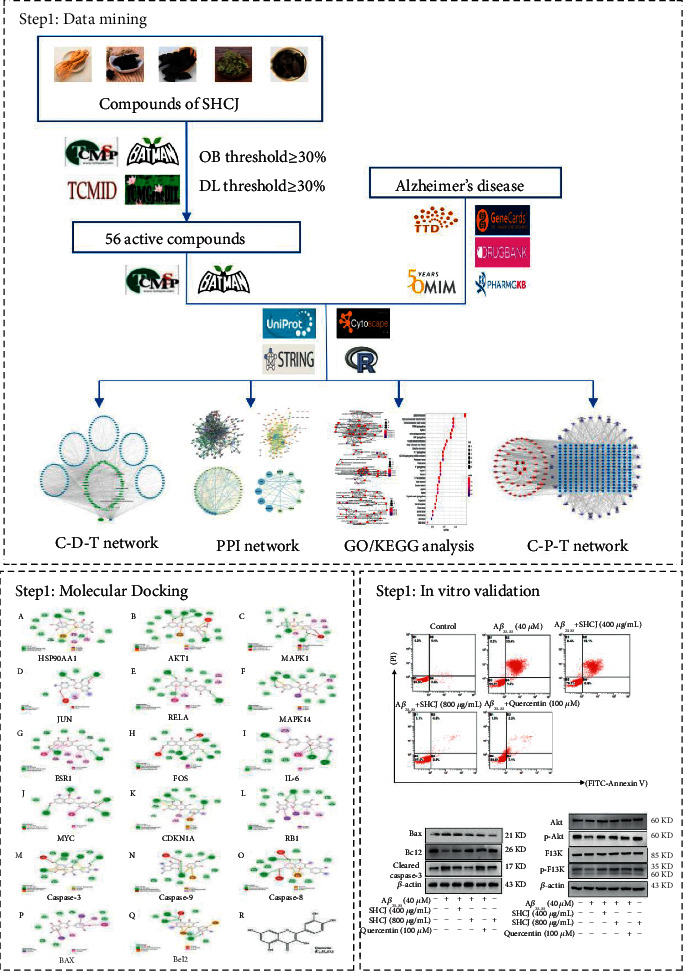
Workflow for prediction and validation of anti-AD mechanisms based on in vitro cell experiments, molecular docking, and network pharmacology.

Figure 1 shows the workflow for the above process.

## 2. Materials and Methods

### 2.1. Data Preparation

#### 2.1.1. Composition of SHCJ

Four publicly available databases, the Traditional Chinese Medicine Integrated Database (TCMID, https://www.megabionet.org/tcmid/), the Bioinformation Analysis Tool for Molecular Mechanism of Traditional Chinese Medicine (BATMAN-TCM, https://bionet.ncpsb.org.cn/batman-tcm/), the Traditional Chinese Medicine Systems Pharmacology Database and Analysis Platform (TCMSP, https://tcmspw.com/tcmsp.php), and a database which uses text mining to obtain information on traditional Chinese medicine and associated genes and diseases (TCMGeneDIT, https://tcm.lifescience.ntu.edu.tw/), were used to extract data regarding the chemical components of five herbs present in SHCJ. These databases are widely used to explore compounds in network pharmacology as they can provide comprehensive information on TCM herbs and their ingredients [14–17].

#### 2.1.2. Active Compound Selection by Predicting Pharmacokinetic ADME

Compounds were screened based on absorption, distribution, metabolism, and excretion (ADME) parameters using the TCMSP database. The bioactive components of SHCJ were then identified by specifying the parameters for oral bioavailability (OB) and drug-likeness (DL). As an important pharmacokinetic parameter of oral drugs, OB reflects the percentage of an orally administered drug that is absorbed and reaches the circulatory system in the human body. On the other hand, DL considers how similar a compound is with respect to conventional medicine and is used to evaluate the “drug-like” state of the prospective compound, which helps to optimize pharmacokinetics and drug characteristics [18, 19]. In this study, using the TCMSP database, active compounds in SHCJ with specific OB and DL values of ≥30% and ≥0.18, respectively, were extracted for use in subsequent studies.

#### 2.1.3. Identification of Common Targets of AD and SHCJ's Active Compounds

AD-related genes were obtained using “Alzheimer Disease” as the keyword in public databases, five of which were the Therapeutic Target Database (TTD, https://db.idrblab.net/ttd/), the Online Mendelian Inheritance in Man (OMIM, https://www.omim.org/), PharmGKB (https://www.pharmgkb.org/), DrugBank (https://go.drugbank.com/), and Gene Card (https://www.genecards.org/) [20–24]. The sixth freely accessible database was UniProt (https://www.uniprot.org/), which provides protein sequences of high quality and their functional annotations [25], allowed for all target proteins to be converted into corresponding *Homo sapiens* genes. A Venn diagram was then used to represent overlapping portions between drug and disease targets, which reflected the common targets involved in AD's treatment with SHCJ.

### 2.2. Bioinformatics Analysis

#### 2.2.1. Analysis of Protein-Protein Interaction (PPI) Networks

Using the Search Tool for the Retrieval of Interacting Genes (STRING) database (https://string-db.org/), a PPI network was built, with only *Homo sapiens* selected as the species [26]. Analysis was performed with a high confidence interval (score ≥0.7). After importing the TSV file into Cytoscape Version 3.7.2 (https://cytoscape.org/), a widely used tool for constructing and visualizing PPI networks, topological characteristics were analysed with the “CytoNCA” plugin to obtain six topological parameters of nodes: local average connectivity (LAC), network centrality (NC), eigenvector centrality (EC), degree centrality (DC), betweenness centrality (BC), and closeness centrality (CC). The core target was subsequently identified based on the median of these parameters. Within the network, node significance was indicated by the values of the parameters, with higher ones indicating greater significance. Eventually, the CytoHubba plugin and the Maximal Clique Centrality (MCC) algorithm were used to screen hub genes independently to assess the results' reliability.

#### 2.2.2. Active Compounds, Disease Target Network Construction

A network of potential targets was built after importing the screening results for the active compounds of herbs and AD-related targets into Cytoscape Version 3.7.2. Within this network, nodes represented targets or active ingredients, while the relationships between biomolecules were indicated by node connections. Visual analysis of the network was achieved by combining the size of the node and edge reaction with the binding index.

### 2.3. Enrichment Analysis

To determine associated biological processes, Entrez gene IDs were first created from the approved symbols of identified SHCJ-AD target genes. Using *R* (4.0.2) and other associated packages, an analysis of enriched Kyoto Encyclopedia of Genes and Genomes (KEGG) pathways and Gene Ontology (GO) terms was then performed, with only those results for which the *p* values and *q* values were <0.05 considered to be of statistical significance.

### 2.4. Compound-Pathway-Target Network Construction

The compound-pathway-target (C-P-T) network was built with Cytoscape Version 3.7.2 to identify links between active compounds, target genes, and enriched pathways, which were represented by nodes with different colors and shapes. Visual analysis of the network is achieved by combining the size of the node and edge reaction with the binding index. The C-T-P network eventually allowed the core compounds to be obtained.

## 3. Validation

### 3.1. Molecular Docking

For molecular docking, the AutoDock Vina 1.1.2 software was used. Before docking, ligand structures were energy-minimized employing an MMFF94 force field, and all receptor proteins were hydrotreated by PyMol. ADFRsuite1.0 was then used to convert small molecules and receptor proteins into PDBQT format for easy identification and docking by AutoDock Vina1.1.2 software. The docking grid was centered on the centroid of proteins, and the center coordinates of the proteins are shown in Table 1. The appropriate *X*, *Y*, and *Z* side lengths were adjusted to construct the box and fully wrap the whole protein. The size parameters (*X*, *Y*, *Z*) of the grid box are shown in Table 1. Grid boxes and PDBQT files of processed proteins and small molecules were used as input files, and Vina was used for docking. The global search exhaustiveness was set to 32, with default values selected for the other parameters. Finally, the docking conformation with the highest output score was considered the binding conformation, and the docking results were analysed visually with PyMol.

### 3.2. In Vitro Cell Experiments

#### 3.2.1. Preparation Technology of SHCJ Freeze-Dried Powder

SHCJ granules were weighed in a 50 mL centrifuge tube and dissolved to 0.5 g/mL with ultrapure water. Then, 2 mL of solution was placed into 5 mL centrifuge tubes on an ultraclean table, and the centrifuge tube mouth was sealed with aluminium foil. The sealed centrifugal tube was freeze-dried for 48 h (SCIENTZ-12N, SCIENTZ, China) to make a dry powder, which was sterilized for 30 min with ultraviolet light. The freeze-dried powder was stored at −80°C after preparation.

#### 3.2.2. Cell Culture

Neuroblastoma cell lines SH-SY5Y were obtained from the cell library of the Chinese Academy of Sciences (SCSP-5014, Shanghai, China). Cells were cultured in Dulbecco's modified Eagle's medium (DMEM, Invitrogen, Carlsbad, USA) containing 1% of penicillin/streptomycin (Invitrogen) and 10% of foetal bovine serum (FBS, Thermo Fisher Scientific, Rockville, MD, USA) prior to incubation at 37°C and under 5% CO_2_.

#### 3.2.3. Cell Viability Assay

After seeding 6 × 10^3^ SH-SY5Y cells into each well of 96-well plates, incubation was performed for 24 h for cell attachment before treating them with different concentrations of SHCJ (0, 100, 200, 400, 800, 1600, and 3200 *μ*g/mL) for 24 and 48 h. A Cell Counting Kit-8 (CCK-8, MedChemExpress, New Jersey, USA) was then used to assess cell proliferation, with absorbance (OD) values determined at 450 nm using an enzyme labelling instrument (EXL808, BIOTEK, Vermont, USA).

#### 3.2.4. Flow Cytometry Analysis for Apoptosis

An SH-SY5Y cell suspension was first prepared at a density of 2 × 10^6^/mL and after seeding them into each well of six-well plates, cell treatment was performed for 24 h with 40 *μ*M of A*β*_25–35_ (Sigma, St. Louis, MO, USA) and SHCJ at different concentrations (0, 100, 200, 400, 800, 1600, and 3200 *μ*g/mL). Cell apoptosis was then measured with an Annexin V-FITC/PI Apoptosis Detection Kit (40302ES20, Yeasen Biotechnology, Shanghai, China) by following the manufacturer's instructions. Finally, apoptosis was determined by flow cytometry (FC500 MPL, Beckman Coulter), with data acquisition and analysis carried out using an MXP Cytometer and CXP Analysis 2.1.

#### 3.2.5. Western Blot Analysis

A cell suspension of similar density to that of flow cytometry analysis was seeded into six-well plates and left for 24 h for cell attachment. This was followed by a 24 h treatment with 40 *μ*M A*β*_25–35_, 400 and 800 *μ*g/mL of SHCJ, and 100 *μ*M of quercetin. The concentration of total proteins was subsequently determined with a Pierce BCA Protein Assay Kit (Thermo Fisher Scientific) and a concentration of 1 *μ*g/*μ*L was used for protein expression analysis. After protein separation by SDS–PAGE and subsequent transfer onto polyvinylidene fluoride (PVDF) membrane, the latter was blocked before being incubated overnight at 4°C with primary antibodies against PI3K (1 : 1000, 4257S, Cell Signaling Technology, Boston, MA, USA), p-PI3K (1 : 1000, 17366S, Cell Signaling Technology), AKT (1 : 1000, ab8805, Abcam, Cambridge, England), p-AKT (1 : 1000, ab38449, Abcam), Bcl-2 (1 : 1000, 4223S, Cell Signaling Technology), BAX (1 : 1000, 5023S, Cell Signaling Technology), cleaved caspase-3 (1 : 1000, 9661S, Cell Signaling Technology), and *β*-actin (1 : 1000, 4970S, Cell Signaling Technology). This was followed by a 1 h incubation with the secondary antibody (HRP-conjugated anti-rabbit/mouse IgG, 1 : 3000, 7074S/7076S, Cell Signaling Technology). After being washed, the membranes were eventually developed with Immobilon Western Chemiluminescent HRP (WBKLS0500, Millipore, Massachusetts, USA) for visualization using the ImageJ software (NIH), with greyscale values being also calculated using the same software to determine protein expression.

### 3.3. Statistical Analysis

Results from at least three separate experiments were presented as the mean ± SEM. SPSS 20.0 was used for data analysis, with the means of groups compared by one-way ANOVA to determine significance at *P* < 0.05.

## 4. Results

### 4.1. Data Statistics

#### 4.1.1. Acquiring Active Compounds of SHCJ by ADME Screening

SHCJ consists of Ginseng Radix et Rhizoma, Polygonati Rhizoma, Rehmanniae Radix Praeparata, Epimedii Folium, and Polygoni Multiflori Radix Praeparata. A total of 439 ingredients were recognized in SHCJ (Supplementary Table 1). ADME screening was carried out using DL ≥ 0.18 and OB ≥ 30% as parameters, and as many as 56 active compounds were identified in SHCJ after removing the repeats (Table 2), including 22 active compounds in Ginseng Radix et Rhizoma, 12 in Polygonati Rhizoma, 2 in Rehmanniae Radix Praeparata, 23 in Epimedii Folium, and 3 in Polygoni Multiflori Radix Praeparata. Beta-sitosterol, sitosterol, stigmasterol, and DFV were found in different herbs simultaneously.

#### 4.1.2. Common Targets of AD and SHCJ's Active Compounds

**Figure 2:**
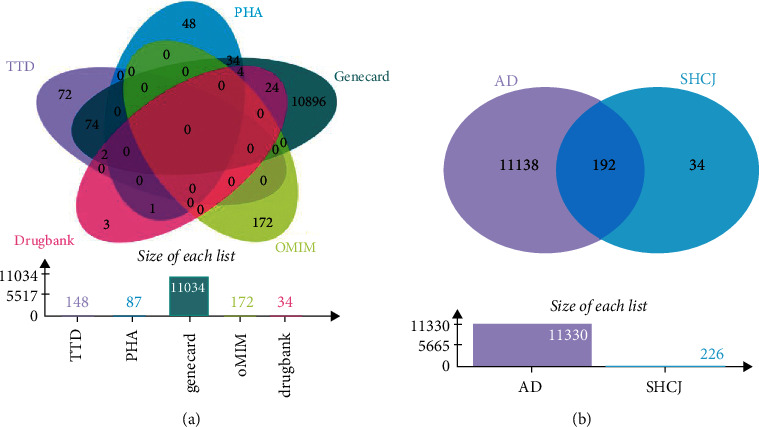
Venn diagram of AD-related targets as obtained from five databases (a) and the intersection of target genes of active compounds and those related to AD (b).

Using the TCMSP and BATMAN database allowed 226 potential target genes of SHCJ's active compounds to be identified, especially after removing duplicate values and transferring protein names into gene symbols (Supplementary Table 2). Next, from the GeneCards, PharmGkb, TTD, DrugBank, and OMIM databases, AD-related target genes were obtained using “Alzheimer Disease” as the keyword. In total, 11034 genes were identified in GeneCards, 172 in OMIM, 34 in DrugBank, 148 in TTD, and 87 in PharmGkb. A total of 11330 related target genes for AD were collected after identification in the intersection (Figure 2(a) and Supplementary Table 3). Eventually, 192 overlapping targets were identified out of the 11330 targets related to AD and the 226 ones of active compounds and these were treated as hub target genes for subsequent research (Figure 2(b) and Supplementary Table 4).

### 4.2. Bioinformatics Analysis

#### 4.2.1. Construction and Topological Analysis of the C-D-T Network

**Figure 3:**
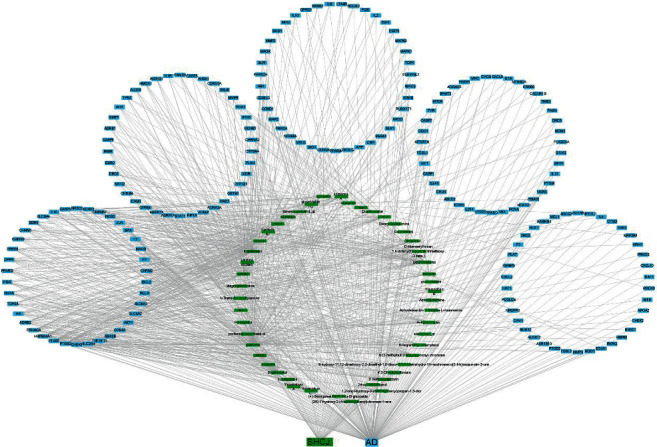
C-D-T network containing 242 nodes and 947 edges. SHCJ's active compounds and the hub target genes in AD are represented by green and blue rectangular nodes, respectively. The connectivity between nodes from right to left gradually increases, and nodes having more edges are of greater significance.

A C-D-T network with 242 nodes and 947 edges was used for visualizing the link between 56 active compounds of SHCJ and 192 hub target genes (Figure 3). Node correlation and network cores are determined by the connectivity of high-degree nodes in complex networks. Nodes that had more edges were of greater significance. The top 5 active compound nodes included quercetin (MOL000098, *n* = 192), kaempferol (MOL000422, *n* = 95), beta-sitosterol (MOL000358, *n* = 53), luteolin (MOL000006, *n* = 49), and stigmasterol (MOL000449, *n* = 43). The top 5 gene nodes were PTGS2, PTGS1, HSP90AA1, PRKACA, and ADRB2, with degree values of 39, 31, 26, 22, and 20, respectively. These genes may represent critical nodes within the network.

#### 4.2.2. Construction of the AD-Related PPI Network and Analysis of Its Topological Features

**Figure 4:**
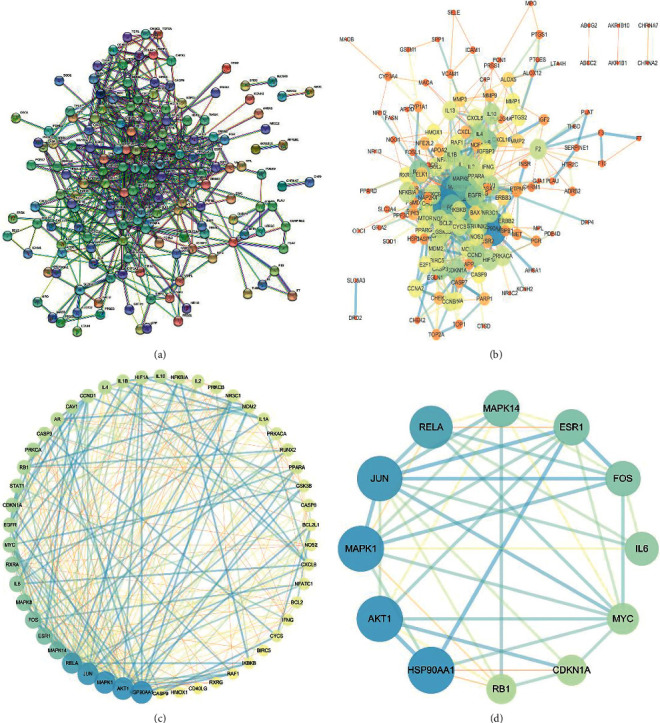
PPI network of SHCJ-AD. (a) The STRING database was used for obtaining an interactive PPI network with 158 nodes and 644 edges. (b) PPI network imported into Cytoscape 3.7.2 for building and visualizing the network. (c) PPI network of significant proteins obtained from (b) and based on six parameters: BC > 77.152593, CC > 0.093230535, DC > 6.00, EC > 0.037252683, NC > 2.6964285, and LAC >2.00. The new network contained 50 nodes and 292 edges. (d) PPI network of core proteins obtained from (c) after filtering the six parameters further: BC > 299.65972, CC > 0.096705395, DC > 14.5, EC > 0.110636165, NC > 6.144697, and LAC >4.435294. The core network contains 12 nodes and 43 edges. Larger node sizes and darker node colors indicate higher degree values.

After submitting overlapping core target genes to the STRING database, a PPI network was built. In total, 158 nodes and 644 edges were obtained (Figure 4(a)) and imported into Cytoscape 3.7.2 for building and visualizing the network (Figure 4(b)). As noted before, within the network, node significance was reflected in the values of the parameters, with higher values indicating greater significance. Target nodes for which parameters exceeded the network's median (LAC >2.00, NC > - 2.6964285, EC > 0.037252683, DC > 6.00, CC > 0.093230535 and BC > 77.152593) were reserved for constructing a new PPI network containing 50 nodes and 292 edges for further study (Figure 4(c)). The median screening was repeated twice to narrow down the range of core target nodes, and nodes with parameters greater than the median (BC > 299.65972, CC > 0.096705395, DC > 14.5, EC > 0.110636165, NC > 6.144697, and LAC >4.435294) were retained to build a core PPI network with 12 nodes and 43 edges (Figure 4(d)). Node size and color indicated the degree value, with larger and darker nodes reflecting higher degree values. The results showed that the top 5 nodes were HSP90AA1, AKT1, MAPK1, JUN, and RELA, and these genes could be actively involved in AD.

### 4.3. Enrichment Analyses

#### 4.3.1. GO Enrichment Analysis

**Figure 5:**
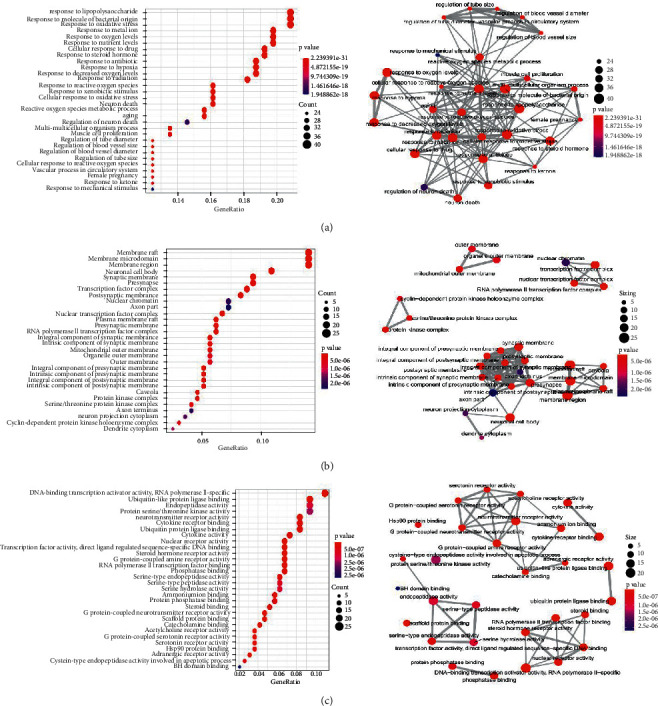
GO enrichment analysis annotating three functional aspects: BP (a), CC (b), and MF (c). The lower *p* value and the redder color represent greater enrichment of the GO terms.

The 192 hub target genes were annotated based on three functions: molecular function (MF), cellular component (CC), and biological process (BP). Of the enriched GO terms identified, 2826 were statistically significant and included 2486 from BP, 120 from CC, and 220 from MF. The top 30 ones are presented in a bubble diagram (Figure 5). The lower *p* value and the redder color represent greater enrichment of the GO terms. In this study, regarding AD treatment, the hub target genes of SHCJ were mostly enriched in the ubiquitin-like protein ligase binding (GO: 0044389), the response to oxidative stress (GO: 0006979), RNA polymerase II-specific (GO: 0001228), the response to lipopolysaccharide (GO: 0032496), membrane regions (GO: 0098589), membrane microdomains (GO: 0098857), the response to molecules of bacterial origin (GO: 0002237), endopeptidase activity (GO: 0004175), and membrane rafts (GO: 0045121).

### 4.4. Enrichment Analysis of KEGG Pathway

**Figure 6:**
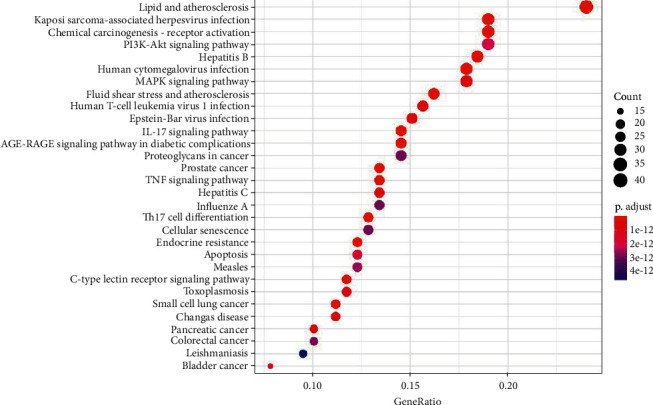
Bubble diagram of the top 30 enriched KEGG pathways. A larger dot size indicates that more genes were annotated in the entry, while redder colors reflected lower *p* values.

**Figure 7:**
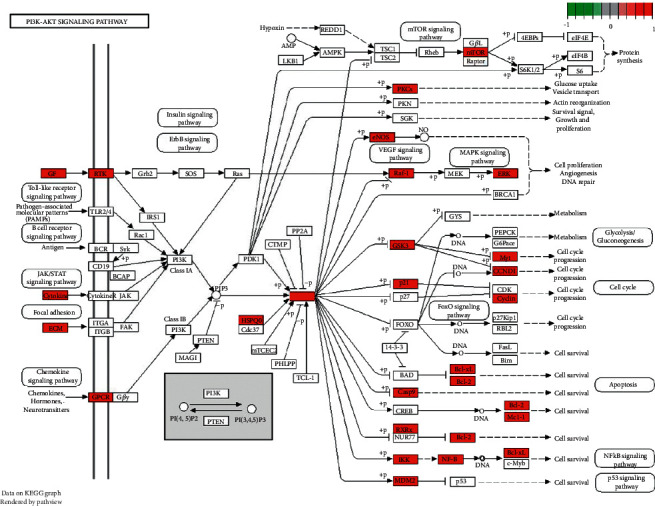
Map of the PI3K/AKT signaling pathway, with SHCJ-AD-related genes represented as red nodes.

An analysis of enriched KEGG pathways was performed to identify key functions and signaling pathways of the hub target genes. A total of 172 statistically significant SHCJ-AD-related pathways were obtained (*P* ≤ 0.05), with the top 30 significantly enriched ones having high gene counts shown as the core pathways in Figure 6, including lipid and atherosclerosis (*n* = 43), Kaposi sarcoma-associated herpesvirus infection (*n* = 34), chemical carcinogenesis-receptor activation (*n* = 34), PI3K-Akt signaling pathway (*n* = 34), and hepatitis B (*n* = 33). These results indicated that SHCJ could treat AD via different mechanisms, with the PI3K-Akt signaling pathway for which most genes were enriched, representing a core pathway in the treatment of SHCJ. Notably, the PI3K/AKT signaling pathway map further demonstrated the regulatory mechanism of SHCJ-AD-related genes represented by red nodes, suggesting that AKT activation could regulate the expression of downstream apoptosis-related genes, such as Casp9, Bcl2, and BAX (Figure 7).

4.4.1. Construction of the C-P-T Network and Analysis of Its Topological Features

**Figure 8:**
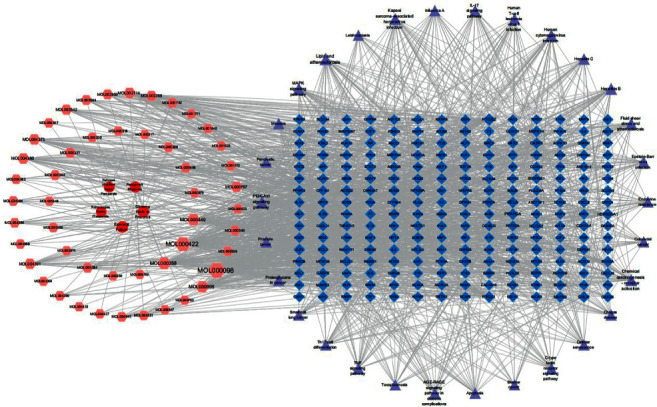
The top 30 pathways in C–P-T networks, with the five herbs in SHCJ represented as a red circular node. Pink hexagon nodes represent active compounds from Ginseng Radix et Rhizoma, Polygonati Rhizoma, Rehmanniae Radix Praeparata, Epimedii Folium, and Polygoni Multiflori Radix Praeparata of SHCJ. Blue square nodes represent the target genes. Purple triangle nodes represent the top 30 significantly enriched pathways where node sizes reflected the size of the degree.

A C-P-T network with 308 nodes and 1626 edges was built to elucidate the interrelationships of active compounds, target genes, and the top 30 pathways (Figure 8). Through topological analysis, three core compounds, namely, quercetin (MOL000098, DC = 133), kaempferol (MOL000422, DC = 108), and beta-sitosterol (MOL000358, DC = 62), were identified based on their highest DC values. Three pathways were also selected as the key pathways: chemical carcinogenesis-receptor activation (DC = 34), lipid and atherosclerosis (DC = 43), and the PI3K-Akt signaling pathway (DC = 34).

## 5. Validation

### 5.1. Molecular Docking Analysis

**Figure 9:**
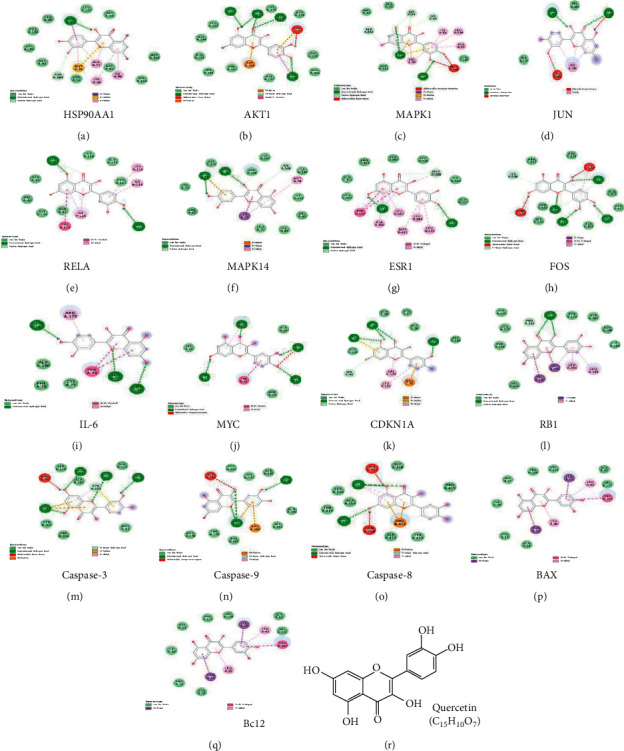
The docking model of quercetin with the top 12 core target genes and apoptosis-related target genes in the topological analysis of AD-related PPI networks. (a-l) Twelve core target genes, including HSP90AA1, AKT1, MAPK1, JUN, RELA, MAPK14, ESR1, FOS, IL-6, MYC, CDKN1A, and RB1, with quercetin. (m-q) Apoptosis-related target genes, including Bcl2, BAX, Caspase-8, Caspase-9, and Caspase-3, with quercetin. (r) The chemical structure and molecular formula of quercetin.

By screening core target genes, 12 important targets were obtained, including HSP90AA1, AKT1, MAPK1, JUN, RELA, MAPK14, ESR1, FOS, IL-6, MYC, CDKN1A, and RB1. Topological analysis showed that quercetin was the top active compound in the prediction. Thus, we present the docking process of quercetin and 12 core target genes, as shown in Figure 9(a–l) and Table 3. Further analysis showed that the cohesive energy between quercetin and 12 core proteins was less than −5 kcal/mol, suggesting stable binding effects. Among them, the cohesive energies of HSP90AA1, AKT1, MAPK14, ESR1, and FOS were all less than −8 kcal/mol, yielding a more stable docking result. In addition, multiple apoptosis-related target genes were identified in the topological analysis of AD-related PPI networks, such as BAX, Bcl2, Caspase-8, Caspase-9, and Caspase-3. Thus, we also performed the docking process of quercetin and these apoptosis-related target genes, as shown in Figure 9(m-q) and Table 3. The cohesive energy between quercetin and Caspase-3, Caspase-9, Caspase-8, and Bcl2 was less than −7 kcal/mol, suggesting a stable binding effect. Figure 9(r) shows the chemical structure and molecular formula of quercetin, while Supplementary Table 5 provides the results of other docking analyses. Therefore, the representative compound quercetin obtained from SHCJ could bind well with 12 core target genes of AD, thus making it likely to be important for AD treatment. Apoptosis may be an important pathological mechanism of AD, and SHCJ could also be involved in AD treatment by inhibiting neuronal apoptosis.

### 5.2. Experimental Outcomes

#### 5.2.1. SHCJ Protected A*β*_25-35_-Treated SH-SY5Y Cells

**Figure 10:**
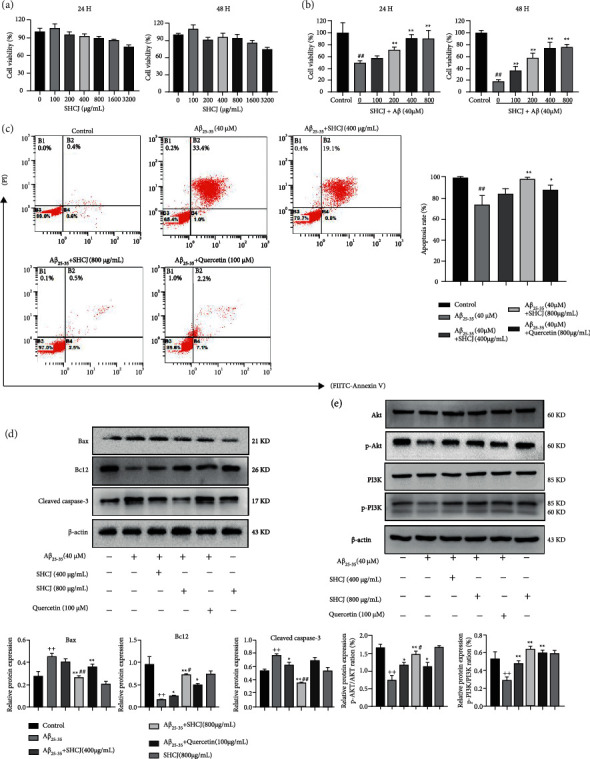
In vitro validation based on SH-SY5Y cells. (a-b) Viability of SH-SY5Y cells after being treated with SHCJ, quercetin, and A*β*_25-35_, based on CCK8 assays. (c) Annexin V-FITC/PI staining was used for analyzing apoptosis which was quantified as the apoptosis rate. (d-e) The effects of quercetin on PI3K/AKT pathway protein expression and apoptosis in SH-SY5Y cells treated with A*β*_25-35_. The data represent the mean ± SEM. *n* = 3. ^++^*P* < 0.01 vs. control; ^*∗*^*P* < 0.05, ^*∗∗*^*P* < 0.01 vs. A*β*_25-35_; ^#^*P* < 0.05, ^##^*P* < 0.01 vs. A*β*_25-35_ + quercetin.

After treatment with different concentrations of SHCJ, the viability of A*β*_25-35_-treated SH-SY5Y cells was assessed by CCK-8 assays.  Figure 10(a) showed no effects on cell viability at SHCJ concentrations of less than or equal to 800 *μ*g/mL. In contrast, compared with untreated cells, those treated with A*β*_25-35_ were of significantly lower viability (Figure 10(b)). However, after 24 and 48 h incubation with different concentrations of SHCJ (0, 200, 400, and 800 *μ*g/mL), A*β*_25-35_-treated cells displayed improved cell proliferation. Accordingly, 400 and 800 *μ*g/mL SHCJ were selected as the doses for use in subsequent experiments.

#### 5.2.2. SHCJ Inhibited A*β*_25-35_-Induced Cell Apoptosis in SH-SY5Y

A*β*_25-35_ treatment resulted in a significantly higher percentage of apoptotic cells, with the subsequent inhibitory effects of SHCJ on A*β*_25-35_-induced apoptosis being dependent on the SHCJ concentration. These results pointed towards SHCJ's neuroprotective effects on A*β*25-35-treated cells, with these effects being better than those of quercetin (Figure 10(c)).

#### 5.2.3. Inhibition of A*β*_25-35_-induced Apoptosis and Protein Expression in the PI3K/AKT Pathway by SHCJ

Bioinformatics analysis results identified multiple apoptosis-related genes, including CASP3, CASP9, BCL2, and BAX, in the hub target genes. In addition, the PI3K/AKT pathway was identified as a key one that defined SHCJ's effects against AD. Thus, the molecular mechanism by which SHCJ is involved in inducing SH-SY5Y cell apoptosis was further explored. Results of western blotting indicated that A*β*_25-35_-treated SH-SY5Y cells had lower levels of the p-AKT/AKT, Bcl-2, and p-PI3K/PI3K proteins compared with unstimulated ones, while cleaved caspase-3 and BAX proteins were upregulated (Figure 9(f)). Treatment with SHCJ or quercetin also increased the phosphorylation of PI3K and Akt while reversing amounts of apoptosis-related proteins, with the effects of SHCJ being superior to that of quercetin (Figures 10(d), 10(e)).

## 6. Discussion

Neuronal loss caused by intracellular neurofibrillary tangles and amyloid deposits is the main pathological feature of AD, whereas apoptosis has been demonstrated to be an important mechanism leading to neuronal loss [27]. In AD, neuronal apoptosis occurs through multiple signaling pathways. Inhibition of the ROCK signaling pathway improved cognitive impairment, alleviated neuronal injury, and inhibited hippocampal tissue and cell apoptosis in AD mice [28]. PI3K regulates neuronal survival through its downstream target AKT-mediated antiapoptotic effects and has been demonstrated to provide neuroprotection by inhibiting the protein glycogen synthase kinase-3 *β* (GSK-3*β*) to interfere with the innate apoptotic signals of the upstream apoptosome [29]. Endoplasmic reticulum (ER) stress is linked to a loss of neurons in AD, and PI3K/AKT pathway activation inhibits ER stress reactive protein and reduces hippocampal apoptosis in AD mice [30].

In modern clinical treatment, SHCJ displays good therapeutic effects and various pharmaceutical active ingredients have been found to have neuroprotective effects. Ginsenoside has a good protective effect on cerebral ischaemia injury, and the neuroprotective mechanism is linked to SIRT1 signaling pathways and TLR4/MyD88 activation [31]. Polysaccharides from Polygonati Rhizoma can be used to treat *β*-_amyloid 25-35_ (A*β*_25-35_)-induced neurotoxicity in PC12 cells [32]. Icariin inhibits the expression of *β*-site APP cleaving enzyme 1 (BACE-1) and amyloid precursor protein (APP), both of which cause AD [33]. Therefore, the active compounds of SHCJ, including their molecular targets and potential mechanisms, were explored using network pharmacology to explore before subsequently verifying the potential mechanism through in vitro experiments and molecular docking.

Using the different public databases, 56 active SHCJ compounds and 192 SHCJ-AD-related targets were identified. The results of this study not only showed that quercetin was the most important natural compound of SHCJ against AD but also that, within the C-D-T network, this compound was associated with most genes. Quercetin is a flavonol bioactive substance that displays various pharmacological activities and its ability to cross the blood-brain barrier (BBB) has been demonstrated, thereby highlighting its potential as a drug to prevent neurodegenerative diseases [34, 35]. Quercetin has high inhibitory activity against A*β* aggregation and reverses A*β*-induced neurotoxicity by forming hydrophobic interactions and hydrogen bonds with the *β*-sheet structure of A*β* [36]. In aged triple-transgenic AD model mice, quercetin inhibits the formation of NFTs and decreases the phosphorylation of tau proteins [37]. All the above-mentioned studies point towards the importance of quercetin in SHCJ's effects against AD.

The PPI network was constructed to reveal that HSP90AA1, AKT1, and MAPK1 might represent core targets in the anti-AD process of SHCJ, given that these genes were linked to most target nodes. We mainly focused on the AKT1 gene, which codes for one type of serine/threonine-protein kinase called AKT kinase (AKT3, AKT2, and AKT1), as one of the critical nodes in the networks. AKT1 activation relies on the PI3K pathway, which mediates a variety of biological processes through serine/threonine phosphorylation of a series of downstream substrates. Studies have demonstrated that synaptic dysfunctions in AD were the result of oxidative modification of AKT1 by ROS species, and the possible mechanism may be caused by a subsequent reduction in the AKT1 target of mTOR signaling, leading to deficiencies in activity-dependent protein translation [38]. AKT1 kinase has been shown to protect against neurodegeneration in animal and cellular models, and overexpressing recombinant AKT1 or p-AKT1 in AD models was shown to promote neurotrophic factor-mediated neuroprotection [39]. Recent studies have shown that enhancing AKT1 inhibits JNK3/caspase-3 activation and plays a neuroprotective role in ischaemia/reperfusion-induced cognitive impairment [40, 41]. AKT1 overexpression effectively inhibited neuronal cytotoxicity induced by the interaction of mitochondrial dependence and endoplasmic reticulum stress-induced apoptotic pathways [42]. In summary, apoptosis is a crucial mechanism of neuronal loss in the pathological process of AD, whereas AKT1, as the core target of SHCJ against AD, can protect neurons by mediating antiapoptotic effects. In addition, we also found a series of apoptosis-related proteins in the PPI network, such as JUN, CASP3, CASP9, BCl2, and BCL2L1, providing further evidence for our hypothesis.

The GO enrichment analysis annotated target genes from BP, CC, and MF. The results suggested that enrichment was mostly observed in membrane regions, membrane microdomains, and membrane rafts. Target genes were also enriched in response to lipopolysaccharide, in oxidative stress, and in molecules of bacterial origin. Of note, target genes were also enriched in neuron death and regulation of neuron death in the BP annotation. The analysis of enriched KEGG pathways yielded 172 signaling pathways for which the target genes were significantly enriched, thus reflecting SHCJ's ability to exert therapeutic effects against AD via different mechanisms. Combined with the results of the C-T-P network analysis, the PI3K-Akt signaling pathway, for which most genes were enriched, was identified as a key signaling pathway in AD treatment. These analyses and the above results mutually corroborate each other. As a vital cell signal transduction pathway, the PI3K/AKT signaling pathway has attracted great attention in neurodegenerative diseases due to its inhibition of apoptosis, promotion of cell proliferation, and maintenance of cell cycle regulation. It was reported that the response of the PI3K/Akt signaling pathway was significantly weakened in the hippocampus of AD patients [43]. AD's pathogenesis involves A*β* interactions with the PI3K/AKT pathway. For instance, it has been shown that A*β* could increase Tau phosphorylation and induce neurotoxicity by inhibiting the PI3K/AKT pathway in neuronal cells [44]. Meanwhile, when the PI3K/AKT signaling pathway gets activated, it can increase the expression level of antiapoptotic proteins in Bcl-2 family members, inhibit mitochondrial apoptosis, and reduce levels of caspase 9, caspase 3, and intracellular reactive oxygen species (ROS) [45].

Validation of the results from network pharmacology was achieved by molecular docking, with the docking results showing that quercetin not only bound to the top 12 core target genes but also combined well with apoptosis-related genes, such as BAX, Bcl2, Caspase-8, Caspase-9, and Caspase-3. Hence, we designed in vitro cell experiments to verify the results obtained after using both approaches (i.e., molecular docking and network pharmacology). A*β* deposition is a major neuropathological feature of AD, so we established an AD model whereby 40 *μ*M of A*β*_25-35_ was used for treating SH-SY5Y cells for 24 h, as previously reported [46]. The results showed that both SHCJ and quercetin could regulate the PI3K/AKT signaling pathway in AD by attenuating neurotoxicity caused by A*β*_25-35_. SHCJ and quercetin were confirmed to upregulate the phosphorylation of AKT and PI3K while increasing the level of the antiapoptotic protein Bcl2. At the same time, being proapoptotic proteins, the levels of cleaved caspase-3 and BAX were reduced. It is noteworthy that quercetin, as a positive control drug for SHCJ in the treatment of AD [47], has an inferior inhibitory effect on A*β*_25-35_-induced SH-SY5Y cell apoptosis compared to SHCJ. These findings reflect the therapeutic advantages of traditional Chinese medicine, for which multiple components act on multiple targets.

## 7. Conclusion

In summary, the main active ingredients of SHCJ, their target genes, and the potential underlying mechanisms of their activities against AD were predicted using a network pharmacology approach. PPI and C-T-P networks were constructed. This network revealed that quercetin was the most important active compound, AKT1 potentially represented a key target gene, and the PI3K-AKT signaling pathway was potentially of great significance. In addition, in vitro experiments and molecular docking confirmed the accuracy of the predictions made by network pharmacology. Finally, within the context of a multidisciplinary intervention strategy, this study provides an innovative and reliable method to explore the active compounds of traditional Chinese medicine, its molecular targets, and potential mechanisms, especially in providing conclusive evidence regarding the therapeutic effects of SHCJ on AD.

## Figures and Tables

**Table 1:** Central coordinates of the protein and the parameters of the docking region.

Gene name	PDB ID	Grid box	
		Center coordinates (*X, Y, Z*)	Size parameters (*X, Y, Z*)
HSP90AA1	6tn5	32.311, 14.712, 20.285	50.0, 52.0, 52.0
AKT1	3mv5	2.29, −0.721, 25.379	66.0, 58.0, 70.0
MAPK1	3sa0	−2.025, 8.346, 46.564	66.0, 52.0, 78.0
JUN	1jnm	15.865, 3.428, 29.021	32.0, 40.0, 96.0
RELA	6qhl	23.715, 12.347, 8.034	58.0, 64.0, 58.0
MAPK14	3lff	11.153, −4.657, −17.657	76.0, 52.0, 62.0
ESR1	3os8	21.44, 8.134, −68.509	62.0, 40.0, 62.0
FOS	1s9k	26.55, 23.416, 65.415	86.0, 72.0, 96.0
IL-6	4ni9	2.884, 21.709, 8.078	58.0, 56.0, 56.0
MYC	5i4z	20.53, 26.142, 9.853	92.0, 38.0, 32.0
CDKN1A	5e0u	−45.634, 41.642, −12.013	46.0, 74.0, 60.0
RB1	2qdj	6.335, 35.71, 20.609	60.0, 72.0, 74.0
Caspase 3	3kjf	4.344, 6.913, 2.268	74.0, 70.0, 54.0
Caspase 9	2ar9	16.849, 40.99, 0.996	46.0, 58.0, 58.0
Caspase 8	3kjn	−3.27, 29.642, 27.204	56.0, 60.0, 70.0
BAX	4bd6	−17.863, −14.654, −3.905	84.0, 72.0, 58.0
Bcl2	1ysw	2.222, -4.204, 3.104	54.0, 56.0, 40.0

**Table 2:** Bioactive compounds of SHCJ.

Mol ID	Molecule name	OB (%)	DL	Herb
MOL002879	Diop	43.59	0.39	Ginseng radix et rhizoma
MOL000449	Stigmasterol	43.83	0.76	Ginseng radix et rhizoma
MOL000358	Beta-sitosterol	36.91	0.75	Ginseng radix et rhizoma Polygonati rhizoma
MOL003648	Inerminact	65.83	0.54	Ginseng radix et rhizoma
MOL000422	Kaempferol	41.88	0.24	Ginseng radix et rhizoma Epimedii folium
MOL004492	Chrysanthemaxanthin	38.72	0.58	Ginseng radix et rhizoma
MOL005308	Aposiopolamine	66.65	0.22	Ginseng radix et rhizoma
MOL005314	Celabenzine	101.88	0.49	Ginseng radix et rhizoma
MOL005317	Deoxyharringtonine	39.27	0.81	Ginseng radix et rhizoma
MOL005318	Dianthramine	40.45	0.20	Ginseng radix et rhizoma
MOL005320	Arachidonate	45.57	0.20	Ginseng radix et rhizoma
MOL005321	Frutinone A	65.90	0.34	Ginseng radix et rhizoma
MOL005344	Ginsenoside rh2	36.32	0.56	Ginseng radix et rhizoma
MOL005348	Ginsenoside-Rh4_qt	31.11	0.78	Ginseng radix et rhizoma
MOL005356	Girinimbin	61.22	0.31	Ginseng radix et rhizoma
MOL005357	Gomisin B	31.99	0.83	Ginseng radix et rhizoma
MOL005360	Malkangunin	57.71	0.63	Ginseng radix et rhizoma
MOL005376	Panaxadiol	33.09	0.79	Ginseng radix et rhizoma
MOL005384	Suchilactone	57.52	0.56	Ginseng radix et rhizoma
MOL005399	Alexandrin_qt	36.91	0.75	Ginseng radix et rhizoma
MOL005401	Ginsenoside Rg5_qt	39.56	0.79	Ginseng radix et rhizoma
MOL000787	Fumarine	59.26	0.83	Ginseng radix et rhizoma
MOL001792	DFV	32.76	0.18	Polygonati rhizoma Epimedii folium
MOL002714	Baicalein	33.52	0.21	Polygonati rhizoma
MOL002959	3′-Methoxydaidzein	48.57	0.24	Polygonati rhizoma
MOL000359	Sitosterol	36.91	0.75	Polygonati Rhizoma
MOL003889	Methylprotodioscin_qt	35.12	0.86	Polygonati Rhizoma
MOL004941	(2R)-7-Hydroxy-2-(4-hydroxyphenyl)chroman-4-one	71.12	0.18	Polygonati Rhizoma
MOL000546	Diosgenin	80.88	0.81	Polygonati Rhizoma
MOL006331	4′,5-Dihydroxyflavone	48.55	0.19	Polygonati Rhizoma
MOL009760	Sibiricoside A_qt	35.26	0.86	Polygonati Rhizoma
MOL009763	(+)-Syringaresinol-O-beta-D-glucoside	43.35	0.77	Polygonati Rhizoma
MOL009766	Zhonghualiaoine 1	34.72	0.78	Polygonati Rhizoma
MOL002268	Rhein	47.07	0.28	Polygoni multiflori radix praeparata
MOL008647	N-trans-feruloyltyramine	86.71	0.26	Polygoni multiflori radix praeparata
MOL001525	Daucosterol	36.91	0.75	Polygoni multiflori radix praeparata
MOL000359	Sitosterol	36.91	0.75	Rehmanniae radix praeparata Epimedii folium
MOL000449	Stigmasterol	43.83	0.76	Rehmanniae radix praeparata
MOL000622	Magnograndiolide	63.71	0.19	Epimedii folium
MOL004367	Olivil	62.23	0.41	Epimedii folium
MOL004388	6-Hydroxy-11, 12-dimethoxy-2, 2-dimethyl-1, 8-dioxo-2, 3, 4, 8-tetrahydro-1h-isochromeno[3, 4-h]isoquinolin-2-ium	60.64	0.66	Epimedii folium
MOL004382	Yinyanghuo A	56.96	0.77	Epimedii folium
MOL004396	1,2-Bis(4-hydroxy-3-methoxyphenyl) propan-1, 3-diol	52.31	0.22	Epimedii folium
MOL004386	Yinyanghuo E	51.63	0.55	Epimedii folium
MOL004391	8-(3-Methylbut-2-enyl)-2-phenyl-chromone	48.54	0.25	Epimedii folium
MOL000098	Quercetin	46.43	0.28	Epimedii folium
MOL004384	Yinyanghuo C	45.67	0.50	Epimedii folium
MOL004373	Anhydroicaritin	45.41	0.44	Epimedii folium
MOL001645	Linoleyl acetate	42.10	0.20	Epimedii folium
MOL004394	Anhydroicaritin-3-O-alpha-L-rhamnoside	41.58	0.61	Epimedii folium
MOL004425	Icariin	41.58	0.61	Epimedii folium
MOL004380	C-homoerythrinan, 1,6-didehydro-3,15,16-trimethoxy-, (3.beta.)-	39.14	0.49	Epimedii folium
MOL003542	8-Isopentenyl-kaempferol	38.04	0.39	Epimedii folium
MOL001510	24-Epicampesterol	37.58	0.71	Epimedii folium
MOL001771	Poriferast-5-en-3beta-ol	36.91	0.75	Epimedii folium
MOL000006	Luteolin	36.16	0.25	Epimedii folium
MOL003044	Chryseriol	35.85	0.27	Epimedii folium
MOL004427	Icariside A7	31.91	0.86	Epimedii folium

OB, oral bioavailability; DL, drug-likeness.

**Table 3:** Molecular docking of core target genes and quercetin.

Gene name kcal/mol	PDB ID	Interactions	Cohesive energy
HSP90AA1	6tn5	van der Waals, conventional hydrogen bond, carbon hydrogen bond, pi-sigma, pi-sulfur, pi-alkyl	−9.0
AKT1	3mv5	van der Waals, conventional hydrogen bond, unfavorable donor-donor, pi-cation, pi-anion, pi-donor hydrogen bond, amide-pi stacked	−8.1
MAPK1	3sa0	van der Waals, conventional hydrogen bond, carbon hydrogen bond, unfavorable donor-donor, unfavorable acceptor- acceptor, pi-sigma, pi-sulfur, pi-alkyl	−7.4
JUN	1jnm	van der Waals, conventional hydrogen bond, unfavorable donor-donor, unfavorable acceptor- acceptor, pi-alkyl	−5.3
RELA	6qhl	van der Waals, conventional hydrogen bond, carbon hydrogen bond, pi-pi stacked	−8.1
MAPK14	3lff	van der Waals, conventional hydrogen bond, carbon hydrogen bond, pi-anion, pi-sigma, pi-alkyl	−9.1
ESR1	3os8	van der Waals, conventional hydrogen bond, carbon hydrogen bond,	−8.0
FOS	1s9k	van der Waals, conventional hydrogen bond, carbon hydrogen bond, pi-pi T-shaped, pi-alkyl	−8.0
IL-6	4ni9	van der Waals, conventional hydrogen bond, pi-pi T-shaped, pi-alkyl	−6.3
MYC	5i4z	van der Waals, conventional hydrogen bond, unfavorable acceptor- acceptor, pi-pi T-shaped, pi-alkyl	−6.0
CDKN1A	5e0u	van der Waals, conventional hydrogen bond, carbon hydrogen bond, pi-anion, pi-sulfur, pi-alkyl	−7.0
RB1	2qdj	van der Waals, conventional hydrogen bond, carbon hydrogen bond, pi-sigma, pi-alkyl	−7.4
Caspase 3	3kjf	van der Waals, conventional hydrogen bond, unfavorable donor-donor, pi-cation, pi-donor hydrogen bond, pi-sulfur, pi-alkyl	−7.5
Caspase 9	2ar9	van der Waals, conventional hydrogen bond, unfavorable acceptor- acceptor, pi-cation, pi-donor hydrogen bond, pi-alkyl	−7.3
Caspase 8	3kjn	van der Waals, conventional hydrogen bond, unfavorable donor-donor, pi-cation, pi-donor hydrogen bond, pi-alkyl	−7.0
BAX	4bd6	van der Waals, pi-sigma, pi-pi T-shaped, pi-alkyl	−6.8
Bcl2	1ysw	van der Waals, conventional hydrogen bond, unfavorable donor-donor, pi-cation, pi-anion, pi-pi shaped, pi-alkyl	−7.2

## Data Availability

All data generated or analysed during this study are included in this article. Further enquiries can be directed to the corresponding author.

## Supplementary Materials

Supplementary Materials Supplementary Table 1: Active compounds recognized in SHCJ. Supplementary Table 2: Targets of active compounds in SHCJ. Supplementary Table 3: Related target genes for AD obtained from public databases. Supplementary Table 4: Hub target genes among targets of active compounds and AD-related targets. Supplementary Table 5: Molecular docking analysis of active compounds recognized in SHCJ and core target genes related to AD.
