# Supplementary material for: Pharmacological Mechanism of Shen Huang Chong Ji for Treating Alzheimer's Disease Based on Network Pharmacology and Experimental Validation
Source: Evid Based Complement Alternat Med. 2022 May 24;2022:9243348. doi: 10.1155/2022/9243348 (PMC9155915; doi:10.1155/2022/9243348)
Supplement: Supplementary Materials — Supplementary Table 1: Active compounds recognized in SHCJ. Supplementary Table 2: Targets of active compounds in SHCJ. Supplementary Table 3: Related target genes for AD obtained from public databases. Supplementary Table 4: Hub target genes among targets of active compounds and AD-related targets. Supplementary Table 5: Molecular docking analysis of active compounds recognized in SHCJ and core target genes related to AD. [file 9243348.f1.pdf]

Supplementary Table 1

Active Compounds Recognized in SHCJ

Herb	Molecule Name
Ginseng Radix et Rhizoma	Loxanol V
Ginseng Radix et Rhizoma	Pisol
Ginseng Radix et Rhizoma	9-HEXADECENOIC ACID
Ginseng Radix et Rhizoma	Methyl myristate
Ginseng Radix et Rhizoma	PENTADECYLIC ACID
Ginseng Radix et Rhizoma	METHYL LINOLEATE
Ginseng Radix et Rhizoma	2,6-dimethyl-3,7-octadiene-2,6-diol
Ginseng Radix et Rhizoma	MLI
Ginseng Radix et Rhizoma	Methyl stearate
Ginseng Radix et Rhizoma	Methyl palmitelaidate
Ginseng Radix et Rhizoma	METHYL PENTADECANOATE
Ginseng Radix et Rhizoma	panaxynol
Ginseng Radix et Rhizoma	Dauricine (8CI)
Ginseng Radix et Rhizoma	(1S,4E,8E,10R)-4,8,11,11-tetramethylbicyclo[8.1.0]undeca-4,8-diene
Ginseng Radix et Rhizoma	neocnidilide
Ginseng Radix et Rhizoma	OCT
Ginseng Radix et Rhizoma	20-Hexadecanoylingenol
Ginseng Radix et Rhizoma	[(3S,4R,5R)-5-[[(2R,3S,4S,5R,6S)-6-(2-acetyl-5-methoxyphenoxy)-3,4,5-trihydroxyoxan-2-yl]methoxy]-3,4-dihydroxyoxolan-3-yl]methyl 3,4,5-trihydroxybenzoate
Ginseng Radix et Rhizoma	L-Adenosine
Ginseng Radix et Rhizoma	Kaempferol-3-arabofuranoside
Ginseng Radix et Rhizoma	3691-11-0
Ginseng Radix et Rhizoma	Campesteryl ferulate
Ginseng Radix et Rhizoma	Elemicin
Ginseng Radix et Rhizoma	Diop
Ginseng Radix et Rhizoma	beta-Humulene
Ginseng Radix et Rhizoma	Stigmasterol
Ginseng Radix et Rhizoma	Psuedohypericin
Ginseng Radix et Rhizoma	beta-sitosterol
Ginseng Radix et Rhizoma	beta-caryophyllene
Ginseng Radix et Rhizoma	Inermin
Ginseng Radix et Rhizoma	Folinic acid
Ginseng Radix et Rhizoma	methyl (Z)-icos-11-enoate
Ginseng Radix et Rhizoma	N-Salicylidene-salicylamine
Ginseng Radix et Rhizoma	epsilon-Cadinene
Ginseng Radix et Rhizoma	kaempferol
Ginseng Radix et Rhizoma	δ-elemene
Ginseng Radix et Rhizoma	(1R,4E,7E,11R)-1,5,9,9-tetramethyl-12-oxabicyclo[9.1.0]dodeca-4,7-diene
Ginseng Radix et Rhizoma	Chrysanthemaxanthin
Ginseng Radix et Rhizoma	12-O-Nicotinoylisolineolone
Ginseng Radix et Rhizoma	TDA
Ginseng Radix et Rhizoma	ginsenoside Ro_qt
Ginseng Radix et Rhizoma	(+)-Maalioxide
Ginseng Radix et Rhizoma	n-Heptadecanol
Ginseng Radix et Rhizoma	1-HEXADECYNE
Ginseng Radix et Rhizoma	13-Tetradecenyl acetate
Ginseng Radix et Rhizoma	16-Oxoseratenediol
Ginseng Radix et Rhizoma	Neohexane
Ginseng Radix et Rhizoma	2,3,4-Trimethyldecane
Ginseng Radix et Rhizoma	2,3,8-Trimethyldecane
Ginseng Radix et Rhizoma	2,6,10,15-tetramethylheptadecane
Ginseng Radix et Rhizoma	2-METHYLTRIDECANE

Ginseng Radix et Rhizoma	ginsenoside-Rh1
Ginseng Radix et Rhizoma	ginsenoside-Rh1_qt
Ginseng Radix et Rhizoma	20(S)-Ginsenoside-Rh1
Ginseng Radix et Rhizoma	20(S)-Ginsenoside-Rh1_qt
Ginseng Radix et Rhizoma	20(S)-ginsenoside-Rg2
Ginseng Radix et Rhizoma	(3S,5R,6S,8R,9R,10R,12R,13R,14R,17S)-17-[(2S)-2-hydroxy-6-methylhept-5-en-2-yl]-4,4,8,10,14-pentamethyl-2,3,5,6,7,9,11,12,13,15,16,17-dodecahydro-1H-cyclopenta[a]phenanthrene-3,6,12-triol
Ginseng Radix et Rhizoma	20(s)-protopanaxadiol
Ginseng Radix et Rhizoma	20(R)-ginsenoside Rg2
Ginseng Radix et Rhizoma	20-(S)-Ginsenoside-Rg3
Ginseng Radix et Rhizoma	20-(S)-Ginsenoside-Rg3_qt
Ginseng Radix et Rhizoma	3,4-Dimethylheptane
Ginseng Radix et Rhizoma	3,5-Dimethyl-p-anisic acid
Ginseng Radix et Rhizoma	3-O-beta-D-Glucuronopyranosyl gypsogenin
Ginseng Radix et Rhizoma	3-O-beta-D-Glucuronopyranosyl gypsogenin_qt
Ginseng Radix et Rhizoma	3-Ethyl-3-methylheptane
Ginseng Radix et Rhizoma	3-methylheptane
Ginseng Radix et Rhizoma	3-methylundecane
Ginseng Radix et Rhizoma	4-Methyldodecane
Ginseng Radix et Rhizoma	5-Isobutylnonane
Ginseng Radix et Rhizoma	5-heptadec-12-enylresorcinol
Ginseng Radix et Rhizoma	5-methyl-tetradecane
Ginseng Radix et Rhizoma	6'-Malonylginsenoside Rd1
Ginseng Radix et Rhizoma	6'-Malonylginsenoside Rd1_qt1
Ginseng Radix et Rhizoma	7-(beta-Xylosyl)cephalomannine
Ginseng Radix et Rhizoma	7-Tetradecyne
Ginseng Radix et Rhizoma	7alpha-L-Rhamnosyl-6-methoxylutcolin
Ginseng Radix et Rhizoma	Nepetin
Ginseng Radix et Rhizoma	Acetal
Ginseng Radix et Rhizoma	Adenosine triphosphate
Ginseng Radix et Rhizoma	Aposiopolamine
Ginseng Radix et Rhizoma	Araloside A
Ginseng Radix et Rhizoma	(4aS,6aR,6aS,6bR,8aR,10S,12aR,14bR)-10-hydroxy-2,2,6a,6b,9,9,12a-heptamethyl-1,3,4,5,6,6a,7,8,8a,10,11,12,13,14b-tetradecahydropicene-4a-carboxylic acid
Ginseng Radix et Rhizoma	Argininyl-fructosyl-glucose
Ginseng Radix et Rhizoma	Argininyl-fructosyl-glucose_qt
Ginseng Radix et Rhizoma	5-[(3aS,6R,6aR)-2-keto-1,3,3a,4,6,6a-hexahydrothieno[3,4-d]imidazol-6-yl]valeric acid
Ginseng Radix et Rhizoma	Celabenzine
Ginseng Radix et Rhizoma	(R)-()-Citronellal
Ginseng Radix et Rhizoma	MAV
Ginseng Radix et Rhizoma	Deoxyharringtonine
Ginseng Radix et Rhizoma	Dianthramine
Ginseng Radix et Rhizoma	Ditertbutyl phthalate
Ginseng Radix et Rhizoma	arachidonate
Ginseng Radix et Rhizoma	Frutinone A
Ginseng Radix et Rhizoma	Gamma-Selinene
Ginseng Radix et Rhizoma	ginsenoside La
Ginseng Radix et Rhizoma	ginsenoside La_qt
Ginseng Radix et Rhizoma	ginsenoside Ro
Ginseng Radix et Rhizoma	Ginsenoside-Ra0
Ginseng Radix et Rhizoma	Gypnoside V_qt
Ginseng Radix et Rhizoma	Ginsenoside-Ra1
Ginseng Radix et Rhizoma	Ginsenoside-Ra2

Ginseng Radix et Rhizoma	Ginsenoside-Ra3
Ginseng Radix et Rhizoma	gingsenoside Rb1
Ginseng Radix et Rhizoma	(3R,5R,8R,9R,10R,12R,13R,14R,17S)-17-[(2S)-2-hydroxy-6-methylhept-5-en-2-yl]-4,4,8,10,14-pentamethyl-2,3,5,6,7,9,11,12,13,15,16,17-dodecahydro-1H-cyclopenta[a]phenanthrene-3,12-diol
Ginseng Radix et Rhizoma	ginsenoside-Rb2
Ginseng Radix et Rhizoma	(3S,5R,8R,9R,10R,12R,13R,14R,17S)-17-[(2S)-2-hydroxy-6-methylhept-5-en-2-yl]-4,4,8,10,14-pentamethyl-2,3,5,6,7,9,11,12,13,15,16,17-dodecahydro-1H-cyclopenta[a]phenanthrene-3,12-diol
Ginseng Radix et Rhizoma	Gypenoside LXIX
Ginseng Radix et Rhizoma	ginsenoside-Rc
Ginseng Radix et Rhizoma	(2S,3R,4S,5S,6R)-2-[(2S)-2-[(3S,5R,8R,9R,10R,12R,13R,14R,17S)-3-[(2R,3R,4S,5S,6R)-4,5-dihydroxy-6-(hydroxymethyl)-3-[(2S,3R,4S,5S,6R)-3,4,5-trihydroxy-6-(hydroxymethyl)oxan-2-yl]oxyoxan-2-yl]oxy-12-hydroxy-4,4,8,10,14-pentamethyl-2,3,5,6,7,9,11,12,13,15,1
Ginseng Radix et Rhizoma	Ginsenoside Re
Ginseng Radix et Rhizoma	ginsenoside rf
Ginseng Radix et Rhizoma	(3S,5R,6S,8R,9R,10R,12R,13R,14R,17S)-17-[(2R)-2-hydroxy-6-methylhept-5-en-2-yl]-4,4,8,10,14-pentamethyl-2,3,5,6,7,9,11,12,13,15,16,17-dodecahydro-1H-cyclopenta[a]phenanthrene-3,6,12-triol
Ginseng Radix et Rhizoma	Sanchinoside C1
Ginseng Radix et Rhizoma	Ginsenoside-Rg3
Ginseng Radix et Rhizoma	Ginsenoside-Rg3_qt
Ginseng Radix et Rhizoma	ginsenoside rh2
Ginseng Radix et Rhizoma	(2R,3S,4S,5R,6R)-2-(hydroxymethyl)-6-[[(3S,5R,8R,9R,10R,12R,13R,14R,17S)-12-hydroxy-4,4,8,10,14-pentamethyl-17-[(2Z)-6-methylhepta-2,5-dien-2-yl]-2,3,5,6,7,9,11,12,13,15,16,17-dodecahydro-1H-cyclopenta[a]phenanthren-3-yl]oxy]oxane-3,4,5-triol
Ginseng Radix et Rhizoma	Ginsenoside-Rh3_qt
Ginseng Radix et Rhizoma	Ginsenoside-Rh4
Ginseng Radix et Rhizoma	Ginsenoside-Rh4_qt
Ginseng Radix et Rhizoma	Ginsenoside-Rs1
Ginseng Radix et Rhizoma	Ginsenoside-Rs2
Ginseng Radix et Rhizoma	Ginsenoyne A
Ginseng Radix et Rhizoma	Ginsenoyne B
Ginseng Radix et Rhizoma	Ginsenoyne C
Ginseng Radix et Rhizoma	Ginsenoyne D
Ginseng Radix et Rhizoma	Ginsenoyne E
Ginseng Radix et Rhizoma	Girinimbin
Ginseng Radix et Rhizoma	Gomisin B
Ginseng Radix et Rhizoma	L-erythro-isocitric acid
Ginseng Radix et Rhizoma	D-erythro-Isocitric acid
Ginseng Radix et Rhizoma	malkangunin
Ginseng Radix et Rhizoma	Malonylginsenoside Rc
Ginseng Radix et Rhizoma	Malonylginsenoside Rc_qt1
Ginseng Radix et Rhizoma	Malonylginsenoside Rd
Ginseng Radix et Rhizoma	Malonylginsenoside Rd_qt
Ginseng Radix et Rhizoma	MAL

Ginseng Radix et Rhizoma	Malvic acid
Ginseng Radix et Rhizoma	GUP
Ginseng Radix et Rhizoma	Methyl tricosanoate
Ginseng Radix et Rhizoma	Mycosinol
Ginseng Radix et Rhizoma	NN-Dimethyldecanamide
Ginseng Radix et Rhizoma	Nonacosanediol-6,8
Ginseng Radix et Rhizoma	notoginsenoside R2
Ginseng Radix et Rhizoma	notoginsenoside R2_qt
Ginseng Radix et Rhizoma	Notoginsenoside R6
Ginseng Radix et Rhizoma	Stearyl acetate
Ginseng Radix et Rhizoma	Panaxadiol
Ginseng Radix et Rhizoma	panaxatriol
Ginseng Radix et Rhizoma	Panaxytriol
Ginseng Radix et Rhizoma	Pancratistatin
Ginseng Radix et Rhizoma	Pandamine
Ginseng Radix et Rhizoma	2-Formylpyrrole
Ginseng Radix et Rhizoma	Ramalic acid
Ginseng Radix et Rhizoma	Methylselenocysteine
Ginseng Radix et Rhizoma	suchilactone
Ginseng Radix et Rhizoma	Suffruticoside A_qt1
Ginseng Radix et Rhizoma	Vulgarin
Ginseng Radix et Rhizoma	Trifolirhizin
Ginseng Radix et Rhizoma	Undecane, 3,6-dimethyl
Ginseng Radix et Rhizoma	PANGAMIC ACID
Ginseng Radix et Rhizoma	3-[[(2S)-2,4-dihydroxy-3,3-dimethylbutanoyl]amino]propanoic acid
Ginseng Radix et Rhizoma	(Z,Z)-alpha-farnesene
Ginseng Radix et Rhizoma	alpha-Guttiferin
Ginseng Radix et Rhizoma	beta-elemene
Ginseng Radix et Rhizoma	(Z)-2-methyl-5-[(1S,2R,4R)-2-methyl-3-methylene-2-norbornanyl]pent-2-en-1-ol
Ginseng Radix et Rhizoma	beta-Selinene
Ginseng Radix et Rhizoma	cis-Widdrol alpha-epoxide
Ginseng Radix et Rhizoma	Dammarane
Ginseng Radix et Rhizoma	alexandrin
Ginseng Radix et Rhizoma	alexandrin_qt
Ginseng Radix et Rhizoma	ginsenoside Rg5
Ginseng Radix et Rhizoma	ginsenoside Rg5_qt
Ginseng Radix et Rhizoma	Methyl margarate
Ginseng Radix et Rhizoma	oleanane
Ginseng Radix et Rhizoma	p-Glucosyloxymandelonitrile
Ginseng Radix et Rhizoma	darutoside
Ginseng Radix et Rhizoma	alloaromadedrene
Ginseng Radix et Rhizoma	DBP
Ginseng Radix et Rhizoma	palmitic acid
Ginseng Radix et Rhizoma	Linoleic
Ginseng Radix et Rhizoma	Fumarine
Ginseng Radix et Rhizoma	MYS
Ginseng Radix et Rhizoma	paeonol
Ginseng Radix et Rhizoma	methyl palmitate
Ginseng Radix et Rhizoma	tetradecane
Ginseng Radix et Rhizoma	Hepanal
Ginseng Radix et Rhizoma	(1R,4S,4aR,8aR)-4-isopropyl-1,6-dimethyl-3,4,4a,7,8,8a-hexahydro-2H-naphthalen-1-ol
Ginseng Radix et Rhizoma	beta-Bisabolene
Polygonati Rhizoma	isoliquiritigenin
Polygonati Rhizoma	DFV
Polygonati Rhizoma	salicylic acid
Polygonati Rhizoma	baicalein

Polygonati Rhizoma	3'-Methoxydaidzein
Polygonati Rhizoma	succinic acid
Polygonati Rhizoma	Sitogluside
Polygonati Rhizoma	beta-sitosterol
Polygonati Rhizoma	sitosterol
Polygonati Rhizoma	methylprotodioscin
Polygonati Rhizoma	methylprotodioscin_qt
Polygonati Rhizoma	(+)-Syringaresinol
Polygonati Rhizoma	(Z)-1-(2,4-dihydroxyphenyl)-3-(4-hydroxyphenyl)prop-2-en-1-one
Polygonati Rhizoma	glucuronic acid
Polygonati Rhizoma	neoliquiritin
Polygonati Rhizoma	(2R)-7-hydroxy-2-(4-hydroxyphenyl)chroman-4-one
Polygonati Rhizoma	GUP
Polygonati Rhizoma	BGC
Polygonati Rhizoma	diosgenin
Polygonati Rhizoma	4',5-Dihydroxyflavone
Polygonati Rhizoma	4-[(1R,3aS,4R,6aS)-4-(4-hydroxy-3,5-dimethoxyphenyl)-1,3,3a,4,6,6a-hexahydrofuro[4,3-c]furan-1-yl]-2,6-dimethoxyphenol
Polygonati Rhizoma	HMF
Polygonati Rhizoma	apigenin
Polygonati Rhizoma	2',7-dihydroxy-3',4'-dimethoxyisoflavane
Polygonati Rhizoma	4-methylolfurfural
Polygonati Rhizoma	Oroxin A
Polygonati Rhizoma	2-Acridinecarboxylic acid
Polygonati Rhizoma	liriodendrin
Polygonati Rhizoma	n-butyl-β-D-fructopyranoside
Polygonati Rhizoma	2-Propen-1-one, 1-(2,4-dihydroxyphenyl)-3-(4-(beta-D-glucopyranosyloxy)phenyl)-, (2E)-
Polygonati Rhizoma	sibiricoside A
Polygonati Rhizoma	sibiricoside A_qt
Polygonati Rhizoma	sibiricoside B
Polygonati Rhizoma	sibiricoside B_qt
Polygonati Rhizoma	(+)-Syringaresinol-O-beta-D-glucoside
Polygonati Rhizoma	vitexin xyloside
Polygonati Rhizoma	(Z)-nonadec-6-enoic acid
Polygonati Rhizoma	zhonghualiaoine 1
Rehmanniae Radix Praeparata	Arachic acid
Rehmanniae Radix Praeparata	EIC
Rehmanniae Radix Praeparata	PENTADECYLIC ACID
Rehmanniae Radix Praeparata	leonuride
Rehmanniae Radix Praeparata	Daturic acid
Rehmanniae Radix Praeparata	acteoside
Rehmanniae Radix Praeparata	methyl (2E,4E)-hexadeca-2,4-dienoate
Rehmanniae Radix Praeparata	zoomaric acid
Rehmanniae Radix Praeparata	Sumiki's acid
Rehmanniae Radix Praeparata	Dihydro-beta-ionone
Rehmanniae Radix Praeparata	Pca
Rehmanniae Radix Praeparata	catalpol
Rehmanniae Radix Praeparata	catapol_qt
Rehmanniae Radix Praeparata	MTL
Rehmanniae Radix Praeparata	lauric acid
Rehmanniae Radix Praeparata	8-epi-Loganic acid
Rehmanniae Radix Praeparata	Forsythiaside
Rehmanniae Radix Praeparata	succinic acid
Rehmanniae Radix Praeparata	Sitogluside

Rehmanniae Radix Praeparata	sitosterol
Rehmanniae Radix Praeparata	FER
Rehmanniae Radix Praeparata	2-(4-hydroxyphenyl)ethyl hexacosanoate
Rehmanniae Radix Praeparata	aeginetic acid
Rehmanniae Radix Praeparata	Ajugol
Rehmanniae Radix Praeparata	Ajugoside
Rehmanniae Radix Praeparata	Ajugoside_qt
Rehmanniae Radix Praeparata	Cerebrosid
Rehmanniae Radix Praeparata	Cistanoside A
Rehmanniae Radix Praeparata	Cistanoside F
Rehmanniae Radix Praeparata	methyl 9,10-methylene-hexadecanoate
Rehmanniae Radix Praeparata	(2S,3R,4R,5S,6R)-2-[[(1S,4aS,5R,7aR)-4a,5-dihydroxy-7-methylol-5,7a-dihydro-1H-cyclopenta[c]pyran-1-yl]oxy]-6-methylol-tetrahydropyran-3,4,5-triol
Rehmanniae Radix Praeparata	melittoside_qt
Rehmanniae Radix Praeparata	Dihydrocatalpol
Rehmanniae Radix Praeparata	geniposide
Rehmanniae Radix Praeparata	geniposide_qt
Rehmanniae Radix Praeparata	glutinoside
Rehmanniae Radix Praeparata	Jiofuran
Rehmanniae Radix Praeparata	jioglutin A
Rehmanniae Radix Praeparata	jioglutin B
Rehmanniae Radix Praeparata	jioglutin C
Rehmanniae Radix Praeparata	jioglutin D
Rehmanniae Radix Praeparata	jioglutin E
Rehmanniae Radix Praeparata	jioglutolide
Rehmanniae Radix Praeparata	Jioglutoside A
Rehmanniae Radix Praeparata	methyl (1S,4aS,7aS)-7-methylene-1-[(2S,3R,4S,5S,6R)-3,4,5-trihydroxy-6-[[(2R,3R,4R,5S,6R)-3,4,5-trihydroxy-6-methyl-tetrahydropyran-2-yl]oxymethyl]tetrahydropyran-2-yl]oxy-4a,5,6,7a-tetrahydro-1H-cyclopenta[d]pyran-4-carboxylate
Rehmanniae Radix Praeparata	Jioglutoside B_qt
Rehmanniae Radix Praeparata	Jionoside A
Rehmanniae Radix Praeparata	Jionoside B
Rehmanniae Radix Praeparata	Melittoside
Rehmanniae Radix Praeparata	METHYL PALMITOLEATE
Rehmanniae Radix Praeparata	6-O-p-coumaroylajugol
Rehmanniae Radix Praeparata	methyl-2,6,10-trimethyltridecanoate
Rehmanniae Radix Praeparata	Purpureaside C
Rehmanniae Radix Praeparata	Rehmaglutin B
Rehmanniae Radix Praeparata	(3aS,4R,6aS)-4-hydroxy-6,6a-dimethylol-3a,4-dihydro-3H-cyclopenta[d]furan-2-one
Rehmanniae Radix Praeparata	(2S,3R,4S,5S,6R)-2-[(1R,2R)-2-hydroxy-2-[(E,3S)-3-hydroxybut-1-enyl]-1,3,3-trimethylcyclohexoxy]-6-(hydroxymethyl)tetrahydropyran-3,4,5-triol
Rehmanniae Radix Praeparata	(2S,3R,4S,5S,6R)-2-[(1R,2R)-2-hydroxy-2-[(E,3R)-3-hydroxybut-1-enyl]-1,3,3-trimethylcyclohexoxy]-6-(hydroxymethyl)tetrahydropyran-3,4,5-triol
Rehmanniae Radix Praeparata	Rehmaionoside C
Rehmanniae Radix Praeparata	rehmannioside B
Rehmanniae Radix Praeparata	rehmannioside C
Rehmanniae Radix Praeparata	6-O-p-hydroxybenzoylajugol
Rehmanniae Radix Praeparata	(3R)-2,6,6-trimethyl-3-[(2R,3R,4S,5S,6R)-3,4,5-trihydroxy-6-(hydroxymethyl)oxan-2-yl]oxycyclohexene-1-carboxylic acid

Rehmanniae Radix Praeparata	Rehmannioside A
Rehmanniae Radix Praeparata	Rehmaglutin A
Rehmanniae Radix Praeparata	Rehmaglutin D
Rehmanniae Radix Praeparata	6-O-vanilloylajugol
Rehmanniae Radix Praeparata	echinacoside
Rehmanniae Radix Praeparata	aucubin
Rehmanniae Radix Praeparata	Acetylcatalpol
Rehmanniae Radix Praeparata	Docosanoate
Rehmanniae Radix Praeparata	Caffeate
Rehmanniae Radix Praeparata	Stigmasterol
Rehmanniae Radix Praeparata	Stachyose
Rehmanniae Radix Praeparata	HMF
Rehmanniae Radix Praeparata	raffinose
Rehmanniae Radix Praeparata	sucrose
Epimedii Folium	(L)-alpha-Terpineol
Epimedii Folium	dec-2-enal
Epimedii Folium	CAM
Epimedii Folium	24-epicampesterol
Epimedii Folium	germacrene
Epimedii Folium	copaene
Epimedii Folium	NON
Epimedii Folium	Linoleyl acetate
Epimedii Folium	24190-29-2
Epimedii Folium	poriferast-5-en-3beta-ol
Epimedii Folium	isoliquiritigenin
Epimedii Folium	DFV
Epimedii Folium	Pulegone
Epimedii Folium	(R)-linalool
Epimedii Folium	Izosafrol
Epimedii Folium	(1S,4R)-fenchone
Epimedii Folium	(6R)-6-isopropyl-3-methyl-1-cyclohex-2-enone
Epimedii Folium	Methyleugenol
Epimedii Folium	tricin
Epimedii Folium	alpha-Cubebene
Epimedii Folium	20-Hexadecanoylingenol
Epimedii Folium	Terragon
Epimedii Folium	()-Borneol
Epimedii Folium	Ginkgetin
Epimedii Folium	Isoginkgetin
Epimedii Folium	oleanolic acid
Epimedii Folium	3,4,5-Trimethoxytoluene
Epimedii Folium	junipene
Epimedii Folium	magnoflorine
Epimedii Folium	salidroside
Epimedii Folium	Tyrosol
Epimedii Folium	Ginnol
Epimedii Folium	Chryseriol
Epimedii Folium	lauric acid
Epimedii Folium	Flavone der.
Epimedii Folium	(2R,3R)-2-(3,4-dimethoxyphenyl)-7-methoxy-3-methyl-5-[(E)-prop-1-enyl]-2,3-dihydrobenzofuran
Epimedii Folium	Vetol
Epimedii Folium	Damascenone
Epimedii Folium	8-Isopentenyl-kaempferol
Epimedii Folium	Azaron
Epimedii Folium	Sitogluside
Epimedii Folium	sitosterol
Epimedii Folium	Docosanoate

Epimedii Folium	kaempferol
Epimedii Folium	(Z)-heptadec-3-ene
Epimedii Folium	3,5-Dimethoxytoluene
Epimedii Folium	Isomenthol
Epimedii Folium	Octyl formate
Epimedii Folium	olivil
Epimedii Folium	Hyperin
Epimedii Folium	quercetin-3-rhamnooside
Epimedii Folium	Hirsutrin
Epimedii Folium	Robinetin
Epimedii Folium	rouhuoside
Epimedii Folium	Sagittatoside A
Epimedii Folium	Anhydroicaritin
Epimedii Folium	sagittatoside B
Epimedii Folium	Trifolin
Epimedii Folium	wanepimedoside A
Epimedii Folium	wanepimedoside_qt
Epimedii Folium	Wushanicariin
Epimedii Folium	wushanicariin_qt
Epimedii Folium	C-Homoerythrinan, 1,6-didehydro-3,15,16-trimethoxy-, (3.beta.)-
Epimedii Folium	Besigomsin
Epimedii Folium	Yinyanghuo A
Epimedii Folium	Yinyanghuo B
Epimedii Folium	Yinyanghuo C
Epimedii Folium	Yinyanghuo D
Epimedii Folium	Yinyanghuo E
Epimedii Folium	Yixinoside A
Epimedii Folium	6-hydroxy-11,12-dimethoxy-2,2-dimethyl-1,8-dioxo-2,3,4,8-tetrahydro-1H-isochromeno[3,4-h]isoquinolin-2-ium
Epimedii Folium	3-Hexenyl-beta-glucopyranoside
Epimedii Folium	5,7,4'-trihydroxy8,3'-diprenylflavone
Epimedii Folium	8-(3-methylbut-2-enyl)-2-phenyl-chromone
Epimedii Folium	acuminatoside
Epimedii Folium	anhydroicaritin
Epimedii Folium	Anhydroicaritin-3-O-alpha-L-rhamnoside
Epimedii Folium	artonin U
Epimedii Folium	1,2-bis(4-hydroxy-3-methoxyphenyl)propan-1,3-diol
Epimedii Folium	baohuoside Ⅵ
Epimedii Folium	3,5,7-Trihydroxy-4'-methoxyl-8-prenylflavone-3-O-rhamnopyranoside
Epimedii Folium	Baohuoside VI
Epimedii Folium	Bilobanol
Epimedii Folium	bilobetin
Epimedii Folium	brevicornin
Epimedii Folium	caohuoside B
Epimedii Folium	caohuoside D
Epimedii Folium	3-[(2S,3R,4R,5R,6S)-4,5-dihydroxy-6-methyl-5-[(2S,3R,4S,5S,6R)-3,4,5-trihydroxy-6-methyl-tetrahydropyran-2-yl]oxy-tetrahydropyran-2-yl]oxy-5-hydroxy-2-(4-methoxyphenyl)-8-(3-methylbut-2-enyl)-7-[(2S,3R,4S,5S,6R)-3,4,5-trihydroxy-6-methylol-tetrahydropyran
Epimedii Folium	2,15-Hexadecanedione
Epimedii Folium	Epimedin B
Epimedii Folium	Epimedin C_qt
Epimedii Folium	Epimedin C

Epimedii Folium	epimedokoreanone A
Epimedii Folium	(2S,3S)-3,5-dihydroxy-2-(4-hydroxyphenyl)-8-(3-methylbut-2-enyl)-7-[(2S,3R,4S,5S,6R)-3,4,5-trihydroxy-6-methylol-tetrahydropyran-2-yl]oxy-chroman-4-one
Epimedii Folium	epimedoside C
Epimedii Folium	epimedoside D
Epimedii Folium	Epimedoside E
Epimedii Folium	epimedoside
Epimedii Folium	DOB
Epimedii Folium	globulol
Epimedii Folium	Hentriacontanol-6
Epimedii Folium	hexandraside D
Epimedii Folium	Hexandraside E
Epimedii Folium	hexandraside F
Epimedii Folium	Icaride A2
Epimedii Folium	Icariin
Epimedii Folium	Icariresinol
Epimedii Folium	Icariside A7
Epimedii Folium	3,4,6-trimethoxyphenanthrene-2,7-diol
Epimedii Folium	icariside C1
Epimedii Folium	icariside I
Epimedii Folium	icariside II
Epimedii Folium	4H-1-Benzopyran-4-one, 3-((6-deoxy-alpha-L-mennopyranosyl)oxy)-5,7-dihydroxy-2-(4-hydroxyphenyl)-8-(3-methyl-2-butenyl)-
Epimedii Folium	3-[(2S,3R,4S,5S,6R)-4,5-dihydroxy-6-methylol-3-[(2S,3R,4R,5R,6S)-3,4,5-trihydroxy-6-methyl-tetrahydropyran-2-yl]oxy-tetrahydropyran-2-yl]oxy-5,7-dihydroxy-2-(4-hydroxyphenyl)-8-(3-methylbut-2-enyl)chromone
Epimedii Folium	Ikarisoside C
Epimedii Folium	Ikarisoside F
Epimedii Folium	Ikshusterol
Epimedii Folium	Lespedin
Epimedii Folium	korepimedoside A
Epimedii Folium	korepimedoside B
Epimedii Folium	emodin
Epimedii Folium	Eucarvone
Epimedii Folium	Astragalin
Epimedii Folium	luteolin
Epimedii Folium	Magnograndiolide
Epimedii Folium	1-hexanol
Epimedii Folium	patchouli alcohol
Epimedii Folium	(+)-Cycloolivil
Epimedii Folium	apigenin
Epimedii Folium	quercetin
Polygoni Multiflori	3,4,3',5'-Tetrahydroxystilbene-3-Glucoside
Polygoni Multiflori	Polygoacetophenoside
Polygoni Multiflori	Tetrahydroxytaxadiene
Polygoni Multiflori	Questinol
Polygoni Multiflori	Guaijaverin
Polygoni Multiflori	Questin
Polygoni Multiflori	Gamma-Sitosterol
Polygoni Multiflori	Fetidine
Polygoni Multiflori	Physcion
Polygoni Multiflori	Chrysophanol-8-O-Beta-D-(6'-O-Galloyl)-Glucopyranoside
Polygoni Multiflori	Rhein
Polygoni Multiflori	Emodin

Polygoni Multiflori	Piceid
Polygoni Multiflori	Chrysophanol
Polygoni Multiflori	Polygodial
Polygoni Multiflori	Tricin
Polygoni Multiflori	N-Trans-Feruloyltyramine
Polygoni Multiflori	Citreorosein
Polygoni Multiflori	Rhein Diglucoside
Polygoni Multiflori	Chrysazin
Polygoni Multiflori	Resveratrol
Polygoni Multiflori	Polygalacic Acid
Polygoni Multiflori	Emodin Anthrone
Polygoni Multiflori	Chrysarobin
Polygoni Multiflori	Daucosterol

Supplementary Table 2

Targets of Active Compounds

MolId	MolName	Target	Symbol
MOL001525	Daucosterol	Progesterone receptor	PGR
MOL001525	Daucosterol	Nuclear receptor coactivator 2	NCOA2
MOL002268	Rhein	Prostaglandin G/H synthase 1	PTGS1
MOL002268	Rhein	Prostaglandin G/H synthase 2	PTGS2
MOL002268	Rhein	Heat shock protein HSP 90-alpha	HSP90AA1
MOL002268	Rhein	Phosphatidylinositol 4,5-bisphosphate 3-kinase catalytic subunit gamma isoform	PIK3CG
MOL002268	Rhein	Nuclear receptor coactivator 6	NCOA6
MOL002268	Rhein	Aldo-keto reductase family 1 member B10	AKR1B10
MOL002268	Rhein	Transcription factor AP-1	JUN
MOL002268	Rhein	Thymidylate synthase	TYMS
MOL002268	Rhein	Interleukin-13	IL13
MOL002268	Rhein	Peroxisome proliferator-activated receptor gamma	PPARG
MOL002268	Rhein	Thrombopoietin receptor	MPL
MOL002268	Rhein	Interleukin-1 beta	IL1B
MOL002268	Rhein	Prostaglandin G/H synthase 1	PTGS1
MOL002268	Rhein	Apolipoprotein A-II	APOA2
MOL002268	Rhein	Gamma-glutamyl hydrolase	GGH
MOL002268	Rhein	Prostaglandin G/H synthase 2	PTGS2
MOL002268	Rhein	Serine hydroxymethyltransferase	SHMT2
MOL002268	Rhein	Serpin B7	SERPINB7
MOL002268	Rhein	Arachidonate 5-lipoxygenase	ALOX5
MOL002268	Rhein	Plasminogen activator inhibitor 1	SERPINE1
MOL008647	rans-Feruloyltyra	Melatonin receptor type 1A	MTNR1A
MOL008647	rans-Feruloyltyra	Melatonin receptor type 1B	MTNR1B
MOL008647	rans-Feruloyltyra	5-hydroxytryptamine receptor 2C	HTR2C
MOL001792	DFV	Prostaglandin G/H synthase 1	PTGS1
MOL001792	DFV	Estrogen receptor	ESR1
MOL001792	DFV	Prostaglandin G/H synthase 2	PTGS2
MOL001792	DFV	Retinoic acid receptor RXR-alpha	RXRA
MOL001792	DFV	Beta-2 adrenergic receptor	ADRB2
MOL001792	DFV	Heat shock protein HSP 90-alpha	HSP90AA1
MOL001792	DFV	cAMP-dependent protein kinase catalytic subunit alpha	PRKACA
MOL001792	DFV	Amine oxidase [flavin-containing]	MAOB
MOL001792	DFV	Sodium-dependent serotonin transporter	SLC6A4
MOL001792	DFV	cAMP-dependent protein kinase inhibitor alpha	PKIA
MOL002714	baicalein	Prostaglandin G/H synthase 1	PTGS1
MOL002714	baicalein	Androgen receptor	AR
MOL002714	baicalein	Prostaglandin G/H synthase 2	PTGS2

MOL002714	baicalein	Heat shock protein HSP 90-alpha	HSP90AA1
MOL002714	baicalein	cAMP-dependent protein kinase catalytic subunit alpha	PRKACA
MOL002714	baicalein	Dipeptidyl peptidase 4	DPP4
MOL002714	baicalein	cGMP-inhibited 3',5'-cyclic phosphodiesterase A	PDE3A
MOL002714	baicalein	Trypsin-1	PRSS1
MOL002714	baicalein	Nuclear receptor coactivator 2	NCOA2
MOL002714	baicalein	Nuclear receptor coactivator 1	NCOA1
MOL002714	baicalein	Transcription factor p65	RELA
MOL002714	baicalein	RAC-alpha serine/threonine-protein kinase	AKT1
MOL002714	baicalein	Apoptosis regulator Bcl-2	BCL2
MOL002714	baicalein	Proto-oncogene c-Fos	FOS
MOL002714	baicalein	Apoptosis regulator BAX	BAX
MOL002714	baicalein	Matrix metalloproteinase-9	MMP9
MOL002714	baicalein	Caspase-3	CASP3
MOL002714	baicalein	Cellular tumor antigen p53	TP63
MOL002714	baicalein	Hypoxia-inducible factor 1-alpha	HIF1A
MOL002714	baicalein	Fos-related antigen 1	FOSL1
MOL002714	baicalein	Fos-related antigen 2	FOSL2
MOL002714	baicalein	G2/mitotic-specific cyclin-B1	CCNB1
MOL002714	baicalein	Myeloperoxidase	MPO
MOL002714	baicalein	Aryl hydrocarbon receptor	AHR
MOL002714	baicalein	Insulin-like growth factor II	IGF2
MOL002714	baicalein	Cytochrome c	CYCS
MOL002714	baicalein	Arachidonate 12-lipoxygenase, 12S-type	ALOX12
MOL002714	baicalein	Nuclear factor of activated T-cells, cytoplasmic 1	NFATC1
MOL002714	baicalein	Tudor domain-containing protein 7	TDRD7
MOL002714	baicalein	Egl nine homolog 1	EGLN1
MOL002714	baicalein	NADPH oxidase 5	NOX5
MOL002714	baicalein	Apolipoprotein D	APOD
MOL002959	'-Methoxydaidzei	Nitric oxide synthase, inducible	NOS2
MOL002959	'-Methoxydaidzei	Prostaglandin G/H synthase 1	PTGS1
MOL002959	'-Methoxydaidzei	Estrogen receptor	ESR1
MOL002959	'-Methoxydaidzei	Androgen receptor	AR
MOL002959	'-Methoxydaidzei	Peroxisome proliferator-activated receptor gamma	PPARG
MOL002959	'-Methoxydaidzei	Prostaglandin G/H synthase 2	PTGS2
MOL002959	'-Methoxydaidzei	Retinoic acid receptor RXR-alpha	RXRA
MOL002959	'-Methoxydaidzei	Estrogen receptor beta	ESR2
MOL002959	'-Methoxydaidzei	Mitogen-activated protein kinase 14	MAPK14
MOL002959	'-Methoxydaidzei	Glycogen synthase kinase-3 beta	GSK3B
MOL002959	'-Methoxydaidzei	Heat shock protein HSP 90-alpha	HSP90AA1
MOL002959	'-Methoxydaidzei	Serine/threonine-protein kinase	CHEK1
MOL002959	'-Methoxydaidzei	cAMP-dependent protein kinase catalytic subunit alpha	PRKACA
MOL002959	'-Methoxydaidzei	Trypsin-1	PRSS1
MOL002959	'-Methoxydaidzei	Cyclin-A2	CCNA2
MOL002959	'-Methoxydaidzei	Nuclear receptor coactivator 1	NCOA1
MOL000358	beta-sitosterol	Progesterone receptor	PGR
MOL000358	beta-sitosterol	Nuclear receptor coactivator 2	NCOA2
MOL000358	beta-sitosterol	Prostaglandin G/H synthase 1	PTGS1
MOL000358	beta-sitosterol	Prostaglandin G/H synthase 2	PTGS2
MOL000358	beta-sitosterol	Heat shock protein HSP 90-alpha	HSP90AA1
MOL000358	beta-sitosterol	Potassium voltage-gated channel subfamily H member 2	KCNH2

MOL000358	beta-sitosterol	cAMP-dependent protein kinase catalytic subunit alpha	PRKACA
MOL000358	beta-sitosterol	D(1A) dopamine receptor	DRD1
MOL000358	beta-sitosterol	Muscarinic acetylcholine receptor	CHRM3
MOL000358	beta-sitosterol	Muscarinic acetylcholine receptor	CHRM1
MOL000358	beta-sitosterol	Sodium channel protein type 5 subunit alpha	SCN5A
MOL000358	beta-sitosterol	Muscarinic acetylcholine receptor	CHRM4
MOL000358	beta-sitosterol	cGMP-inhibited 3',5'-cyclic phosphodiesterase A	PDE3A
MOL000358	beta-sitosterol	Alpha-1A adrenergic receptor	ADRA1A
MOL000358	beta-sitosterol	Muscarinic acetylcholine receptor	CHRM2
MOL000358	beta-sitosterol	Alpha-1B adrenergic receptor	ADRA1B
MOL000358	beta-sitosterol	Beta-2 adrenergic receptor	ADRB2
MOL000358	beta-sitosterol	Neuronal acetylcholine receptor subunit alpha-2	CHRNA2
MOL000358	beta-sitosterol	Sodium-dependent serotonin transporter	SLC6A4
MOL000358	beta-sitosterol	Mu-type opioid receptor	OPRM1
MOL000358	beta-sitosterol	Neuronal acetylcholine receptor subunit alpha-7	CHRNA7
MOL000358	beta-sitosterol	Apoptosis regulator Bcl-2	BCL2
MOL000358	beta-sitosterol	Apoptosis regulator BAX	BAX
MOL000358	beta-sitosterol	Caspase-9	CASP9
MOL000358	beta-sitosterol	Transcription factor AP-1	JUN
MOL000358	beta-sitosterol	Caspase-3	CASP3
MOL000358	beta-sitosterol	Caspase-8	CASP8
MOL000358	beta-sitosterol	Protein kinase C alpha type	PRKCA
MOL000358	beta-sitosterol	Serum paraoxonase/arylesterase 1	PON1
MOL000358	beta-sitosterol	Microtubule-associated protein 2	MAP2
MOL000359	sitosterol	Progesterone receptor	PGR
MOL000359	sitosterol	Nuclear receptor coactivator 2	NCOA2
MOL000359	sitosterol	Mineralocorticoid receptor	NR3C2
MOL004941	-(4-hydroxyphen	Prostaglandin G/H synthase 1	PTGS1
MOL004941	-(4-hydroxyphen	Estrogen receptor	ESR1
MOL004941	-(4-hydroxyphen	Prostaglandin G/H synthase 2	PTGS2
MOL004941	-(4-hydroxyphen	Retinoic acid receptor RXR-alpha	RXRA
MOL004941	-(4-hydroxyphen	cGMP-inhibited 3',5'-cyclic phosphodiesterase A	PDE3A
MOL004941	-(4-hydroxyphen	Beta-2 adrenergic receptor	ADRB2
MOL004941	-(4-hydroxyphen	Heat shock protein HSP 90-alpha	HSP90AA1
MOL004941	-(4-hydroxyphen	Amine oxidase [flavin-containing]	MAOB
MOL004941	-(4-hydroxyphen	cAMP-dependent protein kinase catalytic subunit alpha	PRKACA
MOL004941	-(4-hydroxyphen	cAMP-dependent protein kinase inhibitor alpha	PKIA
MOL004941	-(4-hydroxyphen	Sodium-dependent serotonin transporter	SLC6A4
MOL000546	diosgenin	Progesterone receptor	PGR
MOL000546	diosgenin	Mineralocorticoid receptor	NR3C2
MOL000546	diosgenin	Transcription factor p65	RELA
MOL000546	diosgenin	RAC-alpha serine/threonine-protein kinase	AKT1
MOL000546	diosgenin	Cyclin-dependent kinase inhibitor 1	CDKN1A
MOL000546	diosgenin	Cellular tumor antigen p53	TP63
MOL000546	diosgenin	Prostaglandin G/H synthase 2	PTGS2
MOL000546	diosgenin	Fatty acid synthase	FASN
MOL000546	diosgenin	Superoxide dismutase [Cu-Zn]	SOD1
MOL000546	diosgenin	Hypoxia-inducible factor 1-alpha	HIF1A

MOL000546	diosgenin	Nuclear receptor subfamily 1 group I member 2	NR1I2
MOL000546	diosgenin	Cytosolic phospholipase A2	PLA2G4A
MOL000546	diosgenin	Canalicular multispecific organic anion transporter 1	ABCC2
MOL000546	diosgenin	Serine/threonine-protein kinase mTOR	MTOR
MOL006331	5-Dihydroxyflavo	Prostaglandin G/H synthase 1	PTGS1
MOL006331	5-Dihydroxyflavo	Androgen receptor	AR
MOL006331	5-Dihydroxyflavo	Prostaglandin G/H synthase 2	PTGS2
MOL006331	5-Dihydroxyflavo	Dipeptidyl peptidase 4	DPP4
MOL006331	5-Dihydroxyflavo	Heat shock protein HSP 90-alpha	HSP90AA1
MOL006331	5-Dihydroxyflavo	Amine oxidase [flavin-containing]	MAOB
MOL006331	5-Dihydroxyflavo	cAMP-dependent protein kinase catalytic subunit alpha	PRKACA
MOL009763	aresinol-O-beta-D	DNA topoisomerase 2-alpha	TOP2A
MOL002879	Diop	Sodium channel protein type 5 subunit alpha	SCN5A
MOL002879	Diop	Beta-2 adrenergic receptor	ADRB2
MOL002879	Diop	Muscarinic acetylcholine receptor	CHRM3
MOL000449	Stigmasterol	Progesterone receptor	PGR
MOL000449	Stigmasterol	Mineralocorticoid receptor	NR3C2
MOL000449	Stigmasterol	Nuclear receptor coactivator 2	NCOA2
MOL000449	Stigmasterol	Retinoic acid receptor RXR-alpha	RXRA
MOL000449	Stigmasterol	Nuclear receptor coactivator 1	NCOA1
MOL000449	Stigmasterol	Prostaglandin G/H synthase 1	PTGS1
MOL000449	Stigmasterol	Prostaglandin G/H synthase 2	PTGS2
MOL000449	Stigmasterol	Alpha-2A adrenergic receptor	ADRA2A
MOL000449	Stigmasterol	Sodium-dependent noradrenaline transporter	SLC6A2
MOL000449	Stigmasterol	Sodium-dependent dopamine transporter	SLC6A3
MOL000449	Stigmasterol	Beta-2 adrenergic receptor	ADRB2
MOL000449	Stigmasterol	Aldose reductase	AKR1B1
MOL000449	Stigmasterol	Urokinase-type plasminogen	PLAU
MOL000449	Stigmasterol	Leukotriene A-4 hydrolase	LTA4H
MOL000449	Stigmasterol	Amine oxidase [flavin-containing]	MAOB
MOL000449	Stigmasterol	Amine oxidase [flavin-containing]	MAOA
MOL000449	Stigmasterol	cAMP-dependent protein kinase catalytic subunit alpha	PRKACA
MOL000449	Stigmasterol	Chymotrypsinogen B	CTRB1
MOL000449	Stigmasterol	Muscarinic acetylcholine receptor	CHRM3
MOL000449	Stigmasterol	Muscarinic acetylcholine receptor	CHRM1
MOL000449	Stigmasterol	Beta-1 adrenergic receptor	ADRB1
MOL000449	Stigmasterol	Sodium channel protein type 5 subunit alpha	SCN5A
MOL000449	Stigmasterol	Alpha-1A adrenergic receptor	ADRA1A
MOL000449	Stigmasterol	Muscarinic acetylcholine receptor	CHRM2
MOL000449	Stigmasterol	Alpha-1B adrenergic receptor	ADRA1B
MOL000449	Stigmasterol	Neuronal acetylcholine receptor subunit alpha-7	CHRNA7
MOL000358	beta-sitosterol	Progesterone receptor	PGR
MOL000358	beta-sitosterol	Nuclear receptor coactivator 2	NCOA2
MOL000358	beta-sitosterol	Prostaglandin G/H synthase 1	PTGS1
MOL000358	beta-sitosterol	Prostaglandin G/H synthase 2	PTGS2
MOL000358	beta-sitosterol	Heat shock protein HSP 90-alpha	HSP90AA1
MOL000358	beta-sitosterol	Potassium voltage-gated channel subfamily H member 2	KCNH2
MOL000358	beta-sitosterol	cAMP-dependent protein kinase catalytic subunit alpha	PRKACA

MOL000358	beta-sitosterol	D(1A) dopamine receptor	DRD1
MOL000358	beta-sitosterol	Muscarinic acetylcholine receptor	CHRM3
MOL000358	beta-sitosterol	Muscarinic acetylcholine receptor	CHRM1
MOL000358	beta-sitosterol	Sodium channel protein type 5 subunit alpha	SCN5A
MOL000358	beta-sitosterol	Muscarinic acetylcholine receptor	CHRM4
MOL000358	beta-sitosterol	cGMP-inhibited 3',5'-cyclic phosphodiesterase A	PDE3A
MOL000358	beta-sitosterol	Alpha-1A adrenergic receptor	ADRA1A
MOL000358	beta-sitosterol	Muscarinic acetylcholine receptor	CHRM2
MOL000358	beta-sitosterol	Alpha-1B adrenergic receptor	ADRA1B
MOL000358	beta-sitosterol	Beta-2 adrenergic receptor	ADRB2
MOL000358	beta-sitosterol	Neuronal acetylcholine receptor subunit alpha-2	CHRNA2
MOL000358	beta-sitosterol	Sodium-dependent serotonin transporter	SLC6A4
MOL000358	beta-sitosterol	Mu-type opioid receptor	OPRM1
MOL000358	beta-sitosterol	Neuronal acetylcholine receptor subunit alpha-7	CHRNA7
MOL000358	beta-sitosterol	Apoptosis regulator Bcl-2	BCL2
MOL000358	beta-sitosterol	Apoptosis regulator BAX	BAX
MOL000358	beta-sitosterol	Caspase-9	CASP9
MOL000358	beta-sitosterol	Transcription factor AP-1	JUN
MOL000358	beta-sitosterol	Caspase-3	CASP3
MOL000358	beta-sitosterol	Caspase-8	CASP8
MOL000358	beta-sitosterol	Protein kinase C alpha type	PRKCA
MOL000358	beta-sitosterol	Serum paraoxonase/arylesterase 1	PON1
MOL000358	beta-sitosterol	Microtubule-associated protein 2	MAP2
MOL003648	Inermin	Prostaglandin G/H synthase 1	PTGS1
MOL003648	Inermin	Muscarinic acetylcholine receptor	CHRM3
MOL003648	Inermin	Sodium channel protein type 5 subunit alpha	SCN5A
MOL003648	Inermin	Prostaglandin G/H synthase 2	PTGS2
MOL003648	Inermin	Retinoic acid receptor RXR-alpha	RXRA
MOL003648	Inermin	Alpha-1B adrenergic receptor	ADRA1B
MOL003648	Inermin	Beta-2 adrenergic receptor	ADRB2
MOL003648	Inermin	Alpha-1D adrenergic receptor	ADRA1D
MOL003648	Inermin	Sodium-dependent serotonin transporter	SLC6A4
MOL003648	Inermin	Heat shock protein HSP 90-alpha	HSP90AA1
MOL003648	Inermin	Neuronal acetylcholine receptor subunit alpha-7	CHRNA7
MOL003648	Inermin	cAMP-dependent protein kinase catalytic subunit alpha	PRKACA
MOL003648	Inermin	Trypsin-1	PRSS1
MOL003648	Inermin	Nuclear receptor coactivator 1	NCOA1
MOL000422	kaempferol	Nitric oxide synthase, inducible	NOS2
MOL000422	kaempferol	Prostaglandin G/H synthase 1	PTGS1
MOL000422	kaempferol	Androgen receptor	AR
MOL000422	kaempferol	Peroxisome proliferator-activated receptor gamma	PPARG
MOL000422	kaempferol	Prostaglandin G/H synthase 2	PTGS2
MOL000422	kaempferol	Heat shock protein HSP 90-alpha	HSP90AA1
MOL000422	kaempferol	cAMP-dependent protein kinase catalytic subunit alpha	PRKACA
MOL000422	kaempferol	Nuclear receptor coactivator 2	NCOA2
MOL000422	kaempferol	Dipeptidyl peptidase 4	DPP4
MOL000422	kaempferol	Trypsin-1	PRSS1
MOL000422	kaempferol	Progesterone receptor	PGR
MOL000422	kaempferol	Prothrombin	F2

MOL000422	kaempferol	Muscarinic acetylcholine receptor	CHRM1
MOL000422	kaempferol	Acetylcholinesterase	ACHE
MOL000422	kaempferol	Sodium-dependent noradrenaline transporter	SLC6A2
MOL000422	kaempferol	Muscarinic acetylcholine receptor	CHRM2
MOL000422	kaempferol	Alpha-1B adrenergic receptor	ADRA1B
MOL000422	kaempferol	DNA topoisomerase 2-alpha	TOP2A
MOL000422	kaempferol	Coagulation factor VII	F7
MOL000422	kaempferol	Transcription factor p65	RELA
MOL000422	kaempferol	Inhibitor of nuclear factor kappa-B kinase subunit beta	IKBKB
MOL000422	kaempferol	RAC-alpha serine/threonine-protein kinase	AKT1
MOL000422	kaempferol	Apoptosis regulator Bcl-2	BCL2
MOL000422	kaempferol	Apoptosis regulator BAX	BAX
MOL000422	kaempferol	Tumor necrosis factor	TNFSF15
MOL000422	kaempferol	Transcription factor AP-1	JUN
MOL000422	kaempferol	Activator of 90 kDa heat shock protein ATPase homolog 1	AHSA1
MOL000422	kaempferol	Caspase-3	CASP3
MOL000422	kaempferol	Mitogen-activated protein kinase 8	MAPK8
MOL000422	kaempferol	Interstitial collagenase	MMP1
MOL000422	kaempferol	Signal transducer and activator of transcription 1-alpha/beta	STAT1
MOL000422	kaempferol	Peroxisome proliferator-activated receptor gamma	PPARG
MOL000422	kaempferol	Heme oxygenase 1	HMOX1
MOL000422	kaempferol	Cytochrome P450 3A4	CYP3A4
MOL000422	kaempferol	Cytochrome P450 1A1	CYP1A1
MOL000422	kaempferol	Intercellular adhesion molecule 1	ICAM1
MOL000422	kaempferol	E-selectin	SELE
MOL000422	kaempferol	Vascular cell adhesion protein 1	VCAM1
MOL000422	kaempferol	Nuclear receptor subfamily 1 group I member 2	NR1I2
MOL000422	kaempferol	Cytochrome P450 1B1	CYP1B1
MOL000422	kaempferol	Arachidonate 5-lipoxygenase	ALOX5
MOL000422	kaempferol	Hyaluronan synthase 2	HAS2
MOL000422	kaempferol	Aryl hydrocarbon receptor	AHR
MOL000422	kaempferol	26S proteasome non-ATPase regulatory subunit 3	PSMD3
MOL000422	kaempferol	Solute carrier family 2, facilitated glucose transporter member 4	SLC2A4
MOL000422	kaempferol	Nuclear receptor subfamily 1 group I member 3	NR1I3
MOL000422	kaempferol	Insulin receptor	INSR
MOL000422	kaempferol	Type I iodothyronine deiodinase	DIO1
MOL000422	kaempferol	Serine/threonine-protein phosphatase 2B catalytic subunit	PPP3CA
MOL000422	kaempferol	Glutathione S-transferase Mu 1	GSTM1
MOL000422	kaempferol	Glutathione S-transferase Mu 2	GSTM2
MOL000422	kaempferol	Aldo-keto reductase family 1 member C3	AKR1C3
MOL000422	kaempferol	Antileukoproteinase	SLPI
MOL005308	Aposiopolamine	Muscarinic acetylcholine receptor	CHRM3
MOL005308	Aposiopolamine	Muscarinic acetylcholine receptor	CHRM1
MOL005308	Aposiopolamine	Sodium-dependent noradrenaline transporter	SLC6A2
MOL005308	Aposiopolamine	Sodium-dependent dopamine transporter	SLC6A3
MOL005308	Aposiopolamine	Beta-2 adrenergic receptor	ADRB2

MOL005308	Aposiopolamine	Sodium-dependent serotonin transporter	SLC6A4
MOL005308	Aposiopolamine	Dipeptidyl peptidase 4	DPP4
MOL005317	Deoxyharringtonin	Androgen receptor	AR
MOL005317	Deoxyharringtonin	Mineralocorticoid receptor	NR3C2
MOL005318	Dianthramine	Prostaglandin G/H synthase 1	PTGS1
MOL005318	Dianthramine	Prostaglandin G/H synthase 2	PTGS2
MOL005318	Dianthramine	Heat shock protein HSP 90-alpha	HSP90AA1
MOL005320	arachidonate	Prostaglandin G/H synthase 1	PTGS1
MOL005320	arachidonate	Prostaglandin G/H synthase 2	PTGS2
MOL005320	arachidonate	Retinoic acid receptor RXR-gamma	RXRG
MOL005320	arachidonate	Nuclear receptor coactivator 2	NCOA2
MOL005321	Frutinone A	Prostaglandin G/H synthase 1	PTGS1
MOL005321	Frutinone A	Prothrombin	F2
MOL005321	Frutinone A	Androgen receptor	AR
MOL005321	Frutinone A	Sodium channel protein type 5 subunit alpha	SCN5A
MOL005321	Frutinone A	Peroxisome proliferator-activated receptor gamma	PPARG
MOL005321	Frutinone A	Prostaglandin G/H synthase 2	PTGS2
MOL005321	Frutinone A	Retinoic acid receptor RXR-alpha	RXRA
MOL005321	Frutinone A	cGMP-inhibited 3',5'-cyclic phosphodiesterase A	PDE3A
MOL005321	Frutinone A	Beta-2 adrenergic receptor	ADRB2
MOL005321	Frutinone A	Dipeptidyl peptidase 4	DPP4
MOL005321	Frutinone A	Heat shock protein HSP 90-alpha	HSP90AA1
MOL005321	Frutinone A	Neuronal acetylcholine receptor subunit alpha-7	CHRNA7
MOL005321	Frutinone A	cAMP-dependent protein kinase catalytic subunit alpha	PRKACA
MOL005321	Frutinone A	Acetylcholinesterase	ACHE
MOL005344	ginsenoside rh2	Apoptosis regulator BAX	BAX
MOL005344	ginsenoside rh2	Tumor necrosis factor	TNFSF15
MOL005344	ginsenoside rh2	Caspase-3	CASP3
MOL005344	ginsenoside rh2	Prostaglandin G/H synthase 2	PTGS2
MOL005344	ginsenoside rh2	NF-kappa-B inhibitor alpha	NFKBIA
MOL005344	ginsenoside rh2	Interleukin-1 beta	IL1B
MOL005344	ginsenoside rh2	Caspase-1	CASP1
MOL005344	ginsenoside rh2	Interferon gamma	IFNG
MOL005344	ginsenoside rh2	Pituitary adenylate cyclase-activating polypeptide	ADCYAP1
MOL005344	ginsenoside rh2	Proteasome assembly chaperone 1	PSMG1
MOL005344	ginsenoside rh2	Dual specificity mitogen-activated protein kinase kinase 4	MAP2K4
MOL005344	ginsenoside rh2	Solute carrier family 2, facilitated glucose transporter member 4	SLC2A4
MOL005348	insenoside-Rh4_	Mineralocorticoid receptor	NR3C2
MOL005348	insenoside-Rh4_	Nuclear receptor coactivator 2	NCOA2
MOL005356	Girinimbin	Prostaglandin G/H synthase 1	PTGS1
MOL005356	Girinimbin	Sodium channel protein type 5 subunit alpha	SCN5A
MOL005356	Girinimbin	Prostaglandin G/H synthase 2	PTGS2
MOL005356	Girinimbin	Retinoic acid receptor RXR-alpha	RXRA
MOL005356	Girinimbin	Beta-2 adrenergic receptor	ADRB2
MOL005356	Girinimbin	Neuronal acetylcholine receptor subunit alpha-7	CHRNA7
MOL005356	Girinimbin	cAMP-dependent protein kinase catalytic subunit alpha	PRKACA
MOL005356	Girinimbin	Nuclear receptor coactivator 2	NCOA2
MOL005376	Panaxadiol	Glucocorticoid receptor	NR3C1

MOL005384	suchilactone	Potassium voltage-gated channel subfamily H member 2	KCNH2
MOL005384	suchilactone	Sodium channel protein type 5 subunit alpha	SCN5A
MOL005384	suchilactone	Coagulation factor X	F10
MOL005384	suchilactone	Prostaglandin G/H synthase 2	PTGS2
MOL005384	suchilactone	Coagulation factor VII	F7
MOL005384	suchilactone	Tyrosine-protein phosphatase non-receptor type 1	PTPN1
MOL005384	suchilactone	Beta-2 adrenergic receptor	ADRB2
MOL005384	suchilactone	Heat shock protein HSP 90-alpha	HSP90AA1
MOL005384	suchilactone	Nuclear receptor coactivator 1	NCOA1
MOL005384	suchilactone	Prostaglandin G/H synthase 1	PTGS1
MOL005384	suchilactone	Retinoic acid receptor RXR-alpha	RXRA
MOL005384	suchilactone	cGMP-inhibited 3',5'-cyclic phosphodiesterase A	PDE3A
MOL005384	suchilactone	Alpha-1D adrenergic receptor	ADRA1D
MOL005384	suchilactone	cAMP-dependent protein kinase catalytic subunit alpha	PRKACA
MOL005399	alexandrin_qt	Progesterone receptor	PGR
MOL000787	Fumarine	Prostaglandin G/H synthase 1	PTGS1
MOL000787	Fumarine	Muscarinic acetylcholine receptor	CHRM3
MOL000787	Fumarine	Potassium voltage-gated channel subfamily H member 2	KCNH2
MOL000787	Fumarine	Muscarinic acetylcholine receptor	CHRM1
MOL000787	Fumarine	Sodium channel protein type 5 subunit alpha	SCN5A
MOL000787	Fumarine	Coagulation factor X	F10
MOL000787	Fumarine	Muscarinic acetylcholine receptor	CHRM5
MOL000787	Fumarine	Prostaglandin G/H synthase 2	PTGS2
MOL000787	Fumarine	Coagulation factor VII	F7
MOL000787	Fumarine	Muscarinic acetylcholine receptor	CHRM4
MOL000787	Fumarine	Delta-type opioid receptor	OPRD1
MOL000787	Fumarine	Alpha-1B adrenergic receptor	ADRA1B
MOL000787	Fumarine	Beta-2 adrenergic receptor	ADRB2
MOL000787	Fumarine	Alpha-1D adrenergic receptor	ADRA1D
MOL000787	Fumarine	Mu-type opioid receptor	OPRM1
MOL000787	Fumarine	Heat shock protein HSP 90-alpha	HSP90AA1
MOL000787	Fumarine	cAMP-dependent protein kinase catalytic subunit alpha	PRKACA
MOL000787	Fumarine	Sodium-dependent serotonin transporter	SLC6A4
MOL000787	Fumarine	Voltage-dependent L-type calcium channel subunit alpha-1S	CACNA1S
MOL000787	Fumarine	cGMP-inhibited 3',5'-cyclic phosphodiesterase A	PDE3A
MOL000787	Fumarine	Sodium-dependent dopamine transporter	SLC6A3
MOL000787	Fumarine	cAMP-specific 3',5'-cyclic phosphodiesterase 4D	PDE4D
MOL000787	Fumarine	DNA topoisomerase 2-alpha	TOP2A
MOL000787	Fumarine	D(1A) dopamine receptor	DRD1
MOL000787	Fumarine	Vascular endothelial growth factor receptor 2	KDR
MOL000359	sitosterol	Progesterone receptor	PGR
MOL000359	sitosterol	Nuclear receptor coactivator 2	NCOA2
MOL000359	sitosterol	Mineralocorticoid receptor	NR3C2
MOL000449	Stigmasterol	Progesterone receptor	PGR
MOL000449	Stigmasterol	Mineralocorticoid receptor	NR3C2
MOL000449	Stigmasterol	Nuclear receptor coactivator 2	NCOA2

MOL000449	Stigmasterol	Retinoic acid receptor RXR-alpha	RXRA
MOL000449	Stigmasterol	Nuclear receptor coactivator 1	NCOA1
MOL000449	Stigmasterol	Prostaglandin G/H synthase 1	PTGS1
MOL000449	Stigmasterol	Prostaglandin G/H synthase 2	PTGS2
MOL000449	Stigmasterol	Alpha-2A adrenergic receptor	ADRA2A
MOL000449	Stigmasterol	Sodium-dependent noradrenaline transporter	SLC6A2
MOL000449	Stigmasterol	Sodium-dependent dopamine transporter	SLC6A3
MOL000449	Stigmasterol	Beta-2 adrenergic receptor	ADRB2
MOL000449	Stigmasterol	Aldose reductase	AKR1B1
MOL000449	Stigmasterol	Urokinase-type plasminogen	PLAU
MOL000449	Stigmasterol	Leukotriene A-4 hydrolase	LTA4H
MOL000449	Stigmasterol	Amine oxidase [flavin-containing]	MAOB
MOL000449	Stigmasterol	Amine oxidase [flavin-containing]	MAOA
MOL000449	Stigmasterol	cAMP-dependent protein kinase catalytic subunit alpha	PRKACA
MOL000449	Stigmasterol	Chymotrypsinogen B	CTRB1
MOL000449	Stigmasterol	Muscarinic acetylcholine receptor	CHRM3
MOL000449	Stigmasterol	Muscarinic acetylcholine receptor	CHRM1
MOL000449	Stigmasterol	Beta-1 adrenergic receptor	ADRB1
MOL000449	Stigmasterol	Sodium channel protein type 5 subunit alpha	SCN5A
MOL000449	Stigmasterol	Alpha-1A adrenergic receptor	ADRA1A
MOL000449	Stigmasterol	Muscarinic acetylcholine receptor	CHRM2
MOL000449	Stigmasterol	Alpha-1B adrenergic receptor	ADRA1B
MOL000449	Stigmasterol	Neuronal acetylcholine receptor subunit alpha-7	CHRNA7
MOL001510	24-epicampestero	Progesterone receptor	PGR
MOL001510	24-epicampestero	Nuclear receptor coactivator 2	NCOA2
MOL001645	Linoleyl acetate	Prostaglandin G/H synthase 1	PTGS1
MOL001645	Linoleyl acetate	Prostaglandin G/H synthase 2	PTGS2
MOL001645	Linoleyl acetate	Nuclear receptor coactivator 2	NCOA2
MOL001645	Linoleyl acetate	Retinoic acid receptor RXR-alpha	RXRA
MOL001771	iferast-5-en-3beta	Progesterone receptor	PGR
MOL001771	iferast-5-en-3beta	Nuclear receptor coactivator 2	NCOA2
MOL001792	DFV	Prostaglandin G/H synthase 1	PTGS1
MOL001792	DFV	Estrogen receptor	ESR1
MOL001792	DFV	Prostaglandin G/H synthase 2	PTGS2
MOL001792	DFV	Retinoic acid receptor RXR-alpha	RXRA
MOL001792	DFV	Beta-2 adrenergic receptor	ADRB2
MOL001792	DFV	Heat shock protein HSP 90-alpha	HSP90AA1
MOL001792	DFV	cAMP-dependent protein kinase catalytic subunit alpha	PRKACA
MOL001792	DFV	Amine oxidase [flavin-containing]	MAOB
MOL001792	DFV	Sodium-dependent serotonin transporter	SLC6A4
MOL001792	DFV	cAMP-dependent protein kinase inhibitor alpha	PKIA
MOL003044	Chryseriol	Nitric oxide synthase, inducible	NOS2
MOL003044	Chryseriol	Prostaglandin G/H synthase 1	PTGS1
MOL003044	Chryseriol	Estrogen receptor	ESR1
MOL003044	Chryseriol	Androgen receptor	AR
MOL003044	Chryseriol	Peroxisome proliferator-activated receptor gamma	PPARG
MOL003044	Chryseriol	Prostaglandin G/H synthase 2	PTGS2
MOL003044	Chryseriol	Dipeptidyl peptidase 4	DPP4
MOL003044	Chryseriol	Mitogen-activated protein kinase 14	MAPK14
MOL003044	Chryseriol	Glycogen synthase kinase-3 beta	GSK3B
MOL003044	Chryseriol	Heat shock protein HSP 90-alpha	HSP90AA1

MOL003044	Chryseriol	Serine/threonine-protein kinase	CHEK1
MOL003044	Chryseriol	cAMP-dependent protein kinase catalytic subunit alpha	PRKACA
MOL003044	Chryseriol	Trypsin-1	PRSS1
MOL003044	Chryseriol	Nuclear receptor coactivator 2	NCOA2
MOL003044	Chryseriol	Nuclear receptor coactivator 1	NCOA1
MOL003542	opentenyl-kaemp	Prothrombin	F2
MOL003542	opentenyl-kaemp	Estrogen receptor	ESR1
MOL003542	opentenyl-kaemp	Androgen receptor	AR
MOL003542	opentenyl-kaemp	Peroxisome proliferator-activated receptor gamma	PPARG
MOL003542	opentenyl-kaemp	Coagulation factor X	F10
MOL003542	opentenyl-kaemp	Prostaglandin G/H synthase 2	PTGS2
MOL003542	opentenyl-kaemp	DNA topoisomerase 2-alpha	TOP2A
MOL003542	opentenyl-kaemp	Dipeptidyl peptidase 4	DPP4
MOL003542	opentenyl-kaemp	Glycogen synthase kinase-3 beta	GSK3B
MOL003542	opentenyl-kaemp	Heat shock protein HSP 90-alpha	HSP90AA1
MOL003542	opentenyl-kaemp	Serine/threonine-protein kinase	CHEK1
MOL003542	opentenyl-kaemp	Trypsin-1	PRSS1
MOL003542	opentenyl-kaemp	Prostaglandin G/H synthase 1	PTGS1
MOL003542	opentenyl-kaemp	Sodium channel protein type 5 subunit alpha	SCN5A
MOL003542	opentenyl-kaemp	Coagulation factor VII	F7
MOL003542	opentenyl-kaemp	Vascular endothelial growth factor receptor 2	KDR
MOL003542	opentenyl-kaemp	Retinoic acid receptor RXR-alpha	RXRA
MOL003542	opentenyl-kaemp	Mitogen-activated protein kinase 14	MAPK14
MOL003542	opentenyl-kaemp	Cyclin-A2	CCNA2
MOL003542	opentenyl-kaemp	Nuclear receptor coactivator 2	NCOA2
MOL003542	opentenyl-kaemp	cGMP-inhibited 3',5'-cyclic phosphodiesterase A	PDE3A
MOL003542	opentenyl-kaemp	Potassium voltage-gated channel subfamily H member 2	KCNH2
MOL003542	opentenyl-kaemp	Glycogen phosphorylase, muscle	PYGM
MOL000359	sitosterol	Progesterone receptor	PGR
MOL000359	sitosterol	Nuclear receptor coactivator 2	NCOA2
MOL000359	sitosterol	Mineralocorticoid receptor	NR3C2
MOL000422	kaempferol	Nitric oxide synthase, inducible	NOS2
MOL000422	kaempferol	Prostaglandin G/H synthase 1	PTGS1
MOL000422	kaempferol	Androgen receptor	AR
MOL000422	kaempferol	Peroxisome proliferator-activated receptor gamma	PPARG
MOL000422	kaempferol	Prostaglandin G/H synthase 2	PTGS2
MOL000422	kaempferol	Heat shock protein HSP 90-alpha	HSP90AA1
MOL000422	kaempferol	cAMP-dependent protein kinase catalytic subunit alpha	PRKACA
MOL000422	kaempferol	Nuclear receptor coactivator 2	NCOA2
MOL000422	kaempferol	Dipeptidyl peptidase 4	DPP4
MOL000422	kaempferol	Trypsin-1	PRSS1
MOL000422	kaempferol	Progesterone receptor	PGR
MOL000422	kaempferol	Prothrombin	F2
MOL000422	kaempferol	Muscarinic acetylcholine receptor	CHRM1
MOL000422	kaempferol	Acetylcholinesterase	ACHE
MOL000422	kaempferol	Sodium-dependent noradrenaline transporter	SLC6A2
MOL000422	kaempferol	Muscarinic acetylcholine receptor	CHRM2
MOL000422	kaempferol	Alpha-1B adrenergic receptor	ADRA1B
MOL000422	kaempferol	DNA topoisomerase 2-alpha	TOP2A
MOL000422	kaempferol	Coagulation factor VII	F7
MOL000422	kaempferol	Transcription factor p65	RELA

MOL000422	kaempferol	Inhibitor of nuclear factor kappa-B kinase subunit beta	IKBKB
MOL000422	kaempferol	RAC-alpha serine/threonine-protein kinase	AKT1
MOL000422	kaempferol	Apoptosis regulator Bcl-2	BCL2
MOL000422	kaempferol	Apoptosis regulator BAX	BAX
MOL000422	kaempferol	Tumor necrosis factor	TNFSF15
MOL000422	kaempferol	Transcription factor AP-1	JUN
MOL000422	kaempferol	Activator of 90 kDa heat shock protein ATPase homolog 1	AHSA1
MOL000422	kaempferol	Caspase-3	CASP3
MOL000422	kaempferol	Mitogen-activated protein kinase 8	MAPK8
MOL000422	kaempferol	Interstitial collagenase	MMP1
MOL000422	kaempferol	Signal transducer and activator of transcription 1-alpha/beta	STAT1
MOL000422	kaempferol	Peroxisome proliferator-activated receptor gamma	PPARG
MOL000422	kaempferol	Heme oxygenase 1	HMOX1
MOL000422	kaempferol	Cytochrome P450 3A4	CYP3A4
MOL000422	kaempferol	Cytochrome P450 1A1	CYP1A1
MOL000422	kaempferol	Intercellular adhesion molecule 1	ICAM1
MOL000422	kaempferol	E-selectin	SELE
MOL000422	kaempferol	Vascular cell adhesion protein 1	VCAM1
MOL000422	kaempferol	Nuclear receptor subfamily 1 group I member 2	NR1I2
MOL000422	kaempferol	Cytochrome P450 1B1	CYP1B1
MOL000422	kaempferol	Arachidonate 5-lipoxygenase	ALOX5
MOL000422	kaempferol	Hyaluronan synthase 2	HAS2
MOL000422	kaempferol	Aryl hydrocarbon receptor	AHR
MOL000422	kaempferol	26S proteasome non-ATPase regulatory subunit 3	PSMD3
MOL000422	kaempferol	Solute carrier family 2, facilitated glucose transporter member 4	SLC2A4
MOL000422	kaempferol	Nuclear receptor subfamily 1 group I member 3	NR1I3
MOL000422	kaempferol	Insulin receptor	INSR
MOL000422	kaempferol	Type I iodothyronine deiodinase	DIO1
MOL000422	kaempferol	Serine/threonine-protein phosphatase 2B catalytic subunit	PPP3CA
MOL000422	kaempferol	Glutathione S-transferase Mu 1	GSTM1
MOL000422	kaempferol	Glutathione S-transferase Mu 2	GSTM2
MOL000422	kaempferol	Aldo-keto reductase family 1 member C3	AKR1C3
MOL000422	kaempferol	Antileukoproteinase	SLPI
MOL004367	olivil	Prostaglandin G/H synthase 2	PTGS2
MOL004367	olivil	Heat shock protein HSP 90-alpha	HSP90AA1
MOL004367	olivil	Nuclear receptor coactivator 2	NCOA2
MOL004373	Anhydroicaritin	Nitric oxide synthase, inducible	NOS2
MOL004373	Anhydroicaritin	Prostaglandin G/H synthase 1	PTGS1
MOL004373	Anhydroicaritin	Muscarinic acetylcholine receptor	CHRM3
MOL004373	Anhydroicaritin	Prothrombin	F2
MOL004373	Anhydroicaritin	Potassium voltage-gated channel subfamily H member 2	KCNH2
MOL004373	Anhydroicaritin	Muscarinic acetylcholine receptor	CHRM1
MOL004373	Anhydroicaritin	Estrogen receptor	ESR1
MOL004373	Anhydroicaritin	Androgen receptor	AR
MOL004373	Anhydroicaritin	Sodium channel protein type 5 subunit alpha	SCN5A
MOL004373	Anhydroicaritin	Peroxisome proliferator-activated receptor gamma	PPARG

MOL004373	Anhydroicaritin	Coagulation factor X	F10
MOL004373	Anhydroicaritin	Muscarinic acetylcholine receptor	CHRM5
MOL004373	Anhydroicaritin	Prostaglandin G/H synthase 2	PTGS2
MOL004373	Anhydroicaritin	Retinoic acid receptor RXR-alpha	RXRA
MOL004373	Anhydroicaritin	Acetylcholinesterase	ACHE
MOL004373	Anhydroicaritin	Alpha-1B adrenergic receptor	ADRA1B
MOL004373	Anhydroicaritin	Beta-2 adrenergic receptor	ADRB2
MOL004373	Anhydroicaritin	Estrogen receptor beta	ESR2
MOL004373	Anhydroicaritin	Dipeptidyl peptidase 4	DPP4
MOL004373	Anhydroicaritin	Mitogen-activated protein kinase 14	MAPK14
MOL004373	Anhydroicaritin	Glycogen synthase kinase-3 beta	GSK3B
MOL004373	Anhydroicaritin	Heat shock protein HSP 90-alpha	HSP90AA1
MOL004373	Anhydroicaritin	Serine/threonine-protein kinase	CHEK1
MOL004373	Anhydroicaritin	Trypsin-1	PRSS1
MOL004373	Anhydroicaritin	Nuclear receptor coactivator 2	NCOA2
MOL004373	Anhydroicaritin	Coagulation factor VII	F7
MOL004373	Anhydroicaritin	Vascular endothelial growth factor receptor 2	KDR
MOL004373	Anhydroicaritin	DNA topoisomerase 2-alpha	TOP2A
MOL004373	Anhydroicaritin	Cyclin-A2	CCNA2
MOL004373	Anhydroicaritin	Nuclear receptor coactivator 1	NCOA1
MOL004380	didehydro-3,15,1	Prostaglandin G/H synthase 1	PTGS1
MOL004380	didehydro-3,15,1	D(1A) dopamine receptor	DRD1
MOL004380	didehydro-3,15,1	Muscarinic acetylcholine receptor	CHRM3
MOL004380	didehydro-3,15,1	Potassium voltage-gated channel subfamily H member 2	KCNH2
MOL004380	didehydro-3,15,1	Muscarinic acetylcholine receptor	CHRM1
MOL004380	didehydro-3,15,1	Androgen receptor	AR
MOL004380	didehydro-3,15,1	D(1B) dopamine receptor	DRD5
MOL004380	didehydro-3,15,1	Beta-1 adrenergic receptor	ADRB1
MOL004380	didehydro-3,15,1	Sodium channel protein type 5 subunit alpha	SCN5A
MOL004380	didehydro-3,15,1	Muscarinic acetylcholine receptor	CHRM5
MOL004380	didehydro-3,15,1	Prostaglandin G/H synthase 2	PTGS2
MOL004380	didehydro-3,15,1	Alpha-2A adrenergic receptor	ADRA2A
MOL004380	didehydro-3,15,1	Muscarinic acetylcholine receptor	CHRM4
MOL004380	didehydro-3,15,1	Delta-type opioid receptor	OPRD1
MOL004380	didehydro-3,15,1	cGMP-inhibited 3',5'-cyclic phosphodiesterase A	PDE3A
MOL004380	didehydro-3,15,1	Histamine H1 receptor	HRH1
MOL004380	didehydro-3,15,1	Sodium-dependent noradrenaline transporter	SLC6A2
MOL004380	didehydro-3,15,1	Alpha-1A adrenergic receptor	ADRA1A
MOL004380	didehydro-3,15,1	Muscarinic acetylcholine receptor	CHRM2
MOL004380	didehydro-3,15,1	Alpha-2B adrenergic receptor	ADRA2B
MOL004380	didehydro-3,15,1	Alpha-1B adrenergic receptor	ADRA1B
MOL004380	didehydro-3,15,1	Sodium-dependent dopamine transporter	SLC6A3
MOL004380	didehydro-3,15,1	Beta-2 adrenergic receptor	ADRB2
MOL004380	didehydro-3,15,1	Alpha-1D adrenergic receptor	ADRA1D
MOL004380	didehydro-3,15,1	Neuronal acetylcholine receptor subunit alpha-2	CHRNA2
MOL004380	didehydro-3,15,1	Sodium-dependent serotonin transporter	SLC6A4
MOL004380	didehydro-3,15,1	D(2) dopamine receptor	DRD2
MOL004380	didehydro-3,15,1	Mu-type opioid receptor	OPRM1
MOL004380	didehydro-3,15,1	Neuronal acetylcholine receptor subunit alpha-7	CHRNA7
MOL004380	didehydro-3,15,1	Gamma-aminobutyric acid receptor subunit gamma-3	GABRG3

MOL004380	didehydro-3,15,1	Gamma-aminobutyric acid receptor subunit epsilon	GABRE
MOL004382	Yinyanghuo A	Estrogen receptor	ESR1
MOL004382	Yinyanghuo A	Androgen receptor	AR
MOL004382	Yinyanghuo A	Coagulation factor X	F10
MOL004382	Yinyanghuo A	Prostaglandin G/H synthase 2	PTGS2
MOL004382	Yinyanghuo A	DNA topoisomerase 2-alpha	TOP2A
MOL004382	Yinyanghuo A	Trypsin-1	PRSS1
MOL004382	Yinyanghuo A	Nuclear receptor coactivator 2	NCOA2
MOL004384	Yinyanghuo C	Nitric oxide synthase, inducible	NOS2
MOL004384	Yinyanghuo C	Androgen receptor	AR
MOL004384	Yinyanghuo C	Coagulation factor X	F10
MOL004384	Yinyanghuo C	Prostaglandin G/H synthase 2	PTGS2
MOL004384	Yinyanghuo C	Acetylcholinesterase	ACHE
MOL004384	Yinyanghuo C	Estrogen receptor beta	ESR2
MOL004384	Yinyanghuo C	Dipeptidyl peptidase 4	DPP4
MOL004384	Yinyanghuo C	Trypsin-1	PRSS1
MOL004384	Yinyanghuo C	Nuclear receptor coactivator 2	NCOA2
MOL004386	Yinyanghuo E	Nitric oxide synthase, inducible	NOS2
MOL004386	Yinyanghuo E	Androgen receptor	AR
MOL004386	Yinyanghuo E	Coagulation factor X	F10
MOL004386	Yinyanghuo E	Prostaglandin G/H synthase 2	PTGS2
MOL004386	Yinyanghuo E	Acetylcholinesterase	ACHE
MOL004386	Yinyanghuo E	DNA topoisomerase 2-alpha	TOP2A
MOL004386	Yinyanghuo E	Dipeptidyl peptidase 4	DPP4
MOL004386	Yinyanghuo E	cAMP-dependent protein kinase catalytic subunit alpha	PRKACA
MOL004386	Yinyanghuo E	Trypsin-1	PRSS1
MOL004386	Yinyanghuo E	Nuclear receptor coactivator 2	NCOA2
MOL004388	ioxo-2,3,4,8-tetra	Prostaglandin G/H synthase 1	PTGS1
MOL004388	ioxo-2,3,4,8-tetra	Coagulation factor X	F10
MOL004388	ioxo-2,3,4,8-tetra	Prostaglandin G/H synthase 2	PTGS2
MOL004388	ioxo-2,3,4,8-tetra	DNA topoisomerase 2-alpha	TOP2A
MOL004388	ioxo-2,3,4,8-tetra	Heat shock protein HSP 90-alpha	HSP90AA1
MOL004391	ut-2-enyl)-2-pher	Nitric oxide synthase, inducible	NOS2
MOL004391	ut-2-enyl)-2-pher	Prostaglandin G/H synthase 1	PTGS1
MOL004391	ut-2-enyl)-2-pher	Muscarinic acetylcholine receptor	CHRM3
MOL004391	ut-2-enyl)-2-pher	Prothrombin	F2
MOL004391	ut-2-enyl)-2-pher	Muscarinic acetylcholine receptor	CHRM1
MOL004391	ut-2-enyl)-2-pher	Estrogen receptor	ESR1
MOL004391	ut-2-enyl)-2-pher	Androgen receptor	AR
MOL004391	ut-2-enyl)-2-pher	Beta-1 adrenergic receptor	ADRB1
MOL004391	ut-2-enyl)-2-pher	Sodium channel protein type 5 subunit alpha	SCN5A
MOL004391	ut-2-enyl)-2-pher	Peroxisome proliferator-activated receptor gamma	PPARG
MOL004391	ut-2-enyl)-2-pher	Prostaglandin G/H synthase 2	PTGS2
MOL004391	ut-2-enyl)-2-pher	Retinoic acid receptor RXR-alpha	RXRA
MOL004391	ut-2-enyl)-2-pher	Acetylcholinesterase	ACHE
MOL004391	ut-2-enyl)-2-pher	cGMP-inhibited 3',5'-cyclic phosphodiesterase A	PDE3A
MOL004391	ut-2-enyl)-2-pher	Alpha-1B adrenergic receptor	ADRA1B
MOL004391	ut-2-enyl)-2-pher	Sodium-dependent dopamine transporter	SLC6A3
MOL004391	ut-2-enyl)-2-pher	Beta-2 adrenergic receptor	ADRB2
MOL004391	ut-2-enyl)-2-pher	Alpha-1D adrenergic receptor	ADRA1D
MOL004391	ut-2-enyl)-2-pher	Sodium-dependent serotonin transporter	SLC6A4
MOL004391	ut-2-enyl)-2-pher	Estrogen receptor beta	ESR2

MOL004391	ut-2-enyl)-2-pher	Dipeptidyl peptidase 4	DPP4
MOL004391	ut-2-enyl)-2-pher	Mitogen-activated protein kinase 14	MAPK14
MOL004391	ut-2-enyl)-2-pher	Glycogen synthase kinase-3 beta	GSK3B
MOL004391	ut-2-enyl)-2-pher	Heat shock protein HSP 90-alpha	HSP90AA1
MOL004391	ut-2-enyl)-2-pher	Serine/threonine-protein kinase	CHEK1
MOL004391	ut-2-enyl)-2-pher	Cyclin-A2	CCNA2
MOL004394	:itin-3-O-alpha-L	DNA topoisomerase 2-alpha	TOP2A
MOL004396	'-3-methoxypheny	Estrogen receptor	ESR1
MOL004396	'-3-methoxypheny	Sodium channel protein type 5 subunit alpha	SCN5A
MOL004396	'-3-methoxypheny	Prostaglandin G/H synthase 2	PTGS2
MOL004396	'-3-methoxypheny	Alpha-1B adrenergic receptor	ADRA1B
MOL004396	'-3-methoxypheny	Beta-2 adrenergic receptor	ADRB2
MOL004396	'-3-methoxypheny	Heat shock protein HSP 90-alpha	HSP90AA1
MOL004396	'-3-methoxypheny	Cyclin-A2	CCNA2
MOL004396	'-3-methoxypheny	Nuclear receptor coactivator 2	NCOA2
MOL004425	Icariin	DNA topoisomerase 2-alpha	TOP2A
MOL004427	Icariside A7	Prostaglandin G/H synthase 2	PTGS2
MOL004427	Icariside A7	DNA topoisomerase 2-alpha	TOP2A
MOL004427	Icariside A7	Nuclear receptor coactivator 2	NCOA2
MOL000006	luteolin	Prostaglandin G/H synthase 1	PTGS1
MOL000006	luteolin	Androgen receptor	AR
MOL000006	luteolin	Prostaglandin G/H synthase 2	PTGS2
MOL000006	luteolin	Heat shock protein HSP 90-alpha	HSP90AA1
MOL000006	luteolin	Trypsin-1	PRSS1
MOL000006	luteolin	Nuclear receptor coactivator 2	NCOA2
MOL000006	luteolin	cAMP-dependent protein kinase catalytic subunit alpha	PRKACA
MOL000006	luteolin	Dipeptidyl peptidase 4	DPP4
MOL000006	luteolin	Transcription factor p65	RELA
MOL000006	luteolin	Epidermal growth factor receptor	EGFR
MOL000006	luteolin	RAC-alpha serine/threonine-protein kinase	AKT1
MOL000006	luteolin	G1/S-specific cyclin-D1	CCND1
MOL000006	luteolin	Bcl-2-like protein 1	BCL2L1
MOL000006	luteolin	Cyclin-dependent kinase inhibitor 1	CDKN1A
MOL000006	luteolin	Caspase-9	CASP9
MOL000006	luteolin	72 kDa type IV collagenase	MMP2
MOL000006	luteolin	Matrix metalloproteinase-9	MMP9
MOL000006	luteolin	Mitogen-activated protein kinase 1	MAPK1
MOL000006	luteolin	Interleukin-10	IL10
MOL000006	luteolin	Retinoblastoma-associated protein	RB1
MOL000006	luteolin	Tumor necrosis factor	TNFSF15
MOL000006	luteolin	Transcription factor AP-1	JUN
MOL000006	luteolin	Interleukin-6	IL6
MOL000006	luteolin	Caspase-3	CASP3
MOL000006	luteolin	Cellular tumor antigen p53	TP63
MOL000006	luteolin	NF-kappa-B inhibitor alpha	NFKBIA
MOL000006	luteolin	DNA topoisomerase 1	TOP1
MOL000006	luteolin	E3 ubiquitin-protein ligase Mdm2	MDM2
MOL000006	luteolin	Amyloid beta A4 protein	APP
MOL000006	luteolin	Interstitial collagenase	MMP1
MOL000006	luteolin	Proliferating cell nuclear antigen	PCNA
MOL000006	luteolin	Receptor tyrosine-protein kinase erbB-2	ERBB2
MOL000006	luteolin	Peroxisome proliferator-activated receptor gamma	PPARG
MOL000006	luteolin	Heme oxygenase 1	HMOX1
MOL000006	luteolin	Caspase-7	CASP7

MOL000006	luteolin	Intercellular adhesion molecule 1	ICAM1
MOL000006	luteolin	Induced myeloid leukemia cell differentiation protein Mcl-1	MCL1
MOL000006	luteolin	Baculoviral IAP repeat-containing protein 5	BIRC5
MOL000006	luteolin	Interleukin-2	IL2
MOL000006	luteolin	G2/mitotic-specific cyclin-B1	CCNB1
MOL000006	luteolin	Tyrosinase	TYR
MOL000006	luteolin	Interferon gamma	IFNG
MOL000006	luteolin	Interleukin-4	IL4
MOL000006	luteolin	DNA topoisomerase 2-alpha	TOP2A
MOL000006	luteolin	Solute carrier family 2, facilitated glucose transporter member 4	SLC2A4
MOL000006	luteolin	Insulin receptor	INSR
MOL000006	luteolin	CD40 ligand	CD40LG
MOL000006	luteolin	Prostaglandin E synthase	PTGES
MOL000006	luteolin	Kinetochore protein Nuf2	NUF2
MOL000006	luteolin	Adenylate cyclase type 2	ADCY2
MOL000006	luteolin	Hepatocyte growth factor receptor	MET
MOL000622	Magnograndiolid	Glutamate receptor 2	GRIA2
MOL000098	quercetin	Prostaglandin G/H synthase 1	PTGS1
MOL000098	quercetin	Androgen receptor	AR
MOL000098	quercetin	Peroxisome proliferator-activated receptor gamma	PPARG
MOL000098	quercetin	Prostaglandin G/H synthase 2	PTGS2
MOL000098	quercetin	Heat shock protein HSP 90-alpha	HSP90AA1
MOL000098	quercetin	Nuclear receptor coactivator 2	NCOA2
MOL000098	quercetin	Dipeptidyl peptidase 4	DPP4
MOL000098	quercetin	Aldose reductase	AKR1B1
MOL000098	quercetin	Trypsin-1	PRSS1
MOL000098	quercetin	DNA topoisomerase 2-alpha	TOP2A
MOL000098	quercetin	Prothrombin	F2
MOL000098	quercetin	Potassium voltage-gated channel subfamily H member 2	KCNH2
MOL000098	quercetin	Sodium channel protein type 5 subunit alpha	SCN5A
MOL000098	quercetin	Coagulation factor X	F10
MOL000098	quercetin	Beta-2 adrenergic receptor	ADRB2
MOL000098	quercetin	Stromelysin-1	MMP3
MOL000098	quercetin	cAMP-dependent protein kinase catalytic subunit alpha	PRKACA
MOL000098	quercetin	Coagulation factor VII	F7
MOL000098	quercetin	Retinoic acid receptor RXR-alpha	RXRA
MOL000098	quercetin	Acetylcholinesterase	ACHE
MOL000098	quercetin	Amine oxidase [flavin-containing]	MAOB
MOL000098	quercetin	Transcription factor p65	RELA
MOL000098	quercetin	Epidermal growth factor receptor	EGFR
MOL000098	quercetin	RAC-alpha serine/threonine-protein kinase	AKT1
MOL000098	quercetin	G1/S-specific cyclin-D1	CCND1
MOL000098	quercetin	Apoptosis regulator Bcl-2	BCL2
MOL000098	quercetin	Bcl-2-like protein 1	BCL2L1
MOL000098	quercetin	Proto-oncogene c-Fos	FOS
MOL000098	quercetin	Cyclin-dependent kinase inhibitor 1	CDKN1A
MOL000098	quercetin	Eukaryotic translation initiation factor 6	EIF6
MOL000098	quercetin	Apoptosis regulator BAX	BAX
MOL000098	quercetin	Caspase-9	CASP9
MOL000098	quercetin	Urokinase-type plasminogen	PLAU
MOL000098	quercetin	72 kDa type IV collagenase	MMP2

MOL000098	quercetin	Matrix metalloproteinase-9	MMP9
MOL000098	quercetin	Mitogen-activated protein kinase 1	MAPK1
MOL000098	quercetin	Interleukin-10	IL10
MOL000098	quercetin	Retinoblastoma-associated protein	RB1
MOL000098	quercetin	Tumor necrosis factor	TNFSF15
MOL000098	quercetin	Transcription factor AP-1	JUN
MOL000098	quercetin	Interleukin-6	IL6
MOL000098	quercetin	Activator of 90 kDa heat shock protein ATPase homolog 1	AHSA1
MOL000098	quercetin	Caspase-3	CASP3
MOL000098	quercetin	Cellular tumor antigen p53	TP63
MOL000098	quercetin	ETS domain-containing protein Elk-	ELK1
MOL000098	quercetin	NF-kappa-B inhibitor alpha	NFKBIA
MOL000098	quercetin	Ornithine decarboxylase	ODC1
MOL000098	quercetin	Caspase-8	CASP8
MOL000098	quercetin	DNA topoisomerase 1	TOP1
MOL000098	quercetin	RAF proto-oncogene serine/threonine-protein kinase	RAF1
MOL000098	quercetin	Superoxide dismutase [Cu-Zn]	SOD1
MOL000098	quercetin	Protein kinase C alpha type	PRKCA
MOL000098	quercetin	Interstitial collagenase	MMP1
MOL000098	quercetin	Hypoxia-inducible factor 1-alpha	HIF1A
MOL000098	quercetin	Signal transducer and activator of transcription 1-alpha/beta	STAT1
MOL000098	quercetin	Protein CBFA2T1	RUNX1T1
MOL000098	quercetin	Receptor tyrosine-protein kinase erbB-2	ERBB2
MOL000098	quercetin	Peroxisome proliferator-activated receptor gamma	PPARG
MOL000098	quercetin	Acetyl-CoA carboxylase 1	ACACA
MOL000098	quercetin	Heme oxygenase 1	HMOX1
MOL000098	quercetin	Cytochrome P450 3A4	CYP3A4
MOL000098	quercetin	Caveolin-1	CAV1
MOL000098	quercetin	Myc proto-oncogene protein	MYC
MOL000098	quercetin	Tissue factor	F3
MOL000098	quercetin	Gap junction alpha-1 protein	GJA1
MOL000098	quercetin	Cytochrome P450 1A1	CYP1A1
MOL000098	quercetin	Intercellular adhesion molecule 1	ICAM1
MOL000098	quercetin	Interleukin-1 beta	IL1B
MOL000098	quercetin	E-selectin	SELE
MOL000098	quercetin	Vascular cell adhesion protein 1	VCAM1
MOL000098	quercetin	Interleukin-8	CXCL8
MOL000098	quercetin	Protein kinase C beta type	PRKCB
MOL000098	quercetin	Baculoviral IAP repeat-containing protein 5	BIRC5
MOL000098	quercetin	Dual oxidase 2	DUOX2
MOL000098	quercetin	Nitric oxide synthase, endothelial	NOS3
MOL000098	quercetin	Heat shock protein beta-1	HSPB1
MOL000098	quercetin	Maltase-glucoamylase, intestinal	MGAM
MOL000098	quercetin	Interleukin-2	IL2
MOL000098	quercetin	Nuclear receptor subfamily 1 group I member 2	NR1I2
MOL000098	quercetin	Cytochrome P450 1B1	CYP1B1
MOL000098	quercetin	G2/mitotic-specific cyclin-B1	CCNB1
MOL000098	quercetin	Tissue-type plasminogen activator	PLAT
MOL000098	quercetin	Thrombomodulin	THBD
MOL000098	quercetin	Plasminogen activator inhibitor 1	SERPINE1
MOL000098	quercetin	Interferon gamma	IFNG
MOL000098	quercetin	Arachidonate 5-lipoxygenase	ALOX5

MOL000098	quercetin	Interleukin-1 alpha	IL1A
MOL000098	quercetin	Myeloperoxidase	MPO
MOL000098	quercetin	DNA topoisomerase 2-alpha	TOP2A
MOL000098	quercetin	Neutrophil cytosol factor 1	NCF1
MOL000098	quercetin	ATP-binding cassette sub-family G member 2	ABCG2
MOL000098	quercetin	Hyaluronan synthase 2	HAS2
MOL000098	quercetin	Nuclear factor erythroid 2-related factor 2	NFE2L2
MOL000098	quercetin	NAD(P)H dehydrogenase [quinone]	NQO1
MOL000098	quercetin	Poly [ADP-ribose] polymerase 1	PARP1
MOL000098	quercetin	Aryl hydrocarbon receptor	AHR
MOL000098	quercetin	26S proteasome non-ATPase regulatory subunit 3	PSMD3
MOL000098	quercetin	Solute carrier family 2, facilitated glucose transporter member 4	SLC2A4
MOL000098	quercetin	Collagen alpha-1(III) chain	COL3A1
MOL000098	quercetin	C-X-C motif chemokine 11	CXCL11
MOL000098	quercetin	C-X-C motif chemokine 2	CXCL2
MOL000098	quercetin	DDB1- and CUL4-associated factor	DCAF5
MOL000098	quercetin	Nuclear receptor subfamily 1 group I member 3	NR1I3
MOL000098	quercetin	Serine/threonine-protein kinase	CHEK2
MOL000098	quercetin	Insulin receptor	INSR
MOL000098	quercetin	Claudin-4	CLDN4
MOL000098	quercetin	Peroxisome proliferator-activated receptor alpha	PPARA
MOL000098	quercetin	Peroxisome proliferator-activated receptor delta	PPARD
MOL000098	quercetin	Heat shock factor protein 1	HSF1
MOL000098	quercetin	C-reactive protein	CRP
MOL000098	quercetin	C-X-C motif chemokine 10	CXCL10
MOL000098	quercetin	Inhibitor of nuclear factor kappa-B kinase subunit alpha	CHUK
MOL000098	quercetin	Osteopontin	SPP1
MOL000098	quercetin	Runt-related transcription factor 2	RUNX2
MOL000098	quercetin	Ras association domain-containing protein 1	RASSF1
MOL000098	quercetin	Transcription factor E2F1	E2F1
MOL000098	quercetin	Transcription factor E2F2	E2F2
MOL000098	quercetin	Prostatic acid phosphatase	ACPP
MOL000098	quercetin	Cathepsin D	CTSD
MOL000098	quercetin	Insulin-like growth factor-binding protein 3	IGFBP3
MOL000098	quercetin	Insulin-like growth factor II	IGF2
MOL000098	quercetin	CD40 ligand	CD40LG
MOL000098	quercetin	Interferon regulatory factor 1	IRF1
MOL000098	quercetin	Receptor tyrosine-protein kinase erbB-3	ERBB3
MOL000098	quercetin	Serum paraoxonase/arylesterase 1	PON1
MOL000098	quercetin	Type I iodothyronine deiodinase	DIO1
MOL000098	quercetin	Procollagen C-endopeptidase enhancer 1	PCOLCE
MOL000098	quercetin	Puromycin-sensitive	NPEPPS
MOL000098	quercetin	Hexokinase-2	HK2
MOL000098	quercetin	Ras GTPase-activating protein 1	RASA1
MOL000098	quercetin	Glutathione S-transferase Mu 1	GSTM1
MOL000098	quercetin	Glutathione S-transferase Mu 2	GSTM2
MOL001525	Daucosterol	Progesterone receptor	PGR
MOL001525	Daucosterol	Nuclear receptor coactivator 2	NCOA2

MOL002268	Rhein	Prostaglandin G/H synthase 1	PTGS1
MOL002268	Rhein	Prostaglandin G/H synthase 2	PTGS2
MOL002268	Rhein	Heat shock protein HSP 90-alpha	HSP90AA1
MOL002268	Rhein	Phosphatidylinositol 4,5-bisphosphate 3-kinase catalytic subunit gamma isoform	PIK3CG
MOL002268	Rhein	Nuclear receptor coactivator 6	NCOA6
MOL002268	Rhein	Aldo-keto reductase family 1 member B10	AKR1B10
MOL002268	Rhein	Transcription factor AP-1	JUN
MOL002268	Rhein	Folylpolyglutamate synthase	FPGS
MOL002268	Rhein	Thymidylate synthase	TYMS
MOL002268	Rhein	Interleukin-13	IL13
MOL002268	Rhein	Peroxisome proliferator-activated receptor gamma	PPARG
MOL002268	Rhein	Thrombopoietin receptor	MPL
MOL002268	Rhein	Interleukin-1 beta	IL1B
MOL002268	Rhein	Prostaglandin G/H synthase 1	PTGS1
MOL002268	Rhein	Apolipoprotein A-II	APOA2
MOL002268	Rhein	Gamma-glutamyl hydrolase	GGH
MOL002268	Rhein	Prostaglandin G/H synthase 2	PTGS2
MOL002268	Rhein	Serine hydroxymethyltransferase	SHMT1
MOL002268	Rhein	Serpin B7	SERPINB7
MOL002268	Rhein	Arachidonate 5-lipoxygenase	ALOX5
MOL002268	Rhein	Plasminogen activator inhibitor 1	SERPINE1
MOL008647	rans-Feruloyltyra	Melatonin receptor type 1A	MTNR1A
MOL008647	rans-Feruloyltyra	Melatonin receptor type 1B	MTNR1B
MOL008647	rans-Feruloyltyra	5-hydroxytryptamine receptor 2C	HTR2C

Supplementary Table 3

Related Target Genes for AD Obtained from Public Databases

Public Database	Target Genes
TTD	FGFR1
TTD	AChE
TTD	FGFR2
TTD	FLT-3
TTD	INSR
TTD	MAO-A
TTD	TrkA
TTD	KIT
TTD	KDR
TTD	AGTR1
TTD	ESR2
TTD	HPGDS
TTD	HSP90A
TTD	H1R
TTD	MC4R
TTD	MTNR1A
TTD	MAO-B
TTD	CHRM3
TTD	TrkC
TTD	OPRS1
TTD	PPAR-gamma
TTD	PLG
TTD	PDGFRB
TTD	COX-1
TTD	PRKCE
TTD	MET
TTD	VEGFR1 mRNA
TTD	HTR1A
TTD	HTR1B
TTD	HTR2A
TTD	HTR2C
TTD	HTR4
TTD	ADORA2A
TTD	APP
TTD	TrkB
TTD	BCHE
TTD	D2R
TTD	D3R
TTD	D4R
TTD	GABRA5
TTD	NMDAR1
TTD	NMDAR2A
TTD	NMDAR2B
TTD	IL1B
TTD	NGFR
TTD	MAPK12
TTD	CHRM1
TTD	CHRM2
TTD	CHRNA4
TTD	PDE4D
TTD	SERT
TTD	TSPO
TTD	HTR1D
TTD	ADORA2B
TTD	ADRA2C

TTD	GRIA1
TTD	LEPR
TTD	CHRNA7
TTD	PTGFR
TTD	COX-2
TTD	RARB
TTD	SSTR1
TTD	SSTR2
TTD	SSTR3
TTD	SSTR5
TTD	SLC18A2
TTD	CACNA1C
TTD	TOP2
TTD	GABRG3
TTD	CHRM5
TTD	CHRNA4/B2
TTD	FGFR3
TTD	GSK-3B
TTD	HSD11B1
TTD	IKKB
TTD	PLA2G7
TTD	PTGES
TTD	RARA
TTD	p38 alpha
TTD	p38 beta
TTD	FLT-4
TTD	HTR6
TTD	ANGPT1
TTD	ANGPT2
TTD	CCR2
TTD	CAPN2
TTD	CTSL
TTD	F11 mRNA
TTD	FA12 mRNA
TTD	H3R
TTD	RON
TTD	mGluR2
TTD	mGluR3
TTD	mGluR5
TTD	CHRNB2
TTD	CHRNB4
TTD	PPARD
TTD	PDE9
TTD	MERTK
TTD	HTR7
TTD	AGER
TTD	BACE1
TTD	BRD4
TTD	CSF1R
TTD	NGF
TTD	P2RX7
TTD	P2RY6
TTD	SSTR4
TTD	SNCA
TTD	OX40
TTD	TRPV6
TTD	CACNA1D
TTD	GS

TTD	MAPT
TTD	MycB pssA
TTD	RIPK1
TTD	KCNN4
TTD	FLNA
TTD	PAI
TTD	TMEM97
TTD	TARDBP
TTD	CTSB
TTD	APH-1
TTD	mGluR1
TTD	RAC1
TTD	APP mRNA
TTD	FPR2
TTD	FPR3
TTD	nAChR
TTD	GRIA
TTD	KCN
TTD	CaC
TTD	CHRM
TTD	NMDAR
TTD	OPR
TTD	MAO
TTD	DNA synth
TTD	PRO
TTD	SNDR
TTD	BACE
TTD	NCAM
TTD	CRF
TTD	LPO
TTD	MP
TTD	SSTR
TTD	TauA
TTD	GPCR
TTD	MicroTu
OMIM	AD
OMIM	AD2
OMIM	AD17
OMIM	AD9
OMIM	AD19
OMIM	AD18
OMIM	AD15
OMIM	DLB
OMIM	APP
OMIM	SNCA
OMIM	AD13
OMIM	AD14
OMIM	NOS3
OMIM	AD16
OMIM	COL25A1
OMIM	NPC1
OMIM	GSD
OMIM	PD
OMIM	FTD
OMIM	IBMPFD1
OMIM	BPTF
OMIM	HD
OMIM	PARK8

OMIM	PSEN1
OMIM	APOE
OMIM	CJD
OMIM	MAPT
OMIM	FTDALS1
OMIM	NIID
OMIM	PSEN2
OMIM	PRNP
OMIM	UBQLN1
OMIM	SORL1
OMIM	BACE1
OMIM	ABCA7
OMIM	A2M
OMIM	PLD3
OMIM	TREM2
OMIM	NCSTN
OMIM	PLAU
OMIM	ACE
OMIM	PARK1
OMIM	CST3
OMIM	LRP1
OMIM	SERPINA3
OMIM	IDE
OMIM	ADAM10
OMIM	IL1A
OMIM	OLR1
OMIM	TNF
OMIM	VCP
OMIM	HSD17B10
OMIM	APBB2
OMIM	PARK4
OMIM	PARK2
OMIM	MPO
OMIM	PLOSL1
OMIM	CMT2T
OMIM	PMF
OMIM	DBN1
OMIM	APBB1
OMIM	NLRP3
OMIM	CASP1
OMIM	MTRNR2
OMIM	TFCP2
OMIM	APBA1
OMIM	APBA2
OMIM	SORBS1
OMIM	THOP1
OMIM	GRN
OMIM	PSNP1
OMIM	MME
OMIM	PIN1
OMIM	CLU
OMIM	GAPDH
OMIM	TREML2
OMIM	TF
OMIM	FTDALS6
OMIM	HDLS
OMIM	UBB
OMIM	MTND1

OMIM	HFE
OMIM	BLMH
OMIM	CYP2D6
OMIM	NRG1
OMIM	MTCO2
OMIM	BECN1
OMIM	PPIF
OMIM	GSK3B
OMIM	ABCA2
OMIM	DYRK1A
OMIM	BCHE
OMIM	GAL
OMIM	CD33
OMIM	PICALM
OMIM	QPCT
OMIM	GPR3
OMIM	APH1B
OMIM	APBB3
OMIM	NBIA1
OMIM	TGFB1
OMIM	CASP3
OMIM	ATXN2
OMIM	VEGFA
OMIM	BIN1
OMIM	CD40LG
OMIM	DM1
OMIM	KLK6
OMIM	GAB2
OMIM	NOTCH1
OMIM	FAS
OMIM	HSPA9
OMIM	MTCO1
OMIM	APLP1
OMIM	REST
OMIM	SIRT1
OMIM	PRKCD
OMIM	CD40
OMIM	CETP
OMIM	HTR2A
OMIM	INS
OMIM	GLO1
OMIM	REG1A
OMIM	GAPDHS
OMIM	AGER
OMIM	HSD11B1
OMIM	CTSG
OMIM	PYCARD
OMIM	CALHM1
OMIM	MT3
OMIM	CYP46A1
OMIM	DHCR24
OMIM	UTP11L
OMIM	MEOX2
OMIM	GSAP
OMIM	CCR2
OMIM	GBA
OMIM	EPHB2
OMIM	LRRK2

OMIM	CNTF
OMIM	NOTCH2NLC
OMIM	NM
OMIM	CFH
OMIM	PSENEN
OMIM	SCZD
OMIM	MSA1
OMIM	SLC10A4
OMIM	LILRB2
OMIM	BDNF
OMIM	ATXN8OS
OMIM	HLA-A
OMIM	NOS2
OMIM	HMOX1
OMIM	PRND
OMIM	COMT
OMIM	FGA
OMIM	SLC6A4
OMIM	CMD1V
OMIM	ECE1
OMIM	CHRNA7
OMIM	TRPM7
OMIM	VLDLR
OMIM	MIR29A
OMIM	MIR29B1
OMIM	RELN
OMIM	ESR2
OMIM	CHAT
OMIM	CTSB
OMIM	NTRK1
OMIM	DNM1L
OMIM	HDAC2
OMIM	FTDALS8
DrugBank	PKD2L1
DrugBank	PKD1L3
DrugBank	IGHG1
DrugBank	IGHG2
DrugBank	IGHG4
DrugBank	IGHG3
DrugBank	ATP7B
DrugBank	HTT
DrugBank	PARK7
DrugBank	ATP7A
DrugBank	APP
DrugBank	CTSD
DrugBank	MMP19
DrugBank	CYBB
DrugBank	CYBA
DrugBank	NCF1
DrugBank	NCF2
DrugBank	NCF4
DrugBank	RAC1
DrugBank	RAC2
DrugBank	ADAMTS4
DrugBank	HSD17B10
DrugBank	NPEPPS
DrugBank	HIF1A
DrugBank	KLK6

DrugBank	ANPEP
DrugBank	ITGB7
DrugBank	LRRK2
DrugBank	GSK3A
DrugBank	GSK3B
DrugBank	TBK1
DrugBank	CASP4
DrugBank	NR1D1
PHA	CLCN5
PHA	PKD1
PHA	PRNP
PHA	PKD2
PHA	GLRA1
PHA	AR
PHA	TGFB1
PHA	CLCN1
PHA	NDP
PHA	PKHD1
PHA	MRE11
PHA	EPM2A
PHA	ABCA1
PHA	ATP7B
PHA	PLP1
PHA	PHYH
PHA	LIPA
PHA	ABCA4
PHA	NCF1
PHA	NCF2
PHA	BCKDHB
PHA	PYGM
PHA	NF1
PHA	NPC1
PHA	BCKDHA
PHA	HEXA
PHA	AGL
PHA	PKD1P1
PHA	PYGL
PHA	PKD1L1
PHA	NAGLU
PHA	G6PC
PHA	PKDREJ
PHA	PKD2L2
PHA	PKD1L2
PHA	GNS
PHA	ASPA
PHA	F9
PHA	SLC6A19
PHA	GALC
PHA	PKD1L3
PHA	PKD2L1
PHA	PEX7
PHA	ABCG8
PHA	PSAP
PHA	NPC2
PHA	BEST1
PHA	RMD1
PHA	CELIAC2
PHA	PARK10

PHA	PKD3
PHA	KWE
PHA	MYP1
PHA	PARK3
PHA	CCAL1
PHA	PKD1P6
PHA	PKD1P5
PHA	PKD1P4
PHA	PKD1P3
PHA	PKD1P2
PHA	SLC2A4RG
PHA	JCAD
PHA	SLC25A16
PHA	GATD1
PHA	GBE1
PHA	CCDC180
PHA	PKHD1L1
PHA	AD5
PHA	PSEN2
PHA	CTD
PHA	SH2D1A
PHA	OED
PHA	MYMY1
PHA	MYMY3
PHA	GAA
PHA	PDB4
PHA	PDB1
PHA	PDB5
PHA	PDB6
PHA	IBD3
PHA	IBD5
PHA	IBD2
PHA	IBD4
PHA	IBD6
PHA	IBD7
PHA	IBD9
PHA	IBD8
GeneCard	PSEN1
GeneCard	APP
GeneCard	APOE
GeneCard	PSEN2
GeneCard	MAPT
GeneCard	SNCA
GeneCard	GBA
GeneCard	HFE
GeneCard	NOS3
GeneCard	PKD1
GeneCard	MT-ND1
GeneCard	MPO
GeneCard	NPC1
GeneCard	PRNP
GeneCard	LRRK2
GeneCard	IL6
GeneCard	TNF
GeneCard	IL10
GeneCard	GAA
GeneCard	LMNA
GeneCard	PRKN

GeneCard	A2M
GeneCard	AD5
GeneCard	PLAU
GeneCard	MIR146A
GeneCard	ABCA7
GeneCard	SQSTM1
GeneCard	NOD2
GeneCard	MPZ
GeneCard	LOC106627981
GeneCard	VCP
GeneCard	AD6
GeneCard	SMPD1
GeneCard	AD10
GeneCard	TP53
GeneCard	MIR34A
GeneCard	TGFB1
GeneCard	AD7
GeneCard	MFN2
GeneCard	NEFL
GeneCard	ACE
GeneCard	AD11
GeneCard	AD8
GeneCard	MIR29A
GeneCard	AD17
GeneCard	AD12
GeneCard	AD13
GeneCard	AD14
GeneCard	AD16
GeneCard	HFE-AS1
GeneCard	ATP7B
GeneCard	MIR29B1
GeneCard	MIR106B
GeneCard	VWF
GeneCard	PARK7
GeneCard	RET
GeneCard	ABCA1
GeneCard	IL1B
GeneCard	SORL1
GeneCard	TREM2
GeneCard	CYBB
GeneCard	ABCA4
GeneCard	GDAP1
GeneCard	ADAM10
GeneCard	UNC5C
GeneCard	MIR107
GeneCard	PINK1
GeneCard	MTHFR
GeneCard	DYNC1H1
GeneCard	GJB1
GeneCard	DNM2
GeneCard	MIR328
GeneCard	HLA-DRB1
GeneCard	BDNF
GeneCard	PSAP
GeneCard	PMP22
GeneCard	GFAP
GeneCard	RYR1
GeneCard	AGL

GeneCard	SOD1
GeneCard	NPC2
GeneCard	GDNF
GeneCard	IFNG
GeneCard	GBE1
GeneCard	APBB1
GeneCard	SERPINA3
GeneCard	HTT
GeneCard	GARS1
GeneCard	MME
GeneCard	MIR298
GeneCard	TTR
GeneCard	MT-ND2
GeneCard	TARDBP
GeneCard	HLA-DQB1
GeneCard	INS
GeneCard	G6PC1
GeneCard	BACE1
GeneCard	FIG4
GeneCard	UCHL1
GeneCard	PTEN
GeneCard	FAS
GeneCard	LRP5
GeneCard	GRN
GeneCard	APOA1
GeneCard	CTNNB1
GeneCard	SLC17A5
GeneCard	PON1
GeneCard	CTSD
GeneCard	NAGLU
GeneCard	EDNRB
GeneCard	CFTR
GeneCard	CRP
GeneCard	HLA-B
GeneCard	NOTCH1
GeneCard	SNCB
GeneCard	PPARG
GeneCard	CHAT
GeneCard	LRSAM1
GeneCard	ADAM17
GeneCard	SYNJ1
GeneCard	EGR2
GeneCard	TOMM40
GeneCard	IL1A
GeneCard	TLR4
GeneCard	MT-ATP6
GeneCard	LDLR
GeneCard	AKT1
GeneCard	ALB
GeneCard	TNFRSF1A
GeneCard	CYBA
GeneCard	NDRG1
GeneCard	TLR2
GeneCard	HSD17B10
GeneCard	NCF2
GeneCard	KIF1B
GeneCard	HSPB1
GeneCard	ACHE

GeneCard	LAMP2
GeneCard	SERPINA1
GeneCard	MAPK1
GeneCard	IL4
GeneCard	COMT
GeneCard	SNCAIP
GeneCard	TSC2
GeneCard	GSK3B
GeneCard	EGFR
GeneCard	PLP1
GeneCard	CCL2
GeneCard	CLU
GeneCard	MIR21
GeneCard	LIPA
GeneCard	C9orf72
GeneCard	LRP1
GeneCard	GANAB
GeneCard	IL1RN
GeneCard	SOX10
GeneCard	PHYH
GeneCard	FBN1
GeneCard	VEGFA
GeneCard	CLN3
GeneCard	STAT3
GeneCard	AR
GeneCard	COL4A1
GeneCard	ATXN2
GeneCard	LITAF
GeneCard	CAV3
GeneCard	TNFRSF11A
GeneCard	MMP1
GeneCard	IL2
GeneCard	F2
GeneCard	ESR1
GeneCard	GATA1
GeneCard	CASP3
GeneCard	POLG
GeneCard	DBT
GeneCard	HMOX1
GeneCard	PRKAG2
GeneCard	VHL
GeneCard	BCHE
GeneCard	KRAS
GeneCard	CST3
GeneCard	LPL
GeneCard	CFH
GeneCard	GRIN2B
GeneCard	REN
GeneCard	ERBB2
GeneCard	HEXB
GeneCard	PIK3CA
GeneCard	EDN3
GeneCard	ABCB1
GeneCard	NDP
GeneCard	GALC
GeneCard	CDK5
GeneCard	APC
GeneCard	JAK2

GeneCard	PSENEN
GeneCard	OPTN
GeneCard	ATXN3
GeneCard	CXCL8
GeneCard	PRKCSH
GeneCard	HLA-DQA1
GeneCard	BRAF
GeneCard	SPTLC1
GeneCard	TNFRSF11B
GeneCard	ASPA
GeneCard	CASR
GeneCard	NCF1
GeneCard	BRCA1
GeneCard	MT-CYB
GeneCard	HRAS
GeneCard	MIR132
GeneCard	CP
GeneCard	GATA3
GeneCard	PHKA2
GeneCard	MT-CO1
GeneCard	NTRK1
GeneCard	IL23R
GeneCard	JUP
GeneCard	MEFV
GeneCard	MTOR
GeneCard	MIR15A
GeneCard	APOB
GeneCard	PICALM
GeneCard	PTGS2
GeneCard	ACTA2
GeneCard	ATP2A2
GeneCard	TH
GeneCard	JAG1
GeneCard	TF
GeneCard	CDKN2A
GeneCard	NEFH
GeneCard	MIR17
GeneCard	HSPB8
GeneCard	RAB7A
GeneCard	MMP9
GeneCard	PTPN11
GeneCard	CASP8
GeneCard	AGT
GeneCard	CR1
GeneCard	NOS2
GeneCard	DYRK1A
GeneCard	NF1
GeneCard	PON2
GeneCard	LEP
GeneCard	MT-ND6
GeneCard	NFKB1
GeneCard	COL1A1
GeneCard	NCSTN
GeneCard	NR4A2
GeneCard	PLA2G6
GeneCard	CCR6
GeneCard	HTR2A
GeneCard	DSP

GeneCard	MT-ND3
GeneCard	TTN
GeneCard	GAPDH
GeneCard	PYGL
GeneCard	SMAD4
GeneCard	TBP
GeneCard	CD40
GeneCard	NCF4
GeneCard	NGF
GeneCard	VPS13C
GeneCard	FUS
GeneCard	ICAM1
GeneCard	SAG
GeneCard	PRPH2
GeneCard	AGER
GeneCard	ATM
GeneCard	MIR155
GeneCard	GRIN2A
GeneCard	INSR
GeneCard	SMN1
GeneCard	GYS1
GeneCard	EPM2A
GeneCard	MMP3
GeneCard	MAP2K1
GeneCard	ATP7A
GeneCard	IDE
GeneCard	VPS35
GeneCard	YARS1
GeneCard	MIR210
GeneCard	TBK1
GeneCard	PPARGC1A
GeneCard	COL4A4
GeneCard	CCR5
GeneCard	ERBB4
GeneCard	HLA-A
GeneCard	AGTR1
GeneCard	IL18
GeneCard	G6PD
GeneCard	MECP2
GeneCard	GRIN1
GeneCard	MKS1
GeneCard	TPP1
GeneCard	SLC6A4
GeneCard	HNRNPA1
GeneCard	IL17A
GeneCard	FCGR2A
GeneCard	COL4A5
GeneCard	SERPINE1
GeneCard	CYP27A1
GeneCard	IGF1
GeneCard	LRP6
GeneCard	MT-ND5
GeneCard	ATP13A2
GeneCard	PARK16
GeneCard	BPTF
GeneCard	BEST1
GeneCard	TGFBR2
GeneCard	RAF1

GeneCard	NLRP3
GeneCard	DNAJB2
GeneCard	CTSB
GeneCard	SMAD3
GeneCard	TSC1
GeneCard	ASAH1
GeneCard	CD4
GeneCard	MIR143
GeneCard	ECE1
GeneCard	TMEM67
GeneCard	SLC6A3
GeneCard	CYP2D6
GeneCard	SST
GeneCard	HTRA2
GeneCard	CD36
GeneCard	PARK10
GeneCard	FN1
GeneCard	CHMP2B
GeneCard	CAT
GeneCard	DNM1L
GeneCard	F5
GeneCard	DRD2
GeneCard	RELN
GeneCard	DLD
GeneCard	FGFR3
GeneCard	FLNA
GeneCard	SLC11A1
GeneCard	COL25A1
GeneCard	COL17A1
GeneCard	RBP4
GeneCard	DNAJB11
GeneCard	SYP
GeneCard	STAT1
GeneCard	MAOB
GeneCard	HNRNPA2B1
GeneCard	SMAD6
GeneCard	CETP
GeneCard	DCTN1
GeneCard	CACNA1C
GeneCard	GNAQ
GeneCard	DYNC2H1
GeneCard	CXCL12
GeneCard	PARK12
GeneCard	PIK3R1
GeneCard	GIGYF2
GeneCard	GNAS
GeneCard	IL2RA
GeneCard	RPS27A
GeneCard	ITPR1
GeneCard	IRS1
GeneCard	MTR
GeneCard	BACE2
GeneCard	DRD3
GeneCard	SCARB2
GeneCard	NAGA
GeneCard	CDK5R1
GeneCard	NHLRC1
GeneCard	GSTM1

GeneCard	ELN
GeneCard	VDR
GeneCard	MT-CO2
GeneCard	NOTCH2
GeneCard	BIN1
GeneCard	DKK1
GeneCard	CCL5
GeneCard	IL13
GeneCard	REST
GeneCard	BAX
GeneCard	DZIP1L
GeneCard	GLE1
GeneCard	KCNQ1
GeneCard	AHI1
GeneCard	HSPD1
GeneCard	TRIM2
GeneCard	CYP19A1
GeneCard	MYBPC3
GeneCard	FGFR2
GeneCard	LCAT
GeneCard	HMGCR
GeneCard	CYCS
GeneCard	PARK21
GeneCard	DNAJC6
GeneCard	SPTLC2
GeneCard	COPA
GeneCard	SDHB
GeneCard	NOS1
GeneCard	PLD3
GeneCard	GP1BA
GeneCard	ENPP1
GeneCard	MT-ND4
GeneCard	C4A
GeneCard	B2M
GeneCard	PAX2
GeneCard	TNFRSF1B
GeneCard	CDH1
GeneCard	FGFR1
GeneCard	TERT
GeneCard	SCN1A
GeneCard	NTRK2
GeneCard	KARS1
GeneCard	TLR5
GeneCard	GSTP1
GeneCard	MMACHC
GeneCard	PINK1-AS
GeneCard	MMP2
GeneCard	NOTCH3
GeneCard	CDH23
GeneCard	HLA-DPB1
GeneCard	ACTA1
GeneCard	IQCB1
GeneCard	IDUA
GeneCard	PRKCD
GeneCard	CD40LG
GeneCard	ARSA
GeneCard	MT-TK
GeneCard	GJA1

GeneCard	KRT5
GeneCard	IL21
GeneCard	FBXO7
GeneCard	OLR1
GeneCard	SDHC
GeneCard	RHO
GeneCard	PPARA
GeneCard	UBB
GeneCard	DNAJC13
GeneCard	HARS1
GeneCard	POLR2F
GeneCard	MIF
GeneCard	C3
GeneCard	PPP3CA
GeneCard	IL1R1
GeneCard	CACNA1A
GeneCard	MYH9
GeneCard	NDUFS4
GeneCard	PRKG1
GeneCard	F11
GeneCard	GLB1
GeneCard	NRAS
GeneCard	IL12B
GeneCard	AIFM1
GeneCard	ABL1
GeneCard	VLDLR
GeneCard	TFAM
GeneCard	DMD
GeneCard	CSF1R
GeneCard	PIN1
GeneCard	POMC
GeneCard	APOL1
GeneCard	ABCB11
GeneCard	MT-TL1
GeneCard	PRKCQ
GeneCard	ATG16L1
GeneCard	EIF2AK4
GeneCard	CCND1
GeneCard	NPPB
GeneCard	ENG
GeneCard	ABCD1
GeneCard	CASP1
GeneCard	ACTB
GeneCard	RUNX1
GeneCard	WT1
GeneCard	CREBBP
GeneCard	SOD2
GeneCard	FASLG
GeneCard	MAPK14
GeneCard	EDN1
GeneCard	PEX6
GeneCard	AD9
GeneCard	CSF1
GeneCard	ITCH
GeneCard	PRMT7
GeneCard	LPA
GeneCard	TGFB2
GeneCard	SDHD

GeneCard	FLG
GeneCard	COL4A3
GeneCard	STAT4
GeneCard	CSTB
GeneCard	ADIPOQ
GeneCard	NRG1
GeneCard	CACNA1F
GeneCard	RYR2
GeneCard	NLRP1
GeneCard	EPO
GeneCard	SFTPC
GeneCard	ALOX5
GeneCard	PMM2
GeneCard	EIF2AK2
GeneCard	MIR93
GeneCard	CDKN2B-AS1
GeneCard	CYP46A1
GeneCard	BBS1
GeneCard	CLN6
GeneCard	MT-CO3
GeneCard	PRPS1
GeneCard	LEPR
GeneCard	MAPK3
GeneCard	SLC2A1
GeneCard	PON3
GeneCard	PHKG2
GeneCard	CALHM1
GeneCard	PEX7
GeneCard	S100B
GeneCard	FGF23
GeneCard	CX3CR1
GeneCard	ELANE
GeneCard	EIF2AK3
GeneCard	ADH1C
GeneCard	CREB1
GeneCard	ZEB2
GeneCard	MIR140
GeneCard	MAPK8
GeneCard	RELA
GeneCard	SERPINC1
GeneCard	H2AC18
GeneCard	RIPK1
GeneCard	F7
GeneCard	TUBB
GeneCard	ADAMTS4
GeneCard	CAV1
GeneCard	CASP9
GeneCard	KCNH2
GeneCard	GRIA1
GeneCard	SPP1
GeneCard	TMEM106B
GeneCard	BSND
GeneCard	ATXN8OS
GeneCard	DNAJC5
GeneCard	CD28
GeneCard	CTNS
GeneCard	GAB2
GeneCard	CHRNA7

GeneCard	LDHA
GeneCard	PDGFRB
GeneCard	APOC1
GeneCard	PACRG
GeneCard	TNFAIP3
GeneCard	EDNRA
GeneCard	SIRT1
GeneCard	THBD
GeneCard	USH2A
GeneCard	TWNK
GeneCard	SLC1A2
GeneCard	PDCD1
GeneCard	FMR1
GeneCard	LMX1B
GeneCard	HP
GeneCard	ADAMTS13
GeneCard	WFS1
GeneCard	DHCR24
GeneCard	FTL
GeneCard	MBL2
GeneCard	SRC
GeneCard	CXCR4
GeneCard	DOCK3
GeneCard	SELP
GeneCard	ABCG5
GeneCard	SLC25A4
GeneCard	CAPN1
GeneCard	GSR
GeneCard	CHCHD10
GeneCard	SOS1
GeneCard	CAMK2A
GeneCard	SPG7
GeneCard	EGF
GeneCard	LRAT
GeneCard	HSPA5
GeneCard	ADA
GeneCard	SMN2
GeneCard	PRF1
GeneCard	HTRA1
GeneCard	IFNGR1
GeneCard	CCL11
GeneCard	CNTNAP2
GeneCard	ICOSLG
GeneCard	EP300
GeneCard	ZAP70
GeneCard	IRS2
GeneCard	LTA
GeneCard	UBQLN1
GeneCard	MAPK10
GeneCard	PDE11A
GeneCard	TGFBR1
GeneCard	IGF1R
GeneCard	ITM2B
GeneCard	MIR22
GeneCard	SCN2A
GeneCard	PNKP
GeneCard	PAH
GeneCard	AFG3L2

GeneCard	CPT2
GeneCard	ITGB3
GeneCard	IL12A
GeneCard	GPT
GeneCard	DPYSL2
GeneCard	MIR320A
GeneCard	CLN5
GeneCard	MYC
GeneCard	GRIA3
GeneCard	TCF4
GeneCard	GSK3A
GeneCard	JPH3
GeneCard	SNAP25
GeneCard	APOC3
GeneCard	PDYN
GeneCard	MIR122
GeneCard	BAG3
GeneCard	GRK1
GeneCard	IL6R
GeneCard	PANK2
GeneCard	MIR223
GeneCard	TYR
GeneCard	MIR29C
GeneCard	TUBB4A
GeneCard	PTCH1
GeneCard	CHUK
GeneCard	TUBB3
GeneCard	GAL
GeneCard	SELE
GeneCard	SNCG
GeneCard	NRTN
GeneCard	F13A1
GeneCard	BECN1
GeneCard	ADRB2
GeneCard	PRTN3
GeneCard	FYN
GeneCard	ATP2C1
GeneCard	CSNK2A1
GeneCard	CCL3
GeneCard	CHRNA4
GeneCard	NR2E3
GeneCard	GNB4
GeneCard	ZFYVE26
GeneCard	EIF4G1
GeneCard	COX5A
GeneCard	SDHA
GeneCard	APH1A
GeneCard	MIR125A
GeneCard	CYP2C9
GeneCard	ACAN
GeneCard	ITGAM
GeneCard	VDAC1
GeneCard	STXBP1
GeneCard	EXOC3L2
GeneCard	IL7R
GeneCard	BICC1
GeneCard	HAMP
GeneCard	ITGA2B

GeneCard	F12
GeneCard	MAP2
GeneCard	ATF6
GeneCard	RLBP1
GeneCard	HNF4A
GeneCard	GHRL
GeneCard	HBA1
GeneCard	IKBKB
GeneCard	DDIT3
GeneCard	GRK2
GeneCard	NDUFAF6
GeneCard	NR3C1
GeneCard	CTNNA3
GeneCard	VCL
GeneCard	NGFR
GeneCard	SMC1A
GeneCard	PEX1
GeneCard	ERCC2
GeneCard	NFKBIA
GeneCard	DLG4
GeneCard	NEK8
GeneCard	AQP4
GeneCard	NR1H4
GeneCard	SETBP1
GeneCard	PLCB1
GeneCard	TNNI3
GeneCard	CACNA1D
GeneCard	CFAP410
GeneCard	PRKACA
GeneCard	LOX
GeneCard	MFSD8
GeneCard	NTF3
GeneCard	F3
GeneCard	CALM1
GeneCard	PCDH11X
GeneCard	SLC1A3
GeneCard	SH2D1A
GeneCard	LIPC
GeneCard	LMNB1
GeneCard	MT-ATP8
GeneCard	PNPLA3
GeneCard	CD2AP
GeneCard	DBN1
GeneCard	NDUFV1
GeneCard	IGF2
GeneCard	ITPR3
GeneCard	GLUL
GeneCard	SYK
GeneCard	HIF1A
GeneCard	GCH1
GeneCard	ARSB
GeneCard	MSH6
GeneCard	ACTC1
GeneCard	FARSB
GeneCard	APLP2
GeneCard	RPGR
GeneCard	RBCK1
GeneCard	FGF2

GeneCard	GUSB
GeneCard	MIR144
GeneCard	VCAM1
GeneCard	GALNS
GeneCard	PVALB
GeneCard	MPL
GeneCard	MT-ND4L
GeneCard	IFNA1
GeneCard	CSF3
GeneCard	VPS26A
GeneCard	KIF5A
GeneCard	CDH2
GeneCard	MIR433
GeneCard	CSF2
GeneCard	COX4I1
GeneCard	COG2
GeneCard	MKKS
GeneCard	CHI3L1
GeneCard	MSH2
GeneCard	VAPB
GeneCard	XDH
GeneCard	CAST
GeneCard	FZD4
GeneCard	TNFSF11
GeneCard	EMD
GeneCard	P2RY12
GeneCard	GRIA2
GeneCard	CCR1
GeneCard	VPS13B
GeneCard	CACNA1S
GeneCard	DES
GeneCard	DRD1
GeneCard	PKP2
GeneCard	UBC
GeneCard	CASP7
GeneCard	DHTKD1
GeneCard	FGA
GeneCard	CALR
GeneCard	GLUD2
GeneCard	KCNV2
GeneCard	APAF1
GeneCard	MAOA
GeneCard	GAD2
GeneCard	PAX6
GeneCard	GLUD1
GeneCard	SP1
GeneCard	STH
GeneCard	ABCC8
GeneCard	BCL2
GeneCard	PTH
GeneCard	AGTR2
GeneCard	SREBF1
GeneCard	BMP2
GeneCard	SERPINI1
GeneCard	PPT1
GeneCard	CD79A
GeneCard	JUN
GeneCard	PKD2L1

GeneCard	MT3
GeneCard	MAP1B
GeneCard	ALPL
GeneCard	PDE8B
GeneCard	ENO3
GeneCard	APOD
GeneCard	CHRNB2
GeneCard	SGPL1
GeneCard	MITF
GeneCard	F10
GeneCard	FADD
GeneCard	SIGMAR1
GeneCard	JCAD
GeneCard	PCSK9
GeneCard	SPATA22
GeneCard	IFIH1
GeneCard	TIMP1
GeneCard	AQP1
GeneCard	CHEK2
GeneCard	MAPK8IP1
GeneCard	CLCN7
GeneCard	CXCL10
GeneCard	KIF1A
GeneCard	PITX2
GeneCard	DLST
GeneCard	BGLAP
GeneCard	FOLH1
GeneCard	MYLK
GeneCard	IRGM
GeneCard	UBQLN2
GeneCard	PLG
GeneCard	PRODH
GeneCard	TOR1A
GeneCard	TET2
GeneCard	MIR133B
GeneCard	NFE2L2
GeneCard	RYR3
GeneCard	APOA5
GeneCard	BTNL2
GeneCard	KIF11
GeneCard	HSPA4
GeneCard	SLC5A7
GeneCard	CNTNAP1
GeneCard	PLAT
GeneCard	ETV6
GeneCard	RCAN1
GeneCard	OPA1
GeneCard	HLA-DRB5
GeneCard	CLSTN1
GeneCard	EIF2S1
GeneCard	RETN
GeneCard	TFRC
GeneCard	MIR182
GeneCard	PTPRC
GeneCard	MAP3K5
GeneCard	TLR3
GeneCard	TEK
GeneCard	XIAP

GeneCard	ATP1A1
GeneCard	PDK3
GeneCard	GPC1
GeneCard	SCO1
GeneCard	GNPTAB
GeneCard	HPRT1
GeneCard	ARID1B
GeneCard	CHIT1
GeneCard	GNB3
GeneCard	KL
GeneCard	DAPK1
GeneCard	MBP
GeneCard	NRXN1
GeneCard	CFHR2
GeneCard	CAPN2
GeneCard	KCNJ11
GeneCard	FCGR2B
GeneCard	SLC19A1
GeneCard	MIR98
GeneCard	CRYAA
GeneCard	HRH2
GeneCard	NODAL
GeneCard	TSPO
GeneCard	RUNX2
GeneCard	DCDC2
GeneCard	CRH
GeneCard	CBS
GeneCard	DRD4
GeneCard	TPO
GeneCard	S100A9
GeneCard	HNF1A
GeneCard	MIR30E
GeneCard	CD19
GeneCard	FCGR3A
GeneCard	MIR20A
GeneCard	DISC1
GeneCard	LRP8
GeneCard	GRIN2D
GeneCard	NDUFV2
GeneCard	ERN1
GeneCard	HADHA
GeneCard	TBC1D24
GeneCard	FKTN
GeneCard	CSNK1D
GeneCard	GNE
GeneCard	MIRLET7I
GeneCard	NPPA
GeneCard	ATXN1
GeneCard	NDUFS2
GeneCard	IAPP
GeneCard	SCO2
GeneCard	GRM5
GeneCard	MYD88
GeneCard	CCN2
GeneCard	FOS
GeneCard	CDK1
GeneCard	MIR142
GeneCard	COL4A2

GeneCard	CIB1
GeneCard	COL11A2
GeneCard	PMPCA
GeneCard	UBE2L3
GeneCard	TNNT2
GeneCard	APBA1
GeneCard	SLC2A2
GeneCard	SERPINH1
GeneCard	POLR1C
GeneCard	COL9A2
GeneCard	WDR45
GeneCard	FLNC
GeneCard	NR3C2
GeneCard	TLR9
GeneCard	GRIN2C
GeneCard	SAR1B
GeneCard	KCNN4
GeneCard	MEF2A
GeneCard	NOX1
GeneCard	MATR3
GeneCard	HSPG2
GeneCard	ENO2
GeneCard	SETX
GeneCard	SLC22A5
GeneCard	COQ2
GeneCard	MMP13
GeneCard	MIR34C
GeneCard	DNMT1
GeneCard	SPAST
GeneCard	PLA2G4A
GeneCard	NPY
GeneCard	PTPA
GeneCard	SEMA3C
GeneCard	COL6A1
GeneCard	VSNL1
GeneCard	GNB1
GeneCard	COX6A1
GeneCard	TUBA4A
GeneCard	PEX2
GeneCard	UBE3A
GeneCard	MIR221
GeneCard	ESR2
GeneCard	TERC
GeneCard	PITRM1
GeneCard	CACNA1G
GeneCard	FRMD4A
GeneCard	LBR
GeneCard	BBS4
GeneCard	CUBN
GeneCard	CALCA
GeneCard	RAG1
GeneCard	STAT5B
GeneCard	ADNP
GeneCard	APBA2
GeneCard	SLC18A3
GeneCard	PAWR
GeneCard	GSTO1
GeneCard	FOXG1

GeneCard	PODXL
GeneCard	GGT1
GeneCard	DBH
GeneCard	CHGA
GeneCard	MSRA
GeneCard	RB1CC1
GeneCard	NAE1
GeneCard	TFCP2
GeneCard	BMP6
GeneCard	ERCC1
GeneCard	TLR1
GeneCard	NEFM
GeneCard	HCRT
GeneCard	AXIN1
GeneCard	SETD2
GeneCard	BAD
GeneCard	CIITA
GeneCard	FXN
GeneCard	SERPINF1
GeneCard	PDGFRA
GeneCard	ATF4
GeneCard	SLC18A2
GeneCard	LOC108663987
GeneCard	PDGFB
GeneCard	NRGN
GeneCard	CD14
GeneCard	UGT1A1
GeneCard	SLC40A1
GeneCard	DDC
GeneCard	MARK1
GeneCard	BMP4
GeneCard	GPX1
GeneCard	MTRR
GeneCard	KCNMA1
GeneCard	SYNE1
GeneCard	KCNQ2
GeneCard	CASP6
GeneCard	TGM2
GeneCard	SFTPA2
GeneCard	EPOR
GeneCard	TUBA1B
GeneCard	TUBB2A
GeneCard	PLA2G7
GeneCard	BCL11A
GeneCard	HMGB1
GeneCard	L1CAM
GeneCard	KCNIP3
GeneCard	ACTN4
GeneCard	ITGB1
GeneCard	U2AF1
GeneCard	PRL
GeneCard	YARS2
GeneCard	SPTAN1
GeneCard	GMPPB
GeneCard	LDB3
GeneCard	NEK9
GeneCard	BTK
GeneCard	NCAM1

GeneCard	COL18A1
GeneCard	ADAR
GeneCard	HTR2C
GeneCard	ANG
GeneCard	MIR30A
GeneCard	SCN8A
GeneCard	GSAP
GeneCard	FLT1
GeneCard	JAK3
GeneCard	DYSF
GeneCard	IDH1
GeneCard	WNT1
GeneCard	SFTPA1
GeneCard	CRYAB
GeneCard	SOX2
GeneCard	SLC4A1
GeneCard	PLEC
GeneCard	LOC109504728
GeneCard	KNG1
GeneCard	ALS2
GeneCard	PCSK1N
GeneCard	CH25H
GeneCard	IL4R
GeneCard	CD27
GeneCard	CDKN2B
GeneCard	ERBB3
GeneCard	ATN1
GeneCard	IL37
GeneCard	HSP90AA1
GeneCard	SOX9
GeneCard	GCK
GeneCard	MIR27A
GeneCard	CFB
GeneCard	PTGS1
GeneCard	EDAR
GeneCard	CARD8
GeneCard	PAFAH1B1
GeneCard	SLC25A16
GeneCard	CAMK2G
GeneCard	PFN1
GeneCard	PIK3C3
GeneCard	INA
GeneCard	MIR197
GeneCard	LTF
GeneCard	OGT
GeneCard	NRP1
GeneCard	MYO9B
GeneCard	GSTO2
GeneCard	CHCHD2
GeneCard	TBX1
GeneCard	MARK4
GeneCard	RTN4
GeneCard	KITLG
GeneCard	TNFSF15
GeneCard	AFP
GeneCard	SYNGAP1
GeneCard	C4B
GeneCard	MIR9-1

GeneCard	MBD5
GeneCard	TCTN2
GeneCard	IFNGR2
GeneCard	LOC109461479
GeneCard	ALDOA
GeneCard	TYROBP
GeneCard	SPARC
GeneCard	MTHFD1L
GeneCard	GAP43
GeneCard	CDKN1A
GeneCard	SAA1
GeneCard	DCTN4
GeneCard	BLMH
GeneCard	LRP2
GeneCard	DKK4
GeneCard	MIR483
GeneCard	MIR31
GeneCard	APLP1
GeneCard	BACE1-AS
GeneCard	NDE1
GeneCard	KDR
GeneCard	CHRM1
GeneCard	JAK1
GeneCard	PEX3
GeneCard	SORCS3
GeneCard	NALCN
GeneCard	RB1
GeneCard	TAB2
GeneCard	STK11
GeneCard	AKR1B1
GeneCard	DKK2
GeneCard	SMAD2
GeneCard	APH1B
GeneCard	IKZF1
GeneCard	PTGDS
GeneCard	VIM
GeneCard	PPP3R1
GeneCard	KCNA2
GeneCard	MEF2C
GeneCard	APBB2
GeneCard	C10orf55
GeneCard	ANK3
GeneCard	DNMT3A
GeneCard	LRRC56
GeneCard	HLA-G
GeneCard	ITPR2
GeneCard	MIR26B
GeneCard	OTC
GeneCard	CYP2E1
GeneCard	TJP2
GeneCard	MED12
GeneCard	PECAM1
GeneCard	PPIF
GeneCard	GABRG2
GeneCard	ASXL1
GeneCard	APOH
GeneCard	UQCRC1
GeneCard	MAPK9

GeneCard	NQO1
GeneCard	CR2
GeneCard	TANGO2
GeneCard	KLC1
GeneCard	CDKN1B
GeneCard	EYS
GeneCard	PLA2G2A
GeneCard	PTH1R
GeneCard	IAH1
GeneCard	NECTIN2
GeneCard	FHL1
GeneCard	MSR1
GeneCard	GH1
GeneCard	C5
GeneCard	MT-TS1
GeneCard	MIR222
GeneCard	KRT1
GeneCard	LRPAP1
GeneCard	CPS1
GeneCard	SELL
GeneCard	ATP1A3
GeneCard	AD15
GeneCard	APOA2
GeneCard	TRAF3IP1
GeneCard	SET
GeneCard	PCDH15
GeneCard	TNFSF13B
GeneCard	VDAC2
GeneCard	POGZ
GeneCard	GAD1
GeneCard	GLIS2
GeneCard	NPEPPS
GeneCard	KRT18
GeneCard	NDUFS3
GeneCard	EXOSC3
GeneCard	PEX26
GeneCard	TP63
GeneCard	ADCYAP1
GeneCard	IL15
GeneCard	PEX19
GeneCard	PIK3R4
GeneCard	FANCD2
GeneCard	CYP17A1
GeneCard	PSMC6
GeneCard	SERPINF2
GeneCard	MIR128-1
GeneCard	IGFBP3
GeneCard	GSN
GeneCard	KRT8
GeneCard	CD55
GeneCard	TCIRG1
GeneCard	GSTT1
GeneCard	LCN2
GeneCard	KRT14
GeneCard	NOX4
GeneCard	PEX16
GeneCard	SLC16A2
GeneCard	RAD51C

GeneCard	CBL
GeneCard	IARS2
GeneCard	PLOD1
GeneCard	SEMA3A
GeneCard	ADAM12
GeneCard	MET
GeneCard	HSPA8
GeneCard	HK1
GeneCard	ITGA4
GeneCard	ANXA11
GeneCard	TREX1
GeneCard	CYP7B1
GeneCard	B9D1
GeneCard	SORCS1
GeneCard	ANKH
GeneCard	SLC26A4
GeneCard	TRPC6
GeneCard	TUBA1A
GeneCard	KLK8
GeneCard	CXCR3
GeneCard	KANSL1
GeneCard	EPCAM
GeneCard	MIR200B
GeneCard	ASAH2
GeneCard	LAMA2
GeneCard	EBF3
GeneCard	ADK
GeneCard	SPR
GeneCard	REG1A
GeneCard	STX1A
GeneCard	MMUT
GeneCard	CD46
GeneCard	SPATA7
GeneCard	COL6A2
GeneCard	NDUFS1
GeneCard	SFTPD
GeneCard	IL7
GeneCard	PRPH
GeneCard	TFG
GeneCard	PADI2
GeneCard	MIR200A
GeneCard	WARS1
GeneCard	RETREG1
GeneCard	FANCI
GeneCard	ALDH2
GeneCard	DCTN2
GeneCard	STX3
GeneCard	IL3
GeneCard	PNMT
GeneCard	PIK3CD
GeneCard	HDAC9
GeneCard	FGF8
GeneCard	CCR2
GeneCard	PRSS1
GeneCard	MAG
GeneCard	HBG2
GeneCard	CDC42
GeneCard	XRCC1

GeneCard	NDUFAF2
GeneCard	RPS6KA3
GeneCard	NEU1
GeneCard	MEOX2
GeneCard	ATG13
GeneCard	MIR127
GeneCard	KAT6B
GeneCard	S100A8
GeneCard	PHEX
GeneCard	AKT2
GeneCard	DDX58
GeneCard	MMP12
GeneCard	BLOC1S3
GeneCard	FLI1
GeneCard	MIR141
GeneCard	GHR
GeneCard	CNTF
GeneCard	IL33
GeneCard	ATP2A1
GeneCard	TUBA3D
GeneCard	WWOX
GeneCard	H19
GeneCard	NR1H3
GeneCard	KALRN
GeneCard	IL23A
GeneCard	AFF2
GeneCard	BCL2L1
GeneCard	GSS
GeneCard	SMARCA2
GeneCard	HNMT
GeneCard	CYP11B2
GeneCard	MIR181A1
GeneCard	CYP1A1
GeneCard	MT-TF
GeneCard	PURA
GeneCard	SLC2A3
GeneCard	CTSK
GeneCard	MUTYH
GeneCard	DDX3X
GeneCard	GSTM3
GeneCard	MMP14
GeneCard	ACTG1
GeneCard	DBNL
GeneCard	SNAP29
GeneCard	AVP
GeneCard	F11-AS1
GeneCard	MIR10A
GeneCard	FBLN5
GeneCard	ITGB2
GeneCard	TPMT
GeneCard	GCG
GeneCard	ANKS6
GeneCard	TAF1
GeneCard	ADORA2A
GeneCard	SLC52A3
GeneCard	CD34
GeneCard	MBTPS2
GeneCard	LIFR

GeneCard	NAT2
GeneCard	CSNK1A1
GeneCard	MDM2
GeneCard	MAZ
GeneCard	CD80
GeneCard	SLC25A13
GeneCard	CLTC
GeneCard	ARPC1B
GeneCard	ISG15
GeneCard	WNK1
GeneCard	DNAH11
GeneCard	PSMC2
GeneCard	KIF5B
GeneCard	PSMD4
GeneCard	AMBRA1
GeneCard	GBA2
GeneCard	DNAH8
GeneCard	FGF20
GeneCard	PROS1
GeneCard	DCR
GeneCard	EPRS1
GeneCard	OPN4
GeneCard	NRXN3
GeneCard	CYP3A4
GeneCard	ATL1
GeneCard	MCOLN1
GeneCard	DNTT
GeneCard	HLA-DPA1
GeneCard	RTN3
GeneCard	VRK1
GeneCard	TGIF1
GeneCard	MT-TT
GeneCard	NDUFA13
GeneCard	CSNK1A1L
GeneCard	PLCE1
GeneCard	ATP5F1C
GeneCard	SHANK3
GeneCard	DNMT3B
GeneCard	MRE11
GeneCard	PEBP1
GeneCard	CEP164
GeneCard	RD3
GeneCard	PARP1
GeneCard	YAP1
GeneCard	FH
GeneCard	CALML3
GeneCard	PAX3
GeneCard	SLC2A9
GeneCard	NCKAP1
GeneCard	DAO
GeneCard	GPR3
GeneCard	FKBP10
GeneCard	HGF
GeneCard	NDUFA6
GeneCard	WNT8B
GeneCard	BRF1
GeneCard	FGG
GeneCard	EIF2B5

GeneCard	CHRNA3
GeneCard	PSMC1
GeneCard	CCNF
GeneCard	MIR200C
GeneCard	KCNJ10
GeneCard	NTRK3
GeneCard	IRAK4
GeneCard	BCR
GeneCard	TMEM126B
GeneCard	MIR214
GeneCard	TM2D1
GeneCard	PPID
GeneCard	SON
GeneCard	ATXN7
GeneCard	NDUFS7
GeneCard	MFSD2A
GeneCard	PEX14
GeneCard	MRC1
GeneCard	MIR181C
GeneCard	DVL1
GeneCard	FGB
GeneCard	YWHAQ
GeneCard	PIK3R2
GeneCard	STN1
GeneCard	LAMP1
GeneCard	GRM1
GeneCard	EPHA4
GeneCard	MIAT
GeneCard	USP8
GeneCard	PIK3C2A
GeneCard	MAGI2
GeneCard	TPM3
GeneCard	MT-TV
GeneCard	HDAC6
GeneCard	HOTAIR
GeneCard	CEACAM6
GeneCard	ULK1
GeneCard	FLNB
GeneCard	DSG1
GeneCard	CCK
GeneCard	C19orf12
GeneCard	DSC2
GeneCard	KCNJ5
GeneCard	SPPL2A
GeneCard	SOX2-OT
GeneCard	ATG14
GeneCard	TPH1
GeneCard	IGF2R
GeneCard	SLC6A1
GeneCard	OGG1
GeneCard	CDHR1
GeneCard	MCU
GeneCard	FZD6
GeneCard	BLVRA
GeneCard	KLK3
GeneCard	BGN
GeneCard	NDUFA1
GeneCard	CDK4

GeneCard	DKC1
GeneCard	NF2
GeneCard	HTR7
GeneCard	FLOT1
GeneCard	AIF1
GeneCard	CD33
GeneCard	IREB2
GeneCard	CCNB1
GeneCard	TSPAN12
GeneCard	STAR
GeneCard	TCF7L2
GeneCard	RAB39B
GeneCard	FLCN
GeneCard	GLRA1
GeneCard	DMPK
GeneCard	MT-TH
GeneCard	PRSS23
GeneCard	HSPA1A
GeneCard	MIR486-1
GeneCard	XPNPEP3
GeneCard	ABCC2
GeneCard	ALDH18A1
GeneCard	CCR3
GeneCard	ANXA5
GeneCard	CA2
GeneCard	TNFRSF21
GeneCard	C1S
GeneCard	COMP
GeneCard	CNR1
GeneCard	NAIP
GeneCard	STUB1
GeneCard	NDUFS8
GeneCard	XBP1
GeneCard	KCNQ3
GeneCard	AHSG
GeneCard	PSMB9
GeneCard	PIGL
GeneCard	MIR195
GeneCard	COX10
GeneCard	GZMB
GeneCard	CHM
GeneCard	HNRNPK
GeneCard	KCNE1
GeneCard	ENO1
GeneCard	CCL4
GeneCard	BCYRN1
GeneCard	RAC1
GeneCard	UNC13A
GeneCard	MGP
GeneCard	AP4M1
GeneCard	MAF
GeneCard	LAMB2
GeneCard	NRBF2
GeneCard	SCARB1
GeneCard	DSG2
GeneCard	MLXIPL
GeneCard	ATP5F1B
GeneCard	ACE2

GeneCard	TIMP2
GeneCard	PSMB8
GeneCard	GFRA1
GeneCard	PRDX1
GeneCard	EIF2B2
GeneCard	TRIM32
GeneCard	ADD1
GeneCard	SYN3
GeneCard	SLPI
GeneCard	IDO1
GeneCard	IL9
GeneCard	TPM1
GeneCard	MYOC
GeneCard	KLK6
GeneCard	PTRH2
GeneCard	PRKCG
GeneCard	PLCG2
GeneCard	MMP8
GeneCard	USP9X
GeneCard	DNASE1
GeneCard	DLAT
GeneCard	TGM1
GeneCard	HADHB
GeneCard	TLR7
GeneCard	TNXB
GeneCard	FPR2
GeneCard	TIA1
GeneCard	WASHC5
GeneCard	EHMT1
GeneCard	OTX2
GeneCard	MKI67
GeneCard	SHBG
GeneCard	PKD1L2
GeneCard	IRAK1
GeneCard	PHF6
GeneCard	TAC1
GeneCard	ADARB2
GeneCard	DPP4
GeneCard	FUCA1
GeneCard	ADCY10
GeneCard	PCNT
GeneCard	DRP2
GeneCard	IGFBP1
GeneCard	TET1
GeneCard	LOC106694315
GeneCard	GDF15
GeneCard	MTFMT
GeneCard	MOG
GeneCard	RHOBTB2
GeneCard	MT-TP
GeneCard	HLA-DRA
GeneCard	DST
GeneCard	ZNF423
GeneCard	PHKG1
GeneCard	CUL4B
GeneCard	NPC1L1
GeneCard	PRDX3
GeneCard	BLM

GeneCard	SLC12A4
GeneCard	RAB27A
GeneCard	NDUFB9
GeneCard	ANK2
GeneCard	HM13
GeneCard	EWSR1
GeneCard	C1R
GeneCard	TCTN3
GeneCard	CD68
GeneCard	SLC17A7
GeneCard	RHD
GeneCard	MB
GeneCard	SMARCB1
GeneCard	TMEM43
GeneCard	SLC29A3
GeneCard	GCLC
GeneCard	DNM1
GeneCard	IMPG1
GeneCard	CLDN16
GeneCard	AGRN
GeneCard	RPL5
GeneCard	NDUFS6
GeneCard	TIMP3
GeneCard	CYP2C19
GeneCard	LGALS3
GeneCard	HTR1A
GeneCard	VIP
GeneCard	NPM1
GeneCard	VDAC3
GeneCard	SLC17A6
GeneCard	MIR124-1
GeneCard	PGR
GeneCard	MAP2K2
GeneCard	CALB1
GeneCard	CALB2
GeneCard	GPHN
GeneCard	PABPN1
GeneCard	PRKCA
GeneCard	ITGA2
GeneCard	IVNS1ABP
GeneCard	MICA
GeneCard	SLC6A2
GeneCard	CCDC40
GeneCard	SFRP4
GeneCard	MIR205
GeneCard	SLC22A12
GeneCard	ANXA7
GeneCard	HBG1
GeneCard	THBS2
GeneCard	SUOX
GeneCard	SOCS1
GeneCard	FRAT1
GeneCard	CD274
GeneCard	FGFR4
GeneCard	SRSF2
GeneCard	MED12L
GeneCard	DTNBP1
GeneCard	ASCL1

GeneCard	PACS1
GeneCard	SORT1
GeneCard	IL21-AS1
GeneCard	PEX11B
GeneCard	MYOCD
GeneCard	AXIN2
GeneCard	MYOT
GeneCard	EEF1A2
GeneCard	PDE4D
GeneCard	USH1C
GeneCard	CFP
GeneCard	AIMP1
GeneCard	HPS6
GeneCard	MIR363
GeneCard	ZMPSTE24
GeneCard	CTCF
GeneCard	UBQLN4
GeneCard	PGM1
GeneCard	MIR15B
GeneCard	BCL6
GeneCard	SLC30A10
GeneCard	GAN
GeneCard	IRF8
GeneCard	M6PR
GeneCard	ERCC4
GeneCard	COL12A1
GeneCard	PRKD1
GeneCard	TAP2
GeneCard	BMPR1A
GeneCard	ERCC8
GeneCard	DLL1
GeneCard	ASTN2
GeneCard	GRIA4
GeneCard	GTPBP4
GeneCard	MIR125B1
GeneCard	SCAPER
GeneCard	MIR148A
GeneCard	CD44
GeneCard	TAP1
GeneCard	ACTN2
GeneCard	TAF15
GeneCard	TFEB
GeneCard	RAB5A
GeneCard	GCDH
GeneCard	CA4
GeneCard	BMP1
GeneCard	LMNB2
GeneCard	FTO
GeneCard	TSLP
GeneCard	COL6A3
GeneCard	MMEL1
GeneCard	LRRTM3
GeneCard	ARL3
GeneCard	MIR24-1
GeneCard	THRB
GeneCard	SIGLEC5
GeneCard	ZEB1
GeneCard	DCN

GeneCard	FURIN
GeneCard	PDE5A
GeneCard	EIF2B4
GeneCard	ARG1
GeneCard	THOP1
GeneCard	NOTCH4
GeneCard	PDX1
GeneCard	MIR34B
GeneCard	ASPM
GeneCard	SLC9A6
GeneCard	GATM
GeneCard	DGUOK
GeneCard	NLGN3
GeneCard	GATA2
GeneCard	PF4
GeneCard	DSCAM
GeneCard	HINT1
GeneCard	NOTCH2NLC
GeneCard	KIR3DL1
GeneCard	UGCG
GeneCard	IL6ST
GeneCard	SGCA
GeneCard	HNRNPU
GeneCard	IL16
GeneCard	ACKR1
GeneCard	TPI1
GeneCard	XPNPEP1
GeneCard	PRPF31
GeneCard	HUWE1
GeneCard	NUP133
GeneCard	NOS1AP
GeneCard	ADM
GeneCard	GLS
GeneCard	CAMK2B
GeneCard	ARMS2
GeneCard	CXCR2
GeneCard	NDUFB3
GeneCard	THBS1
GeneCard	TXN
GeneCard	SNAI2
GeneCard	NDUFA10
GeneCard	CYFIP2
GeneCard	PGF
GeneCard	CTSA
GeneCard	ALOX5AP
GeneCard	NUP205
GeneCard	SPG21
GeneCard	RARB
GeneCard	GNRH1
GeneCard	MIR424
GeneCard	RALGPS2
GeneCard	CTSF
GeneCard	CLUAP1
GeneCard	TMEM119
GeneCard	RHOA
GeneCard	SYN1
GeneCard	CD163
GeneCard	GDF5

GeneCard	FUT2
GeneCard	SGCB
GeneCard	SEMA4G
GeneCard	AKT3
GeneCard	PRKCB
GeneCard	ERCC3
GeneCard	CYP11A1
GeneCard	MIR206
GeneCard	HMBS
GeneCard	TNFSF10
GeneCard	HERC2
GeneCard	ATP5F1A
GeneCard	FOXO3
GeneCard	ABCC9
GeneCard	GALR2
GeneCard	TGFA
GeneCard	KMT2C
GeneCard	TNNI3K
GeneCard	FLOT2
GeneCard	MEG3
GeneCard	SLC10A2
GeneCard	SPTBN2
GeneCard	CNP
GeneCard	AGA
GeneCard	MIR99A
GeneCard	DHX30
GeneCard	ETS1
GeneCard	A2ML1
GeneCard	PDE4A
GeneCard	VKORC1
GeneCard	NMNAT1
GeneCard	BHLHB9
GeneCard	DDOST
GeneCard	NTS
GeneCard	BCL2L2
GeneCard	BAP1
GeneCard	TWIST1
GeneCard	MECOM
GeneCard	ATXN10
GeneCard	PDHA1
GeneCard	LORICRIN
GeneCard	OPA3
GeneCard	WRN
GeneCard	MT-TL2
GeneCard	PPP2R2B
GeneCard	ATP1A2
GeneCard	QRICH1
GeneCard	ATP5F1D
GeneCard	TUBB2B
GeneCard	IL11
GeneCard	CDKN3
GeneCard	KCNB1
GeneCard	CYB5A
GeneCard	LRPPRC
GeneCard	HDAC2
GeneCard	GALK1
GeneCard	CD244
GeneCard	MTRFR

GeneCard	TCOF1
GeneCard	DAOA
GeneCard	UBA1
GeneCard	TCAP
GeneCard	SCGB1A1
GeneCard	MIR335
GeneCard	TRAPPC9
GeneCard	ATRIP
GeneCard	HSPA9
GeneCard	IL19
GeneCard	MIRLET7B
GeneCard	APOC2
GeneCard	HPS1
GeneCard	VCAN
GeneCard	RRAS2
GeneCard	GOSR2
GeneCard	TREM1
GeneCard	IL1RAPL2
GeneCard	CACNB2
GeneCard	MIR30B
GeneCard	TENT5A
GeneCard	CDK8
GeneCard	PROC
GeneCard	P3H1
GeneCard	HAX1
GeneCard	EPHA1
GeneCard	NFIB
GeneCard	KIF1C
GeneCard	NFKB2
GeneCard	FOXO1
GeneCard	SMC3
GeneCard	SHOC2
GeneCard	GDI1
GeneCard	HDAC4
GeneCard	ABCG2
GeneCard	ASS1
GeneCard	GDF2
GeneCard	SLC2A10
GeneCard	TBCK
GeneCard	MTHFD1
GeneCard	CEP63
GeneCard	KRIT1
GeneCard	LOC108663996
GeneCard	PIEZO1
GeneCard	GJB6
GeneCard	S100A12
GeneCard	IL18R1
GeneCard	CALM3
GeneCard	MIR139
GeneCard	POU5F1
GeneCard	CASK
GeneCard	MT-TW
GeneCard	HOXD13
GeneCard	LOC110806262
GeneCard	YWHAE
GeneCard	PRPF8
GeneCard	SAMHD1
GeneCard	PREPL

GeneCard	MSX1
GeneCard	KRT19
GeneCard	GAST
GeneCard	CDON
GeneCard	C1QA
GeneCard	OGDH
GeneCard	ITGA8
GeneCard	CREB3L1
GeneCard	EPHX1
GeneCard	MAP3K7
GeneCard	GHSR
GeneCard	AHR
GeneCard	NR1H2
GeneCard	ABCA12
GeneCard	HAR1A
GeneCard	AP2M1
GeneCard	APRT
GeneCard	TRAF3IP2
GeneCard	ALDH5A1
GeneCard	RTN4R
GeneCard	GC
GeneCard	CRAT
GeneCard	ODAD3
GeneCard	ASCC1
GeneCard	MIR181A2
GeneCard	LINC01080
GeneCard	AHDC1
GeneCard	HESX1
GeneCard	NDUFB11
GeneCard	NUP85
GeneCard	NIPBL
GeneCard	SOS2
GeneCard	SEC23B
GeneCard	MIR16-1
GeneCard	GLRB
GeneCard	NUP93
GeneCard	TGM6
GeneCard	CYP3A5
GeneCard	SNRNP200
GeneCard	CTSG
GeneCard	ITGAL
GeneCard	COX6B1
GeneCard	UGT1A8
GeneCard	PAX5
GeneCard	CSF3R
GeneCard	DEFB4A
GeneCard	ACTG2
GeneCard	CFL2
GeneCard	LRP4
GeneCard	TRPM7
GeneCard	GJA5
GeneCard	FTH1
GeneCard	TFAP2A
GeneCard	FANCC
GeneCard	SCT
GeneCard	GALE
GeneCard	DLG3
GeneCard	AGK

GeneCard	PPBP
GeneCard	GABRB2
GeneCard	SLC11A2
GeneCard	KIFBP
GeneCard	CD81
GeneCard	LINC01616
GeneCard	RAC2
GeneCard	AKAP9
GeneCard	ITGAX
GeneCard	GABRA1
GeneCard	CAPN10
GeneCard	MALAT1
GeneCard	KLHDC8B
GeneCard	FGF10
GeneCard	HADH
GeneCard	SEPTIN9
GeneCard	FOXE1
GeneCard	CXCL1
GeneCard	P2RX7
GeneCard	MMP7
GeneCard	LDLRAP1
GeneCard	NDUFA12
GeneCard	LONP1
GeneCard	CASP2
GeneCard	RPS19
GeneCard	RAPSN
GeneCard	NUP160
GeneCard	MC1R
GeneCard	FA2H
GeneCard	WNT10A
GeneCard	TRAPPC2L
GeneCard	CD59
GeneCard	EPHX2
GeneCard	COL9A1
GeneCard	STX11
GeneCard	OCLN
GeneCard	MYPN
GeneCard	POSTN
GeneCard	MGMT
GeneCard	GATAD2B
GeneCard	KCNN2
GeneCard	LINC01772
GeneCard	PIP
GeneCard	FDPS
GeneCard	SOD3
GeneCard	MT-TS2
GeneCard	NQO2
GeneCard	PLCG1
GeneCard	LIMK1
GeneCard	EFEMP1
GeneCard	FARSA
GeneCard	RNASEH2A
GeneCard	MASP2
GeneCard	PTGIS
GeneCard	HAR1B
GeneCard	GNAO1
GeneCard	SMARCC2
GeneCard	TXNRD2

GeneCard	ERLIN2
GeneCard	STIM1
GeneCard	PHB
GeneCard	PAK3
GeneCard	PLAUR
GeneCard	NDUFA9
GeneCard	ADRB1
GeneCard	CYLD
GeneCard	IL18RAP
GeneCard	CXCL9
GeneCard	CCR7
GeneCard	APOA4
GeneCard	NEAT1
GeneCard	CSNK2B
GeneCard	RNASEH2C
GeneCard	FHIT
GeneCard	UBD
GeneCard	AP4S1
GeneCard	FSHR
GeneCard	PTX3
GeneCard	CCDC115
GeneCard	STX1B
GeneCard	XRCC2
GeneCard	NDUFC2
GeneCard	EGR1
GeneCard	RAB8A
GeneCard	TINF2
GeneCard	HNRNPH2
GeneCard	PITX3
GeneCard	INPP5D
GeneCard	CYP1A2
GeneCard	FGF1
GeneCard	CCL20
GeneCard	KRT10
GeneCard	NLRC4
GeneCard	MIR511
GeneCard	NOD1
GeneCard	FBXL4
GeneCard	WNT4
GeneCard	OLIG2
GeneCard	NAA10
GeneCard	SOCS3
GeneCard	CRADD
GeneCard	MICB
GeneCard	PPP6R2
GeneCard	ELP1
GeneCard	PPP2R1A
GeneCard	NTF4
GeneCard	APEX1
GeneCard	PPOX
GeneCard	SETD1A
GeneCard	VPS11
GeneCard	CFAP418
GeneCard	ZNF292
GeneCard	CTNND2
GeneCard	CANX
GeneCard	RBFOX1
GeneCard	SDC1

GeneCard	WNT5A
GeneCard	TLR6
GeneCard	VAC14
GeneCard	MSTN
GeneCard	TRAF6
GeneCard	SLC6A5
GeneCard	PPP1CB
GeneCard	TRPM3
GeneCard	CEBPA
GeneCard	AP3B1
GeneCard	MIR193B
GeneCard	PRND
GeneCard	ALAD
GeneCard	NDUFB8
GeneCard	STAT2
GeneCard	HMGA1
GeneCard	TTPA
GeneCard	NARS2
GeneCard	ARC
GeneCard	TP73
GeneCard	MIR338
GeneCard	WDR26
GeneCard	SLC12A2
GeneCard	MSMB
GeneCard	CYB5R3
GeneCard	CDK5RAP2
GeneCard	MT-RNR2
GeneCard	CD69
GeneCard	HSPB2
GeneCard	NEUROD1
GeneCard	TCN2
GeneCard	HSD17B4
GeneCard	CDC25A
GeneCard	CNBP
GeneCard	SLC2A4
GeneCard	OCA2
GeneCard	CNGB1
GeneCard	DPP6
GeneCard	AMPD1
GeneCard	KIF3A
GeneCard	IRF4
GeneCard	SMAD7
GeneCard	KCNE3
GeneCard	ABCB7
GeneCard	DHCR7
GeneCard	LRP1-AS
GeneCard	PGK1
GeneCard	NOP56
GeneCard	PCNA
GeneCard	RBPJ
GeneCard	WNT3
GeneCard	FAN1
GeneCard	ACO1
GeneCard	ARF1
GeneCard	GABRB3
GeneCard	DHFR
GeneCard	CALM2
GeneCard	UCP2

GeneCard	POMGNT2
GeneCard	ATP12A
GeneCard	FASTKD2
GeneCard	MIR100
GeneCard	MT-TQ
GeneCard	MEGF10
GeneCard	TYK2
GeneCard	RGR
GeneCard	PRPF3
GeneCard	ATAD1
GeneCard	IGFBP2
GeneCard	SUCLA2
GeneCard	SYN2
GeneCard	MIR326
GeneCard	EZR
GeneCard	MX1
GeneCard	ETFA
GeneCard	GRIK2
GeneCard	GLI1
GeneCard	BMPR1B
GeneCard	RAB7B
GeneCard	TRIM21
GeneCard	BLOC1S1
GeneCard	MIR212
GeneCard	PRR12
GeneCard	PDE6G
GeneCard	WIPF1
GeneCard	MIR193A
GeneCard	PLTP
GeneCard	FOXP2
GeneCard	BMP7
GeneCard	DRD5
GeneCard	EIF2B1
GeneCard	IRF7
GeneCard	SNHG3
GeneCard	ANK1
GeneCard	KCNC3
GeneCard	TIMM8A
GeneCard	RPS10
GeneCard	ABCG1
GeneCard	CAMP
GeneCard	DNAAF2
GeneCard	UCP3
GeneCard	C11orf65
GeneCard	SUFU
GeneCard	MT-RNR1
GeneCard	MIR33A
GeneCard	HAVCR2
GeneCard	MYT1L
GeneCard	OXT
GeneCard	VTN
GeneCard	TPH2
GeneCard	TDGF1
GeneCard	EMC1
GeneCard	LIF
GeneCard	MT-TN
GeneCard	MRPS34
GeneCard	IFNAR1

GeneCard	USH1G
GeneCard	FTSJ1
GeneCard	LIPE
GeneCard	RPL18
GeneCard	SMS
GeneCard	CTSC
GeneCard	ATP6V1B1
GeneCard	SLCO2A1
GeneCard	WASF1
GeneCard	CHRNE
GeneCard	VAMP2
GeneCard	CHRNA2
GeneCard	NR1I2
GeneCard	TBR1
GeneCard	HSPA1L
GeneCard	TRMT10C
GeneCard	ANTXR1
GeneCard	F2R
GeneCard	PSNP3
GeneCard	TYRP1
GeneCard	CCL26
GeneCard	ERG
GeneCard	CX3CL1
GeneCard	SF3B4
GeneCard	SERPING1
GeneCard	ODC1
GeneCard	SACS
GeneCard	SOX3
GeneCard	AHCY
GeneCard	PALS1
GeneCard	MIR455
GeneCard	LTBP2
GeneCard	MIRLET7C
GeneCard	GPNMB
GeneCard	MT-TI
GeneCard	CS
GeneCard	PLCB4
GeneCard	DCX
GeneCard	TAGAP
GeneCard	KEAP1
GeneCard	RAB11A
GeneCard	RPL11
GeneCard	GRP
GeneCard	TECPR2
GeneCard	LYST
GeneCard	ZNF408
GeneCard	SRD5A2
GeneCard	MARK3
GeneCard	PSNP2
GeneCard	HDAC1
GeneCard	CTNNA1
GeneCard	PEPD
GeneCard	SPRY4
GeneCard	MIP
GeneCard	PTPN1
GeneCard	PIK3CG
GeneCard	PAPPA
GeneCard	DIO2

GeneCard	MLH3
GeneCard	UFD1
GeneCard	DEFB1
GeneCard	TACO1
GeneCard	LHCGR
GeneCard	HAP1
GeneCard	ADH1B
GeneCard	MIR659
GeneCard	KRT20
GeneCard	C12orf57
GeneCard	COQ8A
GeneCard	WNT3A
GeneCard	TGFBI
GeneCard	ANXA1
GeneCard	GNAI2
GeneCard	FECH
GeneCard	PADI4
GeneCard	ALPP
GeneCard	MAGED2
GeneCard	ACTN1
GeneCard	RASA1
GeneCard	ENTPD1
GeneCard	SREBF2
GeneCard	AOC3
GeneCard	COX15
GeneCard	SYT1
GeneCard	TRPM1
GeneCard	CDK2
GeneCard	MYL2
GeneCard	C1QB
GeneCard	ARHGEF9
GeneCard	RARA
GeneCard	TANC2
GeneCard	NDUFA2
GeneCard	MMP19
GeneCard	DLL4
GeneCard	VSX2
GeneCard	RPS6KB1
GeneCard	EFTUD2
GeneCard	RSPH4A
GeneCard	CPLX1
GeneCard	CSRP3
GeneCard	TRH
GeneCard	CHRM3
GeneCard	BSG
GeneCard	PRRT2
GeneCard	PRPF6
GeneCard	LGI1
GeneCard	NCR1
GeneCard	DLG5
GeneCard	CGA
GeneCard	MIR324
GeneCard	MYO1E
GeneCard	ADAMTSL1
GeneCard	DLG1
GeneCard	VPS33B
GeneCard	EPG5
GeneCard	TBL1XR1

GeneCard	MYH10
GeneCard	ALDOB
GeneCard	TKT
GeneCard	TNFRSF10A
GeneCard	ADRB3
GeneCard	POLD1
GeneCard	DPAGT1
GeneCard	NME8
GeneCard	LPP
GeneCard	NDUFA11
GeneCard	MIR135A1
GeneCard	ODAD2
GeneCard	EFEMP2
GeneCard	IRF6
GeneCard	CEP78
GeneCard	DNMBP
GeneCard	TTBK2
GeneCard	HBEGF
GeneCard	SNRPN
GeneCard	CERT1
GeneCard	NYX
GeneCard	GET1
GeneCard	TRIM8
GeneCard	TNC
GeneCard	GRB2
GeneCard	ZNF365
GeneCard	DSC3
GeneCard	IGH
GeneCard	RPS26
GeneCard	DHDDS
GeneCard	MS4A4A
GeneCard	SLC25A20
GeneCard	NKX2-3
GeneCard	SGCG
GeneCard	BANK1
GeneCard	NT5E
GeneCard	PDCD10
GeneCard	RP9
GeneCard	MSX2
GeneCard	EEF2
GeneCard	THRA
GeneCard	SMARCE1
GeneCard	AKAP10
GeneCard	XYLT2
GeneCard	TJP1
GeneCard	OPRM1
GeneCard	POR
GeneCard	ZNF148
GeneCard	BDKRB2
GeneCard	SNRNP70
GeneCard	ATG5
GeneCard	YY1
GeneCard	PML
GeneCard	MIRLET7E
GeneCard	FAM161A
GeneCard	DDR2
GeneCard	SIX1
GeneCard	TMCO1

GeneCard	PC
GeneCard	UQCRFS1
GeneCard	MIR331
GeneCard	CISD2
GeneCard	GFPT1
GeneCard	HDAC5
GeneCard	RNASEL
GeneCard	CHST3
GeneCard	CRHR1
GeneCard	NEDD4L
GeneCard	RPSA
GeneCard	PHF8
GeneCard	P4HB
GeneCard	SRF
GeneCard	MCCC1
GeneCard	PSMA6
GeneCard	LCK
GeneCard	PDP1
GeneCard	PVT1
GeneCard	SLC9A3R1
GeneCard	HABP2
GeneCard	NR0B2
GeneCard	ECHS1
GeneCard	CLOCK
GeneCard	TRDN
GeneCard	AIMP2
GeneCard	MIR96
GeneCard	MLN
GeneCard	KCNE2
GeneCard	RORA
GeneCard	ADORA1
GeneCard	LCT
GeneCard	TRIP12
GeneCard	GLIS3
GeneCard	VPS33A
GeneCard	OTOF
GeneCard	ZFHX3
GeneCard	THBS4
GeneCard	ATP8A2
GeneCard	BIRC5
GeneCard	CTSH
GeneCard	MLYCD
GeneCard	IBSP
GeneCard	KRT83
GeneCard	CDK6
GeneCard	ATP5F1E
GeneCard	JAZF1
GeneCard	ATRIP-TREX1
GeneCard	CTSL
GeneCard	GOLM1
GeneCard	COL4A6
GeneCard	RPL35A
GeneCard	FERMT3
GeneCard	FANCA
GeneCard	MIR181B1
GeneCard	ACO2
GeneCard	HPGD
GeneCard	EYA4

GeneCard	ARL2BP
GeneCard	MIR103A1
GeneCard	TUBB6
GeneCard	PSMA5
GeneCard	MIR375
GeneCard	PIKFYVE
GeneCard	SEC24C
GeneCard	KIDINS220
GeneCard	ACSL4
GeneCard	AMPH
GeneCard	TOP1
GeneCard	GPX3
GeneCard	DGAT1
GeneCard	CHRNA1
GeneCard	MIPEP
GeneCard	APBA3
GeneCard	MIR106A
GeneCard	PPP2CA
GeneCard	CYP11B1
GeneCard	FDXR
GeneCard	WNT7A
GeneCard	MYORG
GeneCard	ARVCF
GeneCard	CYGB
GeneCard	PTPRJ
GeneCard	HHEX
GeneCard	SALL4
GeneCard	DDX41
GeneCard	COX8A
GeneCard	UQCRC2
GeneCard	MAT2A
GeneCard	TMEM230
GeneCard	CKM
GeneCard	NR2F2
GeneCard	DNAJB6
GeneCard	IFNAR2
GeneCard	PROCR
GeneCard	EIF4E
GeneCard	GTPBP1
GeneCard	ASL
GeneCard	STMN2
GeneCard	MIR26A1
GeneCard	CD247
GeneCard	PTGER4
GeneCard	FIP1L1
GeneCard	EPHB2
GeneCard	PTPRO
GeneCard	NGLY1
GeneCard	ATF2
GeneCard	SHC1
GeneCard	HSD11B1
GeneCard	PQBP1
GeneCard	IGHM
GeneCard	FABP3
GeneCard	CISH
GeneCard	MIR590
GeneCard	CYP2A6
GeneCard	NLGN4X

GeneCard	OAT
GeneCard	BCKDK
GeneCard	CUL3
GeneCard	SPART
GeneCard	MMADHC
GeneCard	APPL1
GeneCard	USF1
GeneCard	EXOSC9
GeneCard	ALDH7A1
GeneCard	PSMB4
GeneCard	MS4A1
GeneCard	UQCRB
GeneCard	PNP
GeneCard	PER3
GeneCard	GNAT2
GeneCard	SOX5
GeneCard	AMBP
GeneCard	MALT1
GeneCard	HRH1
GeneCard	CNNM2
GeneCard	PTK2B
GeneCard	SSB
GeneCard	MIR377
GeneCard	CAPN5
GeneCard	CD9
GeneCard	ITIH4
GeneCard	STAC3
GeneCard	ERVW-1
GeneCard	GABBR2
GeneCard	KLF4
GeneCard	PSMC4
GeneCard	SUMO1
GeneCard	DDB2
GeneCard	SHANK2
GeneCard	TRIO
GeneCard	MMAB
GeneCard	ATR
GeneCard	RPS24
GeneCard	PCBD1
GeneCard	F2RL1
GeneCard	NDUFB10
GeneCard	FSCN2
GeneCard	GTF2H5
GeneCard	WNT10B
GeneCard	HLA-DMA
GeneCard	MFN1
GeneCard	AURKA
GeneCard	ITGA6
GeneCard	QDPR
GeneCard	CARMIL2
GeneCard	SLC8A1
GeneCard	KRTCAP3
GeneCard	HIP1
GeneCard	PHF1
GeneCard	CLIP2
GeneCard	DHX16
GeneCard	KIF3B
GeneCard	KAT5

GeneCard	MIR101-1
GeneCard	SERPINA6
GeneCard	LAMA1
GeneCard	KRT9
GeneCard	RPS29
GeneCard	SIRT3
GeneCard	TLR8
GeneCard	BAK1
GeneCard	PDE6D
GeneCard	PDXK
GeneCard	RAD50
GeneCard	KLF6
GeneCard	MUC6
GeneCard	INPP5B
GeneCard	NRG3
GeneCard	ZFPM2
GeneCard	WAC
GeneCard	SYT2
GeneCard	NID1
GeneCard	CITED2
GeneCard	ACTL6B
GeneCard	BCL2L11
GeneCard	OXA1L
GeneCard	MRPS22
GeneCard	FAH
GeneCard	THAP1
GeneCard	FBXO38
GeneCard	MCL1
GeneCard	IL1RL1
GeneCard	BRAT1
GeneCard	MIR30D
GeneCard	CACNA1B
GeneCard	YWHAG
GeneCard	H3-3A
GeneCard	LDHB
GeneCard	MAP3K1
GeneCard	TRPC4AP
GeneCard	NDUFAF4
GeneCard	LMOD3
GeneCard	SLC25A12
GeneCard	CPT1A
GeneCard	PPARD
GeneCard	GMPPA
GeneCard	UGT1A9
GeneCard	RBFOX3
GeneCard	GPR37
GeneCard	KRT4
GeneCard	CXCL2
GeneCard	FBXW7
GeneCard	TRAF1
GeneCard	SIN3A
GeneCard	SPTB
GeneCard	ANXA2
GeneCard	CALCR
GeneCard	FANCM
GeneCard	CRLF2
GeneCard	MLC1
GeneCard	SLC1A1

GeneCard	HSPA2
GeneCard	BIRC3
GeneCard	FDX1
GeneCard	MLX
GeneCard	POLR2A
GeneCard	PYY
GeneCard	TBCD
GeneCard	ETFB
GeneCard	HLA-DQA2
GeneCard	MIR191
GeneCard	CELF1
GeneCard	ETHE1
GeneCard	STOX1
GeneCard	IL26
GeneCard	SPI1
GeneCard	MAP1LC3A
GeneCard	LIPG
GeneCard	TBX3
GeneCard	LACTB
GeneCard	PLS3
GeneCard	TPP2
GeneCard	MIR574
GeneCard	RFX5
GeneCard	TNFSF12
GeneCard	NOP10
GeneCard	UGT1A10
GeneCard	PPIG
GeneCard	PIGQ
GeneCard	CFLAR
GeneCard	RARS2
GeneCard	ATP2A3
GeneCard	GEMIN4
GeneCard	MADD
GeneCard	NBEA
GeneCard	FARS2
GeneCard	CCDC28B
GeneCard	ESCO2
GeneCard	SPHK2
GeneCard	ATP6V1A
GeneCard	HNRNPDL
GeneCard	NSUN2
GeneCard	PRKAA1
GeneCard	ELAVL1
GeneCard	HEY2
GeneCard	GEMIN2
GeneCard	LOXL1
GeneCard	TECTA
GeneCard	KRT74
GeneCard	ALOX12
GeneCard	LYZ
GeneCard	DAB1
GeneCard	NEDD4
GeneCard	UCP1
GeneCard	TIMMDC1
GeneCard	RPS17
GeneCard	MIR148B
GeneCard	CHD3
GeneCard	HMGA2

GeneCard	CFL1
GeneCard	WASHC4
GeneCard	ADAM9
GeneCard	HMCN1
GeneCard	HOXD10
GeneCard	KCNN3
GeneCard	GHRH
GeneCard	APLN
GeneCard	AXL
GeneCard	NKX2-6
GeneCard	PITX1
GeneCard	ANGPT1
GeneCard	SLC9A9
GeneCard	SLC5A5
GeneCard	RNLS
GeneCard	KDSR
GeneCard	CFAP43
GeneCard	ATP6AP2
GeneCard	PER2
GeneCard	PHGDH
GeneCard	PTK2
GeneCard	CEL
GeneCard	DMBT1
GeneCard	ADRA2A
GeneCard	MIR138-1
GeneCard	RIPK2
GeneCard	TBX22
GeneCard	ZMIZ1
GeneCard	GTPBP3
GeneCard	PNPO
GeneCard	CNTN2
GeneCard	HIVEP2
GeneCard	DEAF1
GeneCard	PGM3
GeneCard	CA12
GeneCard	DISC2
GeneCard	EEF1A1
GeneCard	PPP1R1B
GeneCard	ACKR2
GeneCard	HSD17B3
GeneCard	RBM8A
GeneCard	SDHAF2
GeneCard	PDGFA
GeneCard	MOBP
GeneCard	TACR1
GeneCard	ORAI1
GeneCard	TBX18
GeneCard	YME1L1
GeneCard	NEUROG3
GeneCard	RPL15
GeneCard	SYT11
GeneCard	TAT
GeneCard	SPRY2
GeneCard	GABRA2
GeneCard	TPPP3
GeneCard	ISL1
GeneCard	LHX1
GeneCard	OSM

GeneCard	WNT2B
GeneCard	RPS27
GeneCard	PDE6C
GeneCard	POLR3B
GeneCard	MS4A6E
GeneCard	HDC
GeneCard	MFF
GeneCard	FKBP5
GeneCard	C5AR1
GeneCard	FEN1
GeneCard	SIX5
GeneCard	DDHD2
GeneCard	MIR27B
GeneCard	HTR6
GeneCard	CRYGD
GeneCard	KRT86
GeneCard	STAG3
GeneCard	JAM2
GeneCard	PLD1
GeneCard	ALG2
GeneCard	MRAP
GeneCard	RPL10
GeneCard	ARHGEF2
GeneCard	PCSK1
GeneCard	AICDA
GeneCard	COL13A1
GeneCard	TGM3
GeneCard	HSPA1B
GeneCard	ACP1
GeneCard	MIR199B
GeneCard	GRIN3B
GeneCard	TBX2
GeneCard	RPS28
GeneCard	DEGS1
GeneCard	EIF4EBP1
GeneCard	NFATC1
GeneCard	CENPF
GeneCard	SYNE2
GeneCard	LOXHD1
GeneCard	ECE2
GeneCard	FBL
GeneCard	TALDO1
GeneCard	XRCC6
GeneCard	KDM1A
GeneCard	WNT2
GeneCard	SEPTIN5
GeneCard	XPR1
GeneCard	RELB
GeneCard	JMJD1C
GeneCard	RPL26
GeneCard	TNFRSF10B
GeneCard	GCKR
GeneCard	MS4A6A
GeneCard	LINGO1
GeneCard	EPHA3
GeneCard	DAXX
GeneCard	MGME1
GeneCard	XPC

GeneCard	UGT1A
GeneCard	GNPAT
GeneCard	BLOC1S6
GeneCard	SCD
GeneCard	ARMC9
GeneCard	FASN
GeneCard	MBNL1
GeneCard	PLEK
GeneCard	RXYLT1
GeneCard	CSF2RA
GeneCard	RSPH1
GeneCard	PRDX2
GeneCard	NT5C2
GeneCard	BCAP31
GeneCard	APCS
GeneCard	FIS1
GeneCard	ARID2
GeneCard	KCNJ8
GeneCard	FXYD2
GeneCard	NDUFC2-KCTD14
GeneCard	MT-TM
GeneCard	ARHGAP45
GeneCard	ACADS
GeneCard	MCPH1
GeneCard	COX7B
GeneCard	ARNTL
GeneCard	PUS3
GeneCard	TFPI
GeneCard	MIR134
GeneCard	ATP6V0A4
GeneCard	CD1A
GeneCard	SLAMF1
GeneCard	RPL27
GeneCard	RIC3
GeneCard	SUCLG1
GeneCard	MT-TG
GeneCard	MSN
GeneCard	CCL7
GeneCard	CDKN2C
GeneCard	CIZ1
GeneCard	SOX11
GeneCard	CLPB
GeneCard	TBL2
GeneCard	FGF9
GeneCard	CC2D1A
GeneCard	FMN2
GeneCard	MDH2
GeneCard	PPT2
GeneCard	SERPINB1
GeneCard	RPL7
GeneCard	CIT
GeneCard	CENPJ
GeneCard	PDSS2
GeneCard	RPS6
GeneCard	CACNA2D1
GeneCard	LMX1A
GeneCard	S100A7
GeneCard	RSPH3

GeneCard	NONO
GeneCard	TRAF2
GeneCard	SLC25A19
GeneCard	DVL3
GeneCard	VSX1
GeneCard	DISP1
GeneCard	PSMC3
GeneCard	UBE2D2
GeneCard	LGALS1
GeneCard	NHP2
GeneCard	DSC1
GeneCard	EIF3F
GeneCard	NME1
GeneCard	PRKCH
GeneCard	RPS20
GeneCard	RUBCN
GeneCard	HTR3A
GeneCard	GRID2
GeneCard	HBS1L
GeneCard	GPX4
GeneCard	CSMD1
GeneCard	HLCS
GeneCard	ORMDL3
GeneCard	PM20D1
GeneCard	KYNU
GeneCard	CKB
GeneCard	PRKCZ
GeneCard	CEP250
GeneCard	PSMA1
GeneCard	TOMM20
GeneCard	IGF2BP2
GeneCard	MARS2
GeneCard	TTC19
GeneCard	PRKRA
GeneCard	TRIP4
GeneCard	HELLS
GeneCard	PSMD12
GeneCard	BANF1
GeneCard	APBB3
GeneCard	NARS1
GeneCard	MIR361
GeneCard	TBXAS1
GeneCard	ADIPOR1
GeneCard	ACSF3
GeneCard	PRKAR1B
GeneCard	NES
GeneCard	RXRA
GeneCard	RARS1
GeneCard	ADAMTS1
GeneCard	SFPQ
GeneCard	PHOX2A
GeneCard	EPHB4
GeneCard	SERPINE2
GeneCard	TLR10
GeneCard	MT-TA
GeneCard	GATD1
GeneCard	GOLPH3
GeneCard	SLC29A1

GeneCard	PTCD3
GeneCard	HNRNPL
GeneCard	BST1
GeneCard	EEA1
GeneCard	PDPN
GeneCard	SLC35C1
GeneCard	MAN2A1
GeneCard	ADRA2B
GeneCard	ELAC2
GeneCard	DCHS1
GeneCard	TUBB4B
GeneCard	KLF11
GeneCard	MIR137
GeneCard	ALDH3A2
GeneCard	PPP1R3C
GeneCard	PTPRQ
GeneCard	PRKACG
GeneCard	MARCHF6
GeneCard	MYO9A
GeneCard	FGF14
GeneCard	AK2
GeneCard	PCK1
GeneCard	C9
GeneCard	PREP
GeneCard	VAMP1
GeneCard	GOT2
GeneCard	KY
GeneCard	ANKRD55
GeneCard	SAA2
GeneCard	RPS15
GeneCard	FKBP1A
GeneCard	PNKD
GeneCard	CLASP1
GeneCard	TUG1
GeneCard	TUBB1
GeneCard	CDKAL1
GeneCard	RNF19A
GeneCard	MTNR1B
GeneCard	NSD2
GeneCard	TRIP11
GeneCard	ATPAF2
GeneCard	RAB3A
GeneCard	PTS
GeneCard	ABO
GeneCard	GRIP1
GeneCard	FZD3
GeneCard	EEF1B2
GeneCard	CTNND1
GeneCard	WNT9B
GeneCard	E2F1
GeneCard	CHGB
GeneCard	CRKL
GeneCard	GPBAR1
GeneCard	RMDN3
GeneCard	FGF21
GeneCard	DERL1
GeneCard	HNRNPC
GeneCard	PTPN12

GeneCard	BAZ1B
GeneCard	TRAF3
GeneCard	RNPC3
GeneCard	ATP5PD
GeneCard	ELAVL4
GeneCard	PPL
GeneCard	MYOM2
GeneCard	AMBN
GeneCard	SULT1A3
GeneCard	KDELR2
GeneCard	GLRX
GeneCard	POU3F4
GeneCard	SGK1
GeneCard	UMPS
GeneCard	PPIB
GeneCard	TCN1
GeneCard	UQCRQ
GeneCard	HMOX2
GeneCard	ATP5PF
GeneCard	GAK
GeneCard	COASY
GeneCard	RPS15A
GeneCard	MAT1A
GeneCard	CCT5
GeneCard	AMPD3
GeneCard	MIR19B1
GeneCard	ADAMTS9
GeneCard	TRIM37
GeneCard	CHN1
GeneCard	TRAP1
GeneCard	INCA1
GeneCard	AP1S1
GeneCard	KLC2
GeneCard	CYC1
GeneCard	FXR1
GeneCard	GPC6
GeneCard	WWC1
GeneCard	DGCR8
GeneCard	MOCS2
GeneCard	DKK3
GeneCard	NDUFA4
GeneCard	SNAI1
GeneCard	FZD5
GeneCard	DDAH2
GeneCard	ITGA7
GeneCard	PUF60
GeneCard	KMO
GeneCard	TLL1
GeneCard	CEBPB
GeneCard	ANPEP
GeneCard	DPYSL5
GeneCard	FBLN1
GeneCard	ATP5PO
GeneCard	KLHL41
GeneCard	CTTN
GeneCard	PMEL
GeneCard	PIBF1
GeneCard	CCNA2

GeneCard	SNRPA
GeneCard	COLEC12
GeneCard	ALDH1A1
GeneCard	TAF6
GeneCard	PPIA
GeneCard	ATXN8
GeneCard	CDH11
GeneCard	PTBP1
GeneCard	MIR429
GeneCard	PEMT
GeneCard	FANCG
GeneCard	HSD3B2
GeneCard	RFC2
GeneCard	RAB28
GeneCard	PIK3CB
GeneCard	BAAT
GeneCard	PNLIP
GeneCard	FAM20C
GeneCard	MTMR10
GeneCard	CGB3
GeneCard	IMMT
GeneCard	GAS1
GeneCard	PSMD3
GeneCard	ZNF513
GeneCard	RPL35
GeneCard	ADAMTS5
GeneCard	UBE2D1
GeneCard	SLMAP
GeneCard	CLDN2
GeneCard	IFT57
GeneCard	ALOX15
GeneCard	KCNE5
GeneCard	HSD3B1
GeneCard	TFAP2B
GeneCard	TOP2A
GeneCard	CHRM2
GeneCard	TDP1
GeneCard	YWHAB
GeneCard	RBM10
GeneCard	BLVRB
GeneCard	SLC24A4
GeneCard	DDX39B
GeneCard	ANO3
GeneCard	COL27A1
GeneCard	ABCC4
GeneCard	KRT71
GeneCard	SV2A
GeneCard	CRYBA1
GeneCard	MIR184
GeneCard	RPS7
GeneCard	ATP1B1
GeneCard	SYNPO
GeneCard	ZKSCAN3
GeneCard	CDH13
GeneCard	HRH3
GeneCard	DLK1
GeneCard	KANK2
GeneCard	MAPK12

GeneCard	SOAT1
GeneCard	ZNF627
GeneCard	MTAP
GeneCard	SF3B1
GeneCard	SPINT2
GeneCard	CHRNA5
GeneCard	TACR3
GeneCard	CEP57
GeneCard	H2AX
GeneCard	RIN3
GeneCard	WNT7B
GeneCard	G3BP1
GeneCard	KDM5B
GeneCard	PLCD1
GeneCard	TNK1
GeneCard	LEPQTL1
GeneCard	MACF1
GeneCard	CTH
GeneCard	PPP1R3A
GeneCard	SH2B1
GeneCard	TPK1
GeneCard	COL4A2-AS2
GeneCard	ST3GAL3
GeneCard	CCKBR
GeneCard	TP53BP1
GeneCard	PGBD3
GeneCard	PRKAB1
GeneCard	CPOX
GeneCard	PRKDC
GeneCard	AREG
GeneCard	TIMM50
GeneCard	MIR186
GeneCard	ADIPOR2
GeneCard	SIK1
GeneCard	CCRL2
GeneCard	CD1C
GeneCard	PZP
GeneCard	SZT2
GeneCard	PLIN2
GeneCard	CFAP298
GeneCard	CEACAM16
GeneCard	ABHD11
GeneCard	CMA1
GeneCard	IDH3A
GeneCard	PARL
GeneCard	UBE2A
GeneCard	THRIL
GeneCard	TM2D3
GeneCard	MAK
GeneCard	ACY1
GeneCard	INPP5K
GeneCard	OGA
GeneCard	CNR2
GeneCard	FBP1
GeneCard	COG4
GeneCard	POU4F1
GeneCard	CHKB
GeneCard	RAN

GeneCard	EFNB1
GeneCard	SLC4A4
GeneCard	CD24
GeneCard	CRYGC
GeneCard	TWIST2
GeneCard	SNX27
GeneCard	PSMA7
GeneCard	IL27
GeneCard	ZC3H14
GeneCard	SH3GL2
GeneCard	MTMR14
GeneCard	NTN1
GeneCard	BLOC1S5
GeneCard	MSRB2
GeneCard	GHRHR
GeneCard	ARIH1
GeneCard	ALDH1A2
GeneCard	CUL1
GeneCard	ATG7
GeneCard	CGB5
GeneCard	DCHS2
GeneCard	HPSE2
GeneCard	SLC46A1
GeneCard	HSP90B1
GeneCard	RPLP0
GeneCard	DYRK1B
GeneCard	SAA4
GeneCard	LRP1B
GeneCard	SNRPD1
GeneCard	MIR198
GeneCard	GRIN3A
GeneCard	DLX3
GeneCard	MTPAP
GeneCard	HLA-DOA
GeneCard	TNFRSF6B
GeneCard	CLIP1
GeneCard	SH3KBP1
GeneCard	STK39
GeneCard	SLC39A8
GeneCard	KISS1
GeneCard	PYGB
GeneCard	FRG1
GeneCard	S100A1
GeneCard	GAS6
GeneCard	SPRED1
GeneCard	CPB2
GeneCard	GNAL
GeneCard	TRIM63
GeneCard	MACROD2
GeneCard	XRCC5
GeneCard	GOLGA2
GeneCard	ARHGAP9
GeneCard	NUP88
GeneCard	ABCC1
GeneCard	DNAJB1
GeneCard	MRPS16
GeneCard	SMNDC1
GeneCard	CHMP1A

GeneCard	CLDN3
GeneCard	COA5
GeneCard	MFGE8
GeneCard	TTBK1
GeneCard	LSM2
GeneCard	OPCML
GeneCard	PPP3CC
GeneCard	AGBL1
GeneCard	PCSK2
GeneCard	MIR20B
GeneCard	RAB10
GeneCard	ACAT1
GeneCard	CNTN5
GeneCard	RPL31
GeneCard	THY1
GeneCard	KIZ
GeneCard	FGF4
GeneCard	SCYL1
GeneCard	NEDD9
GeneCard	TRIM28
GeneCard	TBC1D8B
GeneCard	CD151
GeneCard	LBP
GeneCard	FAAH
GeneCard	RSPO1
GeneCard	CES1
GeneCard	CHKA
GeneCard	PRPF4
GeneCard	CDR1
GeneCard	TSG101
GeneCard	SLC39A13
GeneCard	IRS4
GeneCard	OMP
GeneCard	UBE2K
GeneCard	GLP1R
GeneCard	PI4KA
GeneCard	SLC25A24
GeneCard	FDFT1
GeneCard	CELF2
GeneCard	SPIB
GeneCard	PRKACB
GeneCard	EBP
GeneCard	STK24
GeneCard	AHSP
GeneCard	DLX5
GeneCard	RAP1A
GeneCard	TSHZ3
GeneCard	CSNK1E
GeneCard	PIGV
GeneCard	MYOG
GeneCard	IQGAP1
GeneCard	GBF1
GeneCard	SLAMF7
GeneCard	NDRG2
GeneCard	EGLN1
GeneCard	SKP1
GeneCard	LIPT1
GeneCard	DRAM2

GeneCard	GART
GeneCard	HBD
GeneCard	HTR4
GeneCard	PIGP
GeneCard	RHEB
GeneCard	CAV2
GeneCard	CCDC88C
GeneCard	MINPP1
GeneCard	ETS2
GeneCard	BICRA
GeneCard	BCAM
GeneCard	CORO1A
GeneCard	SIAH1
GeneCard	CCL21
GeneCard	FOXI1
GeneCard	PDE10A
GeneCard	TRAPPC2
GeneCard	ACYP1
GeneCard	PDGFRL
GeneCard	FAM126A
GeneCard	PIDD1
GeneCard	FHL2
GeneCard	HBZ
GeneCard	DPM1
GeneCard	PGAP3
GeneCard	DMXL2
GeneCard	KLHL3
GeneCard	SLC1A4
GeneCard	ABAT
GeneCard	SNRPB
GeneCard	CTHRC1
GeneCard	ITSN1
GeneCard	KRT6A
GeneCard	SERAC1
GeneCard	NTNG1
GeneCard	CD200
GeneCard	RAB29
GeneCard	TFE3
GeneCard	SNX3
GeneCard	DROSHA
GeneCard	PKM
GeneCard	ACTN3
GeneCard	PARD3
GeneCard	ASNS
GeneCard	IKBKE
GeneCard	NUDT1
GeneCard	FANCF
GeneCard	TPPP
GeneCard	RASGRP2
GeneCard	CCS
GeneCard	TTF2
GeneCard	APOBEC3G
GeneCard	CSNK2A2
GeneCard	IGHG1
GeneCard	APOM
GeneCard	FMO3
GeneCard	SLC5A2
GeneCard	CBY1

GeneCard	AP3D1
GeneCard	TAPBPL
GeneCard	RECK
GeneCard	UBE2N
GeneCard	KRT81
GeneCard	ROBO2
GeneCard	TOE1
GeneCard	HPSE
GeneCard	PSAT1
GeneCard	CEP55
GeneCard	SARS1
GeneCard	YWHAZ
GeneCard	CLDN1
GeneCard	NNMT
GeneCard	AUP1
GeneCard	SCAP
GeneCard	SDR9C7
GeneCard	PSMB1
GeneCard	RSRC1
GeneCard	CSK
GeneCard	ATCAY
GeneCard	CNTN1
GeneCard	SSTR2
GeneCard	POLR2L
GeneCard	FZD2
GeneCard	SLX4
GeneCard	MKRN3
GeneCard	ADRA1A
GeneCard	PRKD3
GeneCard	SEPTIN2
GeneCard	PPP2R2A
GeneCard	STAM
GeneCard	MID1
GeneCard	MXI1
GeneCard	CDH5
GeneCard	GABBR1
GeneCard	MRPL44
GeneCard	TNRC6A
GeneCard	CHERP
GeneCard	APOL4
GeneCard	ZFYVE27
GeneCard	NAT1
GeneCard	CDCA7
GeneCard	XPO1
GeneCard	AGPS
GeneCard	PRDX5
GeneCard	GORAB
GeneCard	DLC1
GeneCard	RING1
GeneCard	RO60
GeneCard	HECW2
GeneCard	SRSF1
GeneCard	TOR1AIP1
GeneCard	CTRL
GeneCard	SORD
GeneCard	GPI
GeneCard	MIR33B
GeneCard	ICAM3

GeneCard	HACD1
GeneCard	EIF2S3
GeneCard	PTPRG
GeneCard	LAD1
GeneCard	DGCR2
GeneCard	DGKQ
GeneCard	SLIT2
GeneCard	RAB6A
GeneCard	TMED10
GeneCard	HTR1B
GeneCard	PSMD9
GeneCard	TUBG1
GeneCard	PRSS3
GeneCard	PLCB3
GeneCard	DDIT4
GeneCard	MDH1
GeneCard	APC2
GeneCard	APOL2
GeneCard	CCND3
GeneCard	ASPH
GeneCard	SLC35A1
GeneCard	RTN2
GeneCard	UNC80
GeneCard	CRHBP
GeneCard	NOC3L
GeneCard	CYP51A1
GeneCard	SELPLG
GeneCard	MAP3K8
GeneCard	SLC5A1
GeneCard	VARS1
GeneCard	CPLX2
GeneCard	MYO3A
GeneCard	S100A6
GeneCard	ARTN
GeneCard	CAPZA2
GeneCard	GLO1
GeneCard	DDB1
GeneCard	ATXN2L
GeneCard	MICU1
GeneCard	MARCKS
GeneCard	GRM7
GeneCard	JAGN1
GeneCard	ZMYND11
GeneCard	LRRK1
GeneCard	ATOX1
GeneCard	SIPA1L2
GeneCard	RPS14
GeneCard	AHNAK
GeneCard	PSCA
GeneCard	VRK2
GeneCard	MAP2K4
GeneCard	MAVS
GeneCard	DM1-AS
GeneCard	DDX21
GeneCard	GRHL2
GeneCard	HGS
GeneCard	CACNG2
GeneCard	ADAM19

GeneCard	MASP1
GeneCard	MAD1L1
GeneCard	PSMA4
GeneCard	RNF31
GeneCard	CRBN
GeneCard	KCNC1
GeneCard	DEK
GeneCard	PRG2
GeneCard	PIGO
GeneCard	FADS2
GeneCard	KCNA3
GeneCard	COL14A1
GeneCard	ITGAV
GeneCard	UPF3B
GeneCard	YWHAH
GeneCard	ORC6
GeneCard	H2BC21
GeneCard	CDCA7L
GeneCard	RNF146
GeneCard	MIR129-1
GeneCard	FRAXA
GeneCard	MRPS23
GeneCard	LILRB1
GeneCard	SLC39A1
GeneCard	PVR
GeneCard	CIC
GeneCard	BCAR1
GeneCard	DUSP1
GeneCard	FCHO1
GeneCard	RPA1
GeneCard	HNRNPH1
GeneCard	MARK2
GeneCard	CRY2
GeneCard	FADS1
GeneCard	PRKAA2
GeneCard	CDC25C
GeneCard	SNX5
GeneCard	MIR152
GeneCard	S100A4
GeneCard	RIT2
GeneCard	BID
GeneCard	KREMEN1
GeneCard	CTNNA2
GeneCard	ENPP2
GeneCard	SIRT2
GeneCard	SERPIND1
GeneCard	KLK1
GeneCard	PUS1
GeneCard	TELO2
GeneCard	MIR30C1
GeneCard	ATP6AP1
GeneCard	SSR4
GeneCard	NEBL
GeneCard	CENPB
GeneCard	SSBP1
GeneCard	CD58
GeneCard	MLKL
GeneCard	PTGER2

GeneCard	MAP3K20
GeneCard	PSMB3
GeneCard	ADD3
GeneCard	TSPAN7
GeneCard	THOC6
GeneCard	NR1I3
GeneCard	MCM2
GeneCard	PRKCE
GeneCard	EPHA2
GeneCard	COX4I2
GeneCard	NEK10
GeneCard	LPO
GeneCard	NRIP1
GeneCard	HPX
GeneCard	BLNK
GeneCard	PAK1
GeneCard	POLR1D
GeneCard	CUL2
GeneCard	LIPF
GeneCard	MEI1
GeneCard	ACOD1
GeneCard	THOC2
GeneCard	SLC22A2
GeneCard	SLC30A6
GeneCard	HLA-DMB
GeneCard	MIR708
GeneCard	BMI1
GeneCard	NUP62
GeneCard	EML1
GeneCard	TBX6
GeneCard	PSMD2
GeneCard	EIF4H
GeneCard	PLCB2
GeneCard	TMEM175
GeneCard	CCL13
GeneCard	TSEN15
GeneCard	NDN
GeneCard	MPP2
GeneCard	RGS4
GeneCard	INPPL1
GeneCard	C8B
GeneCard	DAB2
GeneCard	ATP5MK
GeneCard	MRPS7
GeneCard	APEH
GeneCard	IGHG3
GeneCard	SIGLEC1
GeneCard	SLC25A6
GeneCard	SMPD2
GeneCard	SLC41A1
GeneCard	LUC7L2
GeneCard	FAR1
GeneCard	SLC6A9
GeneCard	SLC6A20
GeneCard	KRT2
GeneCard	RABGAP1L
GeneCard	EPB42
GeneCard	MEIS2

GeneCard	CCKAR
GeneCard	RSPO4
GeneCard	GATD3
GeneCard	CCR8
GeneCard	LALBA
GeneCard	CAD
GeneCard	PIGR
GeneCard	ZNF592
GeneCard	P2RY2
GeneCard	LEMD2
GeneCard	UBR1
GeneCard	SDC2
GeneCard	TCF3
GeneCard	MPDZ
GeneCard	SSTR3
GeneCard	STBD1
GeneCard	AP4E1
GeneCard	CLEC12A
GeneCard	MAP2K7
GeneCard	AP2A2
GeneCard	TDO2
GeneCard	HSF1
GeneCard	BMS1
GeneCard	PSMB5
GeneCard	TOLLIP
GeneCard	IL1R2
GeneCard	MT2A
GeneCard	COA6
GeneCard	LTC4S
GeneCard	LEF1
GeneCard	PAK2
GeneCard	CSTA
GeneCard	BDNF-AS
GeneCard	LYRM7
GeneCard	INPP5F
GeneCard	COQ6
GeneCard	PABPC1
GeneCard	PIK3R5
GeneCard	LHB
GeneCard	RUNX3
GeneCard	ADSS1
GeneCard	SUCLG2
GeneCard	RMC1
GeneCard	GYG2
GeneCard	SASH1
GeneCard	STT3A
GeneCard	GNRHR
GeneCard	CHD4
GeneCard	TMC6
GeneCard	POP1
GeneCard	QPCT
GeneCard	TBC1D20
GeneCard	MIR218-1
GeneCard	CDC25B
GeneCard	RFC1
GeneCard	ACMSD
GeneCard	CNNM4
GeneCard	UACA

GeneCard	SLITRK3
GeneCard	PSPN
GeneCard	TAGLN
GeneCard	PDHX
GeneCard	ROR2
GeneCard	MIR371A
GeneCard	GOT1
GeneCard	RDX
GeneCard	TAC3
GeneCard	POLH
GeneCard	RAB5B
GeneCard	IFT81
GeneCard	STIP1
GeneCard	DR1
GeneCard	TRAF3IP2-AS1
GeneCard	CYP8B1
GeneCard	DVL2
GeneCard	POLB
GeneCard	LGALS2
GeneCard	CHRDL1
GeneCard	H1-2
GeneCard	GABRG3
GeneCard	AATF
GeneCard	GJC3
GeneCard	PDPK1
GeneCard	TENM4
GeneCard	MS4
GeneCard	LPAR1
GeneCard	MRNIP
GeneCard	FERMT2
GeneCard	PCMT1
GeneCard	CELSR2
GeneCard	FUT8
GeneCard	FMNL2
GeneCard	NCR2
GeneCard	PI4K2A
GeneCard	PTGES
GeneCard	LIMS2
GeneCard	TUFM
GeneCard	MYLK2
GeneCard	PDLIM1
GeneCard	FAM107B
GeneCard	CCR5AS
GeneCard	RICTOR
GeneCard	PYCARD
GeneCard	GFRA2
GeneCard	MAP1LC3B
GeneCard	ERGIC1
GeneCard	SNX6
GeneCard	TNNC1
GeneCard	BLZF1
GeneCard	APLNR
GeneCard	DIAPH1
GeneCard	SPN
GeneCard	SYVN1
GeneCard	GTF2E2
GeneCard	FTCD
GeneCard	UNG

GeneCard	MICAL1
GeneCard	CYP2C18
GeneCard	NCOR2
GeneCard	SREK1IP1
GeneCard	FDX2
GeneCard	BCL3
GeneCard	MIR128-2
GeneCard	YDJC
GeneCard	SNRPD3
GeneCard	KIR3DL2
GeneCard	RGS6
GeneCard	LMAN1
GeneCard	HIKESHI
GeneCard	ICAM2
GeneCard	ABCC3
GeneCard	HDAC3
GeneCard	CNDP1
GeneCard	FCAR
GeneCard	BSN
GeneCard	SASS6
GeneCard	PSMC5
GeneCard	CWF19L1
GeneCard	FUZ
GeneCard	SRRT
GeneCard	RXRB
GeneCard	WNT11
GeneCard	CARTPT
GeneCard	TXN2
GeneCard	SERPINB2
GeneCard	C1QBP
GeneCard	RNF168
GeneCard	COMMD1
GeneCard	FNDC5
GeneCard	CASP4
GeneCard	HLA-DRB3
GeneCard	NCAPH2
GeneCard	PTF1A
GeneCard	PSMD14
GeneCard	H4-16
GeneCard	TAF8
GeneCard	DDRGK1
GeneCard	CHEK1
GeneCard	PENK
GeneCard	HS3ST1
GeneCard	PPA2
GeneCard	SCZD10
GeneCard	WDR11
GeneCard	CYP2C8
GeneCard	HAS2
GeneCard	NEDD8
GeneCard	H6PD
GeneCard	LARS1
GeneCard	AAK1
GeneCard	SRD5A1
GeneCard	SCZD6
GeneCard	CCDC22
GeneCard	TREML2
GeneCard	PLIN1

GeneCard	IL24
GeneCard	MUC3A
GeneCard	RAPGEF3
GeneCard	LRP12
GeneCard	REG3A
GeneCard	MS4A4E
GeneCard	RGS5
GeneCard	TPT1
GeneCard	RPL28
GeneCard	VPS29
GeneCard	ATP6V1B2
GeneCard	SCZD2
GeneCard	KLK15
GeneCard	GADD45A
GeneCard	SACM1L
GeneCard	TAOK1
GeneCard	ADH1A
GeneCard	RPL13
GeneCard	MAPKAPK2
GeneCard	SYNJ2
GeneCard	CNTN4
GeneCard	NCS1
GeneCard	SEPTIN4
GeneCard	GDAP1L1
GeneCard	RGS1
GeneCard	TRIM25
GeneCard	OBSCN
GeneCard	ZHX2
GeneCard	WARS2
GeneCard	CRIPT
GeneCard	KCND2
GeneCard	NAB2
GeneCard	UBE2T
GeneCard	SELENOP
GeneCard	KIF12
GeneCard	SCZD8
GeneCard	SCZD1
GeneCard	CDK5RAP1
GeneCard	SIM1
GeneCard	HCK
GeneCard	GSTA4
GeneCard	SCZD7
GeneCard	FST
GeneCard	KLF5
GeneCard	COG8
GeneCard	CPN1
GeneCard	RBM28
GeneCard	DOCK2
GeneCard	CHRFAM7A
GeneCard	IGFBP7
GeneCard	DLL3
GeneCard	PANK1
GeneCard	TNK2
GeneCard	AP5Z1
GeneCard	SRSF6
GeneCard	CPA6
GeneCard	ARRB2
GeneCard	RABEP1

GeneCard	YBX3
GeneCard	DPP10
GeneCard	MIR9-3
GeneCard	CDK9
GeneCard	MT-TY
GeneCard	HOXB7
GeneCard	CCT7
GeneCard	AP2B1
GeneCard	MAPRE2
GeneCard	NR4A1
GeneCard	RBX1
GeneCard	ALDH4A1
GeneCard	IFNL3
GeneCard	MSL3
GeneCard	LRP10
GeneCard	LFNG
GeneCard	MIR323A
GeneCard	SCZD11
GeneCard	UPF1
GeneCard	CYP2B6
GeneCard	CYP2J2
GeneCard	PSMD7
GeneCard	ROGDI
GeneCard	CTSS
GeneCard	KCNT2
GeneCard	EMG1
GeneCard	TERF1
GeneCard	NRON
GeneCard	CGB7
GeneCard	DOP1B
GeneCard	PSMB2
GeneCard	RPS18
GeneCard	GRM2
GeneCard	ZSWIM6
GeneCard	B4GALT1
GeneCard	CCDC107
GeneCard	TARS1
GeneCard	UBE2E3
GeneCard	TCF21
GeneCard	TMPRSS2
GeneCard	BRD2
GeneCard	KHDRBS1
GeneCard	LCN1
GeneCard	NFAT5
GeneCard	EN2
GeneCard	SLC51A
GeneCard	SCZD3
GeneCard	SCZD12
GeneCard	CD63
GeneCard	RBMX
GeneCard	PTPN3
GeneCard	CAMK4
GeneCard	S1PR1
GeneCard	UBE4B
GeneCard	THSD7A
GeneCard	DGCR5
GeneCard	GABRB1
GeneCard	FKBP6

GeneCard	OPRL1
GeneCard	DNAJC19
GeneCard	PDE6H
GeneCard	SLC1A7
GeneCard	ARAF
GeneCard	PGAM1
GeneCard	IARS1
GeneCard	PPP2R5A
GeneCard	TNKS
GeneCard	AFF1
GeneCard	DECR1
GeneCard	USP40
GeneCard	HEYL
GeneCard	LTBR
GeneCard	TMEFF2
GeneCard	HIP1R
GeneCard	USP24
GeneCard	SLC5A3
GeneCard	SCZD13
GeneCard	SCZD14
GeneCard	DCAF7
GeneCard	PTGDR
GeneCard	EFHC1
GeneCard	KCNJ3
GeneCard	HAPLN1
GeneCard	SLC4A2
GeneCard	MPI
GeneCard	RPS23
GeneCard	PI4KB
GeneCard	LIN28A
GeneCard	PIGG
GeneCard	PCSK6
GeneCard	SPRY4-IT1
GeneCard	ABCA2
GeneCard	FGF16
GeneCard	PSMB10
GeneCard	VGF
GeneCard	KIF5C
GeneCard	MGLL
GeneCard	KRT6B
GeneCard	PXN
GeneCard	SH3GL1
GeneCard	KIF2A
GeneCard	ROCK1
GeneCard	MAD2L2
GeneCard	C3AR1
GeneCard	RPN2
GeneCard	PSMA2
GeneCard	SNRPD2
GeneCard	NTM
GeneCard	PCGF2
GeneCard	PSMA3
GeneCard	MAP2K6
GeneCard	OVOL2
GeneCard	ACBD5
GeneCard	KCTD17
GeneCard	PPP1R13L
GeneCard	TUBGCP6

GeneCard	PDE4B
GeneCard	MS4A2
GeneCard	VPS37A
GeneCard	PPP3CB
GeneCard	KLF2
GeneCard	ID2
GeneCard	TPM4
GeneCard	PSMD1
GeneCard	CBR1
GeneCard	HCRTR2
GeneCard	EOGT
GeneCard	SRR
GeneCard	GABARAPL1
GeneCard	NFIC
GeneCard	GRM8
GeneCard	HSPBP1
GeneCard	GNAI3
GeneCard	SLC31A1
GeneCard	NCOA4
GeneCard	LIN28B
GeneCard	RNF6
GeneCard	OPRK1
GeneCard	NAA15
GeneCard	IGFBP5
GeneCard	RCVRN
GeneCard	SNX1
GeneCard	RPLP2
GeneCard	NGF-AS1
GeneCard	TIPRL
GeneCard	FANCD2OS
GeneCard	RPS11
GeneCard	ADH5
GeneCard	RPS6KA1
GeneCard	ST8SIA4
GeneCard	PGAM5
GeneCard	SEC23A
GeneCard	CD320
GeneCard	TNIK
GeneCard	RNF11
GeneCard	GSDMB
GeneCard	CDH4
GeneCard	LARP7
GeneCard	ATIC
GeneCard	ATP13A3
GeneCard	HOMER2
GeneCard	WNT16
GeneCard	BAG2
GeneCard	ST14
GeneCard	KCNIP2
GeneCard	C20orf203
GeneCard	GNB2
GeneCard	RILP
GeneCard	NUDT15
GeneCard	UBE2E2
GeneCard	PTPN6
GeneCard	PROK2
GeneCard	DNAJC12
GeneCard	SEPTIN1

GeneCard	ZCWPW1
GeneCard	LYN
GeneCard	S1PR2
GeneCard	PACSIN3
GeneCard	KLRC1
GeneCard	CAPNS1
GeneCard	RPL19
GeneCard	CASP12
GeneCard	LAP3
GeneCard	PCSK5
GeneCard	ABCD2
GeneCard	RPL3L
GeneCard	SMARCD1
GeneCard	ELMO1
GeneCard	NUDT6
GeneCard	EGR3
GeneCard	AGO2
GeneCard	NCOR1
GeneCard	CD99
GeneCard	COA7
GeneCard	ABI3
GeneCard	ERCC6L2
GeneCard	ALPK3
GeneCard	CRK
GeneCard	SCG2
GeneCard	LTBP1
GeneCard	PAGR1
GeneCard	GTDC1
GeneCard	NDUFAB1
GeneCard	MT1B
GeneCard	SRP19
GeneCard	MCC
GeneCard	PRICKLE2
GeneCard	CCNT1
GeneCard	HCCS
GeneCard	RPN1
GeneCard	TFF2
GeneCard	DENR
GeneCard	NCKIPSD
GeneCard	NPTX2
GeneCard	MRPL23
GeneCard	HERPUD1
GeneCard	FGF13
GeneCard	ARCN1
GeneCard	ANKS1B
GeneCard	ANGPTL4
GeneCard	AZU1
GeneCard	SAR1A
GeneCard	HELT
GeneCard	MCTP2
GeneCard	GNB5
GeneCard	NPAS2
GeneCard	NAXE
GeneCard	CSPG4
GeneCard	FAF1
GeneCard	KCNAB2
GeneCard	TBATA
GeneCard	CTF1

GeneCard	ELOB
GeneCard	SHROOM3
GeneCard	KIR2DL3
GeneCard	WIPI1
GeneCard	NDEL1
GeneCard	CHL1
GeneCard	BAMBI
GeneCard	IL3RA
GeneCard	REEP2
GeneCard	COBL
GeneCard	EN1
GeneCard	SNRPE
GeneCard	EXOSC10
GeneCard	PDCD4
GeneCard	HRG
GeneCard	PPP6C
GeneCard	LNPEP
GeneCard	RBP1
GeneCard	MAP3K13
GeneCard	IL15RA
GeneCard	KLK11
GeneCard	SULT2A1
GeneCard	HMGN1
GeneCard	PLA2G10
GeneCard	CLDN11
GeneCard	SFXN4
GeneCard	ARNT
GeneCard	CCDC62
GeneCard	IL6-AS1
GeneCard	CTBP2
GeneCard	CREM
GeneCard	CRABP1
GeneCard	PSIP1
GeneCard	RER1
GeneCard	TERF2
GeneCard	HOXA1
GeneCard	NR1D1
GeneCard	RHBDF2
GeneCard	MINK1
GeneCard	CILK1
GeneCard	MTDH
GeneCard	STRADA
GeneCard	CRLF1
GeneCard	IL17RD
GeneCard	DNAJC30
GeneCard	PSMD11
GeneCard	NAV2
GeneCard	PRDM5
GeneCard	RNASE2
GeneCard	SMARCD2
GeneCard	RPS10-NUDT3
GeneCard	OXTR
GeneCard	RAD54B
GeneCard	ANXA6
GeneCard	GRM3
GeneCard	MFHAS1
GeneCard	RPL21
GeneCard	DDAH1

GeneCard	ATG101
GeneCard	CD47
GeneCard	DUSP6
GeneCard	CA9
GeneCard	NAV1
GeneCard	UBE2S
GeneCard	PDE9A
GeneCard	HLA-DOB
GeneCard	ACP2
GeneCard	SLC27A4
GeneCard	AKR1A1
GeneCard	FUBP1
GeneCard	CCHCR1
GeneCard	SELENBP1
GeneCard	AK1
GeneCard	NDUFA8
GeneCard	PLK1
GeneCard	SNX10
GeneCard	RAE1
GeneCard	PIP5K1C
GeneCard	TECR
GeneCard	SLIT1
GeneCard	TBL3
GeneCard	AOC1
GeneCard	TGM5
GeneCard	IGLON5
GeneCard	DNM3
GeneCard	CYBRD1
GeneCard	SLC5A6
GeneCard	NCAN
GeneCard	SNHG1
GeneCard	TST
GeneCard	TUBA8
GeneCard	ST13
GeneCard	ATP6V0C
GeneCard	CRTC1
GeneCard	GNLY
GeneCard	ADAMTS20
GeneCard	ZNF23
GeneCard	PIK3R3
GeneCard	SEM1
GeneCard	EXOC4
GeneCard	IGSF6
GeneCard	TCFL5
GeneCard	FYCO1
GeneCard	EMP3
GeneCard	AP1B1
GeneCard	PNOC
GeneCard	LRMDA
GeneCard	ROBO3
GeneCard	CXADR
GeneCard	PFKFB3
GeneCard	MIR136
GeneCard	KIAA0319
GeneCard	ALAS1
GeneCard	POLRMT
GeneCard	MALRD1
GeneCard	CCDC50

GeneCard	ICMT
GeneCard	ZSCAN21
GeneCard	MESD
GeneCard	RPL7A
GeneCard	WASL
GeneCard	RAB40AL
GeneCard	MIR219A1
GeneCard	MLST8
GeneCard	LY9
GeneCard	CCL8
GeneCard	H4C13
GeneCard	PSMG1
GeneCard	SYNC
GeneCard	SLC18A1
GeneCard	TCEA2
GeneCard	GPT2
GeneCard	PHIP
GeneCard	AZGP1
GeneCard	MIR16-2
GeneCard	NSF
GeneCard	PPP5C
GeneCard	HEY1
GeneCard	SH3PXD2A
GeneCard	CRELD1
GeneCard	NUDC
GeneCard	PRKCI
GeneCard	TBCEL-TECTA
GeneCard	DNASE1L1
GeneCard	PFKP
GeneCard	TET3
GeneCard	NLGN1
GeneCard	FAM167A
GeneCard	MIR497
GeneCard	VPS41
GeneCard	RAB1B
GeneCard	SRSF3
GeneCard	C12orf4
GeneCard	UBA52
GeneCard	NISCH
GeneCard	CCL24
GeneCard	ARNT2
GeneCard	MIR346
GeneCard	DAB2IP
GeneCard	CCDC6
GeneCard	LTA4H
GeneCard	NSMCE2
GeneCard	DLGAP2
GeneCard	VPS53
GeneCard	RDH11
GeneCard	SPATA5L1
GeneCard	CDC5L
GeneCard	TXNL4A
GeneCard	HVCN1
GeneCard	ATG12
GeneCard	STK36
GeneCard	PYROXD1
GeneCard	SLC25A5
GeneCard	NDUFA5

GeneCard	KRT75
GeneCard	MRPL3
GeneCard	HOXB13
GeneCard	SP4
GeneCard	NAT8L
GeneCard	PCK2
GeneCard	PIH1D2
GeneCard	CENPA
GeneCard	ADPRS
GeneCard	EPB41L1
GeneCard	CLEC4E
GeneCard	RPS9
GeneCard	HACE1
GeneCard	MELTF
GeneCard	MAPT-AS1
GeneCard	KLK7
GeneCard	SLC44A1
GeneCard	HCP5
GeneCard	DLG2
GeneCard	RBM4
GeneCard	ADCY8
GeneCard	ABT1
GeneCard	RCBTB1
GeneCard	MPZL1
GeneCard	SPON1
GeneCard	PPME1
GeneCard	NEGR1
GeneCard	MIR26A2
GeneCard	SVIL
GeneCard	WDR41
GeneCard	SDC3
GeneCard	PBRM1
GeneCard	DDX1
GeneCard	KPNA2
GeneCard	PFAS
GeneCard	CBLB
GeneCard	TFB1M
GeneCard	TGDS
GeneCard	PAPSS2
GeneCard	FCHSD2
GeneCard	CAMK1D
GeneCard	ADCY1
GeneCard	SKAP2
GeneCard	CIRBP
GeneCard	DIABLO
GeneCard	GLRX5
GeneCard	TNR
GeneCard	ARR3
GeneCard	ILK
GeneCard	RPS25
GeneCard	RPS12
GeneCard	PTN
GeneCard	EFNB2
GeneCard	PSMD8
GeneCard	IQSEC1
GeneCard	HSPA6
GeneCard	SENP7
GeneCard	PFN2

GeneCard	IPO8
GeneCard	UBXN6
GeneCard	TIMM13
GeneCard	SLC30A4
GeneCard	GOLGA4
GeneCard	RPLP1
GeneCard	MIR381
GeneCard	PRKAR2B
GeneCard	NXNL1
GeneCard	TBXA2R
GeneCard	CDC45
GeneCard	RHOD
GeneCard	EEF1D
GeneCard	RHOT1
GeneCard	TYRO3
GeneCard	COQ7
GeneCard	RPS3
GeneCard	OPRD1
GeneCard	DDR1
GeneCard	PASK
GeneCard	SNRPC
GeneCard	ST2
GeneCard	VAPA
GeneCard	RAD23A
GeneCard	POU2F1
GeneCard	PRDX6
GeneCard	DDX6
GeneCard	RPL13A
GeneCard	TMPRSS15
GeneCard	MIR124-3
GeneCard	MCM7
GeneCard	SMAD1
GeneCard	SHMT2
GeneCard	PCA3
GeneCard	RALA
GeneCard	CAPN8
GeneCard	EED
GeneCard	VIPR1
GeneCard	ESRRB
GeneCard	PER1
GeneCard	ELOC
GeneCard	BIRC2
GeneCard	PFKL
GeneCard	LGMN
GeneCard	IGFBP4
GeneCard	SPEG
GeneCard	METTL27
GeneCard	FKBP4
GeneCard	TEX14
GeneCard	ENTR1
GeneCard	CEMIP2
GeneCard	ADGRA3
GeneCard	PPHLN1
GeneCard	FOSL1
GeneCard	IL1RAP
GeneCard	CYFIP1
GeneCard	ISCA2
GeneCard	THSD4

GeneCard	AMD1
GeneCard	PDLIM5
GeneCard	COPB2
GeneCard	PRRC2A
GeneCard	LUM
GeneCard	LETM1
GeneCard	NELFA
GeneCard	FTMT
GeneCard	LNX1
GeneCard	MEOX1
GeneCard	MPP7
GeneCard	APPBP2
GeneCard	ADAMTS14
GeneCard	CCNC
GeneCard	ISYNA1
GeneCard	PDZD9
GeneCard	TRAIP
GeneCard	ABCA5
GeneCard	SGMS1
GeneCard	RPS2
GeneCard	AGR2
GeneCard	MIR339
GeneCard	POLR2E
GeneCard	WNT5B
GeneCard	EOMES
GeneCard	FZD1
GeneCard	SOCS2
GeneCard	OXCT1
GeneCard	EVPL
GeneCard	HOMER1
GeneCard	YBX1
GeneCard	PPP1R8
GeneCard	SLC7A5
GeneCard	PPP2R1B
GeneCard	FZD8
GeneCard	MUC12
GeneCard	TRPC4
GeneCard	HHAT
GeneCard	QKI
GeneCard	CDR2
GeneCard	ITGA1
GeneCard	DLGAP1
GeneCard	ELAVL3
GeneCard	IRF2
GeneCard	ELAVL2
GeneCard	BCL2A1
GeneCard	MIEF2
GeneCard	NCL
GeneCard	PPP1R15A
GeneCard	ICAM5
GeneCard	CASZ1
GeneCard	USP6
GeneCard	ACP3
GeneCard	STMN1
GeneCard	IMMP2L
GeneCard	PGC
GeneCard	TAF13
GeneCard	TUBA1C

GeneCard	DPEP1
GeneCard	C4BPA
GeneCard	HMGCS2
GeneCard	PSRC1
GeneCard	CRYBB3
GeneCard	BTRC
GeneCard	INAFM2
GeneCard	HEPH
GeneCard	KATNB1
GeneCard	GNA15
GeneCard	IMPA1
GeneCard	MYOZ2
GeneCard	MAST1
GeneCard	CARS1
GeneCard	MRPS2
GeneCard	RCOR1
GeneCard	KCNA4
GeneCard	RPL10A
GeneCard	BEX3
GeneCard	FAT3
GeneCard	RAB6B
GeneCard	RPS27L
GeneCard	SNIP1
GeneCard	PTGER3
GeneCard	CLASRP
GeneCard	FGF12
GeneCard	GHITM
GeneCard	ZNF224
GeneCard	NUP37
GeneCard	ARRB1
GeneCard	ANKRD50
GeneCard	TUBA3C
GeneCard	PPP1R9B
GeneCard	CALML5
GeneCard	WWP2
GeneCard	BRWD1
GeneCard	SPHK1
GeneCard	DHRS2
GeneCard	DGCR6
GeneCard	ETV5
GeneCard	PSMF1
GeneCard	PRDM2
GeneCard	FNIP1
GeneCard	GPC5
GeneCard	SLC22A6
GeneCard	FAM47E
GeneCard	GNAI1
GeneCard	ZRSR2
GeneCard	SSX2
GeneCard	CYP26A1
GeneCard	RAB8B
GeneCard	UBE3C
GeneCard	POLR3K
GeneCard	TNKS2
GeneCard	OTOGL
GeneCard	YLPM1
GeneCard	AKAP13
GeneCard	ADRA2C

GeneCard	BAIAP2
GeneCard	CNOT3
GeneCard	ESM1
GeneCard	MIR582
GeneCard	RBM17
GeneCard	RFWD3
GeneCard	WNT9A
GeneCard	SLC30A3
GeneCard	APOC4
GeneCard	ACADSB
GeneCard	LAMC1
GeneCard	RPTOR
GeneCard	WEE1
GeneCard	PLXNB2
GeneCard	GPX7
GeneCard	NEPRO
GeneCard	ST6GAL1
GeneCard	RGS2
GeneCard	LPIN1
GeneCard	MIR181B2
GeneCard	AP2A1
GeneCard	FIBP
GeneCard	HIF1AN
GeneCard	CYSLTR2
GeneCard	GUCY1B1
GeneCard	AS3MT
GeneCard	ZBTB16
GeneCard	MAPK11
GeneCard	RAB1A
GeneCard	PKNOX1
GeneCard	AMFR
GeneCard	CHRM4
GeneCard	GRK5
GeneCard	CHRNB4
GeneCard	COX5B
GeneCard	SLC3A2
GeneCard	MGRN1
GeneCard	SIRT6
GeneCard	FABP5
GeneCard	AURKB
GeneCard	PAK4
GeneCard	SLC13A3
GeneCard	RAPGEF4
GeneCard	COPS5
GeneCard	MNDA
GeneCard	LSM4
GeneCard	ZNF804A
GeneCard	DYNLL1
GeneCard	FBXO48
GeneCard	VTI1B
GeneCard	VWA7
GeneCard	ATP8A1
GeneCard	ROCK2
GeneCard	KPNB1
GeneCard	ZFYVE1
GeneCard	STK32B
GeneCard	PMVK
GeneCard	TRAPPC10

GeneCard	USF2
GeneCard	GPHA2
GeneCard	MBTPS1
GeneCard	CAMKK2
GeneCard	AEBP1
GeneCard	PRPS2
GeneCard	AKR1C4
GeneCard	TRIM31
GeneCard	MUCL3
GeneCard	TRAM2
GeneCard	ATP2B1
GeneCard	VIPR2
GeneCard	RPL6
GeneCard	AHSA1
GeneCard	RPL38
GeneCard	TAF1L
GeneCard	MIR4709
GeneCard	ST3GAL4
GeneCard	EXTL3
GeneCard	SUPT3H
GeneCard	SYMPK
GeneCard	TLN1
GeneCard	BCAT1
GeneCard	SLC25A28
GeneCard	MACROH2A1
GeneCard	P3H3
GeneCard	WNT6
GeneCard	STC1
GeneCard	SEPTIN3
GeneCard	CPT1C
GeneCard	CTSE
GeneCard	SLC25A11
GeneCard	DHX9
GeneCard	BCAS3
GeneCard	HRH4
GeneCard	SUPT4H1
GeneCard	H4C9
GeneCard	TNFRSF14
GeneCard	MEIS1
GeneCard	H4C8
GeneCard	AZIN2
GeneCard	CNTFR
GeneCard	NCLN
GeneCard	ALDH9A1
GeneCard	SCARF2
GeneCard	NR2C2
GeneCard	KEL
GeneCard	KLC4
GeneCard	PDS5B
GeneCard	ARHGAP1
GeneCard	ANKRD30A
GeneCard	ALCAM
GeneCard	SLC44A5
GeneCard	VPS4B
GeneCard	GALNT2
GeneCard	SEPTIN7
GeneCard	SRM
GeneCard	MIR671

GeneCard	EXOC1
GeneCard	TXNRD1
GeneCard	H4C4
GeneCard	SP6
GeneCard	PRSS12
GeneCard	PDK2
GeneCard	NECAP1
GeneCard	FARP1
GeneCard	RAB11FIP2
GeneCard	HNRNPA3
GeneCard	CEBPD
GeneCard	H4C1
GeneCard	FOSB
GeneCard	PSMA8
GeneCard	APBB1IP
GeneCard	DEFA6
GeneCard	RTN1
GeneCard	AGFG1
GeneCard	SOAT2
GeneCard	KIF4A
GeneCard	DDX54
GeneCard	ADPRH
GeneCard	SLC17A1
GeneCard	MIR95
GeneCard	MAPKAPK3
GeneCard	NKIRAS1
GeneCard	POTEF
GeneCard	RACK1
GeneCard	TNFSF14
GeneCard	TIMM29
GeneCard	ABCG4
GeneCard	ADORA2B
GeneCard	RPS3A
GeneCard	GNPDA2
GeneCard	HNRNPF
GeneCard	CC2D2B
GeneCard	TCHH
GeneCard	PILRA
GeneCard	TARS2
GeneCard	CLMN
GeneCard	VASP
GeneCard	KCTD13
GeneCard	KIR2DL2
GeneCard	TXNL1
GeneCard	PPP1R17
GeneCard	MCM3
GeneCard	CADPS2
GeneCard	H4C11
GeneCard	KLC3
GeneCard	CA1
GeneCard	BMPER
GeneCard	NDUFS5
GeneCard	SEC24B
GeneCard	UBE2G2
GeneCard	KDELR1
GeneCard	CDCP2
GeneCard	EFNA5
GeneCard	CTBS

GeneCard	RPS5
GeneCard	PLA2G1B
GeneCard	FBXO40
GeneCard	GCN1
GeneCard	SECISBP2
GeneCard	FAM30A
GeneCard	RPS13
GeneCard	DDX17
GeneCard	CD82
GeneCard	RPL3
GeneCard	LACRT
GeneCard	CDC7
GeneCard	NDUFB6
GeneCard	CTSZ
GeneCard	IL32
GeneCard	NLRP5
GeneCard	PRSS2
GeneCard	CCNY
GeneCard	RAI2
GeneCard	ACAD8
GeneCard	SUPV3L1
GeneCard	MTRNR2L12
GeneCard	PPIL2
GeneCard	IFI16
GeneCard	DOCK1
GeneCard	FAU
GeneCard	RBM25
GeneCard	MFAP2
GeneCard	SLC25A26
GeneCard	MED15
GeneCard	CDR1-AS
GeneCard	AADAT
GeneCard	ARHGAP24
GeneCard	COX6C
GeneCard	HTR3B
GeneCard	PHF23
GeneCard	RPL23
GeneCard	GGPS1
GeneCard	TACR2
GeneCard	SUPT5H
GeneCard	PDCD1LG2
GeneCard	ATP8B4
GeneCard	CASS4
GeneCard	DPY19L2P2
GeneCard	HNRNPM
GeneCard	CAPZB
GeneCard	CPE
GeneCard	PERP
GeneCard	SLC25A3
GeneCard	VPS26B
GeneCard	PYCR2
GeneCard	HOOK1
GeneCard	SLC30A7
GeneCard	PTPRD
GeneCard	G6PC2
GeneCard	MSI1
GeneCard	MSRB1
GeneCard	COPB1

GeneCard	EIF5A
GeneCard	RPL27A
GeneCard	RAD23B
GeneCard	GGA3
GeneCard	PLAGL1
GeneCard	UBXN4
GeneCard	CHMP4B
GeneCard	ZDHHC17
GeneCard	MAP2K3
GeneCard	SLC30A1
GeneCard	MLLT1
GeneCard	KDM3B
GeneCard	CDK5RAP3
GeneCard	SYNCRIP
GeneCard	TRA2B
GeneCard	OST4
GeneCard	NCAPD2
GeneCard	FABP7
GeneCard	G3BP2
GeneCard	PTPN13
GeneCard	MPIG6B
GeneCard	ABI1
GeneCard	EPS15
GeneCard	CHP1
GeneCard	IWS1
GeneCard	NELFCD
GeneCard	SEL1L
GeneCard	NRF1
GeneCard	DSCAML1
GeneCard	RPL30
GeneCard	ARG2
GeneCard	PTRHD1
GeneCard	EPPK1
GeneCard	THBS3
GeneCard	WASHC1
GeneCard	CAMK2D
GeneCard	PPP2R3A
GeneCard	URB2
GeneCard	ESS2
GeneCard	ST8SIA2
GeneCard	BRSK2
GeneCard	RAB12
GeneCard	LOC111258525
GeneCard	MAP2K5
GeneCard	CLP1
GeneCard	ERAL1
GeneCard	TOP2B
GeneCard	ARL2
GeneCard	TXNIP
GeneCard	CTPS1
GeneCard	GKN1
GeneCard	LAMC3
GeneCard	MRPL58
GeneCard	FOXA2
GeneCard	DFFA
GeneCard	CFDP1
GeneCard	SSX1
GeneCard	ADAM8

GeneCard	ZFR
GeneCard	CD5L
GeneCard	VTA1
GeneCard	NMNAT2
GeneCard	HMHB1
GeneCard	FUT1
GeneCard	USP16
GeneCard	GNA14
GeneCard	PROKR1
GeneCard	NSDHL
GeneCard	MT-TR
GeneCard	FLRT1
GeneCard	TRAPPC14
GeneCard	DNAH6
GeneCard	PSMB7
GeneCard	MAPK7
GeneCard	TDP2
GeneCard	FRMD6
GeneCard	TTC3
GeneCard	MROH8
GeneCard	AKT1S1
GeneCard	PITPNA
GeneCard	PICK1
GeneCard	MASTL
GeneCard	SMAD5
GeneCard	FGR
GeneCard	CREB3L4
GeneCard	COG1
GeneCard	PTCD1
GeneCard	CDC42EP3
GeneCard	MEST
GeneCard	HSP90AB1
GeneCard	RNF186
GeneCard	JAG2
GeneCard	WNT8A
GeneCard	FER
GeneCard	RAB11B
GeneCard	ATL3
GeneCard	PPP1R14A
GeneCard	JUNB
GeneCard	MIR219A2
GeneCard	MAPK13
GeneCard	ACACB
GeneCard	PLXNA1
GeneCard	SAP30BP
GeneCard	CLDN5
GeneCard	NRN1
GeneCard	KIR2DS4
GeneCard	ARIH2
GeneCard	LATS1
GeneCard	RPS16
GeneCard	TBC1D15
GeneCard	TNFAIP6
GeneCard	ACAD11
GeneCard	CDK12
GeneCard	AGPAT1
GeneCard	RPL4
GeneCard	CLINT1

GeneCard	H4C3
GeneCard	PTPN5
GeneCard	EIF4A3
GeneCard	PPP1R10
GeneCard	PPM1B
GeneCard	ACER3
GeneCard	GULOP
GeneCard	PAICS
GeneCard	MCM5
GeneCard	IL20
GeneCard	PSMD13
GeneCard	SRSF9
GeneCard	SEMA3F
GeneCard	TPD52
GeneCard	GPRASP2
GeneCard	CASP5
GeneCard	OLFML3
GeneCard	ANKFY1
GeneCard	IZUMO1
GeneCard	NXF1
GeneCard	NCAM2
GeneCard	NDUFA3
GeneCard	H4C2
GeneCard	ASRGL1
GeneCard	DERL2
GeneCard	RPL12
GeneCard	SLC5A4
GeneCard	CHP2
GeneCard	UNC13C
GeneCard	H4C6
GeneCard	STAMBPL1
GeneCard	ATG4B
GeneCard	SEMA4D
GeneCard	NUB1
GeneCard	ING1
GeneCard	CLPP
GeneCard	LDB1
GeneCard	H4C14
GeneCard	H4C15
GeneCard	BOK
GeneCard	AQP10
GeneCard	H4C12
GeneCard	POLI
GeneCard	EPC1
GeneCard	CTNNBL1
GeneCard	RIMBP2
GeneCard	MAMLD1
GeneCard	RIPPLY3
GeneCard	SF3B2
GeneCard	SLCO1A2
GeneCard	TRIM39
GeneCard	CENPT
GeneCard	TOMM70
GeneCard	SLC12A9
GeneCard	UBAC1
GeneCard	MAP3K6
GeneCard	DHRS11
GeneCard	B3GAT1

GeneCard	GLIS1
GeneCard	RAB35
GeneCard	H4C5
GeneCard	SRSF7
GeneCard	SNX2
GeneCard	NCBP1
GeneCard	MED1
GeneCard	UQCR10
GeneCard	HLA-DQB2
GeneCard	SPTBN1
GeneCard	CLIC1
GeneCard	CARD16
GeneCard	SIX2
GeneCard	PPP1R3B
GeneCard	MIR124-2
GeneCard	LUZP2
GeneCard	AOX1
GeneCard	QARS1
GeneCard	CYB561
GeneCard	MIR125B2
GeneCard	MAB21L2
GeneCard	HYOU1
GeneCard	RPH3A
GeneCard	PDCD6IP
GeneCard	SERPINB6
GeneCard	HSD17B12
GeneCard	HAND1
GeneCard	PLIN3
GeneCard	TMLHE
GeneCard	GAPDHS
GeneCard	ASXL2
GeneCard	C5AR2
GeneCard	TMSB4X
GeneCard	CCDC88A
GeneCard	PLK4
GeneCard	TLN2
GeneCard	BCAT2
GeneCard	PAEP
GeneCard	MCFD2
GeneCard	GBGT1
GeneCard	RBMS3
GeneCard	CSN1S1
GeneCard	MYBBP1A
GeneCard	NHLRC2
GeneCard	PUM1
GeneCard	PCBP2
GeneCard	RANGAP1
GeneCard	ATP5MC1
GeneCard	RUVBL1
GeneCard	H2AC20
GeneCard	TLE1
GeneCard	PHYHIP
GeneCard	KLK10
GeneCard	PCDHA11
GeneCard	ADAM22
GeneCard	ATP5PB
GeneCard	SNAP91
GeneCard	INSRR

GeneCard	HCRTR1
GeneCard	PSMD6
GeneCard	CCN4
GeneCard	CCL1
GeneCard	NAT16
GeneCard	KPNA1
GeneCard	NCKAP1L
GeneCard	FBXW11
GeneCard	LGALS3BP
GeneCard	RAB4A
GeneCard	BAG1
GeneCard	RPS4X
GeneCard	SNX9
GeneCard	ATP6V1E1
GeneCard	CBSL
GeneCard	OARD1
GeneCard	WDR12
GeneCard	H3C1
GeneCard	RSU1
GeneCard	HAGH
GeneCard	CFAP91
GeneCard	MYOM3
GeneCard	OSTF1
GeneCard	DCD
GeneCard	GALP
GeneCard	SUMF2
GeneCard	DPYS
GeneCard	MIR188
GeneCard	IGBP1
GeneCard	UGP2
GeneCard	TSHZ1
GeneCard	SPAG6
GeneCard	SCG5
GeneCard	PALD1
GeneCard	MARCO
GeneCard	NLGN2
GeneCard	RAB38
GeneCard	CNTNAP5
GeneCard	SEC22B
GeneCard	CST6
GeneCard	CALCRL
GeneCard	MIR129-2
GeneCard	LOC109504727
GeneCard	DNAJC14
GeneCard	IL34
GeneCard	CXCL16
GeneCard	SLC16A1
GeneCard	LTB4R
GeneCard	NPY2R
GeneCard	ZFAND2B
GeneCard	MVP
GeneCard	SLC1A5
GeneCard	FUNDC1
GeneCard	NDUFAF7
GeneCard	ADARB1
GeneCard	IGFBP6
GeneCard	ZFPM1
GeneCard	FAT1

GeneCard	NDUFB7
GeneCard	HTR1D
GeneCard	SEC31A
GeneCard	H1-5
GeneCard	ORC4
GeneCard	MIR138-2
GeneCard	RBM45
GeneCard	FLRT3
GeneCard	FEZ1
GeneCard	CDC34
GeneCard	NFE2
GeneCard	RERGL
GeneCard	CHRNA9
GeneCard	SYT14
GeneCard	FRA10AC1
GeneCard	PHOSPHO1
GeneCard	RPL9
GeneCard	IPO5
GeneCard	TRIB3
GeneCard	PDE3B
GeneCard	ACAT2
GeneCard	BUB3
GeneCard	CD48
GeneCard	JAM3
GeneCard	SLC22A1
GeneCard	U2AF2
GeneCard	ETV1
GeneCard	TEFM
GeneCard	CPQ
GeneCard	DMAP1
GeneCard	RRS1
GeneCard	LY86
GeneCard	WAPL
GeneCard	LRRTM1
GeneCard	VWA2
GeneCard	UTP4
GeneCard	FAM111B
GeneCard	PGRMC1
GeneCard	AVPR1B
GeneCard	PLA2G3
GeneCard	TRIM9
GeneCard	DYNLT1
GeneCard	KIF2B
GeneCard	INTS1
GeneCard	UBXN7
GeneCard	GRM4
GeneCard	MRPS27
GeneCard	UBE2G1
GeneCard	AEBP2
GeneCard	CLEC11A
GeneCard	COX14
GeneCard	GRIK3
GeneCard	TNXA
GeneCard	STX8
GeneCard	JPH4
GeneCard	LOC110973015
GeneCard	QPRT
GeneCard	UBE2J1

GeneCard	TBC1D7
GeneCard	KIR2DS2
GeneCard	SRPK2
GeneCard	BZW2
GeneCard	PCBP1
GeneCard	RNU1-1
GeneCard	MAPKAPK5
GeneCard	CMYA5
GeneCard	CLTB
GeneCard	SP3
GeneCard	IFNG-AS1
GeneCard	NPL
GeneCard	SLC7A1
GeneCard	PIM1
GeneCard	KLKB1
GeneCard	GAS7
GeneCard	LRG1
GeneCard	UBE2I
GeneCard	NAGK
GeneCard	INO80
GeneCard	EGLN2
GeneCard	SORCS2
GeneCard	AOAH
GeneCard	ACTR1B
GeneCard	BCAN
GeneCard	KRT6C
GeneCard	MTUS1
GeneCard	LINC01734
GeneCard	CYP26C1
GeneCard	HSPB6
GeneCard	SHE
GeneCard	DFFB
GeneCard	EFCAB6
GeneCard	CDK7
GeneCard	DRC7
GeneCard	ADH4
GeneCard	SYT12
GeneCard	NIT2
GeneCard	MIR24-2
GeneCard	PIP4K2A
GeneCard	IFIT3
GeneCard	CAMKMT
GeneCard	MS4A3
GeneCard	PPP2R3B
GeneCard	CD248
GeneCard	SLC7A8
GeneCard	P2RX4
GeneCard	ECHDC3
GeneCard	PLA2G2D
GeneCard	MAP3K2
GeneCard	DYNC1I2
GeneCard	SLC16A9
GeneCard	SORBS1
GeneCard	CUL7
GeneCard	MAPK8IP3
GeneCard	SLC4A7
GeneCard	RAB21
GeneCard	CYP26B1

GeneCard	TBC1D5
GeneCard	GPM6B
GeneCard	SOBP
GeneCard	THADA
GeneCard	MYL1
GeneCard	DUSP22
GeneCard	PRRC2C
GeneCard	NEUROG2
GeneCard	RASD1
GeneCard	PIWIL1
GeneCard	NAAA
GeneCard	KIR2DL5A
GeneCard	MIR369
GeneCard	TMOD2
GeneCard	SPATA5
GeneCard	RTRAF
GeneCard	RPL36
GeneCard	DDX20
GeneCard	CRHR2
GeneCard	PANK4
GeneCard	SEMA6A
GeneCard	ASAH2B
GeneCard	SLC32A1
GeneCard	ANXA9
GeneCard	DUSP13
GeneCard	WFDC2
GeneCard	GCM1
GeneCard	SCARA3
GeneCard	MSRB3
GeneCard	RPL24
GeneCard	CTDSPL
GeneCard	CORT
GeneCard	BACH1
GeneCard	PPP2R3C
GeneCard	ELK1
GeneCard	SIRT5
GeneCard	RUNX1T1
GeneCard	FBF1
GeneCard	GRIK4
GeneCard	TMEM132D
GeneCard	NR4A3
GeneCard	TFB2M
GeneCard	LRIG3
GeneCard	NGB
GeneCard	VPS26C
GeneCard	SERBP1
GeneCard	TUBAL3
GeneCard	DIO3
GeneCard	ISG20
GeneCard	NFKBIB
GeneCard	NT5C3A
GeneCard	DUSP19
GeneCard	SIM2
GeneCard	CLPTM1
GeneCard	STIM2
GeneCard	CDC123
GeneCard	CAPNS2
GeneCard	PHB2

GeneCard	DNALI1
GeneCard	GUK1
GeneCard	TMPRSS4
GeneCard	PARVB
GeneCard	ANKK1
GeneCard	LOXL4
GeneCard	SLC27A2
GeneCard	RNF5
GeneCard	SEC31B
GeneCard	CALCOCO2
GeneCard	GULP1
GeneCard	PPP3R2
GeneCard	MIR598
GeneCard	EPS8L3
GeneCard	LINGO2
GeneCard	IFIT2
GeneCard	AKAP1
GeneCard	ANAPC1
GeneCard	DLX6
GeneCard	PELP1
GeneCard	CCNK
GeneCard	CDH10
GeneCard	MIR6843
GeneCard	ZC3H10
GeneCard	MIR874
GeneCard	TUSC1
GeneCard	EEF1G
GeneCard	SPPL2B
GeneCard	ZCCHC8
GeneCard	KLK2
GeneCard	NBPF3
GeneCard	TSNAX-DISC1
GeneCard	FXR2
GeneCard	MIR873
GeneCard	MRPS6
GeneCard	TIMELESS
GeneCard	SPPL2C
GeneCard	RCAN3
GeneCard	PSMB6
GeneCard	ELF5
GeneCard	GEMIN8
GeneCard	GRB10
GeneCard	RAB2A
GeneCard	WDR45B
GeneCard	SFN
GeneCard	LILRB2
GeneCard	SAT1
GeneCard	KIAA1191
GeneCard	IFITM3
GeneCard	LMAN2L
GeneCard	FZD7
GeneCard	EXOSC5
GeneCard	RPS8
GeneCard	NEURL1
GeneCard	KRTCAP2
GeneCard	TSPAN2
GeneCard	NTNG2
GeneCard	ALDH1L1

GeneCard	HCN2
GeneCard	MMP16
GeneCard	NUP54
GeneCard	GGA2
GeneCard	INSIG1
GeneCard	RNF144A
GeneCard	RNF114
GeneCard	AVPR1A
GeneCard	MIR181D
GeneCard	TUBB8
GeneCard	VCPKMT
GeneCard	NDRG4
GeneCard	PDIA3
GeneCard	CDC37
GeneCard	SFXN5
GeneCard	PDE7B
GeneCard	CCT8
GeneCard	ILF3
GeneCard	WDFY4
GeneCard	MYO16
GeneCard	NTSR1
GeneCard	YIF1A
GeneCard	COX6A2
GeneCard	ESRRG
GeneCard	PIK3AP1
GeneCard	XRN2
GeneCard	USP6NL
GeneCard	PDZK1
GeneCard	GEMIN7
GeneCard	FABP12
GeneCard	RANBP9
GeneCard	PGBD1
GeneCard	PPP1CA
GeneCard	PKP3
GeneCard	RHBDF1
GeneCard	VMP1
GeneCard	TK1
GeneCard	ARF6
GeneCard	MRPS18A
GeneCard	ATP5MC3
GeneCard	CENPV
GeneCard	F11R
GeneCard	IRAK1BP1
GeneCard	PEA15
GeneCard	RIPPLY2
GeneCard	ZNF521
GeneCard	GSTA1
GeneCard	TAOK2
GeneCard	TARBP2
GeneCard	FTSJ3
GeneCard	CCL23
GeneCard	NAPEPLD
GeneCard	RPS6KA2
GeneCard	TYSND1
GeneCard	SLC16A12
GeneCard	NUMB
GeneCard	SRSF5
GeneCard	ARNTL2

GeneCard	TCF19
GeneCard	RNF139
GeneCard	ID4
GeneCard	MTCL1
GeneCard	B3GALT4
GeneCard	BYSL
GeneCard	ARFGAP1
GeneCard	PTPRA
GeneCard	RNF32
GeneCard	PROSER1
GeneCard	TBC1D17
GeneCard	ZYX
GeneCard	NKAIN2
GeneCard	MYH15
GeneCard	SNU13
GeneCard	MTF1
GeneCard	ADRM1
GeneCard	APOBEC3F
GeneCard	CCNE2
GeneCard	WBP1L
GeneCard	TCP1
GeneCard	DIP2C
GeneCard	DEPTOR
GeneCard	PPRC1
GeneCard	CYB561D2
GeneCard	GTF2B
GeneCard	MTNR1A
GeneCard	LPXN
GeneCard	MAP1LC3C
GeneCard	CD83
GeneCard	IMPA2
GeneCard	PRDM9
GeneCard	CHPF2
GeneCard	RFC3
GeneCard	CSTF1
GeneCard	RANBP17
GeneCard	MRPL9
GeneCard	SLC22A3
GeneCard	SH2D1B
GeneCard	LSP1
GeneCard	CPT1B
GeneCard	ROR1
GeneCard	TIAF1
GeneCard	IDI2
GeneCard	UBE2V1
GeneCard	CDC20
GeneCard	UBA7
GeneCard	DENND2B
GeneCard	RHOC
GeneCard	DCXR
GeneCard	NPAS3
GeneCard	ACIN1
GeneCard	GPC2
GeneCard	HEG1
GeneCard	RPL22
GeneCard	RIC8B
GeneCard	NPLOC4
GeneCard	SULF1

GeneCard	PSMD5
GeneCard	MOV10
GeneCard	MEX3D
GeneCard	SEPTIN12
GeneCard	EEF2K
GeneCard	ABCF1
GeneCard	HNRNPUL1
GeneCard	ENTPD7
GeneCard	LIG3
GeneCard	RPL14
GeneCard	UBXN10
GeneCard	FBP2
GeneCard	MIEF1
GeneCard	THTPA
GeneCard	ALYREF
GeneCard	GALNT12
GeneCard	UCHL1-DT
GeneCard	PTPRK
GeneCard	RPL7L1
GeneCard	PTGIR
GeneCard	SEC13
GeneCard	MIR1275
GeneCard	PAPPA2
GeneCard	IGF2BP3
GeneCard	MIR495
GeneCard	CNTN6
GeneCard	RCAN2
GeneCard	MRPS14
GeneCard	MAPRE1
GeneCard	KHSRP
GeneCard	DMTN
GeneCard	H1-0
GeneCard	HIPK2
GeneCard	CASP14
GeneCard	STX6
GeneCard	MUC17
GeneCard	NDUFB5
GeneCard	RXRG
GeneCard	EIF3A
GeneCard	SLC15A2
GeneCard	NDUFV3
GeneCard	ADAMTS6
GeneCard	POLN
GeneCard	EXO1
GeneCard	RPL37
GeneCard	GMEB1
GeneCard	ZXDC
GeneCard	HEXIM1
GeneCard	INKA1
GeneCard	S1PR3
GeneCard	POLE3
GeneCard	FOXA1
GeneCard	KAT2B
GeneCard	MPHOSPH8
GeneCard	ACKR4
GeneCard	NCK1
GeneCard	TRAPPC4
GeneCard	SUPT16H

GeneCard	SLC4A1AP
GeneCard	GRIK1
GeneCard	USP1
GeneCard	SCFD1
GeneCard	VPS4A
GeneCard	USP34
GeneCard	SLC4A8
GeneCard	ST18
GeneCard	CARF
GeneCard	CEP295NL
GeneCard	KLF15
GeneCard	CELF4
GeneCard	RPS21
GeneCard	PRUNE2
GeneCard	F8A1
GeneCard	HSPH1
GeneCard	MRRF
GeneCard	MRAP2
GeneCard	MTMR6
GeneCard	GAR1
GeneCard	MRPL10
GeneCard	CAND1
GeneCard	MYEF2
GeneCard	HMGB2
GeneCard	PLK2
GeneCard	RBBP4
GeneCard	PDE4DIP
GeneCard	PACSIN2
GeneCard	PRKAR2A
GeneCard	PTK7
GeneCard	RPL8
GeneCard	CDHR3
GeneCard	DCUN1D5
GeneCard	GABARAPL2
GeneCard	MAPRE3
GeneCard	GPAM
GeneCard	TPX2
GeneCard	NDUFB1
GeneCard	CPEB1
GeneCard	RNF212
GeneCard	ELL
GeneCard	FLT3LG
GeneCard	FGF22
GeneCard	COX7A2L
GeneCard	NME2
GeneCard	DOT1L
GeneCard	CCNT2
GeneCard	ENPEP
GeneCard	ATP1A4
GeneCard	RASSF5
GeneCard	NCBP2
GeneCard	C21orf91
GeneCard	MOB2
GeneCard	MELK
GeneCard	MUC15
GeneCard	SMG8
GeneCard	SYNPO2L
GeneCard	RNF182

GeneCard	GPR84
GeneCard	CFAP53
GeneCard	FAM13C
GeneCard	COX7A2
GeneCard	SPRED2
GeneCard	GSTZ1
GeneCard	RSRC2
GeneCard	CROT
GeneCard	SLC6A12
GeneCard	SFRP2
GeneCard	MTRNR2L6
GeneCard	PHACTR4
GeneCard	KCNK5
GeneCard	HRK
GeneCard	ATF3
GeneCard	DECR2
GeneCard	ANGPT4
GeneCard	PLXNA3
GeneCard	AKAP12
GeneCard	GPR34
GeneCard	TIAM1
GeneCard	NEUROD6
GeneCard	CNNM1
GeneCard	FSTL1
GeneCard	GRID1
GeneCard	TIAL1
GeneCard	EPB41L3
GeneCard	MAP3K11
GeneCard	UBE2Q1
GeneCard	RPL32
GeneCard	WTAPP1
GeneCard	CCNG1
GeneCard	TRAF5
GeneCard	IFNL1
GeneCard	RAP1B
GeneCard	EREG
GeneCard	NEIL1
GeneCard	HNRNPR
GeneCard	P2RY1
GeneCard	MIR802
GeneCard	UBE2Z
GeneCard	SEC16A
GeneCard	MRPL45
GeneCard	ZNF320
GeneCard	EXOSC2
GeneCard	MIR218-2
GeneCard	RNF112
GeneCard	RGS10
GeneCard	CRYM
GeneCard	CSN2
GeneCard	CPSF6
GeneCard	FES
GeneCard	AHCYL1
GeneCard	BAG6
GeneCard	ZDHHC8
GeneCard	SDF4
GeneCard	RABL3
GeneCard	CD200R1

GeneCard	VAT1L
GeneCard	CDIPT
GeneCard	NFATC2
GeneCard	MTRNR2L5
GeneCard	CISD1
GeneCard	SEPTIN8
GeneCard	SYTL2
GeneCard	ADAMTS9-AS2
GeneCard	EXOC6
GeneCard	EPHA1-AS1
GeneCard	EIF2AK1
GeneCard	CLPTM1L
GeneCard	TCF7
GeneCard	GIMAP5
GeneCard	MYO7B
GeneCard	MRPS25
GeneCard	SP2
GeneCard	VWA5A
GeneCard	GPR158
GeneCard	NDUFB4
GeneCard	ETV4
GeneCard	PCGF5
GeneCard	KPTN
GeneCard	CBX5
GeneCard	EXOSC1
GeneCard	OBI1-AS1
GeneCard	SCAMP1
GeneCard	NBR1
GeneCard	OGDHL
GeneCard	ARF4
GeneCard	HSD17B8
GeneCard	PTGES3
GeneCard	IRF2BP1
GeneCard	BDH1
GeneCard	SEC23IP
GeneCard	ANKRD2
GeneCard	DNAJA1
GeneCard	SERF1A
GeneCard	ANKRD13D
GeneCard	DSCR8
GeneCard	ACTR2
GeneCard	TUBA3E
GeneCard	AP1M2
GeneCard	CCL25
GeneCard	NMU
GeneCard	MTRNR2L4
GeneCard	PIK3C2G
GeneCard	CAPN9
GeneCard	TSPYL1
GeneCard	CHRM5
GeneCard	CKAP5
GeneCard	IER3
GeneCard	DRG1
GeneCard	DAP3
GeneCard	MGAT3
GeneCard	CAPRIN1
GeneCard	HDGF
GeneCard	DUSP5

GeneCard	HSPA14
GeneCard	MIR431
GeneCard	PDLIM3
GeneCard	RHOB
GeneCard	MAPKAP1
GeneCard	LINC01262
GeneCard	ARL5B
GeneCard	USP48
GeneCard	ZNF2
GeneCard	PRMT1
GeneCard	DSCR9
GeneCard	ECD
GeneCard	ACSBG1
GeneCard	CAPN15
GeneCard	ITIH3
GeneCard	NSL1
GeneCard	MSI2
GeneCard	RARG
GeneCard	PLXDC2
GeneCard	POLL
GeneCard	BNIP1
GeneCard	UCH1LAS
GeneCard	CCT6A
GeneCard	MDK
GeneCard	PRDX4
GeneCard	IRF2BP2
GeneCard	BNIP3
GeneCard	CIBAR1
GeneCard	CYP21A1P
GeneCard	DSTN
GeneCard	ITPKB
GeneCard	PSME2
GeneCard	DEFA1
GeneCard	SLC8A3
GeneCard	KIN
GeneCard	NPY4R
GeneCard	ITGB1BP1
GeneCard	SSTR4
GeneCard	STAMBP
GeneCard	GABRA4
GeneCard	NDUFA7
GeneCard	PMAIP1
GeneCard	SRSF4
GeneCard	MPP1
GeneCard	DYDC1
GeneCard	RAB4B
GeneCard	STEEP1
GeneCard	KCNN1
GeneCard	EPDR1
GeneCard	SLC6A17
GeneCard	BAG5
GeneCard	KATNAL2
GeneCard	RPL29
GeneCard	TECTB
GeneCard	RPS6KA5
GeneCard	USP25
GeneCard	CLK3
GeneCard	EIF2S2

GeneCard	BVES
GeneCard	TP53INP1
GeneCard	NME9
GeneCard	PYM1
GeneCard	OSBPL7
GeneCard	MIR769
GeneCard	SNX4
GeneCard	H2BS1
GeneCard	APCDD1
GeneCard	LOC109461478
GeneCard	S100A11
GeneCard	CRYBA2
GeneCard	ZBP1
GeneCard	CLEC3B
GeneCard	MIR30C2
GeneCard	KRI1
GeneCard	ZNF558
GeneCard	BRD3
GeneCard	PIAS1
GeneCard	JUND
GeneCard	LASP1
GeneCard	AQR
GeneCard	PLD2
GeneCard	ZNF180
GeneCard	NOP2
GeneCard	CLTCL1
GeneCard	PLRG1
GeneCard	TSC22D3
GeneCard	PSME3
GeneCard	DDX23
GeneCard	LINC02914
GeneCard	EIF4B
GeneCard	MCEE
GeneCard	FOXL1
GeneCard	OLAH
GeneCard	DYDC2
GeneCard	TRIM24
GeneCard	GPLD1
GeneCard	NOVA1
GeneCard	MIR512-1
GeneCard	KAT8
GeneCard	GABRG1
GeneCard	PRKAB2
GeneCard	LOC100287944
GeneCard	H3C14
GeneCard	PALM
GeneCard	NPTX1
GeneCard	DSCR4
GeneCard	RAB3D
GeneCard	DHRS9
GeneCard	TLL2
GeneCard	FHL3
GeneCard	GPSM3
GeneCard	PKP4
GeneCard	HTT-AS
GeneCard	NLRP2
GeneCard	ACSL6
GeneCard	NUAK1

GeneCard	CLRN3
GeneCard	STAU1
GeneCard	LOC111365141
GeneCard	ARHGAP15
GeneCard	E2F8
GeneCard	FMR1-AS1
GeneCard	CHRNA6
GeneCard	GRPR
GeneCard	PRMT5
GeneCard	DDX5
GeneCard	SCOC-AS1
GeneCard	GIMAP4
GeneCard	NPY1R
GeneCard	FCER1G
GeneCard	SLC2A14
GeneCard	TOM1
GeneCard	MT-TC
GeneCard	NRAP
GeneCard	MYO10
GeneCard	SLC28A1
GeneCard	RPL36A
GeneCard	E2F5
GeneCard	DHX8
GeneCard	SAP30L
GeneCard	CAMK2N2
GeneCard	MAD2L1
GeneCard	DCLK1
GeneCard	RMND5B
GeneCard	CCAR2
GeneCard	SLC30A2
GeneCard	DTHD1
GeneCard	SQOR
GeneCard	ERP44
GeneCard	SRPK1
GeneCard	EEFSEC
GeneCard	PLEKHG1
GeneCard	SMPD3
GeneCard	NAPSA
GeneCard	MIR4422HG
GeneCard	DMRTA1
GeneCard	SLC25A38
GeneCard	GNAZ
GeneCard	RBMS1
GeneCard	IGF2BP1
GeneCard	CYB5B
GeneCard	PRPSAP2
GeneCard	SCIMP
GeneCard	SELENOS
GeneCard	CALML4
GeneCard	C4BPB
GeneCard	GCNT2
GeneCard	MRPL39
GeneCard	PNPLA7
GeneCard	FZD9
GeneCard	ZPBP
GeneCard	LGALS8
GeneCard	TDRD10
GeneCard	RCC1L

GeneCard	TREML1
GeneCard	E2F7
GeneCard	USP2
GeneCard	RNF115
GeneCard	PPP2CB
GeneCard	PCSK7
GeneCard	SCAF4
GeneCard	HKDC1
GeneCard	LINC01194
GeneCard	NAALADL2
GeneCard	GRIK5
GeneCard	HRC
GeneCard	METTL8
GeneCard	E2F6
GeneCard	KNSTRN
GeneCard	ZBTB38
GeneCard	MIR520C
GeneCard	MDM4
GeneCard	CHD6
GeneCard	SLC2A13
GeneCard	DMXL1
GeneCard	AP3M1
GeneCard	TRMT1L
GeneCard	CALHM2
GeneCard	HNRNPD
GeneCard	LOC108660406
GeneCard	CALHM3
GeneCard	MIR9-2
GeneCard	XAB2
GeneCard	EIF6
GeneCard	NELFE
GeneCard	TRUB1
GeneCard	LCP2
GeneCard	SORBS3
GeneCard	LRIT1
GeneCard	PDCL
GeneCard	BASP1
GeneCard	DCLRE1B
GeneCard	ARFGAP3
GeneCard	MEF2D
GeneCard	RPL26L1
GeneCard	MYOF
GeneCard	PRKAG3
GeneCard	COX7A1
GeneCard	TVP23A
GeneCard	DOCK4
GeneCard	PALMD
GeneCard	MRPL28
GeneCard	RPL18A
GeneCard	BMX
GeneCard	PPP1CC
GeneCard	FRK
GeneCard	PPP1R12A
GeneCard	MYLPF
GeneCard	TSNAX
GeneCard	HDAC10
GeneCard	WRAP73
GeneCard	SLC4A3

GeneCard	TXNDC12
GeneCard	PABPC4
GeneCard	PPP2R5E
GeneCard	EIF1AX
GeneCard	TMCO4
GeneCard	FPR1
GeneCard	NOLC1
GeneCard	TMEM147
GeneCard	TSHZ2
GeneCard	NMB
GeneCard	HSD17B13
GeneCard	SEZ6L2
GeneCard	BBC3
GeneCard	NAP1L1
GeneCard	TREML4
GeneCard	SRBD1
GeneCard	FBXO42
GeneCard	SLC6A13
GeneCard	SRI
GeneCard	MRPS28
GeneCard	MIR3200
GeneCard	CD93
GeneCard	ATP5MC2
GeneCard	IQGAP2
GeneCard	NOL3
GeneCard	PADI3
GeneCard	POLR2H
GeneCard	ULK4
GeneCard	MYOZ1
GeneCard	NKX6-2
GeneCard	IFIT1
GeneCard	PTGFR
GeneCard	SFXN2
GeneCard	SYPL2
GeneCard	ADCY3
GeneCard	AKR1C1
GeneCard	CUEDC2
GeneCard	RPL36AL
GeneCard	TMEM30A
GeneCard	LOC108660405
GeneCard	GTF2H1
GeneCard	PTPRU
GeneCard	EGLN3
GeneCard	TBPL1
GeneCard	GIMAP2
GeneCard	ID1
GeneCard	DYNLL2
GeneCard	SLF2
GeneCard	VPS28
GeneCard	ANKRD22
GeneCard	CSGALNACT2
GeneCard	CARM1
GeneCard	TSR1
GeneCard	BPNT2
GeneCard	RPL23A
GeneCard	MIA3
GeneCard	MIR135A2
GeneCard	TSPAN15

GeneCard	UQCR11
GeneCard	NDUFB2
GeneCard	CADM2
GeneCard	LIN7A
GeneCard	RETREG3
GeneCard	PLEKHG2
GeneCard	LOC109623489
GeneCard	NCAPD3
GeneCard	LIMD1
GeneCard	MCM10
GeneCard	LAPTM4A
GeneCard	SEPHS1
GeneCard	EFR3A
GeneCard	NUCKS1
GeneCard	ITSN2
GeneCard	AGAP1
GeneCard	GLRX3
GeneCard	HPCAL1
GeneCard	CCDC85C
GeneCard	MIR103A2
GeneCard	MLF1
GeneCard	PHAX
GeneCard	ARHGAP20
GeneCard	TMEM59
GeneCard	PLCL1
GeneCard	TPBG
GeneCard	DYNC1I1
GeneCard	TACC2
GeneCard	UPF2
GeneCard	RABEP2
GeneCard	NCDN
GeneCard	KCTD2
GeneCard	SELENOO
GeneCard	NIF3L1
GeneCard	GTF2H3
GeneCard	TRPM2
GeneCard	PFDN1
GeneCard	NCK2
GeneCard	NOC2L
GeneCard	ABL2
GeneCard	RAB27B
GeneCard	RBM15
GeneCard	USE1
GeneCard	RPE
GeneCard	H3C6
GeneCard	SLC27A1
GeneCard	DDX18
GeneCard	MACIR
GeneCard	PSD
GeneCard	ADD2
GeneCard	STK25
GeneCard	LIPM
GeneCard	SNRNP40
GeneCard	POLR2K
GeneCard	PPARGC1B
GeneCard	WASF2
GeneCard	BCAR3
GeneCard	BAIAP2L1

GeneCard	CACNG3
GeneCard	SESN2
GeneCard	CALY
GeneCard	IKZF4
GeneCard	HECW1
GeneCard	RPL37A
GeneCard	PTPRF
GeneCard	STX5
GeneCard	EFNA3
GeneCard	PTPRT
GeneCard	MAP4
GeneCard	TGFB1I1
GeneCard	GEMIN5
GeneCard	TSPAN8
GeneCard	APOO
GeneCard	PRIM1
GeneCard	SLC25A27
GeneCard	NKAP
GeneCard	OXSR1
GeneCard	GADD45G
GeneCard	RYBP
GeneCard	ACSL5
GeneCard	LSAMP
GeneCard	ENTPD5
GeneCard	IL4I1
GeneCard	GSPT2
GeneCard	MIR410
GeneCard	CSMD2
GeneCard	MRPL16
GeneCard	AGAP3
GeneCard	VSTM4
GeneCard	ATG2A
GeneCard	SCAMP5
GeneCard	QPCTL
GeneCard	PPP2R5C
GeneCard	CUTA
GeneCard	AGO1
GeneCard	BLID
GeneCard	KIAA0825
GeneCard	SF3B3
GeneCard	UBE2W
GeneCard	COX7C
GeneCard	DNAH14
GeneCard	CERK
GeneCard	CFAP70
GeneCard	GRK6
GeneCard	TSN
GeneCard	FNBP1
GeneCard	IFRD2
GeneCard	CXXC1
GeneCard	SERPINB9
GeneCard	ZFP90
GeneCard	RRP15
GeneCard	AQP9
GeneCard	ZMYM3
GeneCard	ERI3
GeneCard	STXBP6
GeneCard	TARBP1

GeneCard	ABCC11
GeneCard	TIGD1
GeneCard	FBXW4
GeneCard	SLIRP
GeneCard	MIER1
GeneCard	SART1
GeneCard	MRTO4
GeneCard	EIF3B
GeneCard	RRP1B
GeneCard	MPRIP
GeneCard	DSCR10
GeneCard	HES1
GeneCard	FAAP24
GeneCard	USP10
GeneCard	INKA2
GeneCard	DXO
GeneCard	KIFC3
GeneCard	MRPL43
GeneCard	ACSL3
GeneCard	RUVBL2
GeneCard	HYAL2
GeneCard	USP18
GeneCard	FAM215A
GeneCard	TRIM26
GeneCard	ARHGEF1
GeneCard	CDC42SE2
GeneCard	ANAPC13
GeneCard	LINC00271
GeneCard	LHPP
GeneCard	FGFBP2
GeneCard	HID1
GeneCard	UQCRH
GeneCard	CEP170
GeneCard	PIP4K2C
GeneCard	RAB5C
GeneCard	SYNGR1
GeneCard	EPC2
GeneCard	CXCL14
GeneCard	CSDE1
GeneCard	MS
GeneCard	LINC01567
GeneCard	MED30
GeneCard	GGH
GeneCard	CHD5
GeneCard	MEPCE
GeneCard	NUDT16L1
GeneCard	TTLL7
GeneCard	SND1
GeneCard	ASPSCR1
GeneCard	SLC39A12
GeneCard	NCALD
GeneCard	SAV1
GeneCard	ANP32B
GeneCard	C4B_2
GeneCard	TNMD
GeneCard	CETN2
GeneCard	LDLRAD2
GeneCard	WIPI2

GeneCard	VTI1A
GeneCard	LINC00316
GeneCard	CHMP3
GeneCard	MAGI1
GeneCard	COX8C
GeneCard	TAX1BP3
GeneCard	CMKLR1
GeneCard	TRIB2
GeneCard	MPST
GeneCard	PURB
GeneCard	IL22RA1
GeneCard	ZSCAN25
GeneCard	CLDN18
GeneCard	LMTK2
GeneCard	PKNOX2
GeneCard	CDK18
GeneCard	A3GALT2
GeneCard	TPTEP2-CSNK1E
GeneCard	DNAJC7
GeneCard	FHL5
GeneCard	OSBPL6
GeneCard	CHN2
GeneCard	AFDN
GeneCard	STYX
GeneCard	NMT2
GeneCard	CSN3
GeneCard	CPSF4
GeneCard	RNF185
GeneCard	PDIA4
GeneCard	TKTL1
GeneCard	TUT1
GeneCard	RAB3C
GeneCard	SOX7
GeneCard	ELOA
GeneCard	MIR488
GeneCard	MAGEA4
GeneCard	MLEC
GeneCard	MLLT3
GeneCard	KCNK18
GeneCard	ELOVL6
GeneCard	CDC23
GeneCard	SERF1B
GeneCard	SOX14
GeneCard	KPNA4
GeneCard	SH3GLB1
GeneCard	ECRG4
GeneCard	ADAM15
GeneCard	HLA-DRB9
GeneCard	MIR155HG
GeneCard	SYNJ2BP
GeneCard	UBXN11
GeneCard	OFCC1
GeneCard	BHMT2
GeneCard	CWC15
GeneCard	RPP30
GeneCard	WDR7
GeneCard	FAM131B
GeneCard	CD74

GeneCard	TRMO
GeneCard	BBX
GeneCard	LHX8
GeneCard	PLXNA4
GeneCard	CDK11B
GeneCard	UBE2L6
GeneCard	BTN3A2
GeneCard	FBH1
GeneCard	ARL1
GeneCard	CPM
GeneCard	ENAH
GeneCard	ZNF45
GeneCard	ANO4
GeneCard	ZNF594-DT
GeneCard	NMT1
GeneCard	KYAT1
GeneCard	SF3B6
GeneCard	SLK
GeneCard	KIFC1
GeneCard	PPP1R37
GeneCard	MIR101-2
GeneCard	RBM12
GeneCard	MTRNR2L8
GeneCard	SBNO2
GeneCard	CCAR1
GeneCard	CIP2A
GeneCard	SSH1
GeneCard	TBRG1
GeneCard	PTPRZ1
GeneCard	RASSF8
GeneCard	VAMP8
GeneCard	ATP6V1G1
GeneCard	GPR15
GeneCard	NDST2
GeneCard	COIL
GeneCard	BAALC
GeneCard	TRIM11
GeneCard	MED8
GeneCard	LRRFIP1
GeneCard	SNAP23
GeneCard	C6orf58
GeneCard	GNPNAT1
GeneCard	SLC25A31
GeneCard	NDUFC1
GeneCard	LHFPL2
GeneCard	MAGOHB
GeneCard	GDI2
GeneCard	MESP1
GeneCard	GMFB
GeneCard	NOP14
GeneCard	TAF11
GeneCard	GALR1
GeneCard	ATP2B4
GeneCard	TNFAIP1
GeneCard	CDCA5
GeneCard	ALG10
GeneCard	SREK1
GeneCard	TMBIM6

GeneCard	GNB1L
GeneCard	MIR876
GeneCard	ATP6V1H
GeneCard	DCLRE1A
GeneCard	CLIC4
GeneCard	PAXIP1
GeneCard	RPL34
GeneCard	PDIA6
GeneCard	SURF4
GeneCard	PTGES2
GeneCard	ZNF346
GeneCard	APOF
GeneCard	MAGEA3
GeneCard	NFYA
GeneCard	CMKLR2
GeneCard	DACH1
GeneCard	SLC36A1
GeneCard	ANP32A
GeneCard	MFAP4
GeneCard	CLSTN2
GeneCard	SORBS2
GeneCard	TANK
GeneCard	NRBP1
GeneCard	SCAF11
GeneCard	CAMTA2
GeneCard	RBM39
GeneCard	MSBP2
GeneCard	IK
GeneCard	ZNF276
GeneCard	ITPK1
GeneCard	TEX2
GeneCard	MTMR4
GeneCard	CHRNA10
GeneCard	TSSC4
GeneCard	RPL41
GeneCard	TSPAN9
GeneCard	MYH13
GeneCard	MTCH2
GeneCard	H1-1
GeneCard	CRLF3
GeneCard	PDIA2
GeneCard	RASSF4
GeneCard	ABCA13
GeneCard	BCLAF1
GeneCard	MBD1
GeneCard	MTERF4
GeneCard	CDK5R2
GeneCard	TRAPPC6A
GeneCard	NNAT
GeneCard	GAL3ST1
GeneCard	CHDH
GeneCard	FZD10
GeneCard	RINT1
GeneCard	KCNC4
GeneCard	ACTR3
GeneCard	KRR1
GeneCard	COX17
GeneCard	POC1A

GeneCard	LUC7L
GeneCard	SH2D4B
GeneCard	HSPB11
GeneCard	SLC48A1
GeneCard	AGFG2
GeneCard	NET1
GeneCard	SWAP70
GeneCard	DYRK2
GeneCard	ZFYVE9
GeneCard	LPIN3
GeneCard	SMU1
GeneCard	MYO1G
GeneCard	SAMD5
GeneCard	PLXNA2
GeneCard	DIO1
GeneCard	ID3
GeneCard	RBL2
GeneCard	GPR141
GeneCard	RBMS2
GeneCard	ITM2A
GeneCard	RBM3
GeneCard	ADCYAP1R1
GeneCard	NIP7
GeneCard	PIP5K1A
GeneCard	ENSG00000273516
GeneCard	PCNX1
GeneCard	C17orf67
GeneCard	MTERF1
GeneCard	CRMP1
GeneCard	PHACTR2
GeneCard	MRPS31
GeneCard	API5
GeneCard	BLOC1S2
GeneCard	ALDH16A1
GeneCard	VEZF1
GeneCard	MYG1
GeneCard	FAM50A
GeneCard	MAP4K4
GeneCard	DPCD
GeneCard	ZWINT
GeneCard	SPINT1
GeneCard	RHOBTB1
GeneCard	SULT4A1
GeneCard	SEPTIN6
GeneCard	APLF
GeneCard	MATK
GeneCard	WIF1
GeneCard	LOC642361
GeneCard	BIRC7
GeneCard	MRPL11
GeneCard	PCOLCE
GeneCard	LINC00917
GeneCard	SDCBP
GeneCard	SMOX
GeneCard	TRIM4
GeneCard	CPXM2
GeneCard	MRPS26
GeneCard	TCERG1L

GeneCard	MIR3176
GeneCard	PTP4A1
GeneCard	NFATC4
GeneCard	LIPC-AS1
GeneCard	GPR50
GeneCard	LONRF3
GeneCard	UVRAG
GeneCard	USP30
GeneCard	HTR1E
GeneCard	AP1G1
GeneCard	PTP4A2
GeneCard	SAFB
GeneCard	TSPAN13
GeneCard	PEG10
GeneCard	CHMP4A
GeneCard	CKAP4
GeneCard	ENSA
GeneCard	IMPDH2
GeneCard	MDGA2
GeneCard	SLC2A8
GeneCard	AAMP
GeneCard	NOP53
GeneCard	CCDC141
GeneCard	DUS3L
GeneCard	NDUFA4L2
GeneCard	TSPAN32
GeneCard	SNRPA1
GeneCard	RNPS1
GeneCard	TTC5
GeneCard	ACLY
GeneCard	RPL17
GeneCard	TLE3
GeneCard	HSPA12A
GeneCard	H3-4
GeneCard	ADAM30
GeneCard	SFSWAP
GeneCard	PHYKPL
GeneCard	DYNLRB2
GeneCard	CD53
GeneCard	PIP4P1
GeneCard	SV2C
GeneCard	TOM1L2
GeneCard	NME3
GeneCard	TOPBP1
GeneCard	ZFP36
GeneCard	POLR2I
GeneCard	AKR7L
GeneCard	UBE2J2
GeneCard	UBR3
GeneCard	PDCD6
GeneCard	TOX
GeneCard	TRIP10
GeneCard	TRAF4
GeneCard	CHRNB3
GeneCard	MPP3
GeneCard	NEK11
GeneCard	IPMK
GeneCard	SNRPB2

GeneCard	GET3
GeneCard	GPM6A
GeneCard	NOA1
GeneCard	TCEA1
GeneCard	GIMAP6
GeneCard	RTKN2
GeneCard	MORF4L1
GeneCard	MZF1
GeneCard	MGAT5
GeneCard	SMTNL2
GeneCard	FBXL5
GeneCard	CRTAC1
GeneCard	MIR124-1HG
GeneCard	RGCC
GeneCard	HINT2
GeneCard	LRIF1
GeneCard	RAD1
GeneCard	TIPIN
GeneCard	S100G
GeneCard	PDE7A
GeneCard	ARHGAP21
GeneCard	SHB
GeneCard	ZNF326
GeneCard	UTP25
GeneCard	YIPF1
GeneCard	RRBP1
GeneCard	VPS37C
GeneCard	EXOC3L4
GeneCard	RANBP1
GeneCard	IRGC
GeneCard	ATXN7L1
GeneCard	TSPOAP1
GeneCard	DNAJC1
GeneCard	DNAJA3
GeneCard	CA6
GeneCard	ATF1
GeneCard	TAGLN2
GeneCard	IFNA21
GeneCard	URI1
GeneCard	TRIM27
GeneCard	POP7
GeneCard	STRAP
GeneCard	TLE6
GeneCard	TMEM217
GeneCard	ALKBH8
GeneCard	PEDS1
GeneCard	CDCA4
GeneCard	TIMM23
GeneCard	LOC105374344
GeneCard	RNPEPL1
GeneCard	PWP1
GeneCard	FXYD6-FXYD2
GeneCard	REEP4
GeneCard	CCT4
GeneCard	KANSL2
GeneCard	LCORL
GeneCard	CLDND1
GeneCard	SRRM1

GeneCard	RFX3
GeneCard	MYL6
GeneCard	TMEM97
GeneCard	PA2G4
GeneCard	SETD7
GeneCard	UBE2D3
GeneCard	ERC1
GeneCard	MEF2C-AS1
GeneCard	RUSC1-AS1
GeneCard	WTAP
GeneCard	NUDT13
GeneCard	RPAP3
GeneCard	PPP1R35
GeneCard	NPDC1
GeneCard	CBLC
GeneCard	ATRNL1
GeneCard	GGA1
GeneCard	POLR1H
GeneCard	SIRPB1
GeneCard	IFIT5
GeneCard	ZC3HAV1
GeneCard	TRAF3IP3
GeneCard	S100A10
GeneCard	NSG2
GeneCard	HNRNPH3
GeneCard	MIR1307
GeneCard	RAI14
GeneCard	FRYL
GeneCard	CBX1
GeneCard	SPDEF
GeneCard	ZNF532
GeneCard	SGK3
GeneCard	P2RX6
GeneCard	APOBEC3A
GeneCard	HCLS1
GeneCard	TRPT1
GeneCard	CABCOCO1
GeneCard	BRD1
GeneCard	SPINK2
GeneCard	KCNIP4
GeneCard	PPP1R13B
GeneCard	GMDS
GeneCard	CASTOR3
GeneCard	USP9Y
GeneCard	LSR
GeneCard	SYT17
GeneCard	TFPT
GeneCard	P4HA1
GeneCard	YJU2
GeneCard	FAAP100
GeneCard	PARVA
GeneCard	GOLIM4
GeneCard	TRIM44
GeneCard	ALDOC
GeneCard	EFS
GeneCard	SHANK1
GeneCard	GRID2IP
GeneCard	YTHDF2

GeneCard	RGS14
GeneCard	MED31
GeneCard	DDX19B
GeneCard	SH3BP5
GeneCard	SMC4
GeneCard	ABCA8
GeneCard	PKN1
GeneCard	TPD52L2
GeneCard	MNS1
GeneCard	SUPT7L
GeneCard	PRPF40A
GeneCard	SNAPIN
GeneCard	LYAR
GeneCard	RPP38
GeneCard	PRPF38A
GeneCard	GPN1
GeneCard	CEP76
GeneCard	CACNA2D3
GeneCard	RPUSD3
GeneCard	BAZ2A
GeneCard	PPP4R3A
GeneCard	SRRM2
GeneCard	SCN7A
GeneCard	CBFA2T2
GeneCard	ERCC6L
GeneCard	CINP
GeneCard	CNOT8
GeneCard	C1QTNF3
GeneCard	EIF3I
GeneCard	EDEM1
GeneCard	LINC00461
GeneCard	KIF20B
GeneCard	SUB1
GeneCard	C12orf75
GeneCard	COX7B2
GeneCard	PACSIN1
GeneCard	SENP1
GeneCard	UTS2B
GeneCard	ACAD10
GeneCard	ACAA2
GeneCard	ATG2B
GeneCard	LZTS2
GeneCard	ABLIM1
GeneCard	FXYD7
GeneCard	MAP3K14
GeneCard	C14orf39
GeneCard	SFMBT1
GeneCard	PDE8A
GeneCard	PHF14
GeneCard	MCPH1-AS1
GeneCard	SPON2
GeneCard	CCDC33
GeneCard	SPAG7
GeneCard	ZNF248
GeneCard	EXOC5
GeneCard	OLFM4
GeneCard	NAA25
GeneCard	ELOVL2

GeneCard	EFHD1
GeneCard	NDRG3
GeneCard	GPANK1
GeneCard	KSR2
GeneCard	OSTN
GeneCard	USP11
GeneCard	PUM3
GeneCard	GABPA
GeneCard	PTPRE
GeneCard	ERO1A
GeneCard	C5orf64
GeneCard	EP400P1
GeneCard	SEC24A
GeneCard	GPRC6A
GeneCard	HACD3
GeneCard	S100A5
GeneCard	REEP3
GeneCard	ANKS1A
GeneCard	KNOP1
GeneCard	DNAJA2
GeneCard	WDR46
GeneCard	B4GALNT2
GeneCard	KPNA3
GeneCard	ZNF703
GeneCard	SGTB
GeneCard	PLPP4
GeneCard	SVEP1
GeneCard	TMOD4
GeneCard	ATE1
GeneCard	PTPRS
GeneCard	DUSP3
GeneCard	ASH2L
GeneCard	MNAT1
GeneCard	KCNA10
GeneCard	CCZ1B
GeneCard	ZNF22
GeneCard	OAS2
GeneCard	CNDP2
GeneCard	TFIP11
GeneCard	MIR887
GeneCard	TRMT2A
GeneCard	SUMO2
GeneCard	HLA-DRB6
GeneCard	ATP1B2
GeneCard	AMIGO2
GeneCard	EDIL3
GeneCard	MYRIP
GeneCard	MIR760
GeneCard	YTHDF1
GeneCard	H3C12
GeneCard	MRPS30
GeneCard	SRPRB
GeneCard	SMURF1
GeneCard	SAFB2
GeneCard	EXOSC4
GeneCard	CCDC120
GeneCard	NKRF
GeneCard	TEN1

GeneCard	FNBP4
GeneCard	MARCHF5
GeneCard	ESRP1
GeneCard	APOBEC2
GeneCard	RFC5
GeneCard	ZNF225
GeneCard	USP35
GeneCard	RSL1D1
GeneCard	MRPS12
GeneCard	MAP6
GeneCard	PGD
GeneCard	HDLBP
GeneCard	CPSF1
GeneCard	LBHD1
GeneCard	YTHDC1
GeneCard	KIF2C
GeneCard	HAUS3
GeneCard	BRMS1
GeneCard	LAIR1
GeneCard	TMEM94
GeneCard	ZYG11B
GeneCard	FAF2
GeneCard	CALU
GeneCard	UFC1
GeneCard	PPIH
GeneCard	GPR78
GeneCard	NECAB1
GeneCard	USO1
GeneCard	MIR190A
GeneCard	EPB41L2
GeneCard	ADGRL2
GeneCard	STARD4
GeneCard	TRDMT1
GeneCard	ASIP
GeneCard	SLC7A11
GeneCard	TTK
GeneCard	PRAM1
GeneCard	SLC38A10
GeneCard	EBF1
GeneCard	FAM3C
GeneCard	PRKAG1
GeneCard	CAMK1
GeneCard	H3C2
GeneCard	SCNM1
GeneCard	HECTD2
GeneCard	FAM210B
GeneCard	MRPS9
GeneCard	ASGR1
GeneCard	OSBPL3
GeneCard	CYYR1
GeneCard	SLC30A5
GeneCard	MRPL33
GeneCard	RPA2
GeneCard	RCN2
GeneCard	RBBP6
GeneCard	SIRT7
GeneCard	NACA
GeneCard	CSNK2A3

GeneCard	NMRK2
GeneCard	IQCK
GeneCard	ATP9A
GeneCard	SANBR
GeneCard	CSRP1
GeneCard	CAPZA1
GeneCard	ADAP1
GeneCard	ZNF207
GeneCard	EIF5B
GeneCard	ZNF221
GeneCard	SUV39H1
GeneCard	KAZALD1
GeneCard	FBXO2
GeneCard	VAMP3
GeneCard	MIR572
GeneCard	MMS19
GeneCard	CSTF2
GeneCard	TXNRD3
GeneCard	IP6K3
GeneCard	NAPG
GeneCard	DHX36
GeneCard	RPS19P6
GeneCard	MGST3
GeneCard	PINX1
GeneCard	FRS3
GeneCard	UBA52P1
GeneCard	CHST8
GeneCard	PTPRM
GeneCard	DPP7
GeneCard	CYTH1
GeneCard	H2AZ1
GeneCard	CCT3
GeneCard	VPS52
GeneCard	GNL2
GeneCard	TRADD
GeneCard	FOXQ1
GeneCard	ZKSCAN8
GeneCard	ME2
GeneCard	KCTD5
GeneCard	AGR3
GeneCard	CASC3
GeneCard	ETF1
GeneCard	GLIPR1
GeneCard	MIR9-3HG
GeneCard	MIS12
GeneCard	CSE1L
GeneCard	ST8SIA1
GeneCard	BCL2L13
GeneCard	NFYB
GeneCard	SYT6
GeneCard	EIF4A1
GeneCard	SLC8A2
GeneCard	CUL5
GeneCard	AZIN1
GeneCard	BCL7C
GeneCard	EIF3H
GeneCard	C2orf49
GeneCard	ING3

GeneCard	MARCHF3
GeneCard	EBNA1BP2
GeneCard	HNRNPAB
GeneCard	CDH6
GeneCard	ZNF133
GeneCard	DIMT1
GeneCard	NSUN4
GeneCard	MBD2
GeneCard	C4orf48
GeneCard	TCF15
GeneCard	MAT2B
GeneCard	HGFAC
GeneCard	GLS2
GeneCard	DDX50
GeneCard	CCNB3
GeneCard	TASP1
GeneCard	PLEKHF2
GeneCard	ERG28
GeneCard	GNL3
GeneCard	PRIM2
GeneCard	SWSAP1
GeneCard	R3HDM1
GeneCard	R3HCC1L
GeneCard	DHX58
GeneCard	EYA3
GeneCard	GNA12
GeneCard	ILF2
GeneCard	DAOA-AS1
GeneCard	BEND7
GeneCard	KRTAP11-1
GeneCard	PRS
GeneCard	NAA60
GeneCard	EPN2
GeneCard	BIRC6
GeneCard	PLAAT4
GeneCard	AKR7A2
GeneCard	PPFIBP2
GeneCard	BTAF1
GeneCard	AFAP1L2
GeneCard	LPAR3
GeneCard	ZNF112
GeneCard	ERICH2
GeneCard	OCIAD1
GeneCard	PLPP3
GeneCard	KNDC1
GeneCard	ORC3
GeneCard	TSPO2
GeneCard	HLA-DQB1-AS1
GeneCard	GPKOW
GeneCard	RBM19
GeneCard	TSPAN16
GeneCard	PES1
GeneCard	RNPEP
GeneCard	ANGPTL2
GeneCard	MRPL4
GeneCard	OTOL1
GeneCard	MND1
GeneCard	PTGER1

GeneCard	GTF3C6
GeneCard	LSG1
GeneCard	LILRA3
GeneCard	TMEM26
GeneCard	MAL2
GeneCard	EIF5
GeneCard	C19orf33
GeneCard	PTPMT1
GeneCard	HHIPL1
GeneCard	POLR3D
GeneCard	COTL1
GeneCard	HNF4G
GeneCard	MEAF6
GeneCard	MACROH2A2
GeneCard	SYNGR3
GeneCard	NPFFR1
GeneCard	CETN3
GeneCard	STOML1
GeneCard	SLTM
GeneCard	CCIN
GeneCard	MT1A
GeneCard	PROX1-AS1
GeneCard	GCA
GeneCard	SLC38A2
GeneCard	PUS7L
GeneCard	CHAF1B
GeneCard	BECN2
GeneCard	ANXA4
GeneCard	NXT2
GeneCard	CCDC59
GeneCard	GIGYF1
GeneCard	EXTL2
GeneCard	AQP8
GeneCard	OLFM1
GeneCard	GSTA3
GeneCard	RRP12
GeneCard	ZBTB40
GeneCard	NAF1
GeneCard	UCHL5
GeneCard	AK3
GeneCard	MYNN
GeneCard	RCC2
GeneCard	ZNF283
GeneCard	BRI3
GeneCard	CDC42BPA
GeneCard	NUDT21
GeneCard	CCDC167
GeneCard	CLEC4A
GeneCard	LIMA1
GeneCard	HSF2
GeneCard	H2BC13
GeneCard	CMTR1
GeneCard	SLC39A6
GeneCard	PTPRR
GeneCard	FBXO24
GeneCard	BEST2
GeneCard	TIGAR
GeneCard	SLC1A6

GeneCard	INPP1
GeneCard	ITIH5
GeneCard	MSS51
GeneCard	NUPR1
GeneCard	SOCS5
GeneCard	NOC4L
GeneCard	LIX1
GeneCard	SOCS4
GeneCard	SCD5
GeneCard	MTRNR2L9
GeneCard	RALB
GeneCard	SRP9
GeneCard	CCNL1
GeneCard	TM9SF3
GeneCard	STAC
GeneCard	SPRY4-AS1
GeneCard	TOMM40L
GeneCard	CA5A
GeneCard	TRAK2
GeneCard	ZSWIM8
GeneCard	PRELID1
GeneCard	NIPSNAP1
GeneCard	MIER2
GeneCard	SOD2-OT1
GeneCard	LARP1
GeneCard	DDX52
GeneCard	SPC25
GeneCard	PLBD2
GeneCard	GNGT2
GeneCard	ECI2
GeneCard	USP54
GeneCard	GDNF-AS1
GeneCard	PNLIPRP1
GeneCard	SARNP
GeneCard	CCDC47
GeneCard	IPO7
GeneCard	RHOG
GeneCard	MRI1
GeneCard	HYI
GeneCard	MPV17L
GeneCard	TMOD3
GeneCard	ZNF655
GeneCard	CBR3
GeneCard	CREBZF
GeneCard	TMX3
GeneCard	FBXL7
GeneCard	PCYT1B
GeneCard	CFAP46
GeneCard	GMPS
GeneCard	TP53BP2
GeneCard	PIFO
GeneCard	LINC01184
GeneCard	PGAM5P1
GeneCard	PPIAP59
GeneCard	CCDC61
GeneCard	KRT18P16
GeneCard	PDZD2
GeneCard	RNFT2

GeneCard	PDK1
GeneCard	LTV1
GeneCard	POLR1G
GeneCard	HMGCS1
GeneCard	PHPT1
GeneCard	HS1BP3
GeneCard	MTERF3
GeneCard	LLPH
GeneCard	PTPN4
GeneCard	PCDH9
GeneCard	CKMT1A
GeneCard	COPS3
GeneCard	LRRC37A3
GeneCard	MTRNR2L1
GeneCard	LINC01492
GeneCard	SEMA7A
GeneCard	BAZ1A
GeneCard	GGT3P
GeneCard	TAFA4
GeneCard	LRCH4
GeneCard	ZC3HC1
GeneCard	TSC22D4
GeneCard	GADD45GIP1
GeneCard	PFDN4
GeneCard	ZNF230
GeneCard	EFNA1
GeneCard	WDR82
GeneCard	UQCRHL
GeneCard	ULK2
GeneCard	RSRP1
GeneCard	SPC24
GeneCard	STRIP1
GeneCard	ATP5MG
GeneCard	MDGA1
GeneCard	NPTXR
GeneCard	MIR329-1
GeneCard	SLC22A8
GeneCard	CPNE1
GeneCard	EPS15L1
GeneCard	ADAM28
GeneCard	TMED9
GeneCard	NMUR1
GeneCard	VRK3
GeneCard	UTP11
GeneCard	PHF5A
GeneCard	ZNF638
GeneCard	HS3ST2
GeneCard	TMEM47
GeneCard	CSAG1
GeneCard	GRSF1
GeneCard	TOX2
GeneCard	IFNLR1
GeneCard	PDZD8
GeneCard	SGIP1
GeneCard	AMOT
GeneCard	PPP6R1
GeneCard	MIR329-2
GeneCard	COX6B2

GeneCard	SLCO3A1
GeneCard	SELENOW
GeneCard	KRTAP10-7
GeneCard	EIF2A
GeneCard	USP39
GeneCard	WDR91
GeneCard	PSD2
GeneCard	NMNAT3
GeneCard	NUP50
GeneCard	TXNDC11
GeneCard	RSBN1L
GeneCard	PARD3B
GeneCard	CARD10
GeneCard	TNNC2
GeneCard	POU4F2
GeneCard	BCAS2
GeneCard	THAP4
GeneCard	FICD
GeneCard	GNG2
GeneCard	METTL25B
GeneCard	KLF7
GeneCard	CST8
GeneCard	PPA1
GeneCard	RTCB
GeneCard	EPS8L2
GeneCard	SIGLECL1
GeneCard	CST2
GeneCard	HAUS6
GeneCard	SOCS6
GeneCard	FBLN2
GeneCard	TRIM40
GeneCard	PDE1A
GeneCard	IPO9
GeneCard	HMCES
GeneCard	ZNF165
GeneCard	UNC5B
GeneCard	ACOT2
GeneCard	TPRA1
GeneCard	FAM168B
GeneCard	LUC7L3
GeneCard	DCTPP1
GeneCard	CRISPLD2
GeneCard	CHORDC1
GeneCard	GTF3C1
GeneCard	CSNK1G1
GeneCard	LRRC2
GeneCard	CCNA1
GeneCard	CCDC7
GeneCard	STARD3
GeneCard	MTRNR2L3
GeneCard	ODAM
GeneCard	RBM14
GeneCard	HLA-H
GeneCard	LILRB3
GeneCard	WDR18
GeneCard	CTIF
GeneCard	MIR19B2
GeneCard	PSME1

GeneCard	KCNAB1
GeneCard	DUT
GeneCard	AGBL2
GeneCard	DNAJB4
GeneCard	THSD7B
GeneCard	CMIP
GeneCard	TTC29
GeneCard	B4GALT6
GeneCard	H3C3
GeneCard	POGK
GeneCard	SPATA2
GeneCard	AKAP5
GeneCard	CUL4A
GeneCard	LRRC37A
GeneCard	MTSS2
GeneCard	DDX39A
GeneCard	NPM2
GeneCard	LINC01725
GeneCard	NME4
GeneCard	LINC00423
GeneCard	CTAGE12P
GeneCard	ZNF296
GeneCard	DNAJC9
GeneCard	EGFL8
GeneCard	CWF19L2
GeneCard	ZBTB4
GeneCard	CALML6
GeneCard	ZNF77
GeneCard	RCN1
GeneCard	SMDT1
GeneCard	SYF2
GeneCard	CFAP20
GeneCard	ROPN1L
GeneCard	TMX1
GeneCard	USP36
GeneCard	USP13
GeneCard	JAKMIP3
GeneCard	PFDN2
GeneCard	ESPL1
GeneCard	RUFY3
GeneCard	MTRES1
GeneCard	LOC101927870
GeneCard	HOXC4
GeneCard	SNX7
GeneCard	MTRNR2L2
GeneCard	CMPK1
GeneCard	KLHL12
GeneCard	FXYD6
GeneCard	THAP3
GeneCard	EIF3G
GeneCard	EFHD2
GeneCard	OLA1
GeneCard	PCYT2
GeneCard	SGCZ
GeneCard	CAAP1
GeneCard	TTLL6
GeneCard	CTAGE1
GeneCard	FBXO3

GeneCard	AKR1B10
GeneCard	CCDC25
GeneCard	CCT2
GeneCard	LRRC20
GeneCard	lnc-ZNF296-6
GeneCard	NONHSAG026010.2
GeneCard	DBF4
GeneCard	H3C11
GeneCard	FRAT2
GeneCard	IFNA8
GeneCard	SIGIRR
GeneCard	UNC5CL
GeneCard	H3C4
GeneCard	COPS7B
GeneCard	STOML2
GeneCard	GRWD1
GeneCard	BOD1
GeneCard	FCMR
GeneCard	CFAP74
GeneCard	ZCCHC24
GeneCard	RASGRP3
GeneCard	ARHGDIB
GeneCard	HOMER3
GeneCard	SPOCK2
GeneCard	RNASE1
GeneCard	SYPL1
GeneCard	SLC16A4
GeneCard	RNF214
GeneCard	NSFL1C
GeneCard	PANX2
GeneCard	DHRSX
GeneCard	TAF1C
GeneCard	DCTN6
GeneCard	CCDC14
GeneCard	DEUP1
GeneCard	SCGN
GeneCard	OLIG3
GeneCard	NIT1
GeneCard	THAP7
GeneCard	EHF
GeneCard	STAC2
GeneCard	DGKZ
GeneCard	PRRC2B
GeneCard	RTF1
GeneCard	SRP14
GeneCard	BLOC1S4
GeneCard	VSIR
GeneCard	CDH19
GeneCard	ZADH2
GeneCard	LXN
GeneCard	ZNF404
GeneCard	MTA2
GeneCard	SCAMP3
GeneCard	MRPS17
GeneCard	CNPY3
GeneCard	KIF16B
GeneCard	CARNMT1
GeneCard	LHX6

GeneCard	CST1
GeneCard	CCDC116
GeneCard	GALR3
GeneCard	SNF8
GeneCard	GPS1
GeneCard	PDXP
GeneCard	AK4
GeneCard	RIPOR2
GeneCard	MRPL19
GeneCard	SCGB2A2
GeneCard	SLC29A2
GeneCard	PCDH7
GeneCard	HIRIP3
GeneCard	PRPF38B
GeneCard	RBM5
GeneCard	PARVG
GeneCard	PTK6
GeneCard	CLNK
GeneCard	CHTOP
GeneCard	KLF12
GeneCard	TBC1D9
GeneCard	AKAP8L
GeneCard	EML3
GeneCard	PMCH
GeneCard	HLF
GeneCard	KLHL20
GeneCard	CDKN2A-DT
GeneCard	CR1L
GeneCard	ZNF232
GeneCard	RNF138
GeneCard	TAS1R2
GeneCard	COPS4
GeneCard	AKAP8
GeneCard	CYP27C1
GeneCard	RNF20
GeneCard	SUN5
GeneCard	SELENOM
GeneCard	ZNF239
GeneCard	PRPF19
GeneCard	RFC4
GeneCard	NRSN1
GeneCard	C11orf49
GeneCard	PARP4
GeneCard	LURAP1L
GeneCard	ARHGAP19
GeneCard	COPS6
GeneCard	ARAP1
GeneCard	PIM2
GeneCard	TRAPPC3
GeneCard	CACNB1
GeneCard	PAK1IP1
GeneCard	RPF2
GeneCard	ENY2
GeneCard	PAIP1
GeneCard	MIR1285-2
GeneCard	TNFAIP8
GeneCard	LRRC59
GeneCard	FAM163A

GeneCard	CDC16
GeneCard	LSM1
GeneCard	B4GALT3
GeneCard	MZT2A
GeneCard	GALNT4
GeneCard	H1-3
GeneCard	ERP29
GeneCard	EIF4EBP2
GeneCard	SNRPF
GeneCard	ARHGAP30
GeneCard	NRDC
GeneCard	GPATCH8
GeneCard	CDR2L
GeneCard	ZNF740
GeneCard	TRIM56
GeneCard	TARDBPP3
GeneCard	STIN2-VNTR
GeneCard	ANKMY1
GeneCard	FAHD2A
GeneCard	IFNA5
GeneCard	DAPK2
GeneCard	ARL6IP6
GeneCard	ERGIC2
GeneCard	TRIR
GeneCard	MRPL20
GeneCard	THRAP3
GeneCard	SIGLEC14
GeneCard	ZNF787
GeneCard	RRP8
GeneCard	ZNF3
GeneCard	MKRN2
GeneCard	SLBP
GeneCard	CSTF2T
GeneCard	C1orf112
GeneCard	MANF
GeneCard	DAZAP1
GeneCard	RNF152
GeneCard	MTRNR2L13
GeneCard	C11orf68
GeneCard	SETD3
GeneCard	GATB
GeneCard	BRK1
GeneCard	MRPS35
GeneCard	SPARCL1
GeneCard	GPR17
GeneCard	RPP25
GeneCard	EID1
GeneCard	PTMS
GeneCard	NFIL3
GeneCard	RAPGEF5
GeneCard	NOP58
GeneCard	TCAF2
GeneCard	GDE1
GeneCard	RAPGEF6
GeneCard	SNX17
GeneCard	ARMC3
GeneCard	PRORP
GeneCard	CACUL1

GeneCard	MVB12A
GeneCard	FAM3B
GeneCard	EIF3M
GeneCard	FOSL2
GeneCard	TMCC1
GeneCard	SENP3
GeneCard	MTREX
GeneCard	CD300LF
GeneCard	TGFBRAP1
GeneCard	H2AC21
GeneCard	APIP
GeneCard	LLGL2
GeneCard	NAT8
GeneCard	LIMS1
GeneCard	SNAR-I
GeneCard	KCNC2
GeneCard	NCBP3
GeneCard	RTF2
GeneCard	KRTAP9-2
GeneCard	ZNF512
GeneCard	NPTN
GeneCard	ITPRIP
GeneCard	LMO4
GeneCard	ZNF227
GeneCard	PHF7
GeneCard	BIK
GeneCard	PUM2
GeneCard	RAB11FIP4
GeneCard	SDF2L1
GeneCard	FAM222A
GeneCard	GGACT
GeneCard	PAK6
GeneCard	XPO5
GeneCard	MAPK1IP1L
GeneCard	NME1-NME2
GeneCard	DDX31
GeneCard	SPPL3
GeneCard	HERC4
GeneCard	UBE2U
GeneCard	PPIC
GeneCard	SNRK
GeneCard	S100A3
GeneCard	BOC
GeneCard	ANXA8
GeneCard	KLRG1
GeneCard	CAPS
GeneCard	PHF12
GeneCard	RPAIN
GeneCard	LINC01098
GeneCard	LPAL2
GeneCard	CLVS1
GeneCard	UTP14A
GeneCard	MRPS21
GeneCard	ZNF223
GeneCard	RALY
GeneCard	SHC3
GeneCard	MTFP1
GeneCard	MED16

GeneCard	FOXN2
GeneCard	SNW1
GeneCard	EIF4A2
GeneCard	BOLA1
GeneCard	RBM6
GeneCard	CLASP2
GeneCard	ECHDC1
GeneCard	TWF2
GeneCard	ZDHHC16
GeneCard	OTUB1
GeneCard	GAL3ST4
GeneCard	CPNE4
GeneCard	SMIM8
GeneCard	RPS6KB2
GeneCard	PRPF4B
GeneCard	RBMXL1
GeneCard	TAF7L
GeneCard	GPR89B
GeneCard	ZNF488
GeneCard	SMG6
GeneCard	CBFA2T3
GeneCard	CPSF7
GeneCard	COMTD1
GeneCard	FHIP2B
GeneCard	UPF3A
GeneCard	TBCC
GeneCard	PHYHIPL
GeneCard	BET1L
GeneCard	INTS3
GeneCard	FOXK2
GeneCard	SEZ6L
GeneCard	SLC24A3
GeneCard	SLC25A48
GeneCard	C16orf95
GeneCard	LZIC
GeneCard	PPP1R2
GeneCard	RBM42
GeneCard	ACAP1
GeneCard	CHST15
GeneCard	TULP4
GeneCard	GTF2A1
GeneCard	BOLL
GeneCard	BCAS1
GeneCard	ARGLU1
GeneCard	RORA-AS1
GeneCard	RTCA
GeneCard	C1D
GeneCard	P2RY6
GeneCard	BOP1
GeneCard	RUFY1
GeneCard	CCL15
GeneCard	RFX1
GeneCard	MTCH1
GeneCard	MPG
GeneCard	UBL4A
GeneCard	PILRB
GeneCard	FBXO46
GeneCard	ADI1

GeneCard	DLK2
GeneCard	AVPI1
GeneCard	FCHSD1
GeneCard	BTBD16
GeneCard	FAM98A
GeneCard	TNRC6C
GeneCard	RNF126
GeneCard	SLC29A4
GeneCard	UTP20
GeneCard	SPSB2
GeneCard	ZSCAN9
GeneCard	ZKSCAN1
GeneCard	PGBD5
GeneCard	HSPB7
GeneCard	UBE2C
GeneCard	SUPT20H
GeneCard	RRAGB
GeneCard	ZCRB1
GeneCard	ARHGAP22
GeneCard	COX10-DT
GeneCard	TMEM131
GeneCard	CRYZL1
GeneCard	OMG
GeneCard	SPSB1
GeneCard	MYL12A
GeneCard	KRT33B
GeneCard	RNF41
GeneCard	TICRR
GeneCard	PBLD
GeneCard	FKBPL
GeneCard	METTL16
GeneCard	NME6
GeneCard	SRSF10
GeneCard	CPSF3
GeneCard	SLC10A4
GeneCard	MRPL21
GeneCard	L3MBTL4
GeneCard	BRMS1L
GeneCard	DCAF13
GeneCard	FNBP1L
GeneCard	EXOSC7
GeneCard	ITIH2
GeneCard	MRPL46
GeneCard	CLSTN3
GeneCard	CYLC2
GeneCard	ZC3H4
GeneCard	HP1BP3
GeneCard	GTF3C2
GeneCard	LINCR-0001
GeneCard	CEACAM19
GeneCard	KCTD12
GeneCard	NOL11
GeneCard	KIAA1586
GeneCard	TMEM183A
GeneCard	BRD9
GeneCard	CACNG5
GeneCard	SLC14A2
GeneCard	RAB31

GeneCard	MAST2
GeneCard	SRCIN1
GeneCard	CEP70
GeneCard	MRPL40
GeneCard	NAALAD2
GeneCard	CEP72
GeneCard	ECSIT
GeneCard	VASH2
GeneCard	DIDO1
GeneCard	C1orf35
GeneCard	DNAJB9
GeneCard	RGL1
GeneCard	GAS2
GeneCard	ZC3H12C
GeneCard	IRAK2
GeneCard	USP3
GeneCard	RGN
GeneCard	NUP43
GeneCard	UBE2D4
GeneCard	DNAJC10
GeneCard	CYP20A1
GeneCard	PIP5KL1
GeneCard	CDNF
GeneCard	RAP1GAP
GeneCard	LOC157273
GeneCard	STK26
GeneCard	SURF2
GeneCard	PYCR3
GeneCard	SDIM1
GeneCard	FAM114A2
GeneCard	HSD3BP4
GeneCard	DDX56
GeneCard	ACYP2
GeneCard	ZCCHC17
GeneCard	RUFY2
GeneCard	BMP3
GeneCard	MICAL2
GeneCard	LCA5L
GeneCard	STX12
GeneCard	ADAT2
GeneCard	SRSF8
GeneCard	OOSP2
GeneCard	MRPS5
GeneCard	ZCCHC10
GeneCard	PELI1
GeneCard	OTX1
GeneCard	A2MP1
GeneCard	ZBTB10
GeneCard	ECH1
GeneCard	GNG11
GeneCard	TMX2
GeneCard	EXOSC6
GeneCard	IGSF8
GeneCard	DHX33
GeneCard	LARP4B
GeneCard	MPHOSPH10
GeneCard	ENOPH1
GeneCard	NECAB3

GeneCard	MRM3
GeneCard	BORCS5
GeneCard	CPSF2
GeneCard	CFAP58
GeneCard	SESTD1
GeneCard	CCSER2
GeneCard	SNPH
GeneCard	CYTH2
GeneCard	H2BC11
GeneCard	EFNB3
GeneCard	HIVEP3
GeneCard	ARHGEF5
GeneCard	MBOAT1
GeneCard	DCAF1
GeneCard	THAP11
GeneCard	SNTG1
GeneCard	TVP23B
GeneCard	ZNF629
GeneCard	CBARP
GeneCard	WDR70
GeneCard	MYBPC2
GeneCard	DAZAP2
GeneCard	WDR77
GeneCard	USP14
GeneCard	MRPL55
GeneCard	MRPL17
GeneCard	NUDT4
GeneCard	ARID3B
GeneCard	USP12
GeneCard	CAPSL
GeneCard	CNPY2
GeneCard	OSBP
GeneCard	HNRNPLL
GeneCard	ZNF33B
GeneCard	TMEM176A
GeneCard	MYLIP
GeneCard	RBBP9
GeneCard	ANGPTL7
GeneCard	IPO4
GeneCard	POLR3F
GeneCard	ARF5
GeneCard	REG1B
GeneCard	CYP39A1
GeneCard	MIR8085
GeneCard	CA14
GeneCard	RBM15B
GeneCard	CHMP2A
GeneCard	SLC2A12
GeneCard	PIR
GeneCard	UPP2
GeneCard	MTRNR2L7
GeneCard	APOC4-APOC2
GeneCard	SCML4
GeneCard	RIOK2
GeneCard	PAN2
GeneCard	LRRC7
GeneCard	GEN1
GeneCard	NCKAP5

GeneCard	ZNF581
GeneCard	NFRKB
GeneCard	LOC111589215
GeneCard	SNRPG
GeneCard	ZNF284
GeneCard	RIOX2
GeneCard	ZNF222
GeneCard	MTG1
GeneCard	CLDN8
GeneCard	HCST
GeneCard	KCNK12
GeneCard	PHLDA1
GeneCard	PPAN
GeneCard	DOC2A
GeneCard	SNX25
GeneCard	ZBTB9
GeneCard	CBR4
GeneCard	PNLIPRP2
GeneCard	C3orf62
GeneCard	H3C10
GeneCard	TAS2R60
GeneCard	MIR5582
GeneCard	WDR33
GeneCard	SLC23A2
GeneCard	COLGALT2
GeneCard	CRACR2B
GeneCard	PPIAP55
GeneCard	TERB2
GeneCard	GMNC
GeneCard	NUTF2
GeneCard	EIF1
GeneCard	GORASP2
GeneCard	ITGB3BP
GeneCard	SSBP3
GeneCard	APOBEC3C
GeneCard	CLYBL
GeneCard	ADSS2
GeneCard	POLR1E
GeneCard	MRPL15
GeneCard	ARMCX5-GPRASP2
GeneCard	SNORD63
GeneCard	TMEM259
GeneCard	TWF1
GeneCard	XPO7
GeneCard	RPSAP52
GeneCard	ACTL7B
GeneCard	ARHGAP12
GeneCard	WASH3P
GeneCard	NME5
GeneCard	STK16
GeneCard	FZR1
GeneCard	NUP58
GeneCard	ERH
GeneCard	TRIP6
GeneCard	AHCYL2
GeneCard	HYPK
GeneCard	CCDC134
GeneCard	BOLA2

GeneCard	C9orf78
GeneCard	ZNF30
GeneCard	DMKN
GeneCard	ODF2
GeneCard	RBM26
GeneCard	ARFIP2
GeneCard	FRMPD2
GeneCard	CPNE3
GeneCard	PIH1D1
GeneCard	DDX25
GeneCard	NOMO1
GeneCard	ANKRD36B
GeneCard	TRAM1
GeneCard	VSIG4
GeneCard	TSKU
GeneCard	CDCA3
GeneCard	EHD2
GeneCard	GDF10
GeneCard	MRPS24
GeneCard	RNF103
GeneCard	STC2
GeneCard	ZNF410
GeneCard	SLC4A10
GeneCard	GTF3C3
GeneCard	CMSS1
GeneCard	PAFAH1B3
GeneCard	RAB6C
GeneCard	TEF
GeneCard	IFNW1
GeneCard	APOBEC4
GeneCard	NTN4
GeneCard	KLHL8
GeneCard	TRAPPC5
GeneCard	RASA3
GeneCard	NIFK
GeneCard	LRRFIP2
GeneCard	TIPARP
GeneCard	NFATC3
GeneCard	EPOP
GeneCard	THEM4
GeneCard	CHCHD3
GeneCard	C3orf20
GeneCard	ATP5IF1
GeneCard	DENND4B
GeneCard	SF3A3
GeneCard	MIR520D
GeneCard	CCDC112
GeneCard	TSPOAP1-AS1
GeneCard	STARD13
GeneCard	TSPAN14
GeneCard	PLA2G4E
GeneCard	GTPBP10
GeneCard	TCL1B
GeneCard	CNN2
GeneCard	BEND6
GeneCard	GRAMD1A
GeneCard	DRAP1
GeneCard	AVEN

GeneCard	TCF7L1
GeneCard	CACTIN
GeneCard	REXO4
GeneCard	DDX46
GeneCard	KLHL22
GeneCard	CHI3L2
GeneCard	MRPL47
GeneCard	C7orf25
GeneCard	ZC3H13
GeneCard	MRPL49
GeneCard	WDR5
GeneCard	PRSS45P
GeneCard	ZNF438
GeneCard	ODF3L2
GeneCard	CRACR2A
GeneCard	NUDT5
GeneCard	LMF2
GeneCard	TMSB10
GeneCard	RTP3
GeneCard	ZMYM5
GeneCard	FAM117B
GeneCard	LARP6
GeneCard	TRUB2
GeneCard	CKMT1B
GeneCard	CSTF3
GeneCard	TMPRSS11E
GeneCard	SDAD1
GeneCard	MAGED1
GeneCard	MTCP1
GeneCard	PYROXD2
GeneCard	METAP2
GeneCard	ZNF226
GeneCard	ACTBL2
GeneCard	HOXA5
GeneCard	WDR83
GeneCard	PJA2
GeneCard	FERD3L
GeneCard	SPAG9
GeneCard	SAMD8
GeneCard	MOCS3
GeneCard	THNSL1
GeneCard	DTL
GeneCard	PYGO1
GeneCard	TM9SF2
GeneCard	FAM209A
GeneCard	DHX57
GeneCard	C1orf87
GeneCard	ZW10
GeneCard	PIAS3
GeneCard	MRPS18B
GeneCard	OLFM3
GeneCard	RNF40
GeneCard	GPR180
GeneCard	EEF1AKMT2
GeneCard	MRPL13
GeneCard	CDK17
GeneCard	C7orf61
GeneCard	TMEM255A

GeneCard	TSC22D1
GeneCard	ZFYVE16
GeneCard	RAMP3
GeneCard	NIPSNAP2
GeneCard	THOC7
GeneCard	FRMD4B
GeneCard	ZNF599
GeneCard	ANKRD52
GeneCard	ARMC1
GeneCard	DELE1
GeneCard	HAUS8
GeneCard	AKR7A3
GeneCard	NAPA
GeneCard	STEAP1B
GeneCard	STOML3
GeneCard	CPEB3
GeneCard	ST3GAL6
GeneCard	HTATSF1
GeneCard	DDX10
GeneCard	PCNP
GeneCard	UHMK1
GeneCard	PPM1A
GeneCard	VNN2
GeneCard	MIR663A
GeneCard	MXD1
GeneCard	RCL1
GeneCard	SNX15
GeneCard	PUSL1
GeneCard	ACOT13
GeneCard	SNX33
GeneCard	ARPP19
GeneCard	ADGRL4
GeneCard	SNX18
GeneCard	TBC1D2
GeneCard	FOXR1
GeneCard	EAF1
GeneCard	TAAR6
GeneCard	RFLNA
GeneCard	COPRS
GeneCard	KAT14
GeneCard	RTKN
GeneCard	FBXL13
GeneCard	DHX15
GeneCard	CYSTM1
GeneCard	FOXA3
GeneCard	BANF2
GeneCard	BAHD1
GeneCard	LINC01185
GeneCard	GRAP
GeneCard	PPM1G
GeneCard	DIXDC1
GeneCard	ZZEF1
GeneCard	LAMTOR4
GeneCard	RUFY4
GeneCard	MRPL24
GeneCard	FAM184A
GeneCard	STK33
GeneCard	LARP4

GeneCard	EIF4ENIF1
GeneCard	GPR156
GeneCard	ZC3H18
GeneCard	MPC2
GeneCard	AASDHPPT
GeneCard	CNIH3
GeneCard	MIIP
GeneCard	NIPSNAP3B
GeneCard	RRP1
GeneCard	GPR18
GeneCard	S100P
GeneCard	TNIP3
GeneCard	SCAMP4
GeneCard	CENPM
GeneCard	SKAP1
GeneCard	NOMO3
GeneCard	MAP3K15
GeneCard	SLAIN2
GeneCard	TMA16
GeneCard	CD99L2
GeneCard	TBCA
GeneCard	ZMAT4
GeneCard	THOC5
GeneCard	SMYD3
GeneCard	PLCD4
GeneCard	IQUB
GeneCard	MRPL38
GeneCard	DOK1
GeneCard	JMJD7
GeneCard	NAPSB
GeneCard	YTHDC2
GeneCard	STEAP3-AS1
GeneCard	CHST2
GeneCard	KCNH4
GeneCard	CD2BP2
GeneCard	SEZ6
GeneCard	MPHOSPH6
GeneCard	UBE2H
GeneCard	OSBPL11
GeneCard	BRD8
GeneCard	NPFFR2
GeneCard	CCDC83
GeneCard	PVRIG
GeneCard	PANK3
GeneCard	SLC13A2
GeneCard	GSTA2
GeneCard	MYO1F
GeneCard	FOCAD
GeneCard	ABCC5
GeneCard	ESYT1
GeneCard	EIF3L
GeneCard	GCC1
GeneCard	SUSD3
GeneCard	MARCHF8
GeneCard	MRPL18
GeneCard	ARL6IP4
GeneCard	PAPSS1
GeneCard	ZBTB14

GeneCard	FSIP1
GeneCard	NUMBL
GeneCard	ACOT9
GeneCard	TDRD1
GeneCard	C7orf50
GeneCard	CNSN
GeneCard	PLSCR1
GeneCard	HNRNPUL2
GeneCard	SLC28A2
GeneCard	MTRNR2L10
GeneCard	CDKL1
GeneCard	CCDC60
GeneCard	ANXA13
GeneCard	ZNF483
GeneCard	FECHP1
GeneCard	MEIKIN
GeneCard	KAT7
GeneCard	ENSG00000270679
GeneCard	MK280269-024
GeneCard	MXD4
GeneCard	SEC11C
GeneCard	C5orf67
GeneCard	TRMT61B
GeneCard	DOK2
GeneCard	ZFAND4
GeneCard	RALBP1
GeneCard	NAA38
GeneCard	PTGR1
GeneCard	SUZ12P1
GeneCard	FAM98C
GeneCard	LY86-AS1
GeneCard	TEPSIN
GeneCard	KCTD8
GeneCard	SLC7A2
GeneCard	SLC6A6
GeneCard	ZNF25
GeneCard	NKTR
GeneCard	ENPP6
GeneCard	ARF3
GeneCard	NOB1
GeneCard	C15orf39
GeneCard	OSBPL1A
GeneCard	ACSM6
GeneCard	ATAT1
GeneCard	RBM27
GeneCard	NLK
GeneCard	SFTA1P
GeneCard	FGD5
GeneCard	CYRIA
GeneCard	IGSF21
GeneCard	CMAS
GeneCard	ULBP3
GeneCard	ST8SIA5
GeneCard	ATP6V0D1
GeneCard	TTC23
GeneCard	ARFGAP2
GeneCard	SMC2
GeneCard	ELK3

GeneCard	BTN3A1
GeneCard	DGKI
GeneCard	ARMH3
GeneCard	PDCD5
GeneCard	PRXL2A
GeneCard	KLHL26
GeneCard	TCF25
GeneCard	PEBP4
GeneCard	SLCO2B1
GeneCard	POLA2
GeneCard	SCAANT1
GeneCard	NUDCD3
GeneCard	STAM2
GeneCard	MBLAC1
GeneCard	STRN4
GeneCard	DEFA3
GeneCard	FNTA
GeneCard	ZNF652-AS1
GeneCard	MED24
GeneCard	P2RY4
GeneCard	MED27
GeneCard	TAS2R41
GeneCard	ZBTB43
GeneCard	LOC101928135
GeneCard	EME1
GeneCard	SESN3
GeneCard	DNAJB14
GeneCard	MIR5004
GeneCard	DAPK3
GeneCard	MRPL1
GeneCard	LINC01016
GeneCard	HOOK3
GeneCard	RGL2
GeneCard	COL23A1
GeneCard	DENND2D
GeneCard	ZBTB2
GeneCard	ALPK2
GeneCard	WWP1
GeneCard	TMEM254
GeneCard	MGAT4B
GeneCard	FUBP3
GeneCard	FBXO28
GeneCard	DIRAS2
GeneCard	SFR1
GeneCard	HSF5
GeneCard	PRPF18
GeneCard	SEC61A2
GeneCard	UBAP2L
GeneCard	PITX1-AS1
GeneCard	TTC1
GeneCard	CYRIB
GeneCard	VSTM2A
GeneCard	RGSL1
GeneCard	AK8
GeneCard	LINC00520
GeneCard	MIF4GD
GeneCard	RMND5A
GeneCard	MOB1A

GeneCard	ZCCHC7
GeneCard	H2AP
GeneCard	GOLM2
GeneCard	TAFA5
GeneCard	UBA3
GeneCard	ABHD10
GeneCard	UXT
GeneCard	YTHDF3
GeneCard	INTS12
GeneCard	CNOT7
GeneCard	H3C7
GeneCard	H3C8
GeneCard	SAP30
GeneCard	KAZN
GeneCard	PDCL3
GeneCard	PDZRN4
GeneCard	SF1
GeneCard	UNK
GeneCard	SV2B
GeneCard	MED4
GeneCard	C1orf174
GeneCard	MAGEC2
GeneCard	CDC42EP2
GeneCard	MRPL52
GeneCard	GRPEL1
GeneCard	NR1D2
GeneCard	MRPL48
GeneCard	IST1
GeneCard	LINC02141
GeneCard	UBR4
GeneCard	ARRDC4
GeneCard	C1orf74
GeneCard	IGSF10
GeneCard	RBM4B
GeneCard	HEBP1
GeneCard	FAM120A
GeneCard	HSPA12B
GeneCard	SPCS3
GeneCard	ATG3
GeneCard	SMAP1
GeneCard	SCG3
GeneCard	LSM14B
GeneCard	CSNK1G2
GeneCard	DRG2
GeneCard	NLRP8
GeneCard	ZNF485
GeneCard	GSTK1
GeneCard	EIF3E
GeneCard	ANKRA2
GeneCard	GSTM5
GeneCard	PNN
GeneCard	NXF2
GeneCard	NYAP1
GeneCard	MRPL2
GeneCard	KRT78
GeneCard	LCMT1
GeneCard	EIF2D
GeneCard	SIK2

GeneCard	LSM12
GeneCard	SYNE3
GeneCard	HSFY1
GeneCard	CST7
GeneCard	ACF
GeneCard	DENND4A
GeneCard	PRTG
GeneCard	SSR1
GeneCard	KLHL36
GeneCard	LINC01550
GeneCard	TBCB
GeneCard	SIT1
GeneCard	ZFP3
GeneCard	CCDC121
GeneCard	MIR153-1
GeneCard	KBTBD8
GeneCard	NMBR
GeneCard	GPN2
GeneCard	MEP1B
GeneCard	ENSG00000285897
GeneCard	ZNF44
GeneCard	PHF19
GeneCard	JAKMIP1
GeneCard	TLE4
GeneCard	GPATCH1
GeneCard	SUDS3
GeneCard	MAP3K21
GeneCard	CNTN3
GeneCard	TUT7
GeneCard	TXNDC17
GeneCard	TNFAIP2
GeneCard	LINC00877
GeneCard	PCDH11Y
GeneCard	TBC1D22B
GeneCard	MIS18A
GeneCard	RPL23AP39
GeneCard	UGT1A12P
GeneCard	LSM8
GeneCard	GRIPAP1
GeneCard	TAX1BP1
GeneCard	GEMIN6
GeneCard	RNF2
GeneCard	DCAF10
GeneCard	SAP18
GeneCard	NXPH1
GeneCard	CENPS
GeneCard	PAK5
GeneCard	RAB26
GeneCard	CATSPERB
GeneCard	KATNA1
GeneCard	POLR3C
GeneCard	C8orf33
GeneCard	RNF25
GeneCard	CBLN3
GeneCard	FBXL2
GeneCard	ATP5MF
GeneCard	CDKN2D
GeneCard	RIPPLY1

GeneCard	UBE2L1
GeneCard	NECAP2
GeneCard	STAU2
GeneCard	SNRNP35
GeneCard	ENSG00000259605
GeneCard	lnc-HLA-DQA1-9
GeneCard	lnc-HLA-DRB1-7
GeneCard	HSALNG0049423
GeneCard	HSALNG0049424
GeneCard	ZDHHC7
GeneCard	ARHGEF15
GeneCard	GFOD1
GeneCard	EXOC3
GeneCard	RPA4
GeneCard	ZNF136
GeneCard	SSBP4
GeneCard	ZRANB2
GeneCard	ERMN
GeneCard	TIMM17B
GeneCard	RALGDS
GeneCard	ASB6
GeneCard	CD8B
GeneCard	C11orf1
GeneCard	C1QTNF4
GeneCard	KDM1B
GeneCard	C10orf71
GeneCard	PID1
GeneCard	ANKRD10
GeneCard	TIAM2
GeneCard	GATAD2A
GeneCard	SSH3
GeneCard	OR4D2
GeneCard	TEX51
GeneCard	RNF34
GeneCard	DNAJC4
GeneCard	NUCB1
GeneCard	NRBP2
GeneCard	RAD18
GeneCard	MAGEA6
GeneCard	ZNF285
GeneCard	GSTM4
GeneCard	HIGD1A
GeneCard	SYT3
GeneCard	PPP2R2D
GeneCard	COL21A1
GeneCard	MS4A14
GeneCard	SMG5
GeneCard	MAP1A
GeneCard	PWWP3A
GeneCard	NENF
GeneCard	RETSAT
GeneCard	NKAIN3
GeneCard	MRPS11
GeneCard	NAA80
GeneCard	PNRC2
GeneCard	PLA1A
GeneCard	RNF14
GeneCard	MKNK1

GeneCard	AGO4
GeneCard	MAGI3
GeneCard	GDA
GeneCard	MARCHF10
GeneCard	HSPA4L
GeneCard	CIAPIN1
GeneCard	SYT16
GeneCard	ZNF155
GeneCard	AKIP1
GeneCard	WDR61
GeneCard	MIR4736
GeneCard	RUSC1
GeneCard	PLEKHA6
GeneCard	MTF2
GeneCard	SAP25
GeneCard	YRDC
GeneCard	EVI2A
GeneCard	HIC2
GeneCard	LTN1
GeneCard	PALM2AKAP2
GeneCard	ZNF544
GeneCard	NFX1
GeneCard	PMF1
GeneCard	STK3
GeneCard	KBTBD4
GeneCard	MTRNR2L11
GeneCard	H1-10
GeneCard	MRPL22
GeneCard	SERINC3
GeneCard	MRPL53
GeneCard	SH3BGRL2
GeneCard	MCMBP
GeneCard	ILRUN
GeneCard	DNAAF9
GeneCard	UCHL3
GeneCard	GPR83
GeneCard	COX11
GeneCard	PRRC1
GeneCard	LINC02863
GeneCard	BTBD1
GeneCard	C11orf94
GeneCard	ACRV1
GeneCard	POLR3E
GeneCard	PARP6
GeneCard	MRPL35
GeneCard	TSPAN33
GeneCard	UBE2E1
GeneCard	VKORC1L1
GeneCard	EMC4
GeneCard	MS4A12
GeneCard	C1orf105
GeneCard	ZFYVE19
GeneCard	TDG
GeneCard	TAF9B
GeneCard	ANKMY2
GeneCard	SLC8A1-AS1
GeneCard	MID1IP1
GeneCard	LAMTOR5

GeneCard	TRIML2
GeneCard	MLLT6
GeneCard	PABIR1
GeneCard	IQCH
GeneCard	PMS2P1
GeneCard	CIAO1
GeneCard	LILRA2
GeneCard	AGPAT3
GeneCard	PATE3
GeneCard	ODF2L
GeneCard	EIF4E2
GeneCard	MRPL41
GeneCard	SNURF
GeneCard	TRNAU1AP
GeneCard	DEF6
GeneCard	TAF9
GeneCard	FNTB
GeneCard	IRS3P
GeneCard	MOB1B
GeneCard	ZC3H11A
GeneCard	APOC1P1
GeneCard	GTF2E1
GeneCard	PLA2G4D
GeneCard	SLCO1C1
GeneCard	H2BC14
GeneCard	MAGEH1
GeneCard	UBE2M
GeneCard	LINC01684
GeneCard	KCNU1
GeneCard	DZIP3
GeneCard	PTCHD4
GeneCard	ZSCAN26
GeneCard	ADARB2-AS1
GeneCard	SAE1
GeneCard	GRASLND
GeneCard	POLD3
GeneCard	CCNI
GeneCard	SHTN1
GeneCard	CTNNBIP1
GeneCard	EVI5L
GeneCard	HEXIM2
GeneCard	RAB14
GeneCard	NOTUM
GeneCard	TLE2
GeneCard	FAM241B
GeneCard	ANKRD31
GeneCard	DDX47
GeneCard	SGTA
GeneCard	RHBDD2
GeneCard	NOP16
GeneCard	MIR603
GeneCard	NKPD1
GeneCard	NOMO2
GeneCard	GPATCH4
GeneCard	PRR3
GeneCard	HOXA3
GeneCard	IGSF23
GeneCard	ADCY10P1

GeneCard	CEACAM22P
GeneCard	RPL7P51
GeneCard	RNU6-1276P
GeneCard	ENSG00000271046
GeneCard	SIGLEC22P
GeneCard	ENSG00000265206
GeneCard	ENSG00000264112
GeneCard	lnc-OARD1-1
GeneCard	ENSG00000260634
GeneCard	HSALNG0018586
GeneCard	lnc-CYP27C1-1
GeneCard	HSALNG0049988
GeneCard	HSALNG0076170
GeneCard	piR-35674-264
GeneCard	lnc-RNF43-2
GeneCard	RF00017-2265
GeneCard	lnc-TEX51-4
GeneCard	lnc-USP6NL-2
GeneCard	MN297491
GeneCard	HSALNG0010115
GeneCard	HSALNG0010116
GeneCard	HSALNG0049989
GeneCard	LOC105373605
GeneCard	HSALNG0018603
GeneCard	HSALNG0076172
GeneCard	HSALNG0064191
GeneCard	piR-33103-011
GeneCard	piR-56580-022
GeneCard	piR-40605-005
GeneCard	LOC105376412
GeneCard	LOC105376413
GeneCard	MAIP1
GeneCard	CEACAM16-AS1
GeneCard	LINC02498
GeneCard	ENSG00000269148
GeneCard	ENSG00000242798
GeneCard	lnc-BCL3-1
GeneCard	lnc-BCL3-5
GeneCard	S1PR5
GeneCard	TCEANC2
GeneCard	LANCL2
GeneCard	LARP1B
GeneCard	DYNC1LI2
GeneCard	ADGRF4
GeneCard	NECTIN3
GeneCard	CBX4
GeneCard	SLC38A1
GeneCard	RPL34P11
GeneCard	KLF3
GeneCard	PIP4K2B
GeneCard	TRIM69
GeneCard	C2orf16
GeneCard	PARP3
GeneCard	MRPS15
GeneCard	PAFAH1B2
GeneCard	ARL8B
GeneCard	ZNF597
GeneCard	TNIP2

GeneCard	TMEM132C
GeneCard	PCMTD1
GeneCard	MS4A13
GeneCard	LETMD1
GeneCard	FKBP8
GeneCard	TMEM14A
GeneCard	INTS6
GeneCard	ACOT4
GeneCard	PARP2
GeneCard	MAGOH
GeneCard	IHO1
GeneCard	CHMP7
GeneCard	HNRNPA0
GeneCard	CPB1
GeneCard	KIAA0513
GeneCard	SUMO3
GeneCard	CNOT2
GeneCard	TRABD
GeneCard	KRT84
GeneCard	MRPL30
GeneCard	KIF3C
GeneCard	LRRC8D
GeneCard	BAALC-AS1
GeneCard	ENSG00000224431
GeneCard	VN2R20P
GeneCard	NR2C2AP
GeneCard	PARD6A
GeneCard	SF3B5
GeneCard	POTEE
GeneCard	MIR545
GeneCard	BASP1-AS1
GeneCard	PNO1
GeneCard	ZBTB5
GeneCard	LINC00639
GeneCard	SNHG19
GeneCard	NTMT1
GeneCard	GRAP2
GeneCard	TRA2A
GeneCard	ASB1
GeneCard	EEF1A1P14
GeneCard	KLHL18
GeneCard	VTRNA2-1
GeneCard	SCAMP2
GeneCard	ZNF366
GeneCard	FGFBP1
GeneCard	PARP15
GeneCard	GABPB2
GeneCard	HARBI1
GeneCard	FAM131C
GeneCard	STARD10
GeneCard	VLDLR-AS1
GeneCard	PFDN6
GeneCard	RASL12
GeneCard	PCED1B
GeneCard	AIDA
GeneCard	PFDN5
GeneCard	PCYOX1
GeneCard	PPP1R16B

GeneCard	DDIT4L
GeneCard	LRRIQ3
GeneCard	WASH2P
GeneCard	DDX24
GeneCard	METTL17
GeneCard	MIR302F
GeneCard	OSBPL5
GeneCard	NOL6
GeneCard	ZNRF4
GeneCard	PDAP1
GeneCard	ZSCAN12
GeneCard	RASSF2
GeneCard	NSD3
GeneCard	ZNF414
GeneCard	DTWD1
GeneCard	ACBD6
GeneCard	RCHY1
GeneCard	LMCD1
GeneCard	MAP7D1
GeneCard	SRRM4
GeneCard	MRPS33
GeneCard	OR6V1
GeneCard	C1orf147
GeneCard	COL22A1
GeneCard	NLN
GeneCard	SAMD4B
GeneCard	BATF
GeneCard	MIR384
GeneCard	ZNF473
GeneCard	GOLPH3L
GeneCard	STRBP
GeneCard	MIR3945HG
GeneCard	KLHDC10
GeneCard	ZNF500
GeneCard	LDOC1
GeneCard	FBXL22
GeneCard	SLC38A3
GeneCard	DNAJB7
GeneCard	U2SURP
GeneCard	GTF3C5
GeneCard	ZSCAN16
GeneCard	ADGRF2
GeneCard	GSTA5
GeneCard	ZC3H8
GeneCard	KRT222
GeneCard	GPR26
GeneCard	GSPT1
GeneCard	GOSR1
GeneCard	ARL16
GeneCard	BIN3
GeneCard	TMX4
GeneCard	CIART
GeneCard	RHEBL1
GeneCard	OR2K2
GeneCard	TAF7
GeneCard	MYL10
GeneCard	GLB1L
GeneCard	RBM34

GeneCard	PAXX
GeneCard	LINC01081
GeneCard	LRP4-AS1
GeneCard	RHOJ
GeneCard	ZMYND8
GeneCard	OCIAD2
GeneCard	NEMP1
GeneCard	LENG1
GeneCard	FUOM
GeneCard	MRPS35P1
GeneCard	DMAC1
GeneCard	C17orf80
GeneCard	NLGN4Y
GeneCard	KLHL4
GeneCard	NUDCD2
GeneCard	FAM214B
GeneCard	SPIRE1
GeneCard	STK40
GeneCard	NINJ2
GeneCard	GTF3C4
GeneCard	HSD11B1L
GeneCard	ZBTB39
GeneCard	TEX33
GeneCard	EIF3K
GeneCard	ARPP21
GeneCard	PCBP3
GeneCard	BAALC-AS2
GeneCard	MRPL51
GeneCard	ELL2
GeneCard	PRB1
GeneCard	ZNF234
GeneCard	ZNF235
GeneCard	MEIOSIN
GeneCard	LOC100505715
GeneCard	STAG3L5P
GeneCard	ENSG00000250948
GeneCard	ZNF45-AS1
GeneCard	ENSG00000267114
GeneCard	ENSG00000262039
GeneCard	lnc-CEACAM20-2
GeneCard	lnc-EPHX2-4
GeneCard	HSALNG0126489
GeneCard	HSALNG0126491
GeneCard	HSALNG0064168-001
GeneCard	RF00017-6441
GeneCard	LINC01848
GeneCard	DLGAP5
GeneCard	MON2
GeneCard	ZC3H15
GeneCard	TMBIM4
GeneCard	ZBTB17
GeneCard	NEK7
GeneCard	ISY1
GeneCard	SCRN1
GeneCard	RGS12
GeneCard	C10orf88
GeneCard	FTLP4
GeneCard	RNF183

GeneCard	ZNF862
GeneCard	LINC00515
GeneCard	MRPS10
GeneCard	FAM110A
GeneCard	KRT82
GeneCard	PLEKHO2
GeneCard	MARCHF7
GeneCard	MRPL50
GeneCard	PCDHGC3
GeneCard	ZBTB6
GeneCard	SH3BGRL3
GeneCard	ZCCHC3
GeneCard	AMER2
GeneCard	CCDC89
GeneCard	TYW3
GeneCard	ZNF430
GeneCard	GNRH2
GeneCard	VAT1
GeneCard	IRGQ
GeneCard	SERINC5
GeneCard	SKIL
GeneCard	GTF2A1L
GeneCard	TMCC2
GeneCard	DNPH1
GeneCard	C17orf49
GeneCard	MAPRE1P2
GeneCard	ZNF792
GeneCard	TXNDC5
GeneCard	RPL17-C18orf32
GeneCard	OTULINL
GeneCard	DDT
GeneCard	TANGO6
GeneCard	DUSP16
GeneCard	TMUB1
GeneCard	JPT1
GeneCard	GGT7
GeneCard	MIR4675
GeneCard	PHYHD1
GeneCard	C19orf53
GeneCard	ZNHIT1
GeneCard	ZNF813
GeneCard	CADM3
GeneCard	RPL9P19
GeneCard	LINC01671
GeneCard	FCAMR
GeneCard	DEXI
GeneCard	MRPL34
GeneCard	TOMM22P6
GeneCard	FAM209B
GeneCard	MYL12B
GeneCard	ARHGAP23
GeneCard	AAMDC
GeneCard	MED9
GeneCard	HMBOX1
GeneCard	DHX29
GeneCard	RAMP2
GeneCard	FYTTD1
GeneCard	NECAB2

GeneCard	ZNF622
GeneCard	BTBD3
GeneCard	ARHGAP17
GeneCard	PLA2G4C
GeneCard	SPDYE3
GeneCard	SPATA1
GeneCard	GCFC2
GeneCard	SHISA2
GeneCard	MOB3C
GeneCard	TRIM38
GeneCard	F8A2
GeneCard	F8A3
GeneCard	EMC7
GeneCard	PDIA5
GeneCard	PLPPR1
GeneCard	SRSF12
GeneCard	LINC-PINT
GeneCard	RPL21P119
GeneCard	CASC18
GeneCard	DGCR11
GeneCard	RN7SL2
GeneCard	RN7SL3
GeneCard	PSD3
GeneCard	TCP10L
GeneCard	ING4
GeneCard	RCOR3
GeneCard	APOBEC3H
GeneCard	TOMM22
GeneCard	SRSF11
GeneCard	TRIM42
GeneCard	HAS2-AS1
GeneCard	MYL5
GeneCard	SMIM14
GeneCard	PLCL2
GeneCard	COPS2
GeneCard	SRARP
GeneCard	CLK4
GeneCard	CDH16
GeneCard	ZNF385B
GeneCard	MYBPHL
GeneCard	LINC01122
GeneCard	GALNT7
GeneCard	NHLRC3
GeneCard	RNF217
GeneCard	DCUN1D2
GeneCard	PPP1R11
GeneCard	ITM2C
GeneCard	FRMD8
GeneCard	NAP1L4
GeneCard	ARL4C
GeneCard	ZNF285B
GeneCard	CAVIN4
GeneCard	RAB2B
GeneCard	COPZ1
GeneCard	SKA3
GeneCard	RGS8
GeneCard	MIR519D
GeneCard	ZDHHC23

GeneCard	CHCHD6
GeneCard	CZIB
GeneCard	ZNF433
GeneCard	THOC3
GeneCard	RABL6
GeneCard	MRPS35P3
GeneCard	ARHGEF40
GeneCard	DHRS4
GeneCard	HNRNPA1P41
GeneCard	LCE1D
GeneCard	ADAM2
GeneCard	ENKUR
GeneCard	RNH1
GeneCard	STMN3
GeneCard	NASP
GeneCard	ZNF574
GeneCard	GIMAP1
GeneCard	S100A16
GeneCard	DDX6P2
GeneCard	LSM3
GeneCard	PARP10
GeneCard	VSTM2L
GeneCard	DNAJC8
GeneCard	FKBP2
GeneCard	SEPTIN11
GeneCard	OR7E116P
GeneCard	ZBTB48
GeneCard	RFTN1
GeneCard	MFSD10
GeneCard	TMEM41A
GeneCard	C5orf22
GeneCard	MRTFB
GeneCard	SSR3
GeneCard	UBE2Q2
GeneCard	FBXO44
GeneCard	AKAP11
GeneCard	DOCK11
GeneCard	BRSK1
GeneCard	SLC43A3
GeneCard	DAPL1
GeneCard	ESAM
GeneCard	MIR613
GeneCard	SPATA48
GeneCard	DUSP18
GeneCard	ZNF233
GeneCard	PMS2CL
GeneCard	KRT76
GeneCard	FBXL12
GeneCard	C9orf64
GeneCard	MINDY2
GeneCard	PRR7
GeneCard	LOC100288123
GeneCard	TPM1-AS
GeneCard	ENSG00000248714
GeneCard	ENSG00000267317
GeneCard	RNA5SP431
GeneCard	ENSG00000267282
GeneCard	RNU6-560P

GeneCard	lnc-APOC1-1
GeneCard	lnc-EED-4
GeneCard	lnc-INTS8-3
GeneCard	lnc-NGEF-1
GeneCard	HSALNG0060016
GeneCard	MK280607-097
GeneCard	MN298114-236
GeneCard	piR-48950-118
GeneCard	ZP3P2
GeneCard	RF00017-6032
GeneCard	RF00017-6710
GeneCard	HSALNG0126508
GeneCard	NONHSAG026007.2
GeneCard	HSALNG0126509
GeneCard	piR-61028-297
GeneCard	RF00017-2756
GeneCard	PAXBP1
GeneCard	TEX10
GeneCard	ATG4C
GeneCard	UBL3
GeneCard	PCMTD2
GeneCard	ELF2
GeneCard	C1orf94
GeneCard	CCDC171
GeneCard	LCE1B
GeneCard	TSPAN6
GeneCard	CTPS2
GeneCard	DEDD
GeneCard	TWSG1
GeneCard	EMC8
GeneCard	NAT8B
GeneCard	NUAK2
GeneCard	RPL15P15
GeneCard	LINC00624
GeneCard	CATSPERD
GeneCard	ERMP1
GeneCard	FAM3A
GeneCard	ADGRA1
GeneCard	FAM204A
GeneCard	ARHGEF7
GeneCard	PNMA8A
GeneCard	GCNT7
GeneCard	KRBOX1
GeneCard	STAG3L5P-PVRIG2P-PILRB
GeneCard	LINC01419
GeneCard	PRKRIP1
GeneCard	LINC02098
GeneCard	RIOK1
GeneCard	NEUROG1
GeneCard	CXXC4
GeneCard	USP46
GeneCard	BEX2
GeneCard	ZNF286B
GeneCard	HECTD1
GeneCard	H3C13
GeneCard	MORC4
GeneCard	DUSP26
GeneCard	DPPA2

GeneCard	SLC7A3
GeneCard	CORO2A
GeneCard	PDCD2
GeneCard	CARHSP1
GeneCard	OR52B4
GeneCard	SDR42E1
GeneCard	CAPG
GeneCard	ARMH4
GeneCard	SOCS7
GeneCard	PITHD1
GeneCard	B3GALT1
GeneCard	ACSS3
GeneCard	ENTPD2
GeneCard	ZNF768
GeneCard	KLK9
GeneCard	KCNAB3
GeneCard	PDZK1IP1
GeneCard	FAM83E
GeneCard	RAB20
GeneCard	SPZ1
GeneCard	H3C15
GeneCard	HDHD3
GeneCard	RGS17
GeneCard	UBXN1
GeneCard	PCIF1
GeneCard	PABPC3
GeneCard	BRIX1
GeneCard	LHFPL6
GeneCard	RNU7-51P
GeneCard	CEACAM20
GeneCard	PTGR2
GeneCard	RHBDD1
GeneCard	LHX5
GeneCard	NKX2-8
GeneCard	IQCD
GeneCard	RPS6KA6
GeneCard	KLHL32
GeneCard	LRRC47
GeneCard	CCDC137
GeneCard	NPVF
GeneCard	LOC644135
GeneCard	ABR
GeneCard	TP53TG1
GeneCard	GGCT
GeneCard	ARHGAP28
GeneCard	DUSP10
GeneCard	ZNF273
GeneCard	RTL8C
GeneCard	DDI1
GeneCard	AKR1E2
GeneCard	IL27RA
GeneCard	ASB8
GeneCard	STXBP4
GeneCard	NSRP1
GeneCard	GPATCH2L
GeneCard	RPS20P25
GeneCard	ANKRD23
GeneCard	SLC22A17

GeneCard	DUSP12
GeneCard	AFG3L1P
GeneCard	UNC5D
GeneCard	KLHL1
GeneCard	C3orf56
GeneCard	NOL12
GeneCard	CASKIN2
GeneCard	DEPP1
GeneCard	PSME4
GeneCard	RAD51AP1
GeneCard	POLR2M
GeneCard	GIMAP8
GeneCard	CIAO3
GeneCard	HCG25
GeneCard	ENSG00000266469
GeneCard	LOC111258526
GeneCard	MZT2B
GeneCard	LYPLA2
GeneCard	RPS27P18
GeneCard	FEZ2
GeneCard	VCY
GeneCard	UBE2O
GeneCard	SAMD7
GeneCard	SYCP2L
GeneCard	CDKL2
GeneCard	RPS26P28
GeneCard	OR7E106P
GeneCard	RPL13AP21
GeneCard	LOC101927598
GeneCard	HSFX1
GeneCard	NGEF
GeneCard	GOLGA6L5P
GeneCard	SIGLEC16
GeneCard	IGHV3-71
GeneCard	PATL1
GeneCard	CDK3
GeneCard	UGGT2
GeneCard	ZNF594
GeneCard	MS4A18
GeneCard	MIR3591
GeneCard	GEMIN7-AS1
GeneCard	TOB1
GeneCard	RGL3
GeneCard	ENSG00000284686
GeneCard	HDHD2
GeneCard	CCNT2-AS1
GeneCard	PRG1
GeneCard	AMOTL2
GeneCard	CAPN6
GeneCard	LINC00163
GeneCard	OR4C3
GeneCard	SLC10A6
GeneCard	CTSO
GeneCard	ATRAID
GeneCard	SMIM29
GeneCard	LSM5
GeneCard	CDKL3
GeneCard	MIR1229

GeneCard	CHID1
GeneCard	LOC105373170
GeneCard	PCBD2
GeneCard	ETFRF1
GeneCard	ALDH8A1
GeneCard	NXF2B
GeneCard	CDYL
GeneCard	SFT2D2
GeneCard	ADAP2
GeneCard	LSM6
GeneCard	TNFAIP8L1
GeneCard	TRIM43
GeneCard	TRMT6
GeneCard	NXPE1
GeneCard	MYRF-AS1
GeneCard	BEGAIN
GeneCard	SLC16A5
GeneCard	CAB39L
GeneCard	ASMTL
GeneCard	SPATA46
GeneCard	SLC38A5
GeneCard	MAST4
GeneCard	ATXN7L3
GeneCard	SPATA8
GeneCard	ZNF48
GeneCard	ANKS4B
GeneCard	TMC5
GeneCard	R3HDML
GeneCard	CNIH1
GeneCard	TMEM198B
GeneCard	CHMP6
GeneCard	CDC42EP4
GeneCard	ZKSCAN5
GeneCard	PRR15
GeneCard	COMMD10
GeneCard	HES4
GeneCard	SUCNR1
GeneCard	FNDC3A
GeneCard	CCDC190
GeneCard	C12orf40
GeneCard	C3orf36
GeneCard	MT4
GeneCard	PPEF1
GeneCard	GPR153
GeneCard	PIAS4
GeneCard	ARMCX5
GeneCard	ZNF12
GeneCard	ZSCAN2
GeneCard	LYPLAL1
GeneCard	RPL21P105
GeneCard	LIMD2
GeneCard	NPIPB5
GeneCard	RUSF1
GeneCard	SIVA1
GeneCard	SIDT2
GeneCard	SH3BP1
GeneCard	EVL
GeneCard	ACTL8

GeneCard	RPS17P11
GeneCard	ZCCHC12
GeneCard	ARID3A
GeneCard	CHRAC1
GeneCard	ANKRD12
GeneCard	SPATA17
GeneCard	OR2A1
GeneCard	OR2AE1
GeneCard	PLGLA
GeneCard	TREML3P
GeneCard	LINC01479
GeneCard	TREML5P
GeneCard	LOC154761
GeneCard	ARHGEF35-AS1
GeneCard	RPL39P
GeneCard	ZNF230-DT
GeneCard	ZNF232-AS1
GeneCard	APP-DT
GeneCard	EIF5AP3
GeneCard	LINC02705
GeneCard	PVRIG2P
GeneCard	OR2A13P
GeneCard	ENSG00000224541
GeneCard	ENSG00000238273
GeneCard	ENSG00000255197
GeneCard	ENSG00000246790
GeneCard	ENSG00000245025
GeneCard	ENSG00000235522
GeneCard	C17orf114
GeneCard	RN7SL535P
GeneCard	PPP1R35-AS1
GeneCard	LOC100289518
GeneCard	ENSG00000263164
GeneCard	ENSG00000263220
GeneCard	ENSG00000255355
GeneCard	ENSG00000235085
GeneCard	CD2AP-DT
GeneCard	LINC02695
GeneCard	SUMO2P6
GeneCard	TAS2R62P
GeneCard	RPS16P9
GeneCard	RNU6-611P
GeneCard	ENSG00000186019
GeneCard	ENSG00000258985
GeneCard	ENSG00000261526
GeneCard	ENSG00000263272
GeneCard	ENSG00000267044
GeneCard	ENSG00000267257
GeneCard	ENSG00000267283
GeneCard	ENSG00000241357
GeneCard	ENSG00000200090
GeneCard	RN7SKP102
GeneCard	ENSG00000285664
GeneCard	ENSG00000274354
GeneCard	ENSG00000288045
GeneCard	ENSG00000218809
GeneCard	ENSG00000252200
GeneCard	lnc-ALDH1A2-9

GeneCard	lnc-FERMT2-2
GeneCard	NONHSAG008235.2
GeneCard	ENSG00000262227
GeneCard	ENSG00000279753
GeneCard	HSALNG0017603
GeneCard	ENSG00000279095
GeneCard	HSALNG0007914
GeneCard	ENSG00000228335
GeneCard	YWHAZP9
GeneCard	HSALNG0126529
GeneCard	lnc-CCDC83-3
GeneCard	lnc-CYYR1-5
GeneCard	HSALNG0132408
GeneCard	lnc-CEACAM16-1
GeneCard	lnc-CEACAM16-2
GeneCard	lnc-CEACAM19-2
GeneCard	lnc-CLU-2
GeneCard	lnc-MARK4-1
GeneCard	HSALNG0126505
GeneCard	HSALNG0131068
GeneCard	lnc-CYYR1-6
GeneCard	lnc-GPR141-3
GeneCard	lnc-BLID-6
GeneCard	HSALNG0103265
GeneCard	HSALNG0059997
GeneCard	HSALNG0059998
GeneCard	HSALNG0122970
GeneCard	HSALNG0059996
GeneCard	HSALNG0101310
GeneCard	lnc-SYTL2-5
GeneCard	MN298458
GeneCard	piR-39098-253
GeneCard	lnc-TMEM259-5
GeneCard	lnc-TMEM259-4
GeneCard	NONHSAG009267.2
GeneCard	RF00017-3346
GeneCard	piR-42851
GeneCard	lnc-PSMC3-1
GeneCard	ENSG00000280087
GeneCard	HSALNG0017601
GeneCard	HSALNG0023072
GeneCard	HSALNG0007913
GeneCard	HSALNG0126487
GeneCard	lnc-BLOC1S3-1
GeneCard	HSALNG0126526
GeneCard	lnc-EPHX2-1
GeneCard	HSALNG0103266
GeneCard	HSALNG0049427
GeneCard	HSALNG0064193
GeneCard	HSALNG0086209
GeneCard	HSALNG0106226
GeneCard	HSALNG0114109
GeneCard	HSALNG0122967
GeneCard	HSALNG0064192
GeneCard	NONHSAG008424.2
GeneCard	NONHSAG008427.2
GeneCard	NONHSAG024334.2
GeneCard	piR-34451

GeneCard	piR-43105-088
GeneCard	piR-48209-263
GeneCard	piR-48759-046
GeneCard	piR-57337-010
GeneCard	LOC105370500
GeneCard	lnc-RAB8B-2
GeneCard	lnc-RAPSN-1
GeneCard	RF00026-195
GeneCard	RF00026-501
GeneCard	SNODB480
GeneCard	LOC107985306
GeneCard	FJ601684-431
GeneCard	LOC100420902
GeneCard	HSALNG0126532
GeneCard	HSALNG0126533
GeneCard	lnc-BLOC1S3-2
GeneCard	HSALNG0126506
GeneCard	HSALNG0126511
GeneCard	HSALNG0126512
GeneCard	HSALNG0126525
GeneCard	HSALNG0126522
GeneCard	HSALNG0126473
GeneCard	HSALNG0126500
GeneCard	HSALNG0126501
GeneCard	HSALNG0126503
GeneCard	HSALNG0126504
GeneCard	HSALNG0126523
GeneCard	HSALNG0126524
GeneCard	HSALNG0126513
GeneCard	HSALNG0126538
GeneCard	HSALNG0126530
GeneCard	HSALNG0132406
GeneCard	lnc-CLU-4
GeneCard	HSALNG0061964
GeneCard	HSALNG0106227
GeneCard	HSALNG0062049
GeneCard	HSALNG0084442
GeneCard	HSALNG0106477
GeneCard	HSALNG0122971
GeneCard	HSALNG0060001
GeneCard	HSALNG0064187
GeneCard	HSALNG0084443
GeneCard	HSALNG0084051
GeneCard	HSALNG0122969
GeneCard	lnc-TNFRSF21-1
GeneCard	lnc-TREML4-1
GeneCard	lnc-TREM1-3
GeneCard	MK280046
GeneCard	MN298426
GeneCard	piR-32285-084
GeneCard	piR-35002-105
GeneCard	lnc-SYTL2-4
GeneCard	lnc-TRAPPC6A-2
GeneCard	piR-30449-174
GeneCard	lnc-SLC52A1-3
GeneCard	NONHSAG026004.2
GeneCard	piR-30091-098
GeneCard	piR-31199-347

GeneCard	piR-31534-344
GeneCard	piR-39099-259
GeneCard	piR-48301-221
GeneCard	piR-51999-003
GeneCard	piR-56022-272
GeneCard	piR-56022-298
GeneCard	piR-61514-039
GeneCard	piR-48759-295
GeneCard	piR-56133-041
GeneCard	piR-56133-114
GeneCard	piR-57460-090
GeneCard	MN297596
GeneCard	piR-43105-087
GeneCard	piR-45605-003
GeneCard	piR-52438-098
GeneCard	piR-53819-005
GeneCard	piR-55657-145
GeneCard	piR-43408-141
GeneCard	piR-55654-285
GeneCard	piR-59907-014
GeneCard	RF00017-2757
GeneCard	LOC105375427
GeneCard	LOC105372420
GeneCard	HSALNG0084440
GeneCard	HSALNG0064174
GeneCard	piR-48301-198
GeneCard	piR-39488-149
GeneCard	LOC102724025
GeneCard	ENSG00000255331
GeneCard	TSC22D2
GeneCard	CAMKK1
GeneCard	ARHGEF10L
GeneCard	ASCL3
GeneCard	SETP2
GeneCard	RPL17P46
GeneCard	OR6R2P
GeneCard	IFNA16
GeneCard	RNF113B
GeneCard	TMEM267
GeneCard	ZHX1
GeneCard	MAF1
GeneCard	INO80E
GeneCard	TRIM23
GeneCard	ELOA2
GeneCard	MAGEA2
GeneCard	SGK2
GeneCard	ZMAT3
GeneCard	LINC00476
GeneCard	MRPL54
GeneCard	HDDC2
GeneCard	ARRDC1
GeneCard	HS3ST4
GeneCard	UTP6
GeneCard	UQCC1
GeneCard	RPUSD2
GeneCard	MIR922
GeneCard	DHRS7B
GeneCard	ZIK1

GeneCard	PBX4
GeneCard	LNX2
GeneCard	MIR365B
GeneCard	RPL31P13
GeneCard	MAP3K12
GeneCard	GPR12
GeneCard	FMNL1
GeneCard	TMEM61
GeneCard	HMG20B
GeneCard	CCDC124
GeneCard	PCDHA7
GeneCard	ART4
GeneCard	NOL7
GeneCard	ECHDC2
GeneCard	MIR302A
GeneCard	KRCC1
GeneCard	HEBP2
GeneCard	C9orf92
GeneCard	LINC02151
GeneCard	LINC00319
GeneCard	METTL13
GeneCard	MIR4425
GeneCard	LINC01982
GeneCard	RUNX3-AS1
GeneCard	RNU6-343P
GeneCard	RN7SL809P
GeneCard	ENSG00000184441
GeneCard	ENSG00000257740
GeneCard	ENSG00000258317
GeneCard	lnc-TWIST1-1
GeneCard	lnc-SFRP4-1
GeneCard	USP21
GeneCard	CDK2AP1
GeneCard	C5orf15
GeneCard	CITED1
GeneCard	RHNO1
GeneCard	TCP11
GeneCard	TM2D2
GeneCard	NACC2
GeneCard	TMEM205
GeneCard	C4orf33
GeneCard	MYEOV
GeneCard	CIAO2B
GeneCard	RASSF3
GeneCard	ORMDL2
GeneCard	KLHL38
GeneCard	ZNF839
GeneCard	ZFYVE28
GeneCard	ACBD4
GeneCard	TENT5B
GeneCard	ALDH1L2
GeneCard	CCDC85A
GeneCard	MIR1908
GeneCard	TCP10L3
GeneCard	TRIM46
GeneCard	USP28
GeneCard	MMGT1
GeneCard	SH3GLB2

GeneCard	CEND1
GeneCard	DIRAS1
GeneCard	HLA-V
GeneCard	TMEM109
GeneCard	ATF7IP2
GeneCard	LINC02245
GeneCard	RPL36AP40
GeneCard	PAIP2
GeneCard	RBPJL
GeneCard	NAP1L2
GeneCard	ORMDL1
GeneCard	OVCH1-AS1
GeneCard	TSPAN5
GeneCard	SLC4A9
GeneCard	PPP1R32
GeneCard	GLYATL3
GeneCard	RPS23P3
GeneCard	SERTAD4
GeneCard	NUSAP1
GeneCard	C16orf54
GeneCard	NUDT14
GeneCard	LINC01599
GeneCard	FAM86B3P
GeneCard	WDR44
GeneCard	FILIP1
GeneCard	CCDC32
GeneCard	C11orf54
GeneCard	C15orf48
GeneCard	WWC3
GeneCard	LINC01093
GeneCard	LZTS3
GeneCard	CATSPER3
GeneCard	GLI4
GeneCard	TRIM47
GeneCard	AIFM3
GeneCard	PCDHGA12
GeneCard	GPBP1L1
GeneCard	FLRT2
GeneCard	TTC27
GeneCard	KLHL6
GeneCard	CCDC159
GeneCard	PLK3
GeneCard	PTTG2
GeneCard	MRGPRX3
GeneCard	ZFX
GeneCard	APEX2
GeneCard	CHST1
GeneCard	ASCL4
GeneCard	CNIH2
GeneCard	RIMS3
GeneCard	TMEM123
GeneCard	ZFC3H1
GeneCard	SPATS2
GeneCard	CD300LG
GeneCard	ARAP2
GeneCard	ZNF844
GeneCard	SNORA21
GeneCard	HHLA1

GeneCard	RPRD2
GeneCard	ZFP64
GeneCard	RAMP1
GeneCard	POLDIP2
GeneCard	CDK15
GeneCard	KIAA0408
GeneCard	IGHV3-72
GeneCard	ZFAND5
GeneCard	SSX2B
GeneCard	TBC1D21
GeneCard	MSANTD3
GeneCard	SPRNP1
GeneCard	HOXC11
GeneCard	GSTT2B
GeneCard	SCEL
GeneCard	ADIRF
GeneCard	PRRG2
GeneCard	SH3RF1
GeneCard	TBC1D16
GeneCard	C8orf58
GeneCard	TMCO5B
GeneCard	UGT1A13P
GeneCard	MAGEA11
GeneCard	DSN1
GeneCard	PHAF1
GeneCard	RIMS4
GeneCard	LOC107882133
GeneCard	RPS15AP30
GeneCard	WSCD1
GeneCard	MIR650
GeneCard	CDK16
GeneCard	IQGAP3
GeneCard	BRME1
GeneCard	OTUB2
GeneCard	RNF166
GeneCard	TES
GeneCard	RAPGEFL1
GeneCard	TMA7
GeneCard	GINS3
GeneCard	TASOR2
GeneCard	IKBIP
GeneCard	PIMREG
GeneCard	ELP5
GeneCard	GRINA
GeneCard	MIR539
GeneCard	ST3GAL3-AS1
GeneCard	GFPT2
GeneCard	PDE1B
GeneCard	SNORD111B
GeneCard	HIGD2A
GeneCard	ZNF22-AS1
GeneCard	KIAA0930
GeneCard	MALSU1
GeneCard	ZBTB7C
GeneCard	IFI6
GeneCard	NETO1
GeneCard	CCNB1IP1
GeneCard	FANK1

GeneCard	TBC1D9B
GeneCard	FSBP
GeneCard	SERF2
GeneCard	TMEM53
GeneCard	RPS17P2
GeneCard	H2AW
GeneCard	LRRC1
GeneCard	DNAJB12
GeneCard	MAP3K9
GeneCard	UBE2L2
GeneCard	ANKEF1
GeneCard	ARMC7
GeneCard	TLE5
GeneCard	MAPK8IP2
GeneCard	CCDC90B
GeneCard	ARL17B
GeneCard	CFAP20DC
GeneCard	LINC01588
GeneCard	AQP4-AS1
GeneCard	LINC01099
GeneCard	LOC171391
GeneCard	LINC02064
GeneCard	ENSG00000260971
GeneCard	LINC02815
GeneCard	RNU6-189P
GeneCard	RNU6-565P
GeneCard	ENSG00000282885
GeneCard	ENSG00000258199
GeneCard	RNA5SP173
GeneCard	RNU6-976P
GeneCard	lnc-C1orf74-1
GeneCard	HSALNG0101144
GeneCard	HSALNG0092134
GeneCard	MK280269-012
GeneCard	RF00017-4423
GeneCard	LOC107984526
GeneCard	HSALNG0101147-002
GeneCard	RF00017-5681
GeneCard	RF00017-5682
GeneCard	RNU7-66P
GeneCard	HPCAL4
GeneCard	DPP3
GeneCard	CLIC3
GeneCard	LMCD1-AS1
GeneCard	RBMXP1
GeneCard	TIMM44
GeneCard	LOC109113862
GeneCard	PELI3
GeneCard	KLHL17
GeneCard	PRDM16-DT
GeneCard	TRIM41
GeneCard	DLGAP1-AS2
GeneCard	LAMTOR1
GeneCard	MAGEA8
GeneCard	LIMCH1
GeneCard	TSPAN3
GeneCard	ORAI2
GeneCard	COP1

GeneCard	LONRF2
GeneCard	C14orf93
GeneCard	ETNPPL
GeneCard	RPL3P11
GeneCard	FAM180B
GeneCard	TCP11L1
GeneCard	ZNF460
GeneCard	FAM151A
GeneCard	LMNTD1
GeneCard	BAGE
GeneCard	C3orf14
GeneCard	GOLGA6L4
GeneCard	LINC00290
GeneCard	LINC01508
GeneCard	LNC-LBCS
GeneCard	STK24-AS1
GeneCard	MOCS2-DT
GeneCard	B3GALT1-AS1
GeneCard	CTAGE14P
GeneCard	TADA3
GeneCard	MRPL27
GeneCard	TMEM185A
GeneCard	GPR137B
GeneCard	STX19
GeneCard	LOC646506
GeneCard	STK17B
GeneCard	EEPD1
GeneCard	DPYSL3
GeneCard	HOXC6
GeneCard	RASL11A
GeneCard	PPP1R2B
GeneCard	DRAM1
GeneCard	SPSB3
GeneCard	PARD6B
GeneCard	C9orf47
GeneCard	SNORD35B
GeneCard	LINC00687
GeneCard	TAOK3
GeneCard	SOX21-AS1
GeneCard	FASTK
GeneCard	RFX4
GeneCard	CCL16
GeneCard	RPSAP47
GeneCard	GAPDHP15
GeneCard	PGBD3P4
GeneCard	METTL25
GeneCard	ZNF496
GeneCard	PPP1R9A
GeneCard	FMC1-LUC7L2
GeneCard	GNG4
GeneCard	TP53TG5
GeneCard	FAM83A
GeneCard	MIR4432
GeneCard	HSFX2
GeneCard	NIM1K
GeneCard	H2BC1
GeneCard	PPM1E
GeneCard	MIR4487

GeneCard	VBP1
GeneCard	DGLUCY
GeneCard	RHPN1
GeneCard	GSE1
GeneCard	LINC00343
GeneCard	IGKV4-1
GeneCard	SLC35B1
GeneCard	TAS2R5
GeneCard	C9orf152
GeneCard	IGHV2-70
GeneCard	KRT18P30
GeneCard	PBDC1
GeneCard	ZNF343
GeneCard	RPL15P2
GeneCard	SPX
GeneCard	SYNPR
GeneCard	C6orf118
GeneCard	ARL4D
GeneCard	POTEJ
GeneCard	HAUS5
GeneCard	SGPP2
GeneCard	PITPNB
GeneCard	CARNS1
GeneCard	ZNF684
GeneCard	ALOX12-AS1
GeneCard	LINC02210-CRHR1
GeneCard	ZBTB25
GeneCard	FAM181A-AS1
GeneCard	PAQR7
GeneCard	SLC16A7
GeneCard	CAMKV
GeneCard	NARF
GeneCard	C6orf163
GeneCard	LINC01060
GeneCard	SEC1P
GeneCard	LINC00507
GeneCard	LINC00654
GeneCard	LOC100379224
GeneCard	TCEAL2
GeneCard	TMEM186
GeneCard	AKNAD1
GeneCard	PPP1R21
GeneCard	SCRG1
GeneCard	PA2G4P2
GeneCard	HCFC1R1
GeneCard	SPOCK3
GeneCard	STAG3L4
GeneCard	ZNF791
GeneCard	CENPI
GeneCard	FAM90A1
GeneCard	RNF10
GeneCard	MIR147A
GeneCard	INO80C
GeneCard	FOXS1
GeneCard	CLIP4
GeneCard	C22orf39
GeneCard	MED21
GeneCard	NPIPB4

GeneCard	PPP5D1P
GeneCard	LINC00933
GeneCard	SLC2A9-AS1
GeneCard	ENSG00000257411
GeneCard	ENSG00000255508
GeneCard	SLC41A3
GeneCard	CALCOCO1
GeneCard	TCEAL4
GeneCard	TCP11L2
GeneCard	ERVFRD-1
GeneCard	CLDN12
GeneCard	MCRS1
GeneCard	RITA1
GeneCard	HSPD1P22
GeneCard	RPL3P8
GeneCard	DCANP1
GeneCard	TCEANC
GeneCard	PTBP3
GeneCard	RGS3
GeneCard	MIR4274
GeneCard	RPL39P5
GeneCard	COMMD2
GeneCard	PASD1
GeneCard	RNF165
GeneCard	MSGN1
GeneCard	MIR3126
GeneCard	NMUR2
GeneCard	TMEM174
GeneCard	TEX12
GeneCard	ZNF385C
GeneCard	FASTKD5
GeneCard	CENPQ
GeneCard	C18orf32
GeneCard	LOC285638
GeneCard	ABTB1
GeneCard	TOX4
GeneCard	HAUS2
GeneCard	STRIP2
GeneCard	CLEC2B
GeneCard	BPESC1
GeneCard	C18orf54
GeneCard	SAXO1
GeneCard	MLF2
GeneCard	ENC1
GeneCard	ZNF774
GeneCard	MAGEB2
GeneCard	USP38
GeneCard	OVCH1
GeneCard	LRRD1
GeneCard	C7orf33
GeneCard	LINC01170
GeneCard	RPL7AP36
GeneCard	SH3RF2
GeneCard	EPB41L5
GeneCard	MIR6845
GeneCard	CP20
GeneCard	ZNF20
GeneCard	EIF1AY

GeneCard	PRR14
GeneCard	DDX43
GeneCard	PDCD7
GeneCard	CDK2AP2
GeneCard	ARMCX3
GeneCard	TMEM33
GeneCard	MRFAP1
GeneCard	ZNF747
GeneCard	KCNMB2
GeneCard	RAB40B
GeneCard	ZNF566
GeneCard	ST6GALNAC6
GeneCard	STARD6
GeneCard	MAGEA2B
GeneCard	TRIM52
GeneCard	SLC7A4
GeneCard	OXSM
GeneCard	GTPBP8
GeneCard	ZSCAN5A
GeneCard	SLFN12
GeneCard	C7orf31
GeneCard	RPPH1
GeneCard	ANKRD39
GeneCard	ATP23
GeneCard	MED11
GeneCard	SNRNP48
GeneCard	SEMA4C
GeneCard	FMC1
GeneCard	JPT2
GeneCard	EFCAB10
GeneCard	LINC01590
GeneCard	LINC00587
GeneCard	MIR4688
GeneCard	MIR3160-1
GeneCard	MIR3160-2
GeneCard	SMILR
GeneCard	FAM240B
GeneCard	MIR4695
GeneCard	LINC02520
GeneCard	INHCAP
GeneCard	LOC101929258
GeneCard	HAUS4
GeneCard	TMEM176B
GeneCard	PCDH8
GeneCard	IMP4
GeneCard	ERVK13-1
GeneCard	TCAM1P
GeneCard	LINC01117
GeneCard	LINC01271
GeneCard	LINC01505
GeneCard	LINC01748
GeneCard	MIR3150BHG
GeneCard	TEX36-AS1
GeneCard	LOC101929237
GeneCard	LOC101927533
GeneCard	LINC02109
GeneCard	LINC02208
GeneCard	PSMD10P1

GeneCard	RPL10AP6
GeneCard	LOC105373159
GeneCard	HMGN2P19
GeneCard	LINC01765
GeneCard	LINC02667
GeneCard	LINC02576
GeneCard	PHC1P1
GeneCard	MADD-AS1
GeneCard	RPL12P37
GeneCard	RPL7AP70
GeneCard	ENSG00000232043
GeneCard	ENSG00000234255
GeneCard	ENSG00000250850
GeneCard	ENSG00000253878
GeneCard	ENSG00000255446
GeneCard	ENSG00000267174
GeneCard	ENSG00000255126
GeneCard	ADAMTS7P3
GeneCard	SNRPEP4
GeneCard	RPL7AP20
GeneCard	ENSG00000226334
GeneCard	ENSG00000229751
GeneCard	ENSG00000251867
GeneCard	ENSG00000254459
GeneCard	HSPA8P15
GeneCard	LINC01916
GeneCard	RN7SKP27
GeneCard	RN7SL636P
GeneCard	RNU6-381P
GeneCard	RN7SL299P
GeneCard	RNA5SP189
GeneCard	ENSG00000282246
GeneCard	ENSG00000253252
GeneCard	ANAPC1P3
GeneCard	CYCSP17
GeneCard	OTX2P1
GeneCard	RAP1BP2
GeneCard	SALL4P5
GeneCard	RN7SKP93
GeneCard	RN7SL714P
GeneCard	RN7SL766P
GeneCard	RNU1-148P
GeneCard	RN7SKP168
GeneCard	RN7SL700P
GeneCard	RNU1-18P
GeneCard	RNU6-649P
GeneCard	HSALNG0045503
GeneCard	lnc-GLIS3-2
GeneCard	lnc-CEP295NL-1
GeneCard	RF00017-4572
GeneCard	lnc-ZCCHC7-6
GeneCard	RNU7-188P
GeneCard	HSALNG0011363
GeneCard	HSALNG0030569-001
GeneCard	lnc-NIPSNAP3B-4
GeneCard	lnc-FST-6
GeneCard	lnc-BRI3BP-7
GeneCard	lnc-ETS1-8

GeneCard	HSALNG0071020
GeneCard	HSALNG0071021
GeneCard	lnc-RNF38-4-002
GeneCard	lnc-RUNX3-3
GeneCard	RF00017-1223
GeneCard	piR-47675-007
GeneCard	RF00017-1349
GeneCard	RF00017-4574
GeneCard	ENSG00000228165
GeneCard	ENSG00000230495
GeneCard	RF00017-6424
GeneCard	RF00017-6408
GeneCard	MTCYBP27
GeneCard	MTCYBP36
GeneCard	FJ601684-376
GeneCard	LOC105376031
GeneCard	HSALNG0080722
GeneCard	HSALNG0067061
GeneCard	HSALNG0113014
GeneCard	HSALNG0063889
GeneCard	HSALNG0101148
GeneCard	MK280144-551
GeneCard	piR-31937-039
GeneCard	piR-57133-043
GeneCard	piR-43107-305
GeneCard	RF00697-002
GeneCard	HSALNG0008250
GeneCard	HSALNG0066501
GeneCard	lnc-USP36-3
GeneCard	SENP2
GeneCard	PDILT
GeneCard	ZGRF1
GeneCard	UBE2F
GeneCard	ZNF696
GeneCard	SPSB4
GeneCard	SERTAD1
GeneCard	ZBTB49
GeneCard	MED28
GeneCard	CT75
GeneCard	MIPEPP2
GeneCard	CNOT10
GeneCard	RTL8A
GeneCard	NEDD8-MDP1
GeneCard	FN3KRP
GeneCard	GRAMD4
GeneCard	PLAAT5
GeneCard	WBP2NL
GeneCard	RNR1
GeneCard	FAM133B
GeneCard	ETV3
GeneCard	H2BC3
GeneCard	SMIM12
GeneCard	ANKZF1
GeneCard	GOLGA7
GeneCard	FOLR3
GeneCard	DDX49
GeneCard	ZNF90
GeneCard	SNORD17

GeneCard	DST-AS1
GeneCard	MIR6124
GeneCard	RWDD4
GeneCard	CIDEB
GeneCard	YPEL3
GeneCard	DLG2-AS1
GeneCard	BBLN
GeneCard	C16orf87
GeneCard	C2orf83
GeneCard	NPIPB3
GeneCard	SDR42E2
GeneCard	TOMM5
GeneCard	EDF1
GeneCard	RPUSD4
GeneCard	C1orf131
GeneCard	CENPH
GeneCard	BTG2
GeneCard	MGST1
GeneCard	RWDD2B
GeneCard	WDR89
GeneCard	BMF
GeneCard	ZACN
GeneCard	NDFIP2
GeneCard	REX1BD
GeneCard	ING2
GeneCard	H2AZ2
GeneCard	DUSP4
GeneCard	LRP3
GeneCard	PLBD1
GeneCard	TSKS
GeneCard	GAGE1
GeneCard	H2BC9
GeneCard	AFM
GeneCard	EPB41L4A
GeneCard	FMO6P
GeneCard	RAB39A
GeneCard	SPIC
GeneCard	ASCC3
GeneCard	ZMAT2
GeneCard	CBWD1
GeneCard	PAG1
GeneCard	HAUS1
GeneCard	UGGT1
GeneCard	MARF1
GeneCard	RAB43
GeneCard	CCDC106
GeneCard	INIP
GeneCard	CCER1
GeneCard	OIP5
GeneCard	UBQLNL
GeneCard	ZNF322
GeneCard	DDX55
GeneCard	CACTIN-AS1
GeneCard	MIR924HG
GeneCard	LIN28B-AS1
GeneCard	MIR4462
GeneCard	NPM1P13
GeneCard	KCTD9P6

GeneCard	ENSG00000273217
GeneCard	MTCL1P1
GeneCard	GUCD1
GeneCard	FBXO15
GeneCard	FAM47B
GeneCard	RSPH10B2
GeneCard	PRAMEF17
GeneCard	ENKD1
GeneCard	PRANCR
GeneCard	TAF1B
GeneCard	LDHAL6B
GeneCard	CDC26
GeneCard	CRYBG1
GeneCard	DNTTIP1
GeneCard	ZNF697
GeneCard	EMC2
GeneCard	TRIM16
GeneCard	SERP1
GeneCard	MRFAP1L1
GeneCard	MIR4686
GeneCard	TBC1D10C
GeneCard	HK3
GeneCard	PFKFB1
GeneCard	FOXD4L6
GeneCard	PGLYRP3
GeneCard	RLF
GeneCard	DSCC1
GeneCard	GMCL1
GeneCard	BUD31
GeneCard	MED19
GeneCard	DUSP21
GeneCard	NUFIP2
GeneCard	OLFML2A
GeneCard	HDDC3
GeneCard	SPINK13
GeneCard	GLOD4
GeneCard	SIGLEC11
GeneCard	EGFEM1P
GeneCard	RRN3P1
GeneCard	NEGR1-IT1
GeneCard	LOC652276
GeneCard	C2orf92
GeneCard	PLA2G12A
GeneCard	RAB13
GeneCard	LGALS9C
GeneCard	SPINK6
GeneCard	LINC01626
GeneCard	METTL2B
GeneCard	CD300LB
GeneCard	POMZP3
GeneCard	CATIP
GeneCard	MFSD13A
GeneCard	CMTM3
GeneCard	FAM32A
GeneCard	C9orf43
GeneCard	DDA1
GeneCard	DOCK10
GeneCard	TFAP4

GeneCard	UBQLN3
GeneCard	LILRA5
GeneCard	PPP1R42
GeneCard	RAD51AP2
GeneCard	KRTAP7-1
GeneCard	LINC00477
GeneCard	EXOC1L
GeneCard	LINC01340
GeneCard	OSER1-DT
GeneCard	FTH1P5
GeneCard	ZNF75CP
GeneCard	NPEPPSP1
GeneCard	SPON1-AS1
GeneCard	RNU7-62P
GeneCard	C9orf24
GeneCard	CA7
GeneCard	ATG10
GeneCard	PELO
GeneCard	ANAPC11
GeneCard	CRIP2
GeneCard	C1orf115
GeneCard	PKMYT1
GeneCard	UTP23
GeneCard	ADAD2
GeneCard	C10orf90
GeneCard	ZFHX2
GeneCard	SCAND2P
GeneCard	LRRC2-AS1
GeneCard	ZNF440
GeneCard	MTURN
GeneCard	FAM9A
GeneCard	RPL26P32
GeneCard	SPATA4
GeneCard	CDS2
GeneCard	ACTL7A
GeneCard	KLHL35
GeneCard	LYG1
GeneCard	PCP4
GeneCard	ALKBH7
GeneCard	APOBR
GeneCard	PLEKHS1
GeneCard	AUNIP
GeneCard	SPINDOC
GeneCard	FOXJ2
GeneCard	RASL10B
GeneCard	TSNAXIP1
GeneCard	PNMA5
GeneCard	FAM214A
GeneCard	FAM181A
GeneCard	SMIM24
GeneCard	C8orf44-SGK3
GeneCard	FAM106A
GeneCard	MIR4752
GeneCard	VSIG10L2
GeneCard	LINC00653
GeneCard	MAILR
GeneCard	FNTAP1
GeneCard	MIR4643

GeneCard	LOC440311
GeneCard	DTX3L
GeneCard	ZC2HC1A
GeneCard	PLPP1
GeneCard	SNX21
GeneCard	NUDT9
GeneCard	LINC01722
GeneCard	RPL29P12
GeneCard	SPANXC
GeneCard	KCTD16
GeneCard	SIRPG
GeneCard	ZNF720
GeneCard	WDR88
GeneCard	KCTD18
GeneCard	FBXO4
GeneCard	ZNF24
GeneCard	ISOC2
GeneCard	DDX42
GeneCard	TCEAL8
GeneCard	GALNT6
GeneCard	VCY1B
GeneCard	CDPF1
GeneCard	ESYT2
GeneCard	MAGEB18
GeneCard	PHF20L1
GeneCard	SASH3
GeneCard	CCDC15
GeneCard	COMMD3
GeneCard	EDRF1
GeneCard	FBXW8
GeneCard	NCEH1
GeneCard	TTC33
GeneCard	ZNF281
GeneCard	MORF4L2
GeneCard	KIAA1143
GeneCard	LENG8
GeneCard	JHY
GeneCard	PLEKHJ1
GeneCard	CHAC2
GeneCard	HINT3
GeneCard	C3orf33
GeneCard	LRRC42
GeneCard	SPANXN3
GeneCard	ORM2
GeneCard	SPINT3
GeneCard	ILKAP
GeneCard	OBI1
GeneCard	LRATD1
GeneCard	FBXW9
GeneCard	ZNF35
GeneCard	PPTC7
GeneCard	APMAP
GeneCard	BTF3
GeneCard	PRSS33
GeneCard	NUP62CL
GeneCard	ANKRD36BP1
GeneCard	ASB2
GeneCard	PDZD4

GeneCard	ASB9
GeneCard	SLU7
GeneCard	KRTAP3-1
GeneCard	PROSER2
GeneCard	CDC37L1
GeneCard	KLHL23
GeneCard	LURAP1
GeneCard	OR7A5
GeneCard	PPM1N
GeneCard	R3HDM4
GeneCard	OR4S1
GeneCard	TRIQK
GeneCard	GOLGA6L3
GeneCard	LINC00634
GeneCard	FOXO3B
GeneCard	LINC00158
GeneCard	USP32P2
GeneCard	MYO16-AS1
GeneCard	SNORD23
GeneCard	SNORD35A
GeneCard	SNORD67
GeneCard	WAC-AS1
GeneCard	LINC00564
GeneCard	LINC00692
GeneCard	LINC01460
GeneCard	LINC00348
GeneCard	LINC01181
GeneCard	SNORD111
GeneCard	SEC24B-AS1
GeneCard	ZBED5-AS1
GeneCard	KDM4A-AS1
GeneCard	HMGA2-AS1
GeneCard	HID1-AS1
GeneCard	FAM172BP
GeneCard	LINC01579
GeneCard	LINC01222
GeneCard	LINC00424
GeneCard	LINC00457
GeneCard	LINC01023
GeneCard	LINC01151
GeneCard	LINC01208
GeneCard	PCOLCE-AS1
GeneCard	LOC100506178
GeneCard	CDC42-IT1
GeneCard	CETN4P
GeneCard	LINC02268
GeneCard	LINC02532
GeneCard	RPL17P39
GeneCard	STAU2-AS1
GeneCard	LOC100506606
GeneCard	A2ML1-AS2
GeneCard	HNRNPKP3
GeneCard	HYI-AS1
GeneCard	LINC01801
GeneCard	LINC00377
GeneCard	LINC02044
GeneCard	LINC02150
GeneCard	LINC02204

GeneCard	MIR548Y
GeneCard	MIR548AB
GeneCard	LINC02325
GeneCard	MAP3K2-DT
GeneCard	LINC02762
GeneCard	LINC01445
GeneCard	LINC02518
GeneCard	LINC02716
GeneCard	MUC12-AS1
GeneCard	RALGAPA1P1
GeneCard	SMG1P1
GeneCard	ZNF30-AS1
GeneCard	LOC101926964
GeneCard	LOC101928651
GeneCard	LOC101928725
GeneCard	D21S2088E
GeneCard	EDIL3-DT
GeneCard	CHN2-AS1
GeneCard	GALNT7-DT
GeneCard	LHX1-DT
GeneCard	LINC01960
GeneCard	LINC02181
GeneCard	LINC02233
GeneCard	LINC02254
GeneCard	OR7E89P
GeneCard	MIR4531
GeneCard	LINC02763
GeneCard	LINC01712
GeneCard	LINC02535
GeneCard	LINC02653
GeneCard	MFSD13B
GeneCard	TMCC1-DT
GeneCard	RPL3P4
GeneCard	TMEM202-AS1
GeneCard	RNU4-8P
GeneCard	NOP53-AS1
GeneCard	ENSG00000260306
GeneCard	BSG-AS1
GeneCard	BICRA-AS1
GeneCard	ACSL3-AS1
GeneCard	HMGB2P1
GeneCard	GRAPLDR
GeneCard	LINC01965
GeneCard	LINC02008
GeneCard	LINC02177
GeneCard	LINC02205
GeneCard	LINC02214
GeneCard	NEPRO-AS1
GeneCard	OR7E38P
GeneCard	NR4A1AS
GeneCard	MIR6745
GeneCard	LINC01326
GeneCard	LINC01838
GeneCard	LINC02343
GeneCard	LINC02668
GeneCard	LINC01243
GeneCard	MED8-AS1
GeneCard	OR7E136P

GeneCard	MADCAM1-AS1
GeneCard	SNORD3P1
GeneCard	RPL13AP7
GeneCard	RNA5SP429
GeneCard	RNA5SP96
GeneCard	RNU4-46P
GeneCard	ENSG00000272505
GeneCard	ENSG00000271155
GeneCard	ENSG00000225407
GeneCard	ENSG00000227531
GeneCard	ENSG00000235979
GeneCard	ENSG00000238279
GeneCard	ENSG00000251034
GeneCard	ENSG00000253851
GeneCard	ENSG00000251148
GeneCard	ENSG00000251171
GeneCard	LOC100507472
GeneCard	ENSG00000253965
GeneCard	ENSG00000254632
GeneCard	ENSG00000260973
GeneCard	ENSG00000256083
GeneCard	ENSG00000261000
GeneCard	ENSG00000228005
GeneCard	ENSG00000232325
GeneCard	ENSG00000247970
GeneCard	ENSG00000266289
GeneCard	ENSG00000266521
GeneCard	CTNNA1P1
GeneCard	HMGB1P6
GeneCard	KHDRBS2-OT1
GeneCard	LINC01789
GeneCard	LINC02335
GeneCard	RNU6-1216P
GeneCard	RNU6-786P
GeneCard	RNU6ATAC22P
GeneCard	RPL7P19
GeneCard	RPS18P12
GeneCard	SLC25A5P3
GeneCard	RPL35AP4
GeneCard	RPL6P13
GeneCard	SNX9-AS1
GeneCard	RN7SL44P
GeneCard	RNU1-80P
GeneCard	ENSG00000177725
GeneCard	ENSG00000199713
GeneCard	ENSG00000270060
GeneCard	ENSG00000269807
GeneCard	ENSG00000268746
GeneCard	ENSG00000227554
GeneCard	ENSG00000230606
GeneCard	ENSG00000232692
GeneCard	ENSG00000250623
GeneCard	ENSG00000251205
GeneCard	ENSG00000253200
GeneCard	ENSG00000254002
GeneCard	ENSG00000253471
GeneCard	LOC101928804
GeneCard	LOC101928519

GeneCard	LOC105377622
GeneCard	ENSG00000257509
GeneCard	ENSG00000259925
GeneCard	ENSG00000267139
GeneCard	ENSG00000261187
GeneCard	ENSG00000267138
GeneCard	ENSG00000260482
GeneCard	ENSG00000260681
GeneCard	ENSG00000227681
GeneCard	ENSG00000266602
GeneCard	ENSG00000235612
GeneCard	ENSG00000237076
GeneCard	ENSG00000249631
GeneCard	ENSG00000125695
GeneCard	E2F6P1
GeneCard	ARHGAP16P
GeneCard	ATP8A2P3
GeneCard	HSPA8P5
GeneCard	LINC02165
GeneCard	OR7E59P
GeneCard	LINC01868
GeneCard	MIR7154
GeneCard	LINC02307
GeneCard	LINC02460
GeneCard	LINC02503
GeneCard	LINC02651
GeneCard	LINC02752
GeneCard	LINC02699
GeneCard	RAC1P2
GeneCard	PPIAP17
GeneCard	PPIAP1
GeneCard	OR7E109P
GeneCard	RNU6-678P
GeneCard	RNU6-679P
GeneCard	RNU6-67P
GeneCard	PRPF19P1
GeneCard	RPL5P26
GeneCard	TRIM51CP
GeneCard	RNU2-52P
GeneCard	RPL21P29
GeneCard	RPL34P29
GeneCard	RPL32P16
GeneCard	RPS27P10
GeneCard	RNU6-362P
GeneCard	RNU6-412P
GeneCard	RNU6-432P
GeneCard	RNA5SP349
GeneCard	PHBP8
GeneCard	ENSG00000200506
GeneCard	ENSG00000272789
GeneCard	ENSG00000268093
GeneCard	ENSG00000270403
GeneCard	ENSG00000272008
GeneCard	ENSG00000224731
GeneCard	ENSG00000228737
GeneCard	ENSG00000229502
GeneCard	ENSG00000232693
GeneCard	ENSG00000237321

GeneCard	ENSG00000253238
GeneCard	ENSG00000267764
GeneCard	LOC107984124
GeneCard	ENSG00000254420
GeneCard	ENSG00000255447
GeneCard	ENSG00000254340
GeneCard	ENSG00000257553
GeneCard	ENSG00000260672
GeneCard	ENSG00000263567
GeneCard	ENSG00000267215
GeneCard	ENSG00000267205
GeneCard	ENSG00000233021
GeneCard	ENSG00000259744
GeneCard	ENSG00000259818
GeneCard	ENSG00000230944
GeneCard	ENSG00000237074
GeneCard	DPPA3P3
GeneCard	DYNLL1P4
GeneCard	DNAJA1P1
GeneCard	ARL2BPP7
GeneCard	ATP5MFP3
GeneCard	CSTP2
GeneCard	IRF5P1
GeneCard	HMGB3P23
GeneCard	HMGN2P39
GeneCard	GPS2P1
GeneCard	HIGD1AP3
GeneCard	HMGB3P12
GeneCard	HPRT1P2
GeneCard	DSTNP5
GeneCard	KRT18P45
GeneCard	OR7E39P
GeneCard	NMNAT1P2
GeneCard	MTND5P15
GeneCard	MTND2P9
GeneCard	MTND3P4
GeneCard	MEMO1P3
GeneCard	RABEPKP1
GeneCard	RANP7
GeneCard	RBBP4P4
GeneCard	RAD23BP2
GeneCard	R3HDM2P2
GeneCard	LYPLA1P1
GeneCard	RNU7-156P
GeneCard	RNU6-1200P
GeneCard	RNU7-106P
GeneCard	RPL6P16
GeneCard	RPS20P10
GeneCard	SLC25A6P5
GeneCard	SEC63P2
GeneCard	RPL34P16
GeneCard	RPL34P19
GeneCard	RN7SKP120
GeneCard	RN7SKP122
GeneCard	RN7SKP141
GeneCard	RNA5SP208
GeneCard	RNA5SP228
GeneCard	RNU6-1037P

GeneCard	RN7SL221P
GeneCard	RN7SL271P
GeneCard	RNA5SP140
GeneCard	RN7SL782P
GeneCard	RNU1-23P
GeneCard	RNU4-77P
GeneCard	RNU6-1060P
GeneCard	RPL31P32
GeneCard	RPL23AP44
GeneCard	RPSAP24
GeneCard	PCNPP2
GeneCard	RNU6-261P
GeneCard	RNU6-276P
GeneCard	RNU6-280P
GeneCard	RNU6-478P
GeneCard	RNU7-65P
GeneCard	RN7SL152P
GeneCard	RN7SL825P
GeneCard	RNU2-9P
GeneCard	SMARCE1P4
GeneCard	VDAC1P12
GeneCard	LOC107985953
GeneCard	UNC93B4
GeneCard	ENSG00000285885
GeneCard	ENSG00000286746
GeneCard	ENSG00000268926
GeneCard	ENSG00000273253
GeneCard	ENSG00000226669
GeneCard	ENSG00000235410
GeneCard	ENSG00000252727
GeneCard	ENSG00000253678
GeneCard	LOC105375112
GeneCard	LOC105375116
GeneCard	ZFYVE9P2
GeneCard	lnc-MCTP2-7
GeneCard	lnc-MRPL39-2
GeneCard	lnc-LY86-10
GeneCard	lnc-MOBP-2
GeneCard	lnc-ADCY10-2
GeneCard	lnc-MS4A6A-1
GeneCard	lnc-ZNF320-1
GeneCard	lnc-WDR1-3
GeneCard	LOC105378318
GeneCard	ENSG00000231291
GeneCard	ENSG00000242611
GeneCard	ENSG00000235023
GeneCard	ENSG00000234648
GeneCard	ENSG00000267576
GeneCard	ENSG00000201830
GeneCard	RF00017-6416
GeneCard	BNIP3P13
GeneCard	CIR1P1
GeneCard	ATP5PDP3
GeneCard	AP1S2P1
GeneCard	GPR160P2
GeneCard	GTF2IP11
GeneCard	H3P28
GeneCard	HSPA8P9

GeneCard	NRBF2P3
GeneCard	MARK2P15
GeneCard	MFSD4BP1
GeneCard	PKMP4
GeneCard	NFU1P1
GeneCard	NACAP6
GeneCard	MTCO3P30
GeneCard	RPEP2
GeneCard	SNRPGP16
GeneCard	RFC5P1
GeneCard	RNU6-1103P
GeneCard	RNU6-248P
GeneCard	RNU6-363P
GeneCard	RNU6-637P
GeneCard	SUCLG2P4
GeneCard	ENSG00000277654
GeneCard	HSALNG0004778
GeneCard	HSALNG0017165
GeneCard	HSALNG0030571
GeneCard	HSALNG0030569-002
GeneCard	ENSG00000286505
GeneCard	ENSG00000288100
GeneCard	HE855971
GeneCard	HSALNG0001373
GeneCard	HSALNG0039016
GeneCard	ENSG00000280434
GeneCard	ENSG00000285458
GeneCard	ENSG00000287449
GeneCard	ENSG00000287883
GeneCard	FJ601684-123
GeneCard	HSALNG0004004
GeneCard	HSALNG0035679
GeneCard	HSALNG0045314
GeneCard	ENSG00000251411
GeneCard	LOC100419574
GeneCard	LOC105371762
GeneCard	lnc-AHR-8
GeneCard	lnc-ARVCF-1
GeneCard	lnc-B9D1-3
GeneCard	lnc-CASTOR3-3
GeneCard	lnc-CBX4-4
GeneCard	lnc-CDR2-7
GeneCard	lnc-CMTR1-5
GeneCard	lnc-CUTA-2
GeneCard	lnc-CYP1B1-7
GeneCard	lnc-HAUS6-3
GeneCard	lnc-HEMGN-2
GeneCard	lnc-IL19-1
GeneCard	lnc-IPO5-10
GeneCard	lnc-KRT6A-1
GeneCard	lnc-LDLRAD2-2
GeneCard	lnc-PALLD-4
GeneCard	lnc-PDGFA-13
GeneCard	lnc-PLCG1-2
GeneCard	lnc-ACTA2-2
GeneCard	lnc-APOBEC4-5
GeneCard	lnc-C9orf152-4
GeneCard	lnc-COTL1-3

GeneCard	lnc-F13A1-5
GeneCard	lnc-GEN1-1
GeneCard	lnc-GNB1L-1
GeneCard	lnc-HEMGN-3
GeneCard	lnc-HMGA2-1
GeneCard	lnc-HYI-6
GeneCard	lnc-KCNV2-1
GeneCard	lnc-KCNV2-5
GeneCard	lnc-LIMS2-1
GeneCard	lnc-LRRC32-7
GeneCard	lnc-PLIN2-1
GeneCard	L13304-036
GeneCard	lnc-CDR2-8
GeneCard	lnc-COL21A1-8
GeneCard	lnc-DEPDC1B-5
GeneCard	lnc-DYDC1-6
GeneCard	lnc-FAM83E-2
GeneCard	lnc-JAM2-1
GeneCard	lnc-LTC4S-2
GeneCard	lnc-NDUFS6-14
GeneCard	lnc-NFIA-2
GeneCard	lnc-OVOL2-1
GeneCard	HSALNG0128918
GeneCard	L13304-007
GeneCard	lnc-ACE-5
GeneCard	lnc-CHP2-1
GeneCard	lnc-CMTR1-4
GeneCard	lnc-FER-17
GeneCard	lnc-FOXE1-1
GeneCard	lnc-GFPT1-3
GeneCard	lnc-H2BFS-3
GeneCard	lnc-LRRC49-15
GeneCard	lnc-MFAP5-4
GeneCard	lnc-PDPK1-2
GeneCard	HSALNG0057935
GeneCard	HSALNG0082715
GeneCard	HSALNG0091189
GeneCard	HSALNG0091190
GeneCard	HSALNG0106229
GeneCard	HSALNG0106440
GeneCard	HSALNG0106914-002
GeneCard	HSALNG0122890
GeneCard	HSALNG0122893
GeneCard	HSALNG0054569
GeneCard	HSALNG0084446
GeneCard	HSALNG0087985
GeneCard	HSALNG0120735
GeneCard	HSALNG0091187
GeneCard	HSALNG0092004
GeneCard	HSALNG0092135-002
GeneCard	HSALNG0108347
GeneCard	HSALNG0067505-001
GeneCard	HSALNG0068231
GeneCard	HSALNG0082051
GeneCard	HSALNG0103565
GeneCard	HSALNG0107836
GeneCard	lnc-RNF113B-5
GeneCard	lnc-RPL39L-3

GeneCard	lnc-RPS27L-10
GeneCard	lnc-RUBCN-8
GeneCard	lnc-SIGLEC11-2
GeneCard	lnc-SLC1A1-11
GeneCard	lnc-SMARCC2-7-001
GeneCard	lnc-SMARCC2-7-002
GeneCard	lnc-SRCIN1-4
GeneCard	lnc-TM2D3-2
GeneCard	lnc-TXNRD2-3
GeneCard	NONHSAG044482.2
GeneCard	piR-31961
GeneCard	piR-32677-423
GeneCard	lnc-RUBCN-7
GeneCard	lnc-SUZ12-8
GeneCard	lnc-TMEM232-7
GeneCard	lnc-TSPAN4-2
GeneCard	lnc-USP48-7
GeneCard	MN308698
GeneCard	NONHSAG024474.2
GeneCard	lnc-RIPOR3-9
GeneCard	lnc-RPL18-1
GeneCard	lnc-SDR42E2-1
GeneCard	lnc-SLC15A1-1
GeneCard	lnc-TSEN54-2
GeneCard	NONHSAG008515.2
GeneCard	NONHSAG024340.2
GeneCard	RF00017-1938
GeneCard	RF00017-2539
GeneCard	RF00017-4344
GeneCard	RF00017-5161
GeneCard	piR-57133-321
GeneCard	RF00017-253
GeneCard	RF00017-4549
GeneCard	lnc-SLC25A19-4
GeneCard	lnc-SMIM15-3
GeneCard	lnc-SMPD3-4
GeneCard	lnc-TANGO2-1
GeneCard	lnc-USP35-19
GeneCard	MN309334
GeneCard	NONHSAG024295.2
GeneCard	NONHSAG031352.2
GeneCard	piR-44909
GeneCard	RF00017-5020
GeneCard	piR-54178
GeneCard	piR-61532-071
GeneCard	RF00017-2801
GeneCard	RF00017-4575
GeneCard	RF00017-4927
GeneCard	RF00017-5019
GeneCard	ENSG00000237493
GeneCard	ENSG00000244313
GeneCard	lnc-RINT1-6
GeneCard	lnc-RCL1-2
GeneCard	RF00017-6042
GeneCard	RF00017-6045
GeneCard	RF00017-6048
GeneCard	RF00017-6089
GeneCard	RF00017-6092

GeneCard	RF00017-6093
GeneCard	RF00017-6417
GeneCard	RF00017-7260
GeneCard	RF00017-6818
GeneCard	RF00017-7258
GeneCard	RF00017-7259
GeneCard	DNAAF11P1
GeneCard	PPIAP63
GeneCard	PPIAP79
GeneCard	MTATP6P30
GeneCard	MTDHP1
GeneCard	SMC4P1
GeneCard	LOC105374206
GeneCard	FJ601678-049
GeneCard	HE856151
GeneCard	HSALNG0006263-001
GeneCard	HSALNG0006264
GeneCard	HSALNG0013263-002
GeneCard	ENSG00000276729
GeneCard	HSALNG0001758
GeneCard	HSALNG0013263-003
GeneCard	HSALNG0013263-004
GeneCard	HSALNG0032854
GeneCard	HSALNG0045311
GeneCard	HSALNG0001292
GeneCard	HSALNG0013833
GeneCard	HSALNG0037984
GeneCard	HSALNG0046013
GeneCard	HSALNG0001290
GeneCard	HSALNG0001541
GeneCard	HSALNG0035678
GeneCard	HSALNG0038574-002
GeneCard	HSALNG0042793
GeneCard	ENSG00000248621
GeneCard	LOC105372878
GeneCard	HSALNG0136094
GeneCard	HSALNG0136266
GeneCard	lnc-CD24-3
GeneCard	lnc-CEMP1-10
GeneCard	lnc-EFCAB10-2
GeneCard	lnc-ERCC6L2-7
GeneCard	lnc-KCNJ3-7
GeneCard	lnc-KIF5B-1
GeneCard	lnc-LIMD2-1
GeneCard	lnc-NRN1-1
GeneCard	HSALNG0126886
GeneCard	HSALNG0126889
GeneCard	HSALNG0130766
GeneCard	lnc-BAMBI-3
GeneCard	lnc-C12orf49-2
GeneCard	lnc-CDH6-18
GeneCard	lnc-ERO1A-2
GeneCard	lnc-MED8-4
GeneCard	lnc-PRKD3-2
GeneCard	lnc-CLEC2L-4
GeneCard	lnc-DOC2A-1
GeneCard	lnc-FGB-3
GeneCard	HSALNG0050293

GeneCard	HSALNG0055489
GeneCard	HSALNG0059742
GeneCard	HSALNG0068112-002
GeneCard	HSALNG0068112-003
GeneCard	HSALNG0072180
GeneCard	HSALNG0091496
GeneCard	HSALNG0092289-001
GeneCard	HSALNG0092289-002
GeneCard	HSALNG0099281
GeneCard	HSALNG0099369
GeneCard	HSALNG0106230
GeneCard	HSALNG0107002
GeneCard	HSALNG0113154
GeneCard	HSALNG0117999
GeneCard	HSALNG0050296
GeneCard	HSALNG0060082
GeneCard	HSALNG0075491
GeneCard	HSALNG0084042
GeneCard	HSALNG0086184
GeneCard	HSALNG0099280
GeneCard	HSALNG0114686
GeneCard	HSALNG0115121
GeneCard	HSALNG0115122
GeneCard	HSALNG0118739
GeneCard	HSALNG0120734
GeneCard	HSALNG0085883-002
GeneCard	HSALNG0092135-001
GeneCard	HSALNG0096321-001
GeneCard	HSALNG0099368
GeneCard	HSALNG0103563
GeneCard	HSALNG0108635
GeneCard	HSALNG0113155
GeneCard	HSALNG0116176-001
GeneCard	HSALNG0116176-002
GeneCard	HSALNG0118743
GeneCard	HSALNG0119075
GeneCard	HSALNG0123261
GeneCard	HSALNG0099372
GeneCard	HSALNG0106942-001
GeneCard	HSALNG0117163
GeneCard	HSALNG0117164
GeneCard	HSALNG0118742
GeneCard	lnc-RPL17-3
GeneCard	lnc-TMC5-2
GeneCard	MK280452
GeneCard	NONHSAG014173.2
GeneCard	NONHSAG031788.2
GeneCard	piR-38051-039
GeneCard	piR-38351-103
GeneCard	lnc-SYK-9
GeneCard	lnc-ZHX2-3
GeneCard	MK280180
GeneCard	piR-31937-019
GeneCard	piR-32214-246
GeneCard	piR-39701-006
GeneCard	lnc-SLC19A1-4
GeneCard	lnc-TEX33-1
GeneCard	lnc-TMEM242-11

GeneCard	lnc-WDR33-2
GeneCard	MG828622
GeneCard	MN298678-098
GeneCard	NONHSAG042005.2
GeneCard	piR-31286-002
GeneCard	piR-32214-368
GeneCard	piR-32214-557
GeneCard	piR-38736-015
GeneCard	piR-40881
GeneCard	piR-45035-144
GeneCard	piR-49097-104
GeneCard	piR-52464
GeneCard	piR-55985-053
GeneCard	piR-57981
GeneCard	piR-43105-107
GeneCard	piR-53823
GeneCard	piR-57176-535
GeneCard	piR-60246-059
GeneCard	RF00017-5327
GeneCard	RF00017-5429
GeneCard	MN297551
GeneCard	piR-31210-009
GeneCard	piR-38423-054
GeneCard	piR-42625-021
GeneCard	piR-47628-005
GeneCard	piR-56037-012
GeneCard	piR-57712
GeneCard	RF00017-4093
GeneCard	piR-48218-001
GeneCard	piR-49402
GeneCard	piR-55985-101
GeneCard	piR-55985-185
GeneCard	piR-60129
GeneCard	piR-60898-001
GeneCard	piR-61687-001
GeneCard	RF00017-2498
GeneCard	RF00017-4770
GeneCard	LOC107984312
GeneCard	LOC107986930
GeneCard	ENSG00000248196
GeneCard	ENSG00000249200
GeneCard	HSALNG0047025-001
GeneCard	HSALNG0047821
GeneCard	piR-41245-221
GeneCard	RF00017-564
GeneCard	RF00017-6667
GeneCard	RF00066-159
GeneCard	RF00017-6972
GeneCard	LOC107985536
GeneCard	LOC107986178
GeneCard	LOC105378208
GeneCard	LOC105378209
GeneCard	LOC107984380
GeneCard	LOC107986016
GeneCard	LOC105373581
GeneCard	ENSG00000280149
GeneCard	HSALNG0017166
GeneCard	HSALNG0030524

GeneCard	HSALNG0044617-002
GeneCard	ENSG00000279953
GeneCard	HSALNG0001004
GeneCard	HSALNG0005998
GeneCard	HSALNG0006000
GeneCard	HSALNG0008434
GeneCard	HSALNG0012640
GeneCard	HSALNG0016296
GeneCard	HSALNG0028435
GeneCard	HSALNG0030525
GeneCard	HSALNG0037499
GeneCard	HSALNG0037885
GeneCard	FJ601678-137
GeneCard	HSALNG0001291
GeneCard	HSALNG0023909
GeneCard	HSALNG0028082
GeneCard	HSALNG0029071-002
GeneCard	LOC105375964
GeneCard	HSALNG0017614
GeneCard	HSALNG0020420
GeneCard	HSALNG0032032
GeneCard	HSALNG0044328
GeneCard	LOC105375328
GeneCard	LOC105372106
GeneCard	HSALNG0132503
GeneCard	lnc-COLGALT2-6
GeneCard	lnc-NEPRO-5
GeneCard	HSALNG0126787
GeneCard	HSALNG0130152
GeneCard	HSALNG0132429
GeneCard	hsa-miR-5095-477
GeneCard	lnc-KLHL1-11
GeneCard	lnc-MAP3K3-1
GeneCard	HSALNG0132428
GeneCard	hsa-miR-5095-065
GeneCard	lnc-LRP4-2
GeneCard	lnc-OCA2-4
GeneCard	HSALNG0133645
GeneCard	HSALNG0134037
GeneCard	lnc-PHF23-1
GeneCard	HSALNG0052523
GeneCard	HSALNG0055490
GeneCard	HSALNG0059720
GeneCard	HSALNG0067487
GeneCard	HSALNG0068116
GeneCard	HSALNG0069658
GeneCard	HSALNG0072660
GeneCard	HSALNG0073635
GeneCard	HSALNG0094761
GeneCard	HSALNG0098206-002
GeneCard	HSALNG0104806
GeneCard	HSALNG0109037
GeneCard	HSALNG0118978
GeneCard	HSALNG0121848
GeneCard	HSALNG0050465
GeneCard	HSALNG0052525
GeneCard	HSALNG0054175
GeneCard	HSALNG0054198

GeneCard	HSALNG0062068-001
GeneCard	HSALNG0062068-002
GeneCard	HSALNG0064190
GeneCard	HSALNG0066498
GeneCard	HSALNG0068674
GeneCard	HSALNG0078213
GeneCard	HSALNG0097694
GeneCard	HSALNG0108634
GeneCard	HSALNG0054182
GeneCard	HSALNG0056444
GeneCard	HSALNG0063304
GeneCard	HSALNG0068120
GeneCard	HSALNG0076995-002
GeneCard	HSALNG0085885
GeneCard	HSALNG0092010
GeneCard	HSALNG0094181
GeneCard	HSALNG0094762
GeneCard	HSALNG0098847
GeneCard	HSALNG0108448
GeneCard	HSALNG0113015
GeneCard	HSALNG0119076
GeneCard	HSALNG0050140
GeneCard	HSALNG0059719
GeneCard	HSALNG0064554
GeneCard	HSALNG0068673
GeneCard	HSALNG0069754
GeneCard	HSALNG0073152
GeneCard	HSALNG0073676-002
GeneCard	HSALNG0073677
GeneCard	HSALNG0076995-001
GeneCard	HSALNG0083953
GeneCard	HSALNG0100157
GeneCard	HSALNG0121850
GeneCard	lnc-SERPINI1-13
GeneCard	lnc-TTLL7-10
GeneCard	MN309174-341
GeneCard	piR-38051-017
GeneCard	piR-38580-356
GeneCard	lnc-TMEM232-6
GeneCard	lnc-VSNL1-6
GeneCard	lnc-VSNL1-1
GeneCard	MN308956
GeneCard	piR-32023-213
GeneCard	piR-41195-093
GeneCard	lnc-RNF113B-7
GeneCard	lnc-RSPH1-6
GeneCard	lnc-SGPP2-1
GeneCard	piR-30561
GeneCard	piR-31534-512
GeneCard	piR-39279-038
GeneCard	piR-39655-006
GeneCard	piR-39701-059
GeneCard	piR-42602
GeneCard	piR-43581
GeneCard	piR-56580-048
GeneCard	piR-57461-271
GeneCard	piR-58557-058
GeneCard	piR-60246-038

GeneCard	piR-61028-186
GeneCard	RF00017-200
GeneCard	RF00017-4477
GeneCard	piR-42491-026
GeneCard	piR-42799-010
GeneCard	piR-43325-085
GeneCard	piR-54625
GeneCard	piR-54764-557
GeneCard	piR-56759-398
GeneCard	piR-61101-030
GeneCard	RF00017-1320
GeneCard	RF00017-2296
GeneCard	RF00017-5470
GeneCard	lnc-ZNRF4-2
GeneCard	MK280607-105
GeneCard	MN298644
GeneCard	piR-31199-348
GeneCard	piR-38426-008
GeneCard	piR-38579-041
GeneCard	piR-42491-133
GeneCard	piR-53431-310
GeneCard	piR-57460-157
GeneCard	RF00017-4424
GeneCard	piR-44878-019
GeneCard	piR-50444-066
GeneCard	piR-59928-064
GeneCard	RF00017-5430
GeneCard	LOC105369515
GeneCard	LOC105370356
GeneCard	LOC105371028
GeneCard	LOC105369782
GeneCard	piR-41880-009
GeneCard	LOC105379188
GeneCard	LOC107984408
GeneCard	LOC105375532
GeneCard	LOC105372492
GeneCard	LOC105376219
GeneCard	LOC105377124
GeneCard	ENSG00000257680
GeneCard	LOC107987301
GeneCard	HSALNG0047025-002
GeneCard	lnc-RCL1-3
GeneCard	piR-41405-104
GeneCard	RF00017-6443
GeneCard	RF00017-5623
GeneCard	RF00017-567
GeneCard	RF00017-6094
GeneCard	RF00026-940
GeneCard	RF00017-6672
GeneCard	RF00017-765
GeneCard	RF01960-002
GeneCard	RF00619-032
GeneCard	RF00951-070
GeneCard	HSALNG0042079
GeneCard	HSALNG0011480
GeneCard	HSALNG0024759
GeneCard	HSALNG0028923
GeneCard	HSALNG0029071-001

GeneCard	LOC105375411
GeneCard	LOC100419959
GeneCard	LOC105373902
GeneCard	L13714-244
GeneCard	HSALNG0062059
GeneCard	HSALNG0096321-002
GeneCard	HSALNG0062201
GeneCard	HSALNG0084152
GeneCard	HSALNG0072676
GeneCard	MK280073-483
GeneCard	piR-31926-034
GeneCard	piR-34822-265
GeneCard	piR-38257-008
GeneCard	piR-60051-026
GeneCard	RF00001-225
GeneCard	piR-38732-017
GeneCard	piR-39858-529
GeneCard	piR-52261-220
GeneCard	RF00017-3779
GeneCard	LOC101929756
GeneCard	LOC102724749
GeneCard	LOC112268049
GeneCard	LGALS14
GeneCard	CAMK1G
GeneCard	RBM46
GeneCard	SNAPC3
GeneCard	RINL
GeneCard	PTPN7
GeneCard	ZSCAN18
GeneCard	C4orf46
GeneCard	NXPE3
GeneCard	RPUSD1
GeneCard	THOC1
GeneCard	C1orf116
GeneCard	COL18A1-AS1
GeneCard	C18orf21
GeneCard	LRRC45
GeneCard	SPATA12
GeneCard	PRR16
GeneCard	CHURC1
GeneCard	ZSCAN1
GeneCard	C11orf52
GeneCard	DPY19L2P1
GeneCard	ZFYVE21
GeneCard	EVA1A
GeneCard	ZFP2
GeneCard	GPR52
GeneCard	FPR3
GeneCard	XAF1
GeneCard	LRRC15
GeneCard	ZNF706
GeneCard	ZC3HAV1L
GeneCard	ABHD17C
GeneCard	NRN1L
GeneCard	MAGEB6
GeneCard	TYRL
GeneCard	ST8SIA6
GeneCard	THUMPD2

GeneCard	EIF1B
GeneCard	CYTIP
GeneCard	ZNF75A
GeneCard	KRT36
GeneCard	MOB3A
GeneCard	ODF4
GeneCard	PPIE
GeneCard	LVRN
GeneCard	FFAR3
GeneCard	ZNF329
GeneCard	ZNF345
GeneCard	CAPN12
GeneCard	TMEM95
GeneCard	MGARP
GeneCard	RHOBTB3
GeneCard	FMNL3
GeneCard	ASB13
GeneCard	WSB1
GeneCard	TMEM59L
GeneCard	PTTG1IP
GeneCard	CHST4
GeneCard	MAGEB3
GeneCard	CBWD3
GeneCard	MT1E
GeneCard	PIN4
GeneCard	ARHGAP35
GeneCard	USP32
GeneCard	DTX3
GeneCard	GUSBP3
GeneCard	TMEM115
GeneCard	ZNF175
GeneCard	TBC1D3P2
GeneCard	DENND1C
GeneCard	ACSF2
GeneCard	RALGPS1
GeneCard	RBM11
GeneCard	RNF167
GeneCard	UPP1
GeneCard	DBNDD2
GeneCard	PPP1R16A
GeneCard	ZNF19
GeneCard	DYNLRB1
GeneCard	ZSCAN22
GeneCard	SNX12
GeneCard	PLEKHG7
GeneCard	DPYSL4
GeneCard	SYCE3
GeneCard	GMCL2
GeneCard	LANCL1
GeneCard	SP140L
GeneCard	BFAR
GeneCard	CBX6
GeneCard	SHCBP1L
GeneCard	JADE3
GeneCard	TSSK2
GeneCard	ZNF572
GeneCard	SPATS1
GeneCard	C20orf141

GeneCard	LRRC61
GeneCard	MORN4
GeneCard	CNTNAP4
GeneCard	RAB6D
GeneCard	ZNF670
GeneCard	FASTKD1
GeneCard	GRAMD1C
GeneCard	KLF8
GeneCard	TTC23L
GeneCard	RGS18
GeneCard	ZNF436
GeneCard	OCEL1
GeneCard	UBXN2A
GeneCard	TSSK6
GeneCard	CRCT1
GeneCard	ERP27
GeneCard	RPL18P7
GeneCard	PTPN18
GeneCard	BEND2
GeneCard	HAGHL
GeneCard	EVA1B
GeneCard	ANKRD42
GeneCard	USP5
GeneCard	LOC111255645
GeneCard	RIMKLA
GeneCard	ZNF219
GeneCard	TTC4P1
GeneCard	GPR160
GeneCard	TUBA4B
GeneCard	ARID5A
GeneCard	C19orf25
GeneCard	RPL23AP28
GeneCard	CCDC144NL
GeneCard	PLAAT1
GeneCard	NMRAL1
GeneCard	RXFP3
GeneCard	C6orf141
GeneCard	FAM221A
GeneCard	FDPSP4
GeneCard	ZNF649
GeneCard	SH3BP5L
GeneCard	COMMD4
GeneCard	SPIN2B
GeneCard	ZNF286A
GeneCard	UBBP1
GeneCard	FSD1L
GeneCard	MIR644A
GeneCard	HMGN2
GeneCard	DHRS1
GeneCard	SPATA2L
GeneCard	LETM2
GeneCard	CPED1
GeneCard	EBF4
GeneCard	UBXN8
GeneCard	CPA2
GeneCard	HSFY2
GeneCard	ZMYND12
GeneCard	FAM78A

GeneCard	HAUS7
GeneCard	FAM53A
GeneCard	HSPA13
GeneCard	METTL7A
GeneCard	PHOSPHO2
GeneCard	ZRSR2P1
GeneCard	BEND5
GeneCard	G2E3
GeneCard	CCDC9
GeneCard	TRIM49
GeneCard	CDRT15P3
GeneCard	TMEM25
GeneCard	APOL6
GeneCard	CSNK1G3
GeneCard	OSCP1
GeneCard	SERTM1
GeneCard	PWAR5
GeneCard	PRPF39
GeneCard	ZNF639
GeneCard	CLDN20
GeneCard	LRRN2
GeneCard	FAM126B
GeneCard	NAP1L3
GeneCard	LINC00518
GeneCard	ZNF138
GeneCard	CCNJL
GeneCard	ZNF419
GeneCard	GPR61
GeneCard	LINC00663
GeneCard	LOC101927502
GeneCard	TMEM35A
GeneCard	OR2W1
GeneCard	GPR6
GeneCard	ZC3H3
GeneCard	STMN4
GeneCard	SSU72
GeneCard	TMEM54
GeneCard	UBOX5
GeneCard	RPL21P108
GeneCard	FAM104A
GeneCard	NAP1L5
GeneCard	ZCCHC2
GeneCard	ASAP3
GeneCard	C1orf216
GeneCard	MIR3130-1
GeneCard	ZNF57
GeneCard	RHOXF2
GeneCard	CRYBG3
GeneCard	BIN2
GeneCard	JTB
GeneCard	FAM72B
GeneCard	C6orf201
GeneCard	CLIP3
GeneCard	NACAD
GeneCard	ZNF524
GeneCard	TMEM121
GeneCard	TPRXL
GeneCard	TM9SF1

GeneCard	HMGB4
GeneCard	UBE2DNL
GeneCard	NMRK1
GeneCard	SPRR4
GeneCard	TENM3-AS1
GeneCard	TMEM141
GeneCard	CRYBG2
GeneCard	TEX37
GeneCard	RPS17P5
GeneCard	HSPA7
GeneCard	GPR75
GeneCard	GPX1P2
GeneCard	CMTM2
GeneCard	FAM200A
GeneCard	YAE1
GeneCard	CFAP299
GeneCard	BEX5
GeneCard	TSACC
GeneCard	GPCPD1
GeneCard	TPD52L3
GeneCard	ANKRD45
GeneCard	LSMEM1
GeneCard	SULT1B1
GeneCard	SERPINA13P
GeneCard	MIR2113
GeneCard	C11orf53
GeneCard	ZNF124
GeneCard	EMC6
GeneCard	SLFNL1
GeneCard	CCDC149
GeneCard	TARP
GeneCard	PHLDA3
GeneCard	OSTCP1
GeneCard	ECI1
GeneCard	ZNF497
GeneCard	C11orf71
GeneCard	PEAK1
GeneCard	LCE2A
GeneCard	EIF4A1P6
GeneCard	PCSK4
GeneCard	RNU7-24P
GeneCard	WFDC5
GeneCard	FAAH2
GeneCard	RPS3AP1
GeneCard	THEG
GeneCard	OR1E1
GeneCard	RPSAP20
GeneCard	CPSF1P1
GeneCard	RPL17P37
GeneCard	RPL31P36
GeneCard	UBE2D3P2
GeneCard	SMIM11
GeneCard	SIAH3
GeneCard	H2AC6
GeneCard	RASL11B
GeneCard	C19orf18
GeneCard	TEX19
GeneCard	CRYAA2

GeneCard	DLSTP1
GeneCard	ARL5A
GeneCard	C8orf48
GeneCard	C2orf15
GeneCard	DENND6B
GeneCard	FRG1BP
GeneCard	FAM74A4
GeneCard	OR4D6
GeneCard	PP2D1
GeneCard	BCL2L10
GeneCard	FAM240A
GeneCard	MOGAT3
GeneCard	RNF208
GeneCard	SHLD1
GeneCard	DTD2
GeneCard	RNASE11
GeneCard	TEX44
GeneCard	PNMA6F
GeneCard	RPS7P8
GeneCard	FRMD6-AS2
GeneCard	BDH2P1
GeneCard	RPL5P2
GeneCard	CTXN3
GeneCard	PSME3IP1
GeneCard	TRIML1
GeneCard	PCDHB11
GeneCard	RTP4
GeneCard	LOC343052
GeneCard	THAP6
GeneCard	LOC642487
GeneCard	RNF151
GeneCard	C11orf45
GeneCard	LSMEM2
GeneCard	TSPY2
GeneCard	HSP90AA5P
GeneCard	ZNF200
GeneCard	FAM72A
GeneCard	MT1M
GeneCard	TJP3
GeneCard	PCDHGC5
GeneCard	FITM1
GeneCard	C22orf46
GeneCard	BIN3-IT1
GeneCard	HERC2P9
GeneCard	SLIT1-AS1
GeneCard	LOC403312
GeneCard	DPY19L2P4
GeneCard	NUDT16L2P
GeneCard	LOC254896
GeneCard	IQCF2
GeneCard	FCF1
GeneCard	MIR519B
GeneCard	LINC02085
GeneCard	LRRC66
GeneCard	ANKRD7
GeneCard	DAPP1
GeneCard	EML2
GeneCard	ZNF449

GeneCard	CXorf38
GeneCard	H3-5
GeneCard	PCDHB10
GeneCard	METTL2A
GeneCard	KRTAP19-5
GeneCard	CLUHP3
GeneCard	ADH5P2
GeneCard	SLC16A6
GeneCard	MIR3130-2
GeneCard	GOT1L1
GeneCard	KRTAP13-3
GeneCard	KRTAP8-1
GeneCard	KRTAP19-1
GeneCard	KRTAP19-7
GeneCard	GOLGA2P5
GeneCard	NCBP2AS2
GeneCard	ZHX1-C8orf76
GeneCard	LRRC52-AS1
GeneCard	NOS2P2
GeneCard	MPND
GeneCard	AKR1C8P
GeneCard	TEX55
GeneCard	PTGES2-AS1
GeneCard	ZC3H12B
GeneCard	CCDC54
GeneCard	SLC25A5P2
GeneCard	MIR626
GeneCard	NSG1
GeneCard	TESK1
GeneCard	GOT2P1
GeneCard	MIR5588
GeneCard	SCART1
GeneCard	MRPS33P2
GeneCard	PLEKHA2
GeneCard	RPL31P12
GeneCard	RPL29P29
GeneCard	GSKIP
GeneCard	RPL18P1
GeneCard	DDO
GeneCard	PURG
GeneCard	PTMAP5
GeneCard	RPL21P99
GeneCard	ZNF717
GeneCard	RPL12P34
GeneCard	ARIH2P1
GeneCard	VN1R95P
GeneCard	DISP3
GeneCard	HSPD1P9
GeneCard	RPL9P23
GeneCard	RPSAP43
GeneCard	ARL14
GeneCard	LOC110740340
GeneCard	MEIS3P1
GeneCard	KRT18P24
GeneCard	RPL31P39
GeneCard	KLHL5
GeneCard	C9orf135
GeneCard	PMCHL1

GeneCard	ZNF385A
GeneCard	RPL21P46
GeneCard	SSH2
GeneCard	C16orf78
GeneCard	RPL38P2
GeneCard	RPL22P22
GeneCard	MRPS36P3
GeneCard	FKBP3
GeneCard	ABHD14B
GeneCard	RPL21P43
GeneCard	WDR11-AS1
GeneCard	RRM2P4
GeneCard	RPL27AP8
GeneCard	OR2BH1P
GeneCard	PPIHP2
GeneCard	RPS2P38
GeneCard	UBQLN4P2
GeneCard	ZNF641
GeneCard	RSL24D1P11
GeneCard	PCBP2P3
GeneCard	UNGP2
GeneCard	ATP6V0B
GeneCard	TYMSOS
GeneCard	TERF1P3
GeneCard	MRPS33P3
GeneCard	RPL11P3
GeneCard	RPL4P2
GeneCard	LOC101929279
GeneCard	OR6X1
GeneCard	RACK1P1
GeneCard	GOLGA8M
GeneCard	OR51A1P
GeneCard	RPL17P6
GeneCard	RPL12P24
GeneCard	OR55B1P
GeneCard	ST13P9
GeneCard	RPS25P2
GeneCard	MIR525
GeneCard	ALDH7A1P1
GeneCard	FAM27E3
GeneCard	FOLH1B
GeneCard	ZNF486
GeneCard	MT1XP1
GeneCard	PLEKHF1
GeneCard	OR6B3
GeneCard	PCNX4
GeneCard	TBC1D26
GeneCard	PABPC1P1
GeneCard	ADAM3B
GeneCard	GOLGA8R
GeneCard	DIAPH3-AS2

Supplementary Table 4

The Hub Target Genes
PGR
PTGS1
PTGS2
HSP90AA1
PIK3CG
AKR1B10
JUN
IL13
PPARG
MPL
IL1B
APOA2
GGH
SHMT2
ALOX5
SERPINE1
MTNR1A
MTNR1B
HTR2C
ESR1
RXRA
ADRB2
PRKACA
MAOB
SLC6A4
AR
DPP4
PRSS1
RELA
AKT1
BCL2
FOS
BAX
MMP9
CASP3
TP63
HIF1A
FOSL1
FOSL2
CCNB1
MPO
AHR
IGF2
CYCS
ALOX12
NFATC1
EGLN1
APOD
NOS2
ESR2
MAPK14
GSK3B
CHEK1
CCNA2
KCNH2
DRD1

CHRM3
CHRM1
CHRM4
ADRA1A
CHRM2
CHRNA2
OPRM1
CHRNA7
CASP9
CASP8
PRKCA
PON1
MAP2
NR3C2
CDKN1A
FASN
SOD1
NR1I2
PLA2G4A
ABCC2
MTOR
TOP2A
ADRA2A
SLC6A2
SLC6A3
AKR1B1
PLAU
LTA4H
MAOA
ADRB1
F2
F7
IKBKB
TNFSF15
AHSA1
MAPK8
MMP1
STAT1
HMOX1
CYP3A4
CYP1A1
ICAM1
SELE
VCAM1
HAS2
PSMD3
SLC2A4
NR1I3
INSR
DIO1
PPP3CA
GSTM1
SLPI
RXRG
NFKBIA
CASP1
IFNG
ADCYAP1

PSMG1
MAP2K4
NR3C1
F10
PTPN1
CHRM5
OPRD1
CACNA1S
PDE4D
KDR
PYGM
DRD5
HRH1
ADRA2B
DRD2
GABRG3
EGFR
CCND1
BCL2L1
MMP2
MAPK1
IL10
RB1
IL6
TOP1
MDM2
APP
PCNA
ERBB2
CASP7
MCL1
BIRC5
IL2
TYR
IL4
CD40LG
PTGES
MET
GRIA2
MMP3
EIF6
ELK1
ODC1
RAF1
RUNX1T1
CAV1
MYC
F3
GJA1
CXCL8
PRKCB
NOS3
HSPB1
PLAT
THBD
IL1A
NCF1
ABCG2

NFE2L2
NQO1
PARP1
CXCL2
CHEK2
PPARA
PPARD
HSF1
CRP
CXCL10
CHUK
SPP1
RUNX2
E2F1
CTSD
IGFBP3
ERBB3
PCOLCE
NPEPPS
RASA1

Supplementary Table 5

The Results of Molecular Docking Analysis

Target	MOL	Active Compounds	LibDock Score	Hotspots
HSP90AA1	MOL000098	quercetin	87.0016	32.50,12.88,22.57,A,33,2<br>31.50,7.28,27.17,A,68,12<br>29.10,11.07,24.97,P,57,2
HSP90AA1	MOL000006	luteolin	105.736	31.90,7.47,28.37,A,81,11<br>30.10,11.68,22.97,A,37,1<br>5
HSP90AA1	MOL000358	beta-sitosterol	111.251	34.10,10.88,24.37,A,53,6<br>30.10,11.68,22.97,A,37,1<br>6
HSP90AA1	MOL000359	sitosterol	108.553	31.90,6.08,31.37,A,88,3<br>34.10,12.28,23.77,A,47,1<br>9
HSP90AA1	MOL000422	kaempferol	84.5532	32.50,12.88,22.57,A,33,9<br>29.10,11.07,24.97,P,57,11<br>31.50,7.28,27.17,A,68,17
HSP90AA1	MOL000449	Stigmasterol	111.732	30.10,11.68,22.97,A,37,3<br>34.10,10.88,24.37,A,53,7<br>31.30,12.07,19.97,P,29,3
HSP90AA1	MOL000622	Magnograndiolide	86.0255	32.50,12.88,22.57,A,33,8<br>30.30,11.88,20.97,P,37,14<br>30.90,8.68,27.57,A,74,19
HSP90AA1	MOL000787	Fumarine	120.724	30.90,8.68,27.57,A,74,8<br>29.10,11.07,24.97,P,57,17<br>29.10,13.28,20.37,P,33,2
HSP90AA1	MOL001510	24-epicampesterol	112.466	30.10,11.68,22.97,A,37,6<br>34.10,10.88,24.37,A,53,8<br>31.30,12.07,19.97,P,29,1
HSP90AA1	MOL001525	Daucosterol	116.552	34.50,9.47,23.77,A,46,11<br>31.90,9.07,26.37,A,65,23<br>32.70,13.88,23.37,A,43,2
HSP90AA1	MOL001645	Linoleyl acetate	108.399	29.70,10.47,24.37,A,52,4<br>31.90,7.47,28.37,A,81,10<br>36.70,7.08,18.77,P,26,21
HSP90AA1	MOL001771	oriferast-5-en-3beta-	110.523	33.70,7.88,30.77,A,87,16<br>29.30,10.47,25.97,A,62,2<br>7
HSP90AA1	MOL001792	DFV	95.2832	30.10,11.68,22.97,A,37,7<br>32.50,12.88,22.57,A,33,1<br>0
HSP90AA1	MOL002268	Rhein	104.388	30.90,8.68,27.57,A,74,11<br>30.50,12.68,21.37,A,25,1<br>7
HSP90AA1	MOL002714	baicalein	102.138	29.70,10.47,24.37,A,52,1<br>1<br>34.90,7.67,26.77,P,59,14
HSP90AA1	MOL002879	Diop	106.158	34.10,13.07,24.57,A,55,5<br>35.90,6.88,17.97,A,13,17<br>31.50,7.28,27.17,A,68,23
HSP90AA1	MOL002959	3'-Methoxydaidzein	88.2484	30.10,11.68,22.97,A,37,1<br>3<br>32.10,12.88,21.17,A,23,1
HSP90AA1	MOL003044	Chryseriol	94.8724	32.90,10.68,23.57,A,44,9<br>27.90,14.07,21.57,P,41,14<br>30.90,8.68,27.57,A,74,18
HSP90AA1	MOL003542	sopentenyl-kaempfe	110.251	29.10,9.68,27.57,A,75,9<br>31.30,11.07,23.97,A,48,2<br>3

HSP90AA1	MOL003648	Inermin	99.9131	31.10,12.68,22.77,A,35,9<br>34.10,10.88,24.37,A,53,1<br>1
HSP90AA1	MOL004367	olivil	115.446	36.30,7.47,20.37,P,31,6<br>29.70,10.47,24.37,A,52,1<br>9
HSP90AA1	MOL004373	Anhydroicaritin	111.579	29.50,8.47,27.97,P,68,3<br>32.90,10.68,23.57,A,44,1<br>9
HSP90AA1	MOL004380	5-didehydro-3,15,16	96.8498	34.10,13.07,24.57,A,55,9<br>32.50,12.88,22.57,A,33,1<br>2
HSP90AA1	MOL004382	Yinyanghuo A	114.763	35.10,12.07,23.57,A,45,1<br>5<br>34.50,9.47,23.77,A,46,16
HSP90AA1	MOL004384	Yinyanghuo C	111.088	30.50,13.47,20.57,A,18,2<br>32.90,10.68,23.57,A,44,1<br>0
HSP90AA1	MOL004386	Yinyanghuo E	98.7187	31.30,11.07,23.97,A,48,2<br>34.30,7.47,29.37,A,83,12<br>29.10,11.07,24.97,P,57,2
HSP90AA1	MOL004388	dioxo-2,3,4,8-tetrah	98.7759	32.50,11.28,24.97,A,57,1<br>7<br>32.30,7.47,22.57,P,47,20
HSP90AA1	MOL004391	but-2-enyl)-2-pheny	108.492	29.70,10.47,24.37,A,52,2<br>30.50,13.47,20.57,A,18,1<br>5
HSP90AA1	MOL004394	aritin-3-O-alpha-L-r	127.179	34.10,13.07,24.57,A,55,3<br>7<br>30.90,8.68,27.57,A,74,43
HSP90AA1	MOL004396	xy-3-methoxyphenyl	105.835	31.10,12.68,22.77,A,35,3<br>35.10,12.07,23.57,A,45,1<br>0
HSP90AA1	MOL004425	Icariin	96.8833	32.30,7.47,22.57,P,47,11<br>34.30,7.47,29.37,A,83,28<br>33.70,11.68,22.57,A,32,4
HSP90AA1	MOL004427	Icariside A7	98.9879	30.10,10.07,27.17,A,69,1<br>7<br>32.90,7.28,29.97,A,84,18
HSP90AA1	MOL004941	-2-(4-hydroxyphenyl	98.5393	35.50,9.68,22.37,A,30,5<br>32.90,9.68,25.17,A,59,9<br>29.10,12.88,22.17,P,45,1
HSP90AA1	MOL005308	Aposiopolamine	81.2921	29.30,14.28,19.97,A,15,1<br>30.50,12.68,21.37,A,25,3<br>31.90,9.07,26.37,A,65,14
HSP90AA1	MOL005317	Deoxyharringtonine	126.727	32.90,10.68,23.57,A,44,1<br>32.90,7.28,29.97,A,84,15<br>36.70,9.47,20.57,P,35,28
HSP90AA1	MOL005318	Dianthramine	81.9319	29.30,10.47,25.97,A,62,1<br>1<br>31.90,9.07,26.37,A,65,14
HSP90AA1	MOL005320	arachidonate	105.908	34.30,7.47,29.37,A,83,5<br>29.70,10.47,24.37,A,52,7<br>35.30,10.88,23.17,A,40,1
HSP90AA1	MOL005321	Frutinone A	95.0267	30.90,8.68,27.57,A,74,6<br>32.90,10.68,23.57,A,44,1<br>4
HSP90AA1	MOL005348	Ginsenoside-Rh4_qt	118.225	32.30,5.67,30.17,A,85,30<br>32.70,13.88,23.37,A,43,3<br>2
HSP90AA1	MOL005356	Girinimbin	96.8052	29.30,14.28,19.97,A,15,2<br>31.10,10.28,25.57,A,61,1<br>4

HSP90AA1	MOL005376	Panaxadiol	104.473	31.30,8.88,24.57,A,54,18<br>32.90,10.68,23.57,A,44,2<br>1
HSP90AA1	MOL005384	suchilactone	115.368	32.30,5.67,30.17,A,85,8<br>29.50,8.47,27.97,P,68,15<br>32.50,12.28,23.97,A,49,2
HSP90AA1	MOL005399	alexandrin_qt	114.873	31.90,9.07,26.37,A,65,12<br>32.50,12.28,23.97,A,49,1<br>9
HSP90AA1	MOL006331	',5-Dihydroxyflavon	89.037	31.90,7.47,28.37,A,81,2<br>30.10,11.68,22.97,A,37,1<br>3
HSP90AA1	MOL008647	Trans-Feruloyltyram	108.528	29.10,12.88,22.17,P,45,2<br>34.10,10.88,24.37,A,53,6<br>34.50,9.47,23.77,A,46,7
HSP90AA1	MOL009763	garesinol-O-beta-D-	139.073	31.50,7.28,27.17,A,68,23<br>30.50,12.68,21.37,A,25,3<br>7
AKT1	MOL000098	quercetin	73.3052	10.96,-3.11,14.09,P,1,17<br>11.36,0.89,16.29,P,10,18<br>8.36,-2.91,24.49,P,46,21
AKT1	MOL000006	luteolin	93.3177	6.56,-6.71,19.69,P,21,10<br>13.56,-3.31,17.09,P,12,20<br>12.16,-1.31,15.89,P,7,21
AKT1	MOL000358	beta-sitosterol	100.431	3.96,1.89,15.89,A,7,17<br>1.16,3.09,16.29,A,12,21<br>11.56,-
AKT1	MOL000359	sitosterol	110.739	6.76,4.89,16.69,A,17,11<br>4.16,3.69,15.89,A,8,14<br>-0.64,3.49,16.89,A,22,19
AKT1	MOL000422	kaempferol	76.4154	8.76,-9.11,18.09,A,27,14<br>10.56,-8.11,16.89,A,20,16<br>8.36,-2.91,24.49,P,46,19
AKT1	MOL000449	Stigmasterol	102.405	10.36,-8.51,18.49,A,28,4<br>7.96,5.29,17.89,A,26,20<br>11.56,-
AKT1	MOL000622	Magnograndiolide	80.2729	9.16,-7.91,16.69,A,15,6<br>9.36,-8.91,21.09,A,34,9<br>11.56,-
AKT1	MOL001510	24-epicampesterol	107.692	10.36,-8.51,18.49,A,28,3<br>9.36,6.89,19.09,A,29,26<br>7.96,5.29,17.89,A,26,29
AKT1	MOL001525	Daucosterol	70.3039	10.96,8.09,20.89,A,33,24<br>10.36,9.09,23.69,A,39,29<br>7.56,8.89,20.49,A,32,30
AKT1	MOL001645	Linoleyl acetate	121.946	4.96,-6.31,24.09,A,40,2<br>5.56,-4.91,24.09,A,41,3<br>10.56,-
AKT1	MOL001771	poriferast-5-en-3beta-ol	111.7	10.56,-8.11,16.89,A,20,26<br>9.36,-8.91,21.09,A,34,29<br>8.76,-9.11,18.09,A,27,30
AKT1	MOL001792	DFV	83.8764	8.76,-9.11,18.09,A,27,14<br>6.56,-9.51,17.89,P,17,18<br>10.96,-3.11,14.09,P,1,19
AKT1	MOL002268	Rhein	74.2905	11.96,1.29,31.29,A,52,12<br>9.36,9.49,31.49,A,54,17<br>9.16,4.49,31.09,P,65,20
AKT1	MOL002714	baicalein	82.5208	8.36,-2.91,24.49,P,46,14<br>10.56,-8.11,16.89,A,20,18<br>8.76,-9.11,18.09,A,27,19
AKT1	MOL002879	Diop	117.546	10.56,-8.11,16.89,A,20,4<br>6.36,-6.11,23.29,A,37,16<br>3.76,-7.11,24.69,A,43,19

AKT1	MOL002959	3'-Methoxydaidzein	96.398	10.36,-8.51,18.49,A,28,5 10.76,-2.11,15.69,A,4,16 8.36,-9.91,21.29,P,29,21
AKT1	MOL003044	Chryseriol	102.011	8.36,-2.91,24.49,P,46,14 11.56,-3.91,16.69,A,16,16 10.36,-
AKT1	MOL003542	8-Isopentenyl-kaempferol	112.732	11.56,-0.71,16.29,A,11,20 7.96,-8.91,16.69,A,14,25 10.56,-
AKT1	MOL003648	Inermin	83.9107	6.56,-6.71,19.69,P,21,14 8.76,-9.11,18.09,A,27,20 5.96,-9.31,15.69,P,6,21
AKT1	MOL004367	olivil	114.89	6.76,1.89,21.09,P,28,3 6.76,4.89,16.69,A,17,24 4.96,0.49,16.49,A,13,27
AKT1	MOL004373	Anhydroicaritin	107.294	9.36,6.89,19.09,A,29,17 11.56,-0.71,16.29,A,11,21 6.76,4.89,16.69,A,17,22
AKT1	MOL004380	C-Homoerythrinan, 1,6-didehydro-	74.0408	9.36,6.89,19.09,A,29,20 6.76,4.89,16.69,A,17,22 12.16,0.09,15.49,P,5,23
AKT1	MOL004382	Yinyanghuo A	126.649	11.36,0.89,16.29,P,10,3 10.76,-2.11,15.69,A,4,26 9.36,-8.91,21.09,A,34,31
AKT1	MOL004384	Yinyanghuo C	89.0822	9.36,-8.91,21.09,A,34,12 7.96,-10.11,18.09,P,18,17 9.16,-7.91,16.69,A,15,18
AKT1	MOL004386	Yinyanghuo E	90.6348	10.36,-8.51,18.49,A,28,3 6.56,-9.51,17.89,P,17,20 8.36,-2.91,24.49,P,46,22
AKT1	MOL004388	6-hydroxy-11,12-dimethoxy-2,2-dimethyl-1,8-dioxo-2,3,4,8-tetrahydro-1H-isochromeno[3,4-	104.543	7.96,-8.91,16.69,A,14,9 9.16,-7.91,16.69,A,15,10 4.96,-6.31,24.09,A,40,26
AKT1	MOL004391	8-(3-methylbut-2-enyl)-2-phenyl-chromone	114.977	10.36,-8.51,18.49,A,28,6 11.56,-0.71,16.29,A,11,14 6.36,-6.11,23.29,A,37,21
AKT1	MOL004394	Anhydroicaritin-3-O-alpha-L-rhamnoside	111.26	10.76,-2.11,15.69,A,4,37 10.36,-8.51,18.49,A,28,44
AKT1	MOL004396	1,2-bis(4-hydroxy-3-methoxyphenyl)pr	116.422	9.36,-8.91,21.09,A,34,3 6.56,-6.71,19.69,P,21,4 8.76,-9.11,18.09,A,27,20
AKT1	MOL004427	Icariside A7	71.7345	9.36,-8.91,21.09,A,34,29 8.36,-9.91,21.29,P,29,31 6.56,-6.71,19.69,P,21,32
AKT1	MOL004941	(2R)-7-hydroxy-2-(4-hydroxyphenyl)chr	89.8111	9.36,-8.91,21.09,A,34,8 9.16,-7.91,16.69,A,15,13 7.16,-3.51,23.29,P,39,19
AKT1	MOL005308	Aposiopolamine	93.9236	9.76,-9.51,16.89,A,19,12 9.36,-10.71,17.69,P,15,18 6.56,-6.71,19.69,P,21,19
AKT1	MOL005317	Deoxyharringtonine	128.694	6.36,-6.11,23.29,A,37,5 8.76,-9.11,18.09,A,27,27 11.56,-
AKT1	MOL005318	Dianthramine	75.3423	8.76,-9.11,18.09,A,27,3 6.56,-6.71,19.69,P,21,7 13.56,-3.31,17.09,P,12,21
AKT1	MOL005320	arachidonate	125.156	9.56,8.69,20.29,A,31,5 10.76,-2.11,15.69,A,4,15 10.56,-

AKT1	MOL005321	Frutinone A	90.1252	5.16,5.09,15.89,A,10,6<br>4.16,-1.11,17.09,A,23,15<br>1.56,-2.11,17.49,A,25,18
AKT1	MOL005384	suchilactone	115.72	11.56,-3.91,16.69,A,16,8<br>10.56,-8.11,16.89,A,20,16<br>5.56,-4.91,24.09,A,41,27
AKT1	MOL005399	alexandrin_qt	92.1736	10.76,-2.11,15.69,A,4,14<br>8.76,-9.11,18.09,A,27,28<br>9.36,-8.91,21.09,A,34,30
AKT1	MOL006331	4',5-<br>Dihydroxyflavone	90.1262	10.36,-<br>8.51,18.49,A,28,16<br>9.36,-10.71,17.69,P,15,17
AKT1	MOL008647	N-Trans-<br>Feruloyltyramine	119.141	4.76,-6.71,23.69,P,41,2<br>9.36,-8.91,21.09,A,34,6<br>10.76,-2.11,15.69,A,4,22
AKT1	MOL009763	(+)-Syringaresinol-<br>O-beta-D-<br>glucoside	132.741	6.56,-6.71,19.69,P,21,10<br>9.76,-9.51,16.89,A,19,37<br>9.36,6.89,19.09,A,29,41
MAPK1	MOL000098	quercetin	87.7245	-5.80,11.92,36.74,A,41,13<br>-7.40,12.32,34.94,P,17,19<br>-2.80,12.72,38.94,P,60,20
MAPK1	MOL000006	luteolin	91.7385	-4.40,12.32,36.54,P,39,10<br>-9.60,15.32,38.94,P,63,14<br>-
MAPK1	MOL000358	beta-sitosterol	85.1202	-2.40,13.12,39.74,A,80,4<br>-5.80,11.92,36.74,A,41,18<br>-
MAPK1	MOL000359	sitosterol	75.0828	-1.60,14.12,41.54,A,92,11<br>-<br>2.60,15.92,38.34,A,66,23
MAPK1	MOL000422	kaempferol	81.7641	-5.80,11.92,36.74,A,41,16<br>-7.40,12.32,34.94,P,17,18<br>-2.80,12.72,38.94,P,60,21<br>-
MAPK1	MOL000449	Stigmasterol	92.7545	7.40,14.32,35.74,A,29,18<br>-7.00,10.52,32.94,A,1,20
MAPK1	MOL000622	Magnograndiolide	66.3205	-8.00,15.32,37.34,A,53,9<br>-<br>5.60,12.12,35.34,A,18,16
MAPK1	MOL000787	Fumarine	91.3679	-2.40,14.12,40.14,A,88,7<br>-<br>3.20,14.12,36.14,A,33,18
MAPK1	MOL001510	24-epicampesterol	94.1591	-7.00,13.12,35.54,A,22,3<br>-4.40,12.32,36.74,A,42,6<br>-
MAPK1	MOL001645	Linoleyl acetate	118.306	-9.20,14.72,37.74,P,46,11<br>-<br>2.40,14.12,40.14,A,88,13
MAPK1	MOL001771	poriferast-5-en-<br>3beta-ol	92.8462	-6.00,10.92,34.74,A,11,1<br>-7.80,13.52,37.34,A,51,7<br>-
MAPK1	MOL001792	DFV	105.191	-5.00,12.72,38.34,P,52,6<br>-<br>7.40,14.32,35.74,A,29,10
MAPK1	MOL002268	Rhein	83.9238	-6.00,13.32,36.94,A,46,8<br>-7.40,12.32,34.94,P,17,18<br>-9.20,15.72,39.94,P,76,20
MAPK1	MOL002268	Rhein	78.9808	-4.60,13.72,38.54,A,69,8<br>-4.40,12.32,36.54,P,39,18<br>-9.60,15.32,38.94,P,63,20
MAPK1	MOL002714	baicalein	86.2509	-4.40,12.32,36.54,P,39,12<br>-9.60,15.32,38.94,P,63,14<br>-

MAPK1	MOL002879	Diop	81.6015	-<br>3.00,13.52,38.94,A,73,21<br>-
MAPK1	MOL002959	3'-<br>Methoxydaidzein	97.0345	-<br>3.00,13.52,38.94,A,73,17<br>-
MAPK1	MOL003044	Chryseriol	96.6791	-2.40,14.12,40.14,A,88,9<br>-<br>4.40,12.32,36.74,A,42,18
MAPK1	MOL003542	8-Isopentenyl-<br>kaempferol	101.745	-2.80,12.72,38.94,P,60,3<br>-7.40,12.32,34.94,P,17,19<br>-
MAPK1	MOL003648	Inermin	93.7842	-7.20,13.92,38.54,P,56,14<br>-<br>7.20,12.52,36.74,A,43,17
MAPK1	MOL004367	olivil	114.812	-<br>5.00,13.92,37.34,A,52,21<br>-
MAPK1	MOL004373	Anhydroicaritin	108.018	-2.80,12.72,38.94,P,60,1<br>-7.40,12.32,34.94,P,17,6<br>-
MAPK1	MOL004380	C-<br>Homoerythrinan,<br>1,6-didehydro-	79.6425	-<br>1.80,13.32,40.74,A,90,12<br>-
MAPK1	MOL004382	Yinyanghuo A	101.006	-8.60,13.92,38.54,P,55,4<br>-6.20,10.72,33.34,P,2,6<br>-
MAPK1	MOL004384	Yinyanghuo C	98.3525	-5.60,12.12,35.34,A,18,3<br>-<br>3.00,14.92,38.74,A,71,12
MAPK1	MOL004386	Yinyanghuo E	69.6435	-<br>3.00,13.52,38.94,A,73,14<br>-7.20,13.92,38.54,P,56,22
MAPK1	MOL004388	6-hydroxy-11,12-<br>dimethoxy-2,2-<br>dimethyl-1,8-<br>dioxo-2,3,4,8-<br>tetrahydro-1H-<br>isochromeno[3,4-	69.0006	-2.60,15.52,37.34,A,54,2<br>-<br>3.00,13.52,38.94,A,73,18<br>-2.40,12.92,42.54,P,93,25
MAPK1	MOL004391	8-(3-methylbut-2-<br>enyl)-2-phenyl-<br>chromone	99.8411	-4.40,12.32,36.74,A,42,6<br>-<br>7.60,14.32,38.34,A,65,18
MAPK1	MOL004396	1,2-bis(4-hydroxy-<br>3-<br>methoxyphenyl)pr	117.168	-5.80,11.92,36.74,A,41,3<br>-<br>9.20,16.72,35.54,A,25,13
MAPK1	MOL004941	(2R)-7-hydroxy-2-<br>(4-<br>hydroxyphenyl)chr	104.549	-6.00,12.32,38.34,A,63,9<br>-<br>8.80,14.92,38.14,A,62,13
MAPK1	MOL005308	Aposiopolamine	81.6376	-2.60,18.12,36.54,A,39,5<br>-3.40,12.72,37.74,A,58,11<br>-
MAPK1	MOL005317	Deoxyharringtonin<br>e	87.1261	-7.20,13.92,38.54,P,56,26<br>-<br>7.80,13.52,37.34,A,51,27
MAPK1	MOL005318	Dianthramine	88.8599	-4.60,13.72,38.54,A,69,6<br>-2.00,11.52,41.74,P,87,15<br>-2.20,15.72,34.94,P,19,19
MAPK1	MOL005320	arachidonate	118.296	-<br>2.60,15.92,38.34,A,66,12<br>-
MAPK1	MOL005321	Frutinone A	90.404	-2.40,13.12,39.74,A,80,3<br>-<br>7.60,14.32,38.34,A,65,14

MAPK1	MOL005356	Girinimbin	89.4855	-7.00,13.12,35.54,A,22,3<br>-8.00,15.32,37.34,A,53,6<br>-
MAPK1	MOL005384	suchilactone	103.385	-7.60,14.32,36.74,A,44,7<br>-4.80,12.12,38.14,A,61,11<br>-3.00,14.32,35.94,P,30,15
MAPK1	MOL005399	alexandrin_qt	88.591	-<br>1.80,16.52,36.34,A,38,23<br>-7.00,11.72,35.54,A,21,28
MAPK1	MOL006331	4',5-<br>Dihydroxyflavone	86.9838	-7.80,13.52,37.34,A,51,1<br>-3.00,13.52,38.94,A,73,8<br>-
MAPK1	MOL008647	N-Trans-<br>Feruloyltyramine	108.091	-0.80,13.52,40.14,P,79,3<br>-7.40,12.32,34.94,P,17,4<br>-
JUN	MOL000098	quercetin	92.5499	13.17,0.76,14.86,A,91,6<br>6.57,1.56,12.06,A,73,13<br>9.97,-0.04,10.66,P,65,20
JUN	MOL000006	luteolin	95.8399	10.97,5.36,10.86,A,70,11<br>12.17,4.76,8.86,P,52,14<br>10.17,-3.84,11.06,P,68,20
JUN	MOL000358	beta-sitosterol	94.6901	6.17,-0.04,15.86,A,93,4<br>2.97,1.36,13.06,A,84,12<br>13.17,0.76,14.86,A,91,30
JUN	MOL000359	sitosterol	107.771	12.57,4.76,9.06,A,61,19<br>2.97,1.36,13.06,A,84,27<br>12.57,5.76,12.06,A,74,28
JUN	MOL000422	kaempferol	88.2745	13.17,0.76,14.86,A,91,7<br>6.57,1.56,12.06,A,73,16<br>9.97,-0.04,10.66,P,65,21
JUN	MOL000449	Stigmasterol	39.1558	12.17,7.36,16.06,A,95,15<br>11.37,6.56,12.26,A,77,20<br>13.17,8.36,19.06,A,100,2
JUN	MOL001510	24-epicampesterol	119.337	2.97,1.36,13.06,A,84,14<br>6.17,-0.04,15.86,A,93,15<br>10.97,5.36,10.86,A,70,26
JUN	MOL001645	Linoleyl acetate	135.724	8.17,-9.64,0.86,A,6,1<br>6.17,-14.44,4.66,A,33,9<br>2.77,-14.04,6.46,A,43,14
JUN	MOL001771	poriferast-5-en-<br>3beta-ol	85.025	12.17,7.36,16.06,A,95,23<br>13.17,8.36,19.06,A,100,2<br>7
JUN	MOL001792	DFV	106.59	8.77,-5.64,12.26,A,76,9<br>14.77,-<br>0.04,14.86,A,90,14
JUN	MOL002268	Rhein	123.495	11.37,6.56,12.26,A,77,11<br>12.77,5.56,10.46,A,65,13<br>7.97,0.96,10.66,P,66,15
JUN	MOL002714	baicalein	90.0553	9.97,-0.04,10.66,P,65,10<br>11.57,6.16,14.06,A,88,16<br>12.17,7.36,16.06,A,95,20
JUN	MOL002879	Diop	132.918	11.97,6.76,10.66,A,68,4<br>13.17,8.36,19.06,A,100,1<br>6
JUN	MOL002959	3'-<br>Methoxydaidzein	100.269	4.37,-16.04,3.86,A,26,6<br>6.17,-14.44,4.66,A,33,8<br>11.37,-
JUN	MOL003044	Chryseriol	90.3244	7.57,5.56,13.86,P,87,14<br>13.17,0.76,14.86,A,91,15<br>14.17,-
JUN	MOL003542	8-Isopentenyl-<br>kaempferol	105.751	7.57,5.56,13.86,P,87,11<br>13.17,0.76,14.86,A,91,16<br>14.17,-

JUN	MOL003648	Inermin	81.1743	4.37,-13.84,5.86,A,39,4<br>3.57,-14.04,4.66,P,25,5<br>1.17,-13.44,7.26,A,52,9
JUN	MOL004367	olivil	121.577	11.57,7.96,10.46,P,63,7<br>6.17,-0.04,15.86,A,93,26<br>11.57,6.16,14.06,A,88,27
JUN	MOL004373	Anhydroicaritin	100.178	14.77,-<br>0.04,14.86,A,90,21<br>11.57,6.16,14.06,A,88,22
JUN	MOL004380	C-<br>Homoerythrinan,<br>1,6-didehydro-	100.937	11.57,6.16,14.06,A,88,18<br>14.17,-<br>1.64,13.46,A,86,20
JUN	MOL004382	Yinyanghuo A	128.981	13.37,4.36,8.26,P,50,4<br>9.77,-4.44,12.66,A,81,19<br>11.37,6.56,12.26,A,77,29
JUN	MOL004388	6-hydroxy-11,12-<br>dimethoxy-2,2-<br>dimethyl-1,8-<br>dioxo-2,3,4,8-<br>tetrahydro-1H-<br>isochromeno[3,4-	104.709	12.57,5.76,12.06,A,74,18<br>12.17,7.36,16.06,A,95,26<br>9.97,-0.04,10.66,P,65,27
JUN	MOL004391	8-(3-methylbut-2-<br>enyl)-2-phenyl-<br>chromone	107.802	11.37,6.56,12.26,A,77,14<br>12.77,5.56,10.46,A,65,16<br>14.17,-
JUN	MOL004394	Anhydroicaritin-3-<br>O-alpha-L-<br>rhamnoside	110.697	17.37,-9.84,7.26,A,53,32<br>15.37,-<br>10.44,10.26,A,64,37
JUN	MOL004396	1,2-bis(4-hydroxy-<br>3-<br>methoxyphenyl)pr	109.209	13.17,0.76,14.86,A,91,7<br>14.17,-<br>1.64,13.46,A,86,10
JUN	MOL004425	Icariin	127.031	2.97,10.96,6.66,P,36,13<br>4.17,6.76,5.26,P,28,36<br>6.17,5.96,3.46,P,12,43
JUN	MOL004427	Icariside A7	35.9047	5.57,11.76,11.86,A,72,26<br>5.37,10.96,9.66,P,61,30<br>6.77,15.36,11.06,P,69,31
JUN	MOL004941	(2R)-7-hydroxy-2-<br>(4-<br>hydroxyphenyl)chr	79.0571	10.97,5.36,10.86,A,70,15<br>7.57,5.56,13.86,P,87,17<br>12.17,4.76,8.86,P,52,18
JUN	MOL005308	Aposiopolamine	98.6936	6.17,-0.04,15.86,A,93,1<br>11.57,6.16,14.06,A,88,12<br>12.57,5.76,12.06,A,74,15
JUN	MOL005317	Deoxyharringtonin<br>e	43.7426	16.17,-5.84,5.26,P,27,13<br>17.17,-4.24,6.26,P,30,14<br>17.37,-10.04,6.66,P,33,21
JUN	MOL005318	Dianthramine	90.0999	9.37,-13.24,4.26,P,20,7<br>11.37,-17.84,3.46,A,21,14<br>5.97,-17.64,2.86,A,19,16
JUN	MOL005320	arachidonate	137.942	2.77,-14.04,6.46,A,43,9<br>5.97,-14.04,3.26,A,20,13<br>6.37,-7.04,0.46,A,2,20
JUN	MOL005321	Frutinone A	99.1078	7.57,-5.04,11.66,A,71,1<br>8.57,-5.24,14.06,A,87,3<br>10.77,-2.24,10.66,P,64,19
JUN	MOL005356	Girinimbin	102.82	12.77,5.56,10.46,A,65,1<br>11.37,6.56,12.26,A,77,3<br>6.17,-0.04,15.86,A,93,19
JUN	MOL005384	suchilactone	130.681	1.77,-1.24,9.26,A,63,1<br>2.97,1.36,13.06,A,84,8<br>12.77,5.56,10.46,A,65,27
JUN	MOL005399	alexandrin_qt	103.269	6.57,1.56,12.06,A,73,18<br>14.17,-<br>1.64,13.46,A,86,23

JUN	MOL006331	4',5-Dihydroxyflavone	79.4552	8.77,-5.64,12.26,A,76,2<br>15.77,-1.24,12.86,A,82,14
JUN	MOL008647	N-Trans-Feruloyltyramine	136.499	6.17,-14.44,4.66,A,33,7<br>9.77,-11.84,2.06,A,17,11<br>2.77,-14.04,6.46,A,43,17
JUN	MOL009763	(+)-Syringaresinol-O-beta-D-glucoside	90.9099	13.17,8.16,17.26,A,97,19<br>11.57,6.16,14.06,A,88,25<br>11.97,6.76,10.66,A,68,40
RELA	MOL000098	quercetin	64.4319	22.84,22.69,4.16,A,32,2<br>20.64,23.29,3.36,A,18,6<br>23.84,20.69,4.16,P,19,18
RELA	MOL000006	luteolin	69.377	20.64,23.29,3.36,A,18,4<br>22.84,22.69,4.16,A,32,11<br>23.84,20.69,4.16,P,19,12
RELA	MOL000358	beta-sitosterol	82.5069	20.44,23.49,2.36,A,16,13<br>22.84,20.49,3.76,A,23,21<br>23.84,21.89,5.36,P,30,25
RELA	MOL000359	sitosterol	75.2792	24.84,20.29,3.56,A,20,2<br>23.64,22.29,4.36,A,38,4<br>21.44,22.69,3.56,A,21,6
RELA	MOL000422	kaempferol	67.963	22.44,21.89,3.76,A,24,13<br>23.64,22.29,4.36,A,38,14<br>24.84,20.29,3.56,A,20,16
RELA	MOL000449	Stigmasterol	77.6599	22.84,20.49,3.76,A,23,2<br>20.44,23.49,2.36,A,16,6<br>23.84,21.89,5.36,P,30,30
RELA	MOL000787	Fumarine	69.0548	20.64,23.29,3.36,A,18,18<br>22.84,22.69,4.16,A,32,21<br>25.04,21.29,4.16,P,20,25
RELA	MOL001510	24-epicampesterol	81.4243	22.84,20.49,3.76,A,23,1<br>20.44,23.49,2.36,A,16,7<br>23.84,21.89,5.36,P,30,18
RELA	MOL001525	Daucosterol	73.9129	23.84,21.89,5.36,P,30,1<br>20.44,23.49,2.36,A,16,17<br>22.84,20.49,3.76,A,23,20
RELA	MOL001645	Linoleyl acetate	102.431	20.64,23.29,3.36,A,18,5<br>22.84,20.49,3.76,A,23,9<br>23.44,16.49,4.16,A,30,17
RELA	MOL001771	poriferast-5-en-3beta-ol	84.2977	22.84,20.49,3.76,A,23,1<br>20.44,23.49,2.36,A,16,7<br>23.84,21.89,5.36,P,30,15
RELA	MOL001792	DFV	64.9361	22.84,22.69,4.16,A,32,14<br>20.64,23.89,3.96,A,28,16<br>22.44,24.89,5.16,P,29,18
RELA	MOL002268	Rhein	66.6413	20.64,23.29,3.36,A,18,11<br>22.84,22.69,4.16,A,32,13<br>23.84,20.69,4.16,P,19,21
RELA	MOL002714	baicalein	48.8625	22.44,21.89,3.76,A,24,16<br>23.64,22.29,4.36,A,38,18<br>24.84,20.29,3.56,A,20,19
RELA	MOL002879	Diop	95.3997	24.04,16.49,6.36,A,51,4<br>22.84,22.69,4.16,A,32,22<br>21.44,22.69,3.56,A,21,23
RELA	MOL002959	3'-Methoxydaidzein	70.9079	20.44,23.49,2.36,A,16,17<br>23.84,20.29,3.36,A,17,19<br>23.24,23.29,4.76,P,23,20
RELA	MOL003044	Chryseriol	73.1797	22.84,20.49,3.76,A,23,18<br>24.44,20.49,5.56,P,32,21<br>24.04,16.49,6.36,A,51,22
RELA	MOL003542	8-Isopentenyl-kaempferol	87.7906	22.44,21.89,3.76,A,24,9<br>24.04,17.89,4.36,A,34,23<br>24.04,16.49,6.36,A,51,25

RELA	MOL003648	Inermin	64.7208	20.64,23.89,3.96,A,28,17<br>22.84,22.69,4.16,A,32,19<br>22.44,24.89,5.16,P,29,21
RELA	MOL004367	olivil	96.8114	22.04,16.89,6.76,P,38,6<br>17.84,23.89,4.56,P,22,7<br>21.44,22.69,3.56,A,21,27
RELA	MOL004373	Anhydroicaritin	99.1972	22.44,21.89,3.76,A,24,17<br>24.04,17.89,4.36,A,34,22<br>25.24,15.69,3.96,A,25,25
RELA	MOL004380	C-Homoerythrinan, 1,6-didehydro-	73.1193	20.64,23.29,3.36,A,18,7<br>19.44,23.49,4.36,A,39,8<br>24.84,20.29,3.56,A,20,20
RELA	MOL004382	Yinyanghuo A	100.109	21.44,22.69,3.56,A,21,19<br>23.44,18.69,5.56,A,47,26<br>23.44,16.49,4.16,A,30,27
RELA	MOL004384	Yinyanghuo C	79.3662	22.84,22.69,4.16,A,32,2<br>20.64,23.29,3.36,A,18,3<br>23.84,20.69,4.16,P,19,20
RELA	MOL004386	Yinyanghuo E	79.4097	22.84,22.69,4.16,A,32,2<br>20.64,23.29,3.36,A,18,3<br>23.84,20.69,4.16,P,19,20
RELA	MOL004388	6-hydroxy-11,12-dimethoxy-2,2-dimethyl-1,8-dioxo-2,3,4,8-tetrahydro-1H-isochromeno[3,4-	69.0877	23.24,17.89,4.76,A,41,2<br>20.44,23.49,2.36,A,16,10<br>23.84,21.89,5.36,P,30,27
RELA	MOL004391	8-(3-methylbut-2-enyl)-2-phenyl-chromone	87.8271	23.84,20.69,4.16,P,19,4<br>23.24,17.89,4.76,A,41,6<br>20.44,23.49,2.36,A,16,13
RELA	MOL004394	Anhydroicaritin-3-O-alpha-L-rhamnoside	99.9231	20.44,23.49,2.36,A,16,7<br>23.84,21.89,5.36,P,30,9<br>24.04,16.49,6.36,A,51,26
RELA	MOL004396	1,2-bis(4-hydroxy-3-methoxyphenyl)pr	99.6134	23.84,20.29,3.36,A,17,13<br>24.44,15.29,3.96,P,18,21<br>23.84,16.69,6.36,P,37,23
RELA	MOL004425	Icariin	76.6092	19.44,23.49,4.36,A,39,25<br>20.84,24.49,-1.24,A,8,39<br>20.44,16.09,8.96,P,48,41
RELA	MOL004427	Icariside A7	59.9056	20.64,23.29,3.36,A,18,9<br>22.84,22.69,4.16,A,32,14<br>23.24,17.89,4.76,A,41,21
RELA	MOL004941	(2R)-7-hydroxy-2-(4-hydroxyphenyl)chr	59.3846	22.84,22.69,4.16,A,32,13<br>20.64,23.89,3.96,A,28,15<br>22.44,24.89,5.16,P,29,18
RELA	MOL005308	Aposiopolamine	68.8183	25.04,7.49,15.76,A,92,1<br>22.64,7.29,15.36,A,89,3<br>20.64,9.09,14.76,P,86,19
RELA	MOL005317	Deoxyharringtonine	93.3052	25.44,16.69,4.16,A,31,1<br>24.04,16.49,6.36,A,51,5<br>20.64,23.29,3.36,A,18,27
RELA	MOL005318	Dianthramine	74.6061	22.84,22.69,4.16,A,32,11<br>19.24,23.89,4.16,P,21,15<br>25.04,21.29,4.16,P,20,21
RELA	MOL005320	arachidonate	108.656	20.64,23.29,3.36,A,18,14<br>23.24,19.29,3.96,A,27,18<br>23.84,16.69,6.36,P,37,22
RELA	MOL005321	Frutinone A	57.5379	21.44,16.69,7.96,P,47,13<br>23.24,17.89,4.76,A,41,15<br>24.84,17.49,6.36,A,52,17
RELA	MOL005348	Ginsenoside-Rh4_qt	60.6336	22.44,21.89,3.76,A,24,19<br>24.04,17.89,4.36,A,34,28<br>20.64,23.29,3.36,A,18,30

RELA	MOL005356	Girinimbin	58.7796	20.64,16.09,7.76,P,45,7<br>24.84,17.49,6.36,A,52,15<br>25.44,16.69,4.16,A,31,20
RELA	MOL005384	suchilactone	92.9714	20.64,23.29,3.36,A,18,11<br>22.84,20.49,3.76,A,23,14<br>22.84,22.69,4.16,A,32,16
RELA	MOL005399	alexandrin_qt	78.4925	20.64,23.89,3.96,A,28,23<br>24.84,20.29,3.56,A,20,27<br>23.24,17.89,4.76,A,41,28
RELA	MOL006331	4',5-Dihydroxyflavone	65.0419	22.84,22.69,4.16,A,32,2<br>20.64,23.29,3.36,A,18,6<br>23.84,20.69,4.16,P,19,18
RELA	MOL008647	N-Trans-Feruloyltyramine	93.4074	22.44,24.89,5.16,P,29,2<br>20.64,23.89,3.96,A,28,11<br>22.84,22.69,4.16,A,32,12
RELA	MOL009763	(+)-Syringaresinol-O-beta-D-glucoside	114.972	22.04,16.89,6.76,P,38,9<br>24.84,16.29,4.96,P,25,10<br>22.84,20.49,3.76,A,23,37
MAPK14	MOL000098	quercetin	85.7335	5.73,10.76,-23.25,A,22,2<br>2.53,12.56,-17.45,A,59,12
MAPK14	MOL000006	luteolin	83.2868	2.33,13.76,-23.45,P,20,10<br>5.73,10.76,-23.25,A,22,11<br>2.53,12.56,-
MAPK14	MOL000358	beta-sitosterol	103.186	2.53,12.56,-17.45,A,59,6<br>2.13,11.16,-21.05,A,40,17<br>4.73,11.76,-
MAPK14	MOL000359	sitosterol	88.4406	2.53,12.56,-17.45,A,59,10<br>5.93,8.56,-20.85,A,43,18
MAPK14	MOL000422	kaempferol	87.842	5.73,10.76,-23.25,A,22,9<br>2.33,13.76,-23.45,P,20,11<br>2.53,12.56,-
MAPK14	MOL000449	Stigmasterol	102.413	4.73,11.76,-22.25,A,32,2<br>2.53,12.56,-17.45,A,59,7<br>2.13,11.16,-
MAPK14	MOL000622	Magnograndiolide	79.3968	18.13,5.96,-20.85,A,41,6<br>17.93,4.36,-26.65,P,6,18<br>18.13,2.76,-
MAPK14	MOL000787	Fumarine	75.2489	4.93,11.96,-18.05,A,54,14<br>2.53,12.56,-17.45,A,59,15
MAPK14	MOL001510	24-epicampesterol	88.3721	2.53,12.56,-17.45,A,59,20<br>4.73,11.76,-22.25,A,32,21
MAPK14	MOL001525	Daucosterol	98.5296	2.53,12.56,-17.45,A,59,12<br>2.13,11.16,-21.05,A,40,18
MAPK14	MOL001645	Linoleyl acetate	119.201	8.93,13.96,-23.05,A,27,1<br>4.93,11.96,-18.05,A,54,7<br>8.53,7.16,-16.65,A,65,17
MAPK14	MOL001771	poriferast-5-en-3beta-ol	97.3555	4.73,11.76,-22.25,A,32,1<br>2.53,12.56,-17.45,A,59,8<br>2.13,11.16,-
MAPK14	MOL001792	DFV	94.8437	4.73,11.76,-22.25,A,32,1<br>5.93,9.56,-19.45,A,45,7<br>4.93,11.96,-
MAPK14	MOL002714	baicalein	90.3524	4.73,11.76,-22.25,A,32,4<br>6.13,7.76,-19.25,A,46,17<br>7.13,7.16,-17.05,A,61,20
MAPK14	MOL002879	Diop	106.394	5.73,10.76,-23.25,A,22,4<br>6.13,7.76,-16.45,A,67,22<br>9.53,5.96,-16.65,A,64,25

MAPK14	MOL002959	3'-Methoxydaidzein	67.9102	2.53,12.56,-17.45,A,59,5 5.73,10.76,-23.25,A,22,16
MAPK14	MOL003044	Chryseriol	83.9557	2.33,13.76,-23.45,P,20,10 5.73,10.76,-23.25,A,22,11 2.53,12.56,-
MAPK14	MOL003542	8-Isopentenyl-kaempferol	98.3008	9.73,12.96,-22.65,P,28,19 4.93,11.96,-18.05,A,54,25 2.53,12.56,-
MAPK14	MOL003648	Inermin	87.7178	4.73,11.76,-22.25,A,32,13 5.93,9.56,-19.45,A,45,17 4.93,11.96,-
MAPK14	MOL004367	olivil	111.482	16.13,6.16,-25.45,P,9,3 18.13,7.76,-21.25,A,38,11 14.93,1.76,-
MAPK14	MOL004373	Anhydroicaritin	91.0006	9.33,5.96,-14.85,A,76,21 7.13,12.16,-15.05,A,74,26
MAPK14	MOL004380	C-Homoerythrinan, 1,6-didehydro-	93.5422	4.73,11.76,-22.25,A,32,13 8.93,13.96,-23.05,A,27,22
MAPK14	MOL004382	Yinyanghuo A	120.332	16.93,3.96,-24.25,A,13,11 18.13,7.76,-21.25,A,38,15
MAPK14	MOL004384	Yinyanghuo C	86.1155	2.53,12.56,-17.45,A,59,14 4.93,11.96,-18.05,A,54,18
MAPK14	MOL004386	Yinyanghuo E	89.1774	2.53,12.56,-17.45,A,59,14 4.93,11.96,-18.05,A,54,18
MAPK14	MOL004388	6-hydroxy-11,12-dimethoxy-2,2-dimethyl-1,8-dioxo-2,3,4,8-tetrahydro-1H-isochromeno[3,4-	27.8841	2.13,11.16,-21.05,A,40,9 2.33,13.76,-23.45,P,20,20 -0.07,13.76,-26.05,A,6,26
MAPK14	MOL004391	8-(3-methylbut-2-enyl)-2-phenyl-chromone	88.061	2.33,13.76,-23.45,P,20,4 5.73,10.76,-23.25,A,22,7 2.53,12.56,-
MAPK14	MOL004396	1,2-bis(4-hydroxy-3-methoxyphenyl)pr	110.738	18.13,8.16,-20.85,P,35,5 18.13,3.76,-25.85,A,8,19 14.93,1.76,-
MAPK14	MOL004427	Icariside A7	63.3909	2.53,12.56,-17.45,A,59,17 5.93,10.56,-
MAPK14	MOL004941	(2R)-7-hydroxy-2-(4-hydroxyphenyl)chr	89.9586	18.13,1.16,-24.65,A,11,2 16.13,2.56,-24.05,A,14,5 18.13,7.76,-
MAPK14	MOL005308	Aposiopolamine	67.0051	6.53,11.96,-22.65,A,29,13 5.73,10.76,-23.25,A,22,16
MAPK14	MOL005317	Deoxyharringtonine	97.0167	-0.07,13.76,-26.05,A,6,5 2.53,12.56,-17.45,A,59,30
MAPK14	MOL005318	Dianthramine	71.8003	4.73,11.76,-22.25,A,32,14 5.73,10.56,-23.85,P,16,15 2.53,12.56,-
MAPK14	MOL005320	arachidonate	113.108	4.73,11.76,-22.25,A,32,12 5.93,9.56,-19.45,A,45,15 7.13,7.16,-17.05,A,61,18
MAPK14	MOL005321	Frutinone A	79.8533	4.73,11.76,-22.25,A,32,14 4.93,11.96,-18.05,A,54,15 5.93,9.56,-19.45,A,45,18

MAPK14	MOL005348	Ginsenoside-Rh4_qt	87.4445	2.13,11.16,-21.05,A,40,16 9.73,12.76,-23.05,A,26,29
MAPK14	MOL005356	Girinimbin	85.1249	7.33,6.56,-18.25,A,53,1 6.13,7.76,-16.45,A,67,3 4.73,11.76,-
MAPK14	MOL005384	suchilactone	93.2926	2.33,11.96,-10.25,A,99,1 3.93,10.36,-18.85,A,50,20
MAPK14	MOL005399	alexandrin_qt	97.6333	2.13,11.16,-21.05,A,40,10 5.93,8.56,-20.85,A,43,23 8.33,5.76,-17.45,A,58,29
MAPK14	MOL006331	4',5-Dihydroxyflavone	74.2241	5.73,10.76,-23.25,A,22,2 2.53,12.56,-17.45,A,59,13
MAPK14	MOL008647	N-Trans-Feruloyltyramine	105.504	9.73,14.16,-23.25,P,22,2 5.93,8.36,-17.65,A,57,16 7.93,5.76,-15.85,A,69,22
MAPK14	MOL009763	(+)-Syringaresinol-O-beta-D-glucoside	121.029	1.93,10.56,-20.45,P,36,5 5.73,10.56,-23.85,P,16,9 1.33,12.76,-
ESR1	MOL000098	quercetin	100.24	6.25,13.26,-26.51,P,4,17 9.25,16.06,-24.11,P,21,18 8.65,16.26,-21.31,P,69,22
ESR1	MOL000006	luteolin	99.5107	7.25,18.26,-22.11,P,54,10 8.05,15.46,-25.31,A,20,11 5.45,16.06,-
ESR1	MOL000358	beta-sitosterol	110.448	9.45,15.86,-22.51,A,56,19 7.65,18.26,-
ESR1	MOL000359	sitosterol	114.996	9.45,15.86,-22.51,A,56,20 4.65,13.46,-
ESR1	MOL000422	kaempferol	99.0399	8.45,17.86,-22.11,P,53,11 5.45,16.06,-18.11,A,97,17 5.25,14.66,-26.51,P,5,19
ESR1	MOL000449	Stigmasterol	98.4224	8.05,17.46,-25.51,A,16,1 7.25,14.26,-23.31,A,44,6 4.85,15.86,-
ESR1	MOL000622	Magnograndiolide	95.0231	6.85,14.66,-19.51,A,89,9 7.85,12.86,-21.11,A,73,15 6.25,15.06,-26.91,A,1,19
ESR1	MOL000787	Fumarine	92.577	6.05,18.26,-25.51,A,17,12 7.85,15.46,-19.11,A,91,18
ESR1	MOL001510	24-epicampesterol	105.392	4.65,13.46,-19.91,A,85,14 8.65,15.86,-
ESR1	MOL001645	Linoleyl acetate	112.489	7.45,16.26,-25.91,A,10,3 6.85,17.26,-21.31,A,69,9 6.25,12.46,-26.11,A,5,22
ESR1	MOL001771	poriferast-5-en-3beta-ol	121.811	4.85,15.86,-18.91,A,94,14 6.65,13.46,-
ESR1	MOL001792	DFV	96.2617	6.85,17.26,-21.31,A,69,2 7.45,18.66,-23.11,P,33,11 6.25,13.26,-26.51,P,4,19
ESR1	MOL002268	Rhein	92.3996	6.45,16.66,-19.11,A,92,10 7.65,18.26,-23.71,A,43,11 5.45,13.06,-20.51,P,78,15
ESR1	MOL002714	baicalein	100.158	6.05,14.66,-25.11,A,24,4 9.05,17.86,-25.11,P,18,10 5.25,14.06,-

ESR1	MOL002879	Diop	53.9475	6.65,14.66,-<br>17.51,A,99,14<br>7.05,15.86,-
ESR1	MOL002959	3'-<br>Methoxydaidzein	101.374	6.85,17.26,-21.31,A,69,1<br>5.45,16.06,-18.11,A,97,8<br>5.85,13.86,-22.31,P,46,11
ESR1	MOL003044	Chryseriol	103.079	7.25,18.26,-22.11,P,54,10<br>8.05,15.46,-25.31,A,20,11<br>5.45,16.06,-
ESR1	MOL003542	8-Isopentenyl-<br>kaempferol	51.3221	4.85,15.86,-<br>18.91,A,94,16<br>8.25,16.06,-19.91,P,87,19
ESR1	MOL003648	Inermin	94.0881	5.65,16.86,-19.91,P,88,12<br>7.45,15.26,-<br>23.91,A,39,17
ESR1	MOL004367	olivil	119.362	6.85,14.66,-<br>19.51,A,89,12<br>6.65,17.26,-
ESR1	MOL004373	Anhydroicaritin	89.8572	6.25,14.86,-<br>20.91,A,75,22<br>7.25,14.26,-
ESR1	MOL004380	C-<br>Homoerythrinan,<br>1,6-didehydro-	35.254	7.45,15.26,-23.91,A,39,3<br>8.05,12.46,-22.11,A,62,8<br>5.45,16.06,-
ESR1	MOL004382	Yinyanghuo A	119.545	5.65,13.46,-21.11,A,74,14<br>8.85,17.26,-<br>24.31,A,35,19
ESR1	MOL004384	Yinyanghuo C	101.192	7.45,16.26,-25.91,A,10,2<br>7.25,14.66,-<br>18.51,A,95,12
ESR1	MOL004386	Yinyanghuo E	95.0681	7.45,16.26,-25.91,A,10,2<br>7.25,14.66,-<br>18.51,A,95,12
ESR1	MOL004391	8-(3-methylbut-2-<br>enyl)-2-phenyl-<br>chromone	97.812	8.05,17.46,-25.51,A,16,7<br>7.45,16.26,-25.91,A,10,8<br>5.25,14.06,-
ESR1	MOL004396	1,2-bis(4-hydroxy-<br>3-<br>methoxyphenyl)pr	119.061	6.05,15.86,-<br>17.51,A,100,10<br>5.85,17.26,-
ESR1	MOL004941	(2R)-7-hydroxy-2-<br>(4-<br>hydroxyphenyl)chr	98.9323	8.05,17.46,-25.51,A,16,2<br>4.85,15.86,-<br>18.91,A,94,16
ESR1	MOL005308	Aposiopolamine	97.2675	5.85,14.26,-26.31,A,3,3<br>7.05,15.86,-<br>18.31,A,96,13
ESR1	MOL005318	Dianthramine	97.5259	5.25,15.86,-19.51,P,92,15<br>5.45,12.86,-25.51,P,16,18<br>8.65,11.06,-21.71,P,57,21
ESR1	MOL005320	arachidonate	111.066	6.25,14.86,-20.91,A,75,11<br>8.05,17.26,-<br>24.31,A,34,16
ESR1	MOL005321	Frutinone A	103.979	6.45,16.66,-19.11,A,92,6<br>7.45,15.26,-<br>23.91,A,39,14
ESR1	MOL005348	Ginsenoside-<br>Rh4_qt	75.3119	4.65,13.46,-<br>19.91,A,85,14<br>7.25,14.66,-
ESR1	MOL005356	Girinimbin	99.1042	6.05,14.66,-25.11,A,24,15<br>8.05,18.66,-<br>22.31,A,59,18
ESR1	MOL005376	Panaxadiol	101.539	5.45,16.06,-18.11,A,97,15<br>9.05,16.86,-23.11,A,45,21<br>7.45,15.26,-

ESR1	MOL005384	suchilactone	89.3552	8.25,15.86,-18.71,P,95,17 6.25,16.26,- 20.91,A,76,24
ESR1	MOL005399	alexandrin_qt	108.409	6.05,14.46,-25.71,P,14,1 4.45,15.06,-19.51,A,90,11 7.05,16.06,-
ESR1	MOL006331	4',5-Dihydroxyflavone	95.9276	6.05,18.26,-25.51,A,17,1 7.85,16.46,-18.11,A,98,14 6.65,13.46,-24.51,P,19,19
ESR1	MOL008647	N-Trans-Feruloyltyramine	117.519	6.05,14.66,-25.11,A,24,9 7.45,15.66,-26.91,A,2,10 7.25,14.66,-
FOS	MOL000098	quercetin	125.251	62.76,26.56,37.27,A,29,6 58.16,32.76,40.67,P,77,19 64.96,30.36,35.67,P,20,2
FOS	MOL000006	luteolin	122.487	57.56,33.96,40.07,P,74,12 62.76,28.96,39.07,A,63,1 5
FOS	MOL000358	beta-sitosterol	130.632	65.16,32.76,37.47,A,35,6 57.56,35.56,36.47,A,20,2 8
FOS	MOL000359	sitosterol	135.629	59.96,32.16,38.87,A,60,1 1 56.16,33.76,37.67,A,39,1
FOS	MOL000422	kaempferol	117.029	59.36,31.16,42.27,A,86,9 61.76,26.96,37.27,A,30,1 3
FOS	MOL000449	Stigmasterol	131.157	62.36,31.76,37.87,A,43,1 1 56.36,32.96,36.87,A,25,1
FOS	MOL000622	Magnograndiolide	96.1256	63.16,28.96,35.87,A,14,8 60.36,33.36,38.47,A,55,1 2
FOS	MOL000787	Fumarine	126.322	60.56,30.76,39.07,A,65,1 0 61.56,34.16,38.27,A,52,1
FOS	MOL001510	24-epicampesterol	123.932	62.76,26.56,37.27,A,29,1 4 63.36,30.76,36.47,A,18,1
FOS	MOL001525	Daucosterol	111.154	63.36,33.16,37.87,A,44,2 3 56.56,34.36,38.67,A,57,2
FOS	MOL001645	Linoleyl acetate	140.855	59.56,27.16,43.67,A,96,6 56.16,31.76,35.67,A,13,1 6
FOS	MOL001771	poriferast-5-en-3beta-ol	135.036	62.36,35.56,39.07,A,67,1 3 57.76,33.36,38.07,A,48,2
FOS	MOL001792	DFV	118.999	58.96,34.16,39.47,P,65,6 58.56,32.76,41.27,A,84,7 56.16,31.76,37.07,A,26,1
FOS	MOL002268	Rhein	112.418	58.56,32.76,41.27,A,84,8 62.56,29.56,36.87,A,23,1 1
FOS	MOL002714	baicalein	116.469	58.96,34.16,39.47,P,65,10 57.96,30.36,42.47,P,84,14 63.56,28.76,37.67,A,37,1
FOS	MOL002879	Diop	144.593	58.56,29.36,42.87,A,91,5 62.96,30.36,38.27,A,50,1 3
FOS	MOL002959	3'-Methoxydaidzein	127.183	58.76,35.16,37.87,A,45,1 6 56.16,31.76,35.67,A,13,1

FOS	MOL003044	Chryseriol	123.112	62.76,26.56,37.27,A,29,4<br>61.36,34.96,39.07,P,60,21<br>59.16,31.36,39.47,A,73,2
FOS	MOL003542	8-Isopentenyl-<br>kaempferol	138.718	64.96,30.36,35.67,P,20,3<br>64.16,32.96,36.27,P,29,5<br>62.56,27.36,36.27,A,15,1
FOS	MOL003648	Inermin	114.285	59.96,32.16,38.87,A,60,1<br>3<br>63.36,33.16,37.87,A,44,1
FOS	MOL004367	olivil	147.468	65.16,32.76,37.47,A,35,1<br>7<br>58.56,31.76,42.67,A,88,2
FOS	MOL004373	Anhydroicaritin	141.524	57.56,33.96,40.07,P,74,5<br>63.56,28.76,37.67,A,37,1<br>9
FOS	MOL004380	C-<br>Homoerythrinan,<br>1,6-didehydro-	115.984	58.96,31.16,41.27,A,83,1<br>2<br>65.16,32.76,37.47,A,35,2
FOS	MOL004382	Yinyanghuo A	143.083	61.56,34.16,38.27,A,52,2<br>2<br>62.96,30.36,38.27,A,50,2
FOS	MOL004384	Yinyanghuo C	136.631	60.56,30.76,39.07,A,65,1<br>0<br>62.16,27.96,37.87,A,40,1
FOS	MOL004386	Yinyanghuo E	140.28	60.56,30.76,39.07,A,65,1<br>0<br>62.16,27.96,37.87,A,40,1
FOS	MOL004388	6-hydroxy-11,12-<br>dimethoxy-2,2-<br>dimethyl-1,8-<br>dioxo-2,3,4,8-<br>tetrahydro-1H-<br>isochromeno[3,4-	120.826	58.96,34.16,39.47,P,65,19<br>62.16,27.56,34.87,A,6,22<br>65.16,31.76,37.27,A,34,2<br>4
FOS	MOL004391	8-(3-methylbut-2-<br>enyl)-2-phenyl-<br>chromone	136.669	59.96,32.16,38.87,A,60,7<br>58.76,27.56,44.47,A,98,1<br>3
FOS	MOL004396	1,2-bis(4-hydroxy-<br>3-<br>methoxyphenyl)pr	143.42	57.96,34.76,39.27,P,64,4<br>60.96,28.36,39.87,P,72,12<br>57.56,36.76,35.07,P,15,2
FOS	MOL004427	Icariside A7	118.014	63.16,32.56,35.27,P,17,15<br>58.16,32.76,40.67,P,77,30<br>56.36,34.16,38.87,P,59,3
FOS	MOL004941	(2R)-7-hydroxy-2-<br>(4-<br>hydroxyphenyl)chr	120.314	56.76,34.36,35.47,A,12,2<br>59.36,33.16,39.47,A,74,8<br>58.76,30.76,43.47,A,95,1
FOS	MOL005308	Aposiopolamine	108.168	59.36,31.16,42.27,A,86,3<br>62.76,28.56,35.07,A,7,13<br>63.56,27.56,37.27,A,31,1
FOS	MOL005317	Deoxyharringtonin<br>e	110.581	57.36,35.56,35.27,A,11,5<br>62.76,33.56,36.27,P,31,12<br>63.36,35.16,39.47,P,66,1
FOS	MOL005318	Dianthramine	114.202	58.16,34.36,39.27,A,70,2<br>58.76,35.76,37.47,P,46,7<br>58.16,30.96,41.27,P,79,1
FOS	MOL005320	arachidonate	144.383	54.76,32.16,35.27,A,9,6<br>59.56,30.16,40.27,A,77,1<br>0
FOS	MOL005321	Frutinone A	107.471	63.56,28.76,37.67,A,37,2<br>63.36,30.76,36.47,A,18,6<br>57.56,32.36,42.67,A,89,1
FOS	MOL005348	Ginsenoside-<br>Rh4_qt	102.471	61.56,34.16,38.27,A,52,1<br>62.76,26.56,37.27,A,29,1<br>7

FOS	MOL005356	Girinimbin	112.005	58.96,31.16,41.27,A,83,3<br>59.36,33.16,39.47,A,74,6<br>63.56,27.56,37.27,A,31,1
FOS	MOL005356	Girinimbin	112.005	58.96,31.16,41.27,A,83,3<br>59.36,33.16,39.47,A,74,6<br>63.56,27.56,37.27,A,31,1
FOS	MOL005376	Girinimbin	92.8526	59.16,31.36,39.47,A,73,2<br>0<br>60.76,35.76,37.67,P,49,25
FOS	MOL005384	suchilactone	143.332	58.56,29.36,42.87,A,91,1<br>6<br>60.36,34.56,39.07,A,66,2
FOS	MOL005399	alexandrin_qt	122.17	63.96,30.16,38.87,A,59,1<br>2<br>62.76,34.76,38.67,A,58,1
FOS	MOL006331	4',5-Dihydroxyflavone	117.71	62.96,30.36,38.27,A,50,1<br>58.96,31.16,41.27,A,83,8<br>57.56,27.76,46.27,P,99,1
FOS	MOL008647	N-Trans-Feruloyltyramine	142.776	54.96,32.96,37.07,A,27,1<br>2<br>58.56,29.36,42.87,A,91,1
FOS	MOL009763	(+)-Syringaresinol-O-beta-D-glucoside	130.07	65.96,32.96,38.27,P,54,1<br>61.56,26.76,34.07,P,6,9<br>62.76,34.76,38.67,A,58,1
IL-6	MOL000098	quercetin	98.9915	20.51,31.12,6.55,A,88,12<br>20.71,29.72,6.35,A,84,13<br>27.31,36.32,5.55,P,87,17
IL-6	MOL000006	luteolin	112.193	19.11,34.92,3.15,A,56,4<br>20.51,31.12,6.55,A,88,16<br>20.71,29.72,6.35,A,84,18
IL-6	MOL000422	kaempferol	103.126	20.71,29.72,6.35,A,84,16<br>20.51,31.12,6.55,A,88,17<br>27.31,36.32,5.55,P,87,19
IL-6	MOL000622	Magnograndiolide	84.322	21.91,32.32,5.15,A,77,12<br>19.31,38.12,3.15,A,58,16<br>19.11,35.92,2.75,P,67,17
IL-6	MOL000787	Fumarine	77.1172	21.91,32.32,5.15,A,77,18<br>19.71,35.32,4.95,A,74,20<br>21.31,33.92,3.35,A,59,21
IL-6	MOL001645	Linoleyl acetate	129.582	17.91,22.92,-4.85,A,14,1<br>18.51,20.52,-5.05,A,11,3<br>24.91,20.52,-
IL-6	MOL001792	DFV	101.684	19.51,36.12,3.55,A,63,9<br>21.31,28.72,6.75,A,98,14<br>20.51,35.72,1.55,P,55,19
IL-6	MOL002268	Rhein	91.1466	20.71,37.52,3.15,A,57,11<br>20.51,30.12,5.15,A,76,17<br>20.11,34.92,2.95,P,69,20
IL-6	MOL002714	baicalein	108.912	19.11,34.92,3.15,A,56,4<br>20.51,31.12,6.55,A,88,17<br>20.71,29.72,6.35,A,84,19
IL-6	MOL002879	Diop	86.2024	28.51,36.92,3.35,A,61,19<br>26.51,30.72,1.35,A,47,24<br>25.51,28.72,-
IL-6	MOL002959	3'-Methoxydaidzein	92.7741	21.11,31.12,4.95,A,71,5<br>22.11,33.52,3.35,P,71,11<br>20.71,37.52,3.15,A,57,16
IL-6	MOL003044	Chryseriol	94.6263	20.51,37.12,2.35,P,64,14<br>20.51,31.12,6.55,A,88,15<br>19.71,28.92,6.35,P,96,20
IL-6	MOL003542	8-Isopentenyl-kaempferol	116.066	19.91,32.32,3.95,P,76,3<br>20.11,34.92,2.95,P,69,5<br>25.71,35.92,6.75,A,100,2

IL-6	MOL003648	Inermin	108.857	22.51,27.12,6.75,P,99,3 21.31,35.72,3.75,A,65,17 19.11,34.92,3.15,A,56,19
IL-6	MOL004367	olivil	111.767	27.31,36.32,5.55,P,87,6 21.91,32.32,5.15,A,77,13 19.51,36.12,3.55,A,63,24
IL-6	MOL004373	Anhydroicaritin	123.662	19.91,32.32,3.95,P,76,1 20.11,34.92,2.95,P,69,3 25.71,35.92,6.75,A,100,2
IL-6	MOL004380	C-Homoerythrinan, 1,6-didehydro-	99.0789	21.11,31.12,4.95,A,71,9 21.11,34.12,4.95,A,73,16 20.71,37.52,3.15,A,57,24
IL-6	MOL004382	Yinyanghuo A	85.489	22.51,27.12,6.75,P,99,6 25.71,35.92,6.75,A,100,15
IL-6	MOL004386	Yinyanghuo E	91.0784	25.71,35.92,6.75,A,100,3 20.11,30.32,4.95,P,83,22 21.51,25.92,6.55,A,87,25
IL-6	MOL004388	dioxo-2,3,4,8-tetrahydro-1H-isochromeno[3,4-h]isoquinolin-2-	84.2676	19.11,34.92,3.15,A,56,2 25.71,35.92,6.75,A,100,21 20.71,29.72,6.35,A,84,26
IL-6	MOL004391	8-(3-methylbut-2-enyl)-2-phenyl-chromone	98.6761	18.51,34.52,2.95,P,68,4 19.31,37.12,4.35,A,69,6 20.71,29.72,6.35,A,84,16
IL-6	MOL004396	1,2-bis(4-hydroxy-3-methoxyphenyl)pr	111.877	20.51,31.12,6.55,A,88,3 21.51,35.12,2.15,P,62,12 19.11,35.92,2.75,P,67,14
IL-6	MOL004941	(2R)-7-hydroxy-2-(4-hydroxyphenyl)chr	105.642	20.71,29.72,6.35,A,84,15 20.51,31.12,6.55,A,88,16 20.51,37.12,2.35,P,64,19
IL-6	MOL005308	Aposiopolamine	95.5172	19.91,28.72,6.75,A,97,1 21.31,28.72,6.75,A,98,6 19.11,35.92,2.75,P,67,18
IL-6	MOL005318	Dianthramine	78.2815	25.71,35.92,6.75,A,100,3 20.51,31.12,6.55,A,88,12 18.91,33.72,3.75,P,74,21
IL-6	MOL005320	arachidonate	125.93	24.91,20.52,-2.05,A,38,10 16.11,22.12,-3.45,A,26,19
IL-6	MOL005321	Frutinone A	95.593	25.71,35.92,6.75,A,100,1 20.71,32.72,4.15,A,68,14 19.91,28.72,6.75,A,97,17
IL-6	MOL005356	Girinimbin	107.238	19.91,28.72,6.75,A,97,2 21.11,36.52,2.15,A,52,19 19.31,38.12,3.15,A,58,20
IL-6	MOL005384	suchilactone	108.15	19.31,37.12,4.35,A,69,7 20.51,31.12,6.55,A,88,18 19.91,28.72,6.75,A,97,24
IL-6	MOL005399	alexandrin_qt	82.379	19.31,37.12,4.35,A,69,14 20.71,29.72,6.35,A,84,19 26.71,35.52,6.55,A,89,29
IL-6	MOL006331	4',5-Dihydroxyflavone	98.5775	20.71,37.52,3.15,A,57,2 20.31,35.12,2.75,A,54,6 20.71,29.72,6.35,A,84,14
IL-6	MOL008647	N-Trans-Feruloyltyramine	126.247	22.31,26.52,6.75,A,95,11 21.11,34.12,4.95,A,73,17 21.11,36.52,2.15,A,52,20
MYC	MOL000098	quercetin	85.4999	44.60,43.33,6.97,A,33,12 42.60,46.33,7.17,P,41,19 47.60,39.73,4.37,P,23,22

MYC	MOL000006	luteolin	92.1022	47.00,41.53,5.37,A,24,9<br>42.80,43.13,7.57,A,40,11<br>42.40,45.53,7.97,P,47,14
MYC	MOL000358	beta-sitosterol	103.46	47.00,41.53,5.37,A,24,13<br>44.20,43.93,7.37,A,38,21<br>45.40,42.73,9.17,P,53,25
MYC	MOL000359	sitosterol	101.7	44.60,42.13,7.77,A,42,6<br>47.80,40.73,4.97,A,20,10<br>42.40,45.53,7.97,P,47,30
MYC	MOL000422	kaempferol	79.1184	47.60,39.73,4.37,P,23,11<br>44.60,43.33,6.97,A,33,17<br>42.60,46.33,7.17,P,41,18
MYC	MOL000449	Stigmasterol	96.756	44.20,43.93,7.37,A,38,2<br>47.80,40.73,4.97,A,20,7<br>45.40,42.73,9.17,P,53,30
MYC	MOL000622	Magnograndiolide	73.8574	44.60,43.33,6.97,A,33,6<br>45.40,42.73,9.17,P,53,17<br>47.80,40.73,4.97,A,20,19
MYC	MOL000787	Fumarine	93.7856	44.60,43.33,6.97,A,33,14<br>45.20,43.13,5.57,A,26,18<br>48.80,39.73,4.57,P,24,25
MYC	MOL001510	24-epicampesterol	101.409	44.60,43.33,8.77,A,57,2<br>44.60,42.13,7.77,A,42,3<br>46.40,41.33,6.37,A,28,7
MYC	MOL001525	Daucosterol	88.3966	47.80,40.73,4.97,A,20,10<br>44.60,42.13,7.77,A,42,20<br>44.60,43.33,8.77,A,57,22
MYC	MOL001645	Linoleyl acetate	99.9731	44.60,42.13,7.77,A,42,7<br>46.40,41.33,6.37,A,28,10<br>48.80,39.73,3.57,A,17,17
MYC	MOL001771	poriferast-5-en-3beta-ol	100.606	44.20,43.93,7.37,A,38,1<br>47.80,40.73,4.97,A,20,8<br>45.40,42.73,9.17,P,53,15
MYC	MOL001792	DFV	84.5383	45.80,42.13,7.17,A,35,2<br>43.40,44.73,8.17,A,51,10<br>48.60,39.93,4.57,A,18,14
MYC	MOL002268	Rhein	60.192	45.20,43.13,5.57,A,26,11<br>44.40,42.33,6.37,A,29,12<br>44.60,42.13,7.77,A,42,13
MYC	MOL002714	baicalein	81.2363	44.20,43.93,7.37,A,38,4<br>42.40,45.53,7.97,P,47,14<br>48.80,39.73,3.57,A,17,18
MYC	MOL002879	Diop	90.2087	50.80,38.93,14.37,A,83,1<br>9<br>44.80,42.13,4.77,A,19,25
MYC	MOL002959	3'-Methoxydaidzein	80.1748	45.80,42.13,7.17,A,35,1<br>43.40,44.73,8.17,A,51,8<br>48.60,39.93,4.57,A,18,16
MYC	MOL003044	Chryseriol	92.086	45.60,42.13,5.77,A,27,9<br>42.20,44.13,7.77,P,44,12<br>47.80,40.73,4.97,A,20,16
MYC	MOL003542	8-Isopentenyl-kaempferol	86.1744	45.20,42.93,8.97,A,59,22<br>42.80,43.13,7.57,A,40,25<br>44.20,42.13,5.37,A,25,26
MYC	MOL003648	Inermin	98.7157	48.80,39.73,4.57,P,24,5<br>47.00,41.53,5.37,A,24,6<br>44.60,42.13,7.77,A,42,13
MYC	MOL004367	olivil	104.107	44.40,42.53,8.37,P,48,2<br>45.40,41.33,4.57,P,25,3<br>49.00,39.53,3.37,P,20,4
MYC	MOL004373	Anhydroicaritin	84.9107	45.40,42.73,9.17,P,53,1<br>47.60,39.73,4.37,P,23,6<br>48.80,39.73,3.57,A,17,27

MYC	MOL004380	C-Homoerythrinan, 1,6-didehydro-	85.2259	44.80,42.13,4.77,A,19,7 45.20,42.93,8.97,A,59,12 48.80,39.73,3.57,A,17,20
MYC	MOL004382	Yinyanghuo A	94.2848	45.80,42.13,7.17,A,35,12 43.40,42.13,6.97,A,32,18 44.80,42.13,4.77,A,19,19
MYC	MOL004384	Yinyanghuo C	79.2083	45.60,42.33,8.17,A,50,18 47.00,41.53,5.37,A,24,24 44.80,42.13,4.77,A,19,25
MYC	MOL004386	Yinyanghuo E	80.1774	45.80,42.13,7.17,A,35,23 44.80,42.13,4.77,A,19,25 43.40,42.13,6.97,A,32,26
MYC	MOL004388	6-hydroxy-11,12-dimethoxy-2,2-dimethyl-1,8-dioxo-2,3,4,8-tetrahydro-1H-isochromeno[3,4-	76.247	45.60,42.13,5.77,A,27,17 42.20,44.13,7.77,P,44,19 47.80,40.73,4.97,A,20,26
MYC	MOL004391	8-(3-methylbut-2-enyl)-2-phenyl-chromone	92.0853	46.20,42.53,4.97,A,22,2 43.80,42.13,4.97,P,28,4 43.20,45.33,7.97,A,49,7
MYC	MOL004394	Anhydroicaritin-3-O-alpha-L-rhamnoside	85.4353	42.80,43.13,7.57,A,40,7 46.40,40.73,4.77,P,26,9 43.80,42.13,4.97,P,28,11
MYC	MOL004396	1,2-bis(4-hydroxy-3-methoxyphenyl)pr	87.3239	44.60,42.13,7.77,A,42,3 44.20,41.53,8.97,P,52,5 46.00,41.13,4.97,A,21,20
MYC	MOL004425	Icariin	76.0302	47.80,40.73,4.97,A,20,25 43.20,45.33,7.97,A,49,28 40.60,45.13,7.57,A,41,47
MYC	MOL004427	Icariside A7	84.9489	48.80,39.73,3.57,A,17,17 44.80,42.13,4.77,A,19,18 42.20,44.13,7.77,P,44,20
MYC	MOL004941	(2R)-7-hydroxy-2-(4-hydroxyphenyl)chr	81.4326	43.40,42.13,6.97,A,32,13 48.80,39.73,4.57,P,24,17 42.20,44.13,7.77,P,44,18
MYC	MOL005308	Aposiopolamine	78.7358	45.60,42.33,8.17,A,50,13 47.00,41.53,5.37,A,24,14 45.60,42.13,5.77,A,27,15
MYC	MOL005317	Deoxyharringtonine	100.85	48.80,39.73,4.57,P,24,28 45.20,43.13,5.57,A,26,32 44.60,43.33,6.97,A,33,34
MYC	MOL005318	Dianthramine	62.7779	43.20,45.33,7.97,A,49,2 47.80,40.73,4.97,A,20,12 46.20,42.53,4.97,A,22,14
MYC	MOL005320	arachidonate	100.372	37.20,45.13,11.57,A,71,5 38.00,46.33,11.97,A,73,6 45.80,42.13,7.17,A,35,17
MYC	MOL005321	Frutinone A	81.5946	48.80,39.73,3.57,A,17,3 45.80,42.13,7.17,A,35,15 44.60,43.33,8.77,A,57,17
MYC	MOL005348	Ginsenoside-Rh4_qt	89.3999	47.00,41.53,5.37,A,24,26 48.60,39.93,4.57,A,18,28 42.80,43.13,7.57,A,40,30
MYC	MOL005356	Girinimbin	89.0469	48.80,39.73,3.57,A,17,1 45.60,42.13,5.77,A,27,13 43.40,42.13,6.97,A,32,18
MYC	MOL005376	Panaxadiol	81.1163	44.20,42.13,5.37,A,25,20 42.20,44.13,7.97,A,47,29 43.80,42.53,8.57,A,55,30
MYC	MOL005384	suchilactone	97.4792	47.00,41.53,5.37,A,24,10 45.20,43.13,5.57,A,26,11 44.60,43.33,6.97,A,33,12

MYC	MOL005399	alexandrin_qt	97.4024	46.00,41.13,4.97,A,21,14<br>43.20,45.33,7.97,A,49,18<br>48.60,39.93,4.57,A,18,23
MYC	MOL006331	4',5-Dihydroxyflavone	56.235	43.40,42.13,6.97,A,32,2<br>45.80,42.13,7.17,A,35,6<br>42.20,44.13,7.77,P,44,18
MYC	MOL008647	N-Trans-Feruloyltyramine	100.138	48.80,39.73,4.57,P,24,3<br>42.20,44.13,7.77,P,44,4<br>43.40,42.13,6.97,A,32,22
MYC	MOL009763	(+)-Syringaresinol-O-beta-D-glucoside	120.575	45.40,41.33,4.57,P,25,9<br>45.60,42.13,5.77,A,27,32<br>48.80,39.73,3.57,A,17,39
CDKN1A	MOL000098	quercetin	67.6121	-25.34,48.97,-<br>14.31,A,63,2<br>-26.74,49.37,-
CDKN1A	MOL000006	luteolin	98.408	-32.34,56.17,-23.31,A,7,4<br>-30.54,52.37,-<br>23.91,P,12,12
CDKN1A	MOL000358	beta-sitosterol	81.6822	-36.54,57.97,-<br>23.91,A,4,10<br>-31.14,54.17,-
CDKN1A	MOL000359	sitosterol	98.6929	-32.94,54.97,-24.71,A,3,7<br>-31.54,54.57,-<br>22.11,A,14,10
CDKN1A	MOL000422	kaempferol	66.3948	-26.74,49.37,-<br>12.31,A,73,7<br>-25.34,48.97,-
CDKN1A	MOL000449	Stigmasterol	95.1326	-31.14,58.77,-<br>10.91,A,81,7<br>-31.74,58.17,-
CDKN1A	MOL000622	Magnograndiolide	85.7204	-31.54,54.37,-<br>20.51,A,27,5<br>-32.94,57.17,-
CDKN1A	MOL000787	Fumarine	91.1649	-31.14,54.17,-<br>24.91,A,2,18<br>-31.94,55.97,-
CDKN1A	MOL001510	24-epicampesterol	86.1383	-31.14,58.77,-<br>10.91,A,81,8<br>-31.74,58.17,-
CDKN1A	MOL001525	Daucosterol	83.2616	-36.54,57.97,-<br>23.91,A,4,14<br>-31.74,54.57,-
CDKN1A	MOL001645	Linoleyl acetate	114.894	-31.54,54.37,-<br>20.51,A,27,3<br>-32.34,56.17,-23.31,A,7,6
CDKN1A	MOL001771	poriferast-5-en-3beta-ol	94.4304	-36.54,57.97,-<br>23.91,A,4,16<br>-31.94,55.97,-
CDKN1A	MOL001792	DFV	107.676	-26.74,53.97,-9.11,A,87,1<br>-26.14,51.17,-<br>11.71,A,77,7
CDKN1A	MOL002268	Rhein	92.9233	-30.94,58.97,-<br>18.11,A,36,10<br>-30.74,53.37,-
CDKN1A	MOL002714	baicalein	88.3563	-31.34,56.37,-<br>15.11,A,55,4<br>-31.54,54.57,-
CDKN1A	MOL002879	Diop	103.647	-37.74,58.17,-<br>22.91,A,9,13<br>-31.94,55.97,-
CDKN1A	MOL002959	3'-Methoxydaidzein	98.0434	-26.14,51.17,-<br>11.71,A,77,1<br>-25.34,47.17,-

CDKN1A	MOL003044	Chryseriol	99.4597	-32.34,56.17,-23.31,A,7,4 -30.54,52.37,-23.91,P,12,12
CDKN1A	MOL003542	8-Isopentenyl-kaempferol	106.006	-36.54,57.97,-23.91,A,4,17 -31.54,54.37,-
CDKN1A	MOL003648	Inermin	101.293	-29.74,52.97,-20.91,A,24,4 -31.94,55.97,-
CDKN1A	MOL004367	olivil	115.036	-39.14,57.97,-23.31,P,17,6 -32.34,56.17,-
CDKN1A	MOL004373	Anhydroicaritin	94.3067	-31.74,54.57,-23.51,A,6,18 -38.74,59.77,-
CDKN1A	MOL004380	C-Homoerythrinan, 1,6-didehydro-	80.488	-31.54,54.37,-20.51,A,27,9 -31.34,55.97,-
CDKN1A	MOL004382	Yinyanghuo A	121.532	-32.34,56.17,-23.31,A,7,16 -38.74,59.77,-
CDKN1A	MOL004384	Yinyanghuo C	89.1586	-30.34,62.97,-13.51,P,64,19 -30.74,58.57,-
CDKN1A	MOL004386	Yinyanghuo E	107.43	-30.14,58.37,-7.31,P,99,19 -28.14,56.17,-
CDKN1A	MOL004388	6-hydroxy-11,12-dimethoxy-2,2-dimethyl-1,8-dioxo-2,3,4,8-tetrahydro-1H-isochromeno[3,4-	80.8365	-31.74,54.57,-23.51,A,6,9 -29.74,52.97,-20.91,A,24,12 -28.14,56.17,-16.11,P,40,19
CDKN1A	MOL004391	8-(3-methylbut-2-enyl)-2-phenyl-chromone	102.78	-30.54,52.37,-23.91,P,12,4 -32.34,52.57,-
CDKN1A	MOL004394	Anhydroicaritin-3-O-alpha-L-rhamnoside	94.7532	-31.54,54.37,-20.51,A,27,4 -31.14,54.17,-24.91,A,2,7
CDKN1A	MOL004396	1,2-bis(4-hydroxy-3-methoxyphenyl)pr	113.497	-24.74,50.17,-13.51,A,69,10 -24.74,50.97,-
CDKN1A	MOL004427	Icariside A7	92.2454	-37.74,58.17,-22.91,A,9,10 -41.54,61.57,-
CDKN1A	MOL004941	(2R)-7-hydroxy-2-(4-hydroxyphenyl)chr	91.0743	-28.34,58.97,-19.91,A,30,2 -29.74,54.17,-
CDKN1A	MOL005308	Aposiopolamine	92.8925	-31.14,54.17,-24.91,A,2,3 -36.54,57.97,-23.91,A,4,14
CDKN1A	MOL005317	Deoxyharringtonine	80.4613	-30.94,58.97,-18.11,A,36,27 -32.34,56.17,-
CDKN1A	MOL005318	Dianthramine	80.5083	-31.74,51.97,-19.91,A,29,2 -32.94,57.17,-
CDKN1A	MOL005320	arachidonate	125.898	-38.74,59.77,-21.51,A,21,8 -30.74,53.37,-
CDKN1A	MOL005321	Frutinone A	88.5711	-36.54,57.97,-23.91,A,4,2 -30.34,53.97,-25.71,P,7,13

CDKN1A	MOL005356	Girinimbin	94.8741	-32.94,57.17,- 22.11,A,15,13 -30.74,53.37,-
CDKN1A	MOL005384	suchilactone	106.599	-24.74,65.17,-9.71,P,82,6 -30.14,58.77,- 10.31,P,80,17
CDKN1A	MOL005399	alexandrin_qt	108.388	-24.34,54.97,-8.51,A,93,9 -25.14,50.77,- 10.51,A,82,19
CDKN1A	MOL006331	4',5-Dihydroxyflavone	89.8052	-22.94,54.97,-7.71,A,99,1 -24.74,50.97,- 12.31,A,74,13
CDKN1A	MOL008647	N-Trans-Feruloyltyramine	123.161	-22.54,53.37,- 8.11,A,94,12 -26.14,51.17,-
CDKN1A	MOL009763	(+)-Syringaresinol-O-beta-D-glucoside	113.64	-28.54,65.37,- 12.71,P,73,13 -31.74,58.17,-
RB1	MOL000098	quercetin	80.9989	- 10.52,37.05,24.99,A,53,6 -9.12,43.85,23.99,P,44,21
RB1	MOL000006	luteolin	90.9993	- 14.32,39.45,20.39,P,21,10 -
RB1	MOL000358	beta-sitosterol	94.9363	- 12.92,38.26,22.19,A,38,6 -
RB1	MOL000359	sitosterol	92.3485	-11.12,36.66,26.39,A,56,2 - 12.12,37.66,25.79,A,55,3
RB1	MOL000422	kaempferol	87.8474	0.88,38.85,29.99,P,70,11 - 3.72,36.85,28.59,A,65,17
RB1	MOL000449	Stigmasterol	99.688	- 10.52,36.45,23.59,A,48,1 -
RB1	MOL000622	Magnograndiolide	75.9954	2.08,37.05,29.99,A,80,8 0.68,39.26,31.39,P,77,17 -0.72,31.86,33.79,P,88,18
RB1	MOL000787	Fumarine	62.874	- 12.12,37.66,25.79,A,55,2 0
RB1	MOL001510	24-epicampesterol	95.0305	- 10.12,35.85,25.39,A,54,2 -
RB1	MOL001525	Daucosterol	89.2167	- 12.12,37.66,25.79,A,55,1 6
RB1	MOL001645	Linoleyl acetate	115.939	2.08,37.05,29.99,A,80,1 -4.92,36.26,29.19,A,71,7 -
RB1	MOL001771	poriferast-5-en-3beta-ol	97.3357	- 10.12,35.85,25.39,A,54,2 -
RB1	MOL001792	DFV	85.772	- 12.12,36.85,22.39,A,40,2 -
RB1	MOL002268	Rhein	81.0317	2.28,36.05,30.99,A,88,8 2.28,38.66,30.39,P,73,18 0.08,30.86,34.79,P,94,21
RB1	MOL002714	baicalein	86.0045	- 12.92,38.26,22.19,A,38,1 1

RB1	MOL002879	Diop	94.6178	- 10.52,37.05,24.99,A,53,1 4
RB1	MOL002959	3'-Methoxydaidzein	97.4539	- 14.12,37.66,21.79,P,30,10 -
RB1	MOL003044	Chryseriol	86.6156	- 14.32,39.45,20.39,P,21,10 -
RB1	MOL003542	8-Isopentenyl-kaempferol	92.4392	-14.32,39.45,20.39,P,21,3 - 10.92,38.05,22.19,A,37,1
RB1	MOL003648	Inermin	86.3126	-14.32,39.45,20.39,P,21,5 - 10.52,36.45,23.59,A,48,1
RB1	MOL004367	olivil	113.857	0.48,38.66,32.79,P,83,3 -6.32,36.26,31.59,P,79,7 -
RB1	MOL004373	Anhydroicaritin	99.5343	-14.32,39.45,20.39,P,21,1 - 12.12,36.85,22.39,A,40,2
RB1	MOL004380	C-Homoerythrinan, 1,6-didehydro-	87.8053	2.28,36.05,30.99,A,88,7 1.28,38.26,30.99,A,90,9 -
RB1	MOL004382	Yinyanghuo A	114.203	- 12.12,36.85,22.39,A,40,1 6
RB1	MOL004384	Yinyanghuo C	97.7447	- 12.32,32.85,22.79,A,44,2 -
RB1	MOL004386	Yinyanghuo E	99.5916	- 12.32,32.85,22.79,A,44,2 -
RB1	MOL004388	6-hydroxy-11,12-dimethoxy-2,2-dimethyl-1,8-dioxo-2,3,4,8-tetrahydro-1H-isochromeno[3,4-	74.5749	-0.72,31.86,33.79,P,88,1 0.28,34.66,29.19,A,69,10 2.08,37.05,29.99,A,80,21
RB1	MOL004391	8-(3-methylbut-2-enyl)-2-phenyl-chromone	96.5146	-14.32,39.45,20.39,P,21,4 - 11.92,37.45,24.19,A,50,1
RB1	MOL004394	Anhydroicaritin-3-O-alpha-L-rhamnoside	109.632	- 10.32,41.45,22.19,P,35,29 -
RB1	MOL004396	1,2-bis(4-hydroxy-3-methoxyphenyl)pr	98.3575	- 10.92,38.05,22.19,A,37,3 -
RB1	MOL004425	Icariin	80.4506	- 0.32,31.25,34.59,A,97,26 0.08,34.45,30.99,A,87,39
RB1	MOL004427	Icariside A7	82.0436	- 12.92,38.26,22.19,A,38,1 7
RB1	MOL004941	(2R)-7-hydroxy-2-(4-hydroxyphenyl)chr	82.3028	0.08,34.45,30.99,A,87,9 1.28,38.26,30.99,A,90,15 -0.12,40.05,30.19,P,71,18
RB1	MOL005308	Aposiopolamine	83.1305	-11.52,35.66,24.59,A,52,1 - 12.12,36.85,22.39,A,40,3
RB1	MOL005317	Deoxyharringtonine	119.259	-4.92,36.26,29.19,A,71,5 2.48,34.45,31.39,A,92,22 -

RB1	MOL005318	Dianthramine	74.8458	-9.72,43.26,24.79,P,48,7<br>-<br>13.32,39.85,21.99,A,33,1
RB1	MOL005320	arachidonate	113.487	-2.32,36.26,29.39,A,73,8<br>-<br>4.92,36.26,29.19,A,71,10
RB1	MOL005321	Frutinone A	94.0331	-<br>13.32,39.85,21.99,A,33,3<br>-
RB1	MOL005348	Ginsenoside-Rh4_qt	96.9637	0.08,34.45,30.99,A,87,19<br>-<br>3.72,36.85,28.59,A,65,29
RB1	MOL005356	Girinimbin	90.4803	-<br>10.52,37.05,24.99,A,53,1<br>3
RB1	MOL005376	Panaxadiol	71.1091	-<br>10.52,35.26,26.59,A,57,2<br>9
RB1	MOL005384	suchilactone	99.207	-<br>13.52,33.66,21.19,A,21,1<br>-7.92,33.45,21.99,A,32,2
RB1	MOL005399	alexandrin_qt	107.808	3.88,37.66,28.99,P,61,1<br>-<br>0.92,34.05,29.79,A,76,12
RB1	MOL006331	4',5-Dihydroxyflavone	84.7517	-<br>12.92,38.26,22.19,A,38,8<br>-
RB1	MOL008647	N-Trans-Feruloyltyramine	108.949	-8.52,37.26,27.99,P,54,3<br>-6.52,36.05,29.19,A,70,7<br>-
RB1	MOL009763	(+)-Syringaresinol-O-beta-D-glucoside	106.38	3.88,38.45,30.79,P,75,10<br>2.08,37.05,29.99,A,80,32<br>-
Caspase-3	MOL000098	quercetin	115.29	18.36,-6.01,13.46,A,89,6<br>18.16,-1.41,11.46,A,77,12<br>17.57,1.19,9.06,P,65,19
Caspase-3	MOL000006	luteolin	111.879	14.96,-1.41,16.66,A,94,4<br>16.77,0.39,10.06,P,74,20<br>19.16,-0.21,9.06,P,64,21
Caspase-3	MOL000358	beta-sitosterol	107.789	17.36,-0.41,12.86,A,87,6<br>15.56,0.79,17.26,A,95,23<br>21.57,-8.01,6.66,A,38,28
Caspase-3	MOL000359	sitosterol	105.495	15.96,-0.41,15.86,A,91,4<br>22.57,-8.01,5.26,A,29,19<br>18.96,-
Caspase-3	MOL000422	kaempferol	104.356	18.36,-6.01,13.46,A,89,7<br>18.16,-1.41,11.46,A,77,13<br>18.57,-0.81,8.86,A,54,16
Caspase-3	MOL000449	Stigmasterol	111.693	15.96,-0.41,15.86,A,91,1<br>19.96,-<br>3.81,10.06,A,65,10
Caspase-3	MOL000622	Magnograndiolide	65.6427	14.96,-<br>1.41,16.66,A,94,15<br>16.16,0.79,12.06,P,84,18
Caspase-3	MOL000787	Fumarine	96.1825	17.36,-<br>0.41,12.86,A,87,10<br>19.36,-0.01,9.86,A,61,12
Caspase-3	MOL001510	24-epicampesterol	103.811	15.56,0.79,17.26,A,95,2<br>17.36,-0.41,12.86,A,87,8<br>22.96,-7.61,6.86,A,41,26
Caspase-3	MOL001525	Daucosterol	92.5653	21.96,-8.81,7.66,A,45,24<br>21.57,-7.21,5.66,A,33,27<br>22.96,-9.41,4.06,A,24,28

Caspase-3	MOL001645	Linoleyl acetate	117.69	17.77,0.19,10.06,A,67,2<br>18.96,-2.21,9.86,A,60,4<br>22.96,-7.61,6.86,A,41,14
Caspase-3	MOL001771	poriferast-5-en-<br>3beta-ol	109.904	15.96,-0.41,15.86,A,91,2<br>18.16,-1.41,11.46,A,77,8<br>22.96,-9.21,6.26,A,36,26
Caspase-3	MOL001792	DFV	103.397	14.56,-<br>3.01,16.46,A,92,13<br>14.37,-
Caspase-3	MOL002268	Rhein	100.773	18.57,-0.81,8.86,A,54,8<br>19.96,-2.61,9.46,P,69,15<br>16.36,0.19,13.46,P,89,20
Caspase-3	MOL002714	baicalein	87.2431	18.36,-6.01,13.46,A,89,4<br>18.96,-2.21,9.86,A,60,17<br>17.77,0.19,10.06,A,67,18
Caspase-3	MOL002879	Diop	114.924	19.96,-<br>3.81,10.06,A,65,14<br>17.36,-
Caspase-3	MOL002959	3'-<br>Methoxydaidzein	102.841	18.36,-4.41,13.06,A,88,1<br>19.96,-<br>3.81,10.06,A,65,17
Caspase-3	MOL003044	Chryseriol	96.6152	17.36,-0.41,12.86,A,87,9<br>19.16,-0.21,9.06,P,64,12<br>15.56,0.79,17.26,A,95,22
Caspase-3	MOL003542	8-Isopentenyl-<br>kaempferol	116.813	17.57,1.19,9.06,P,65,19<br>19.36,-0.01,9.86,A,61,20<br>18.36,-
Caspase-3	MOL003648	Inermin	82.5628	17.77,1.19,9.06,A,57,4<br>18.16,-1.41,11.46,A,77,6<br>18.36,-
Caspase-3	MOL004367	olivil	122.487	18.16,-1.41,11.46,A,77,16<br>18.57,-0.81,8.86,A,54,21<br>21.96,-8.81,7.66,A,45,27
Caspase-3	MOL004373	Anhydroicaritin	117.181	16.16,0.79,12.06,P,84,3<br>14.96,-<br>1.41,16.66,A,94,19
Caspase-3	MOL004380	C-<br>Homoerythrinan,<br>1,6-didehydro-	88.2126	19.96,-3.81,10.06,A,65,7<br>18.16,0.99,12.06,P,86,21<br>18.57,-
Caspase-3	MOL004382	Yinyanghuo A	143.016	19.96,-<br>3.81,10.06,A,65,22<br>21.96,-8.81,7.66,A,45,26
Caspase-3	MOL004384	Yinyanghuo C	109.118	18.57,-0.81,8.86,A,54,2<br>14.96,-<br>1.41,16.66,A,94,13
Caspase-3	MOL004386	Yinyanghuo E	113.222	21.57,-8.01,6.66,A,38,3<br>18.96,-2.21,9.86,A,60,18<br>17.36,-
Caspase-3	MOL004388	6-hydroxy-11,12-<br>dimethoxy-2,2-<br>dimethyl-1,8-<br>dioxo-2,3,4,8-<br>tetrahydro-1H-<br>isochromeno[3,4-	88.9282	16.36,0.19,13.46,P,89,19<br>18.36,-<br>6.01,13.46,A,89,24<br>17.57,-1.61,9.06,P,63,27
Caspase-3	MOL004391	8-(3-methylbut-2-<br>enyl)-2-phenyl-<br>chromone	98.5387	19.96,-3.81,10.06,A,65,2<br>17.16,-0.01,11.66,A,81,7<br>14.96,-
Caspase-3	MOL004394	Anhydroicaritin-3-<br>O-alpha-L-<br>rhamnoside	116.682	14.56,-<br>3.01,16.46,A,92,27<br>19.96,-
Caspase-3	MOL004396	1,2-bis(4-hydroxy-<br>3-<br>methoxyphenyl)pr	115.721	17.36,-<br>0.41,12.86,A,87,13<br>17.57,1.19,9.06,P,65,14

Caspase-3	MOL004425	Icariin	120.936	13.16,-<br>2.41,16.66,A,93,25<br>18.16,-1.41,11.46,A,77,28
Caspase-3	MOL004427	cariside A7	91.5555	18.96,-2.21,9.86,A,60,29<br>19.57,0.39,11.66,P,81,30<br>16.16,0.79,12.06,P,84,33
Caspase-3	MOL004941	(2R)-7-hydroxy-2-(4-hydroxyphenyl)chr	104.101	19.36,-0.01,9.86,A,61,2<br>14.96,-<br>1.41,16.66,A,94,15
Caspase-3	MOL005308	Aposiopolamine	99.3928	14.96,-1.41,16.66,A,94,5<br>17.77,0.19,10.06,A,67,13<br>16.96,-
Caspase-3	MOL005317	Deoxyharringtonine	107.276	17.77,1.19,9.06,A,57,1<br>19.96,-3.81,10.06,A,65,7<br>15.96,-
Caspase-3	MOL005318	Dianthramine	86.7343	16.96,-1.21,10.06,A,66,11<br>16.36,0.19,13.46,P,89,15<br>18.57,-2.21,8.06,P,54,21
Caspase-3	MOL005320	arachidonate	122.657	22.96,-9.21,6.26,A,36,10<br>19.96,-<br>3.81,10.06,A,65,16
Caspase-3	MOL005321	Frutinone A	91.4527	19.36,-0.01,9.86,A,61,1<br>18.16,-1.41,11.46,A,77,3<br>18.36,-
Caspase-3	MOL005348	Ginsenoside-Rh4_qt	107.5	22.96,-7.61,6.86,A,41,6<br>18.16,-1.41,11.46,A,77,26<br>19.36,-0.01,9.86,A,61,28
Caspase-3	MOL005356	Girinimbin	91.2668	18.96,-0.01,11.26,A,75,3<br>15.96,-<br>0.41,15.86,A,91,14
Caspase-3	MOL005376	Panaxadiol	79.8059	13.16,-2.41,16.66,A,93,6<br>19.96,-<br>3.81,10.06,A,65,20
Caspase-3	MOL005384	suchilactone	101.794	18.57,-7.41,14.46,A,90,1<br>18.96,-0.01,11.26,A,75,16<br>16.36,0.19,13.46,P,89,17
Caspase-3	MOL005399	alexandrin_qt	98.0734	18.36,-4.41,13.06,A,88,11<br>14.56,-<br>3.01,16.46,A,92,22
Caspase-3	MOL006331	4',5-Dihydroxyflavone	98.2186	18.57,-7.41,14.46,A,90,1<br>19.36,-0.01,9.86,A,61,16<br>17.57,1.19,9.06,P,65,17
Caspase-3	MOL008647	N-Trans-Feruloyltyramine	142.441	17.57,1.19,9.06,P,65,4<br>22.96,-7.61,6.86,A,41,12<br>18.16,-
Caspase-3	MOL009763	(+)-Syringaresinol-O-beta-D-glucoside	143.793	19.96,-2.61,9.46,P,69,9<br>18.16,0.99,12.06,P,86,11<br>17.77,-
Caspase-9	MOL000098	quercetin	94.1014	18.57,37.93,-17.80,A,2,6<br>20.17,42.13,-<br>12.60,A,14,13
Caspase-9	MOL000358	beta-sitosterol	94.2693	18.57,37.93,-17.80,A,2,4<br>21.57,42.93,-<br>12.20,A,20,27
Caspase-9	MOL000359	sitosterol	104.27	17.37,39.13,-18.00,A,1,11<br>20.17,42.13,-<br>12.60,A,14,19
Caspase-9	MOL000422	kaempferol	73.4995	28.97,29.53,-<br>5.60,A,60,14<br>30.97,29.73,-
Caspase-9	MOL000449	Stigmasterol	94.1775	18.37,38.93,-11.80,A,23,1<br>19.57,37.93,-<br>12.00,A,21,2

Caspase-9	MOL000787	Fumarine	65.3078	19.17,42.73,-17.60,A,3,7 18.57,37.93,-17.80,A,2,14
Caspase-9	MOL001510	24-epicampesterol	93.8254	17.37,39.13,-18.00,A,1,15 20.17,42.13,-
Caspase-9	MOL001525	Daucosterol	74.6382	30.97,36.13,-13.00,A,13,24 33.77,34.53,-
Caspase-9	MOL001645	Linoleyl acetate	106.023	33.17,37.33,-12.40,A,18,5 20.57,37.53,-
Caspase-9	MOL001771	poriferast-5-en-3beta-ol	107.215	19.17,42.73,-17.60,A,3,19 20.17,40.93,-
Caspase-9	MOL001792	DFV	97.364	18.57,37.93,-17.80,A,2,7 20.17,42.13,-12.60,A,14,14
Caspase-9	MOL002268	Rhein	75.9202	20.17,40.93,-10.80,A,33,11 18.97,41.33,-
Caspase-9	MOL002714	baicalein	96.704	20.57,37.53,-14.60,P,12,12 19.97,42.13,-
Caspase-9	MOL002879	Diop	94.8591	20.57,42.73,-11.40,A,28,20 32.37,36.53,-
Caspase-9	MOL002959	3'-Methoxydaidzein	59.2254	31.77,37.53,-12.20,A,19,13 33.17,35.33,-
Caspase-9	MOL003044	Chryseriol	80.9393	24.17,40.33,-11.20,P,32,12 19.97,42.13,-
Caspase-9	MOL003542	8-Isopentenyl-kaempferol	88.9453	20.17,40.53,-9.60,P,51,19 21.57,42.93,-12.20,A,20,21
Caspase-9	MOL003648	Inermin	85.3909	19.97,42.13,-10.40,P,42,3 20.97,39.93,-10.40,P,41,5 21.57,42.93,-
Caspase-9	MOL004367	olivil	110.652	25.57,40.13,-12.20,P,24,3 18.97,41.33,-13.40,P,18,4 19.97,43.53,-11.80,P,26,6
Caspase-9	MOL004373	Anhydroicaritin	107.587	21.57,43.73,-11.00,P,34,4 19.57,37.93,-12.00,A,21,22
Caspase-9	MOL004380	C-Homoerythrinan, 1,6-didehydro-	77.4659	19.17,42.73,-17.60,A,3,13 18.37,38.93,-
Caspase-9	MOL004382	Yinyanghuo A	109.441	28.57,34.73,-8.40,P,60,6 33.17,35.33,-13.00,A,12,22
Caspase-9	MOL004384	Yinyanghuo C	59.1649	33.17,37.33,-12.40,A,18,12 29.77,34.93,-
Caspase-9	MOL004388	6-hydroxy-11,12-dimethoxy-2,2-dimethyl-1,8-dioxo-2,3,4,8-tetrahydro-1H-isochromeno[3,4-	77.9283	17.37,39.13,-18.00,A,1,22 19.17,42.73,-17.60,A,3,24 20.17,42.13,-12.60,A,14,26
Caspase-9	MOL004391	8-(3-methylbut-2-enyl)-2-phenyl-chromone	95.1571	18.57,37.93,-17.80,A,2,16 19.57,37.93,-

Caspase-9	MOL004394	Anhydroicaritin-3-O-alpha-L-rhamnoside	40.6387	19.17,42.73,-17.60,A,3,44 17.37,39.13,-
Caspase-9	MOL004396	1,2-bis(4-hydroxy-3-methoxyphenyl)pr	102.584	20.17,42.13,-12.60,A,14,10 19.97,42.13,-
Caspase-9	MOL004427	Icariside A7	79.8341	19.17,42.73,-17.60,A,3,17 18.97,41.33,-
Caspase-9	MOL004941	(2R)-7-hydroxy-2-(4-hydroxyphenyl)chr	72.3115	19.17,42.73,-17.60,A,3,8 17.37,39.13,-18.00,A,1,15
Caspase-9	MOL005308	Aposiopolamine	87.8679	18.57,37.93,-17.80,A,2,6 20.17,40.93,-10.80,A,33,13
Caspase-9	MOL005317	Deoxyharringtonine	80.785	17.37,41.53,-17.80,P,3,14 19.77,37.93,-16.20,P,6,21 20.17,42.13,-
Caspase-9	MOL005318	Dianthramine	77.0754	27.77,30.33,-16.40,A,4,2 26.97,29.53,-14.40,P,14,7 21.77,31.33,-15.20,P,9,15
Caspase-9	MOL005320	arachidonate	113.423	32.37,36.53,-13.60,A,11,4 31.77,33.13,-10.60,A,34,6
Caspase-9	MOL005321	Frutinone A	88.4255	19.97,42.13,-10.40,P,42,13 18.37,38.93,-
Caspase-9	MOL005348	Ginsenoside-Rh4_qt	97.3867	19.17,42.73,-17.60,A,3,17 20.17,42.13,-
Caspase-9	MOL005384	suchilactone	99.6169	20.17,40.93,-10.80,A,33,1 18.17,37.73,-12.20,P,22,3
Caspase-9	MOL005399	alexandrin_qt	97.2948	16.37,38.73,-18.00,P,2,1 18.57,37.93,-17.80,A,2,20
Caspase-9	MOL006331	4',5-Dihydroxyflavone	80.55	31.17,28.33,-4.40,A,76,13 30.97,29.73,-
Caspase-9	MOL008647	N-Trans-Feruloyltyramine	105.421	33.17,42.13,-7.00,P,71,2 31.77,37.53,-12.20,A,19,16
Caspase-9	MOL009763	(+)-Syringaresinol-O-beta-D-glucoside	132.387	18.97,41.33,-13.40,P,18,4 21.57,43.73,-11.00,P,34,10
Caspase-8	MOL000098	quercetin	103.368	-8.91,35.72,36.36,A,41,13 -
Caspase-8	MOL000006	luteolin	106.153	-8.91,32.12,43.96,A,86,4 -7.31,33.52,37.76,A,58,18
Caspase-8	MOL000358	beta-sitosterol	111.787	-12.11,33.72,42.36,A,81,6 -12.31,31.52,46.36,A,99,1
Caspase-8	MOL000359	sitosterol	115.16	-8.51,34.12,38.76,A,63,2 -12.11,33.72,42.36,A,81,7 -
Caspase-8	MOL000422	kaempferol	96.8411	-8.91,35.72,36.36,A,41,14 -
Caspase-8	MOL000449	Stigmasterol	104.99	-12.11,33.72,42.36,A,81,10

Caspase-8	MOL000622	Magnograndiolide	77.9999	-9.11,33.92,40.56,A,71,5<br>-<br>8.91,32.12,43.96,A,86,16
Caspase-8	MOL000787	Fumarine	99.9685	-12.11,33.72,42.36,A,81,4<br>-<br>12.31,31.52,46.36,A,99,1
Caspase-8	MOL001510	24-epicampesterol	98.4481	-8.91,32.12,43.96,A,86,8<br>-<br>7.31,27.92,44.96,A,91,16
Caspase-8	MOL001525	Daucosterol	98.0274	-<br>10.91,33.32,41.16,A,74,9<br>-
Caspase-8	MOL001645	Linoleyl acetate	116.502	-<br>10.91,29.72,46.36,A,98,3
Caspase-8	MOL001771	poriferast-5-en-3beta-ol	103.293	-9.11,33.92,40.56,A,71,19<br>-<br>7.91,31.52,45.56,A,96,14<br>-
Caspase-8	MOL001792	DFV	103.127	-9.11,35.72,37.96,A,61,7<br>-<br>9.51,33.12,42.36,A,80,17
Caspase-8	MOL002268	Rhein	93.4428	-8.71,33.92,36.76,A,46,11<br>-8.11,33.32,39.36,P,69,20<br>-
Caspase-8	MOL002714	baicalein	91.6477	-9.11,35.72,37.96,A,61,4<br>-7.31,34.32,42.16,P,86,12<br>-8.51,35.32,35.76,P,37,14
Caspase-8	MOL002879	Diop	115.129	-6.31,34.52,37.76,A,59,4<br>-7.71,36.32,36.56,A,45,6<br>-
Caspase-8	MOL002959	3'-Methoxydaidzein	105.945	-12.11,33.72,42.36,A,81,1<br>-<br>13.71,32.52,45.36,A,93,8
Caspase-8	MOL003044	Chryseriol	108.496	-8.91,32.12,43.96,A,86,4<br>-<br>7.31,33.52,37.76,A,58,18
Caspase-8	MOL003542	8-Isopentenyl-kaempferol	109.953	-9.11,33.92,40.56,A,71,16<br>-6.51,33.92,37.76,P,58,19<br>-
Caspase-8	MOL003648	Inermin	100.395	-9.91,36.72,36.36,A,42,4<br>-8.51,34.12,38.76,A,63,9<br>-
Caspase-8	MOL004367	olivil	124.11	-7.91,36.72,36.36,P,45,4<br>-<br>9.51,33.12,42.36,A,80,13
Caspase-8	MOL004373	Anhydroicaritin	115.088	-9.11,33.92,40.56,A,71,21<br>-<br>9.51,29.92,45.56,A,95,26
Caspase-8	MOL004380	C-Homoerythrinan, 1,6-didehydro-	85.7481	-<br>12.31,31.52,46.36,A,99,7<br>-
Caspase-8	MOL004382	Yinyanghuo A	136.578	-5.91,33.92,35.36,P,30,6<br>-<br>8.91,32.12,43.96,A,86,15
Caspase-8	MOL004384	Yinyanghuo C	112.454	-8.71,33.92,36.76,A,46,3<br>-<br>8.91,32.12,43.96,A,86,18
Caspase-8	MOL004386	Yinyanghuo E	114.48	-8.71,33.92,36.76,A,46,3<br>-<br>8.91,32.12,43.96,A,86,18

Caspase-8	MOL004388	6-hydroxy-11,12-<br>dimethoxy-2,2-<br>dimethyl-1,8-<br>dioxo-2,3,4,8-<br>tetrahydro-1H-<br>isochromeno[3,4-	83.764	-6.51,33.92,37.76,P,58,19<br>-<br>13.71,33.32,41.96,A,77,2<br>1<br>-<br>11.51,38.32,35.56,A,33,2
Caspase-8	MOL004391	8-(3-methylbut-2-<br>enyl)-2-phenyl-<br>chromone	96.0478	-<br>12.11,40.12,35.16,A,31,1<br>7
Caspase-8	MOL004394	Anhydroicaritin-3-<br>O-alpha-L-<br>rhamnoside	131.727	-<br>3.51,24.12,41.56,A,75,27<br>-
Caspase-8	MOL004396	1,2-bis(4-hydroxy-<br>3-<br>methoxyphenyl)pr	111.833	-<br>13.51,33.32,43.56,A,85,1<br>0
Caspase-8	MOL004425	Icariin	93.3796	-<br>5.31,38.32,44.16,A,89,20<br>-
Caspase-8	MOL004427	Icariside A7	110.125	-<br>8.91,28.52,44.96,A,92,14<br>-
Caspase-8	MOL004941	(2R)-7-hydroxy-2-<br>(4-<br>hydroxyphenyl)chr	101.064	-9.11,35.72,37.96,A,61,2<br>-<br>14.71,41.12,34.96,A,28,1
Caspase-8	MOL005308	Aposiopolamine	94.9543	-<br>7.31,33.52,37.76,A,58,12<br>-
Caspase-8	MOL005317	Deoxyharringtonin<br>e	126.376	-<br>12.91,42.12,35.56,A,34,1<br>-
Caspase-8	MOL005318	Dianthramine	95.8557	-<br>7.51,35.92,37.76,A,60,12<br>-
Caspase-8	MOL005320	arachidonate	115.328	-<br>8.51,30.32,44.56,A,90,12
Caspase-8	MOL005321	Frutinone A	84.7428	-9.11,35.72,37.96,A,61,19<br>-9.91,36.72,36.36,A,42,1<br>-8.91,35.72,36.36,A,41,2<br>-
Caspase-8	MOL005348	Ginsenoside-<br>Rh4_qt	119.136	-<br>7.91,31.52,45.56,A,96,14<br>-
Caspase-8	MOL005356	Girinimbin	88.1552	-7.51,35.92,37.76,A,60,2<br>-<br>12.11,33.72,42.36,A,81,1
Caspase-8	MOL005376	Panaxadiol	86.0786	-<br>8.91,28.52,44.96,A,92,20<br>-
Caspase-8	MOL005384	suchilactone	101.391	-8.91,28.52,44.96,A,92,1<br>-<br>10.91,33.32,41.16,A,74,1
Caspase-8	MOL005399	alexandrin_qt	109.807	-<br>8.51,30.32,44.56,A,90,17<br>-
Caspase-8	MOL006331	4',5-<br>Dihydroxyflavone	94.7976	-8.91,32.12,43.96,A,86,6<br>-<br>7.31,33.52,37.76,A,58,14
Caspase-8	MOL008647	N-Trans-<br>Feruloyltyramine	129.882	-7.11,34.32,35.36,P,31,4<br>-<br>12.31,31.52,46.36,A,99,9
Caspase-8	MOL009763	(+)-Syringaresinol-<br>O-beta-D-<br>glucoside	139.36	-6.51,33.92,37.76,P,58,11<br>-8.51,35.32,35.76,P,37,13<br>-

BAX	MOL000098	quercetin	84.832	7.76,-29.68,-1.15,A,43,2<br>5.76,-35.28,3.45,A,75,12<br>6.16,-37.08,6.45,P,55,19
BAX	MOL000006	luteolin	88.1813	6.76,-29.88,0.25,A,53,11<br>7.56,-36.68,5.85,A,84,18<br>6.36,-37.28,7.85,P,65,21
BAX	MOL000358	beta-sitosterol	107.24	8.36,-30.28,0.85,A,56,9<br>4.36,-31.88,1.25,A,58,19<br>6.16,-35.68,5.65,A,83,28
BAX	MOL000359	sitosterol	102.413	3.76,-33.08,0.65,A,54,15<br>4.56,-34.48,4.25,A,78,18<br>8.36,-30.28,0.85,A,56,22
BAX	MOL000422	kaempferol	84.2762	7.76,-29.68,-1.15,A,43,9<br>5.76,-35.28,3.45,A,75,17<br>6.16,-37.08,6.45,P,55,18
BAX	MOL000449	Stigmasterol	101.685	8.16,-27.08,1.25,A,59,7<br>3.36,-32.48,2.45,A,68,22<br>7.76,-31.28,2.05,A,66,24
BAX	MOL000622	Magnograndiolide	69.8422	6.16,-30.08,-1.35,A,40,8<br>4.76,-29.48,-0.95,A,44,9<br>6.76,-28.88,1.65,A,63,16
BAX	MOL000787	Fumarine	93.4325	6.56,-32.28,2.65,A,70,4<br>5.96,-28.88,-0.35,A,49,12<br>8.36,-35.08,9.45,A,96,26
BAX	MOL001510	24-epicampesterol	103.078	7.76,-29.68,-1.15,A,43,13<br>6.16,-35.68,5.65,A,83,26<br>4.36,-30.48,1.45,A,61,27
BAX	MOL001525	Daucosterol	96.7948	4.76,-29.48,-0.95,A,44,7<br>7.96,-28.28,0.65,A,55,11<br>3.56,-34.68,2.85,A,71,19
BAX	MOL001645	Linoleyl acetate	101.26	4.96,-29.48,0.85,A,57,4<br>4.56,-34.48,4.25,A,78,10<br>7.56,-36.68,5.85,A,84,17
BAX	MOL001771	poriferast-5-en-3beta-ol	104.408	8.16,-27.08,1.25,A,59,8<br>7.56,-36.68,5.85,A,84,26<br>3.36,-32.48,2.45,A,68,27
BAX	MOL001792	DFV	98.4017	2.76,-20.28,4.85,A,80,10<br>-2.24,-20.88,9.05,A,94,14<br>2.36,-24.08,4.45,P,50,19
BAX	MOL002268	Rhein	91.2653	0.76,-22.68,10.05,P,82,15<br>-2.84,-19.48,8.45,A,92,17<br>5.56,-21.88,8.85,P,73,21
BAX	MOL002714	baicalein	80.183	4.96,-33.28,3.05,A,73,11<br>6.36,-37.28,7.85,P,65,15<br>7.76,-31.28,2.05,A,66,17
BAX	MOL002879	Diop	88.6331	5.96,-28.88,-0.35,A,49,3<br>3.96,-30.28,-0.75,A,45,5<br>7.76,-35.48,8.25,A,90,27
BAX	MOL002959	3'-Methoxydaidzein	89.2194	5.76,-35.28,3.45,A,75,1<br>7.76,-35.28,6.65,A,86,8<br>6.76,-29.88,0.25,A,53,19
BAX	MOL003044	Chryseriol	89.3009	6.56,-32.28,2.65,A,70,9<br>3.36,-29.08,-2.15,P,33,14<br>3.56,-34.68,2.85,A,71,16
BAX	MOL003542	8-Isopentenyl-kaempferol	100.364	6.76,-29.88,0.25,A,53,9<br>6.36,-37.28,7.85,P,65,19<br>7.56,-36.68,5.85,A,84,20
BAX	MOL003648	Inermin	83.1698	7.76,-29.68,-1.15,A,43,4<br>7.76,-31.28,2.05,A,66,9<br>4.96,-33.28,3.05,A,73,10
BAX	MOL004367	olivil	105.651	8.56,-31.28,9.45,P,77,4<br>6.16,-35.68,5.65,A,83,11<br>7.76,-31.28,2.05,A,66,24

BAX	MOL004373	Anhydroicaritin	99.6021	6.16,-37.08,6.45,P,55,6<br>5.96,-28.88,-0.35,A,49,17<br>5.76,-35.28,3.45,A,75,19
BAX	MOL004380	C-Homoerythrinan, 1,6-didehydro-	79.319	3.76,-33.08,0.65,A,54,8<br>4.56,-34.48,4.25,A,78,16<br>6.16,-35.68,5.65,A,83,18
BAX	MOL004382	Yinyanghuo A	113.828	5.36,-28.68,1.65,A,64,11<br>4.96,-33.28,3.05,A,73,18<br>4.16,-28.48,-1.35,A,41,27
BAX	MOL004384	Yinyanghuo C	90.996	6.16,-37.08,6.45,P,55,21<br>6.76,-29.88,0.25,A,53,23<br>4.76,-29.48,-0.95,A,44,25
BAX	MOL004386	Yinyanghuo E	96.9356	8.36,-30.28,0.85,A,56,14<br>4.96,-30.88,0.05,A,51,23<br>3.76,-33.08,0.65,A,54,25
BAX	MOL004388	6-hydroxy-11,12-dimethoxy-2,2-dimethyl-1,8-dioxo-2,3,4,8-tetrahydro-1H-isochromeno[3,4-	83.7921	6.56,-32.28,2.65,A,70,10<br>4.96,-29.48,0.85,A,57,22<br>8.16,-27.08,1.25,A,59,24
BAX	MOL004391	8-(3-methylbut-2-enyl)-2-phenyl-chromone	98.3812	3.96,-19.68,5.25,A,82,6<br>2.76,-20.28,4.85,A,80,7<br>-2.84,-19.48,8.45,A,92,22
BAX	MOL004394	Anhydroicaritin-3-O-alpha-L-rhamnoside	124.82	6.36,-37.28,7.85,P,65,28<br>7.76,-31.28,2.05,A,66,32<br>3.56,-34.68,2.85,A,71,45
BAX	MOL004396	1,2-bis(4-hydroxy-3-methoxyphenyl)pr	88.0687	8.56,-31.28,9.45,P,77,4<br>7.76,-35.48,8.25,A,90,10<br>5.36,-34.48,12.05,P,93,21
BAX	MOL004425	Icariin	101.648	7.76,-31.28,2.05,A,66,21<br>6.76,-34.88,11.25,P,87,46<br>7.56,-28.28,-0.75,A,46,47
BAX	MOL004427	Icariside A7	81.5559	6.16,-30.88,1.45,A,60,10<br>7.56,-36.68,5.85,A,84,27<br>6.16,-35.68,7.45,P,61,32
BAX	MOL004941	(2R)-7-hydroxy-2-(4-hydroxyphenyl)chr	81.3849	2.56,-21.08,3.65,A,76,2<br>0.36,-22.48,9.65,A,97,15<br>0.96,-20.28,2.25,P,41,19
BAX	MOL005308	Aposiopolamine	75.1408	10.76,-28.28,-6.95,A,21,1<br>7.76,-29.68,-1.15,A,43,11<br>6.76,-28.88,1.65,A,63,16
BAX	MOL005317	Deoxyharringtonine	107.836	7.76,-35.28,6.65,A,86,22<br>4.36,-31.88,1.25,A,58,32<br>3.56,-34.68,2.85,A,71,35
BAX	MOL005318	Dianthramine	71.2661	3.56,-34.68,2.85,A,71,3<br>5.96,-28.88,-0.35,A,49,12<br>4.36,-34.48,5.45,P,52,19
BAX	MOL005320	arachidonate	106.027	4.76,-29.48,-0.95,A,44,8<br>6.56,-32.28,2.65,A,70,12<br>8.36,-31.48,10.85,P,85,21
BAX	MOL005321	Frutinone A	78.2474	7.76,-35.48,8.25,A,90,6<br>4.56,-34.48,4.25,A,78,14<br>6.16,-30.88,1.45,A,60,17
BAX	MOL005348	Ginsenoside-Rh4_qt	102.156	8.16,-27.08,1.25,A,59,16<br>6.76,-29.88,0.25,A,53,19<br>7.76,-31.28,2.05,A,66,26
BAX	MOL005356	Girinimbin	81.0246	7.76,-35.28,6.65,A,86,1<br>6.76,-29.88,0.25,A,53,15<br>4.76,-29.48,-0.95,A,44,20
BAX	MOL005376	Panaxadiol	89.8061	7.76,-35.48,8.25,A,90,7<br>4.96,-30.88,0.05,A,51,20<br>6.76,-28.88,1.65,A,63,30

BAX	MOL005384	suchilactone	88.5185	4.76,-29.48,-0.95,A,44,2<br>8.36,-30.28,0.85,A,56,8<br>6.76,-36.28,7.25,A,88,21
BAX	MOL005399	alexandrin_qt	104.83	7.56,-36.68,5.85,A,84,12<br>5.36,-28.68,1.65,A,64,27<br>4.16,-28.48,-1.35,A,41,28
BAX	MOL006331	4',5-Dihydroxyflavone	79.9819	4.76,-29.48,-0.95,A,44,2<br>4.56,-34.48,4.25,A,78,13<br>6.16,-37.08,6.45,P,55,17
BAX	MOL008647	N-Trans-Feruloyltyramine	94.5218	7.76,-35.28,6.65,A,86,7<br>4.96,-33.28,3.05,A,73,16<br>4.36,-31.88,1.25,A,58,20
BAX	MOL009763	(+)-Syringaresinol-O-beta-D-glucoside	124.036	7.76,-35.48,8.25,A,90,19<br>4.36,-30.48,1.45,A,61,32<br>7.96,-28.28,0.65,A,55,37
Bcl2	MOL000098	quercetin	83.8346	8.06,7.72,-5.90,A,10,16<br>9.06,5.32,-5.30,P,33,20<br>5.66,8.12,-5.90,P,29,21
Bcl2	MOL000006	luteolin	77.1073	5.06,-10.08,-8.90,A,3,4<br>4.06,-3.88,-7.70,A,4,18<br>4.86,-1.88,-6.70,P,23,21
Bcl2	MOL000358	beta-sitosterol	112.64	9.26,6.32,-4.30,A,16,13<br>8.06,7.72,-5.90,A,10,20<br>17.26,-0.88,2.70,A,37,28
Bcl2	MOL000359	sitosterol	103.135	9.26,6.32,-4.30,A,16,15<br>10.66,5.72,-1.50,A,20,20<br>8.06,7.72,-5.90,A,10,28
Bcl2	MOL000422	kaempferol	96.9172	4.26,-2.48,-7.50,A,5,9<br>7.66,-3.48,-5.10,P,36,11<br>8.86,-5.88,-6.10,P,27,21
Bcl2	MOL000449	Stigmasterol	110.795	4.26,-2.48,-7.50,A,5,1<br>7.06,-3.88,-5.70,A,13,6<br>8.26,-4.88,-5.70,A,11,7
Bcl2	MOL000622	Magnograndiolide	65.022	13.06,-7.08,0.50,A,26,9<br>13.46,-9.08,5.90,A,65,12<br>17.46,-7.08,0.70,A,27,16
Bcl2	MOL000787	Fumarine	101.893	17.46,-7.08,0.70,A,27,15<br>14.06,-7.48,1.70,A,34,18<br>10.46,-7.28,5.10,P,71,25
Bcl2	MOL001510	24-epicampesterol	103.223	9.26,6.32,-4.30,A,16,21<br>10.66,5.72,-1.50,A,20,27<br>8.06,7.72,-5.90,A,10,28
Bcl2	MOL001510	24-epicampesterol	103.223	9.26,6.32,-4.30,A,16,21<br>10.66,5.72,-1.50,A,20,27<br>8.06,7.72,-5.90,A,10,28
Bcl2	MOL001645	Linoleyl acetate	112.766	12.86,-9.28,2.50,A,36,4<br>16.06,-7.48,1.50,A,33,7<br>17.26,-3.08,11.50,P,97,21
Bcl2	MOL001771	poriferast-5-en-3beta-ol	100.868	17.46,-7.08,0.70,A,27,27<br>12.06,-10.28,1.10,A,29,29
Bcl2	MOL001792	DFV	77.3364	14.06,-7.48,1.70,A,34,7<br>12.86,-9.28,2.50,A,36,9<br>12.06,-7.88,0.70,P,62,19
Bcl2	MOL002268	Rhein	81.2143	4.06,-6.88,-9.70,P,4,15<br>4.06,-3.28,-6.30,A,9,17<br>8.86,-5.88,-6.10,P,27,20
Bcl2	MOL002714	baicalein	85.5592	8.26,-4.88,-5.70,A,11,4<br>7.06,-4.88,-10.10,P,2,12<br>7.66,-3.28,-4.30,P,45,14
Bcl2	MOL002879	Diop	104.877	17.26,-2.08,11.30,A,98,20<br>17.46,-7.08,0.70,A,27,22<br>12.26,-7.88,4.70,A,52,27

Bcl2	MOL002959	3'-Methoxydaidzein	88.228	5.46,-2.48,-6.50,A,7,6<br>4.06,-3.88,-7.70,A,4,8<br>8.86,-5.08,-7.30,P,19,11
Bcl2	MOL003044	Chryseriol	78.9466	8.06,-5.08,-5.50,P,31,10<br>7.26,-2.68,-6.50,P,24,12<br>4.06,-11.08,-9.50,A,1,22
Bcl2	MOL003542	8-Isopentenyl-kaempferol	92.8198	17.06,-0.48,5.10,P,72,19<br>14.06,-8.08,4.50,A,49,22<br>14.06,-7.48,1.70,A,34,26
Bcl2	MOL003648	Inermin	97.907	8.86,-5.08,-7.30,P,19,12<br>4.06,-3.88,-7.70,A,4,17<br>5.46,-2.48,-6.50,A,7,19
Bcl2	MOL004367	olivil	107.683	9.26,-7.08,-4.90,P,38,1<br>5.86,-2.68,-8.10,P,13,6<br>5.06,-10.08,-8.90,A,3,27
Bcl2	MOL004373	Anhydroicaritin	101.112	12.26,-7.88,4.70,A,52,21<br>17.46,-7.08,0.70,A,27,25<br>10.26,-6.88,7.30,A,84,27
Bcl2	MOL004380	C-Homoerythrinan, 1,6-didehydro-	72.5927	17.46,-7.08,0.70,A,27,9<br>14.06,-8.08,4.50,A,49,18<br>12.86,-9.28,2.50,A,36,24
Bcl2	MOL004382	Yinyanghuo A	114.927	17.46,-7.08,0.70,A,27,19<br>17.26,-0.88,2.70,A,37,26<br>16.06,-2.48,4.30,A,47,27
Bcl2	MOL004384	Yinyanghuo C	82.8389	17.26,-8.48,1.50,A,32,13<br>14.06,-8.08,4.50,A,49,23<br>12.86,-9.28,2.50,A,36,25
Bcl2	MOL004386	Yinyanghuo E	102.841	8.66,-4.08,-6.30,P,25,22<br>4.26,-2.48,-7.50,A,5,25<br>4.06,-4.28,-5.70,A,12,26
Bcl2	MOL004388	6-hydroxy-11,12-dimethoxy-2,2-dimethyl-1,8-dioxo-2,3,4,8-tetrahydro-1H-isochromeno[3,4-	34.6675	16.06,-2.48,4.30,A,47,12<br>16.06,-7.48,1.50,A,33,21<br>17.06,-0.48,5.10,P,72,27
Bcl2	MOL004391	8-(3-methylbut-2-enyl)-2-phenyl-chromone	90.8299	14.66,-9.88,5.10,A,55,2<br>11.06,-8.48,6.70,A,77,14<br>16.06,-2.48,4.30,A,47,21
Bcl2	MOL004394	Anhydroicaritin-3-O-alpha-L-rhamnoside	109.645	15.26,-8.08,6.70,P,77,8<br>17.26,-0.88,2.70,A,37,26<br>16.06,-2.48,4.30,A,47,27
Bcl2	MOL004396	1,2-bis(4-hydroxy-3-methoxyphenyl)pr	105.742	16.06,-7.48,1.50,A,33,3<br>14.66,-9.88,5.10,A,55,10<br>11.26,-7.28,5.30,A,60,13
Bcl2	MOL004427	Icariside A7	88.3736	5.06,-10.08,-8.90,A,3,17<br>7.66,-3.48,-5.10,P,36,31<br>6.26,-1.48,-7.50,P,18,32
Bcl2	MOL004941	(2R)-7-hydroxy-2-(4-hydroxyphenyl)chr	87.7389	13.46,-9.08,5.90,A,65,1<br>17.26,-8.48,1.50,A,32,12<br>10.46,-7.28,5.10,P,71,19
Bcl2	MOL005308	Aposiopolamine	94.1829	10.86,8.12,4.10,A,45,2<br>10.66,9.92,5.70,A,64,6<br>5.46,5.12,6.50,A,75,11
Bcl2	MOL005317	Deoxyharringtonine	91.5761	17.46,-2.48,9.50,P,84,14<br>16.06,-1.68,3.10,A,40,22<br>17.26,-8.48,1.50,A,32,32
Bcl2	MOL005318	Dianthramine	84.6251	4.06,-3.88,-7.70,A,4,3<br>7.26,-2.68,-6.50,P,24,7<br>4.06,-6.88,-9.70,P,4,18
Bcl2	MOL005320	arachidonate	114.073	14.66,-9.88,5.10,A,55,3<br>12.26,-7.88,4.70,A,52,6<br>17.26,-3.08,11.50,P,97,22

Bcl2	MOL005321	Frutinone A	86.5979	4.06,-3.28,-6.30,A,9,2<br>9.86,-8.08,-4.10,A,18,16<br>7.06,-4.88,-10.10,P,2,19
Bcl2	MOL005348	Ginsenoside-Rh4_qt	103.031	17.46,-7.08,0.70,A,27,24<br>12.26,-7.88,4.70,A,52,26<br>11.06,-8.48,6.70,A,77,28
Bcl2	MOL005356	Girinimbin	66.9315	14.06,-8.08,4.50,A,49,14<br>17.46,-7.08,0.70,A,27,18<br>12.66,-8.08,1.30,A,31,20
Bcl2	MOL005376	Panaxadiol	72.0105	17.26,-8.48,1.50,A,32,16<br>14.46,-9.08,2.90,A,38,18<br>12.66,-8.08,1.30,A,31,21
Bcl2	MOL005384	suchilactone	97.9435	16.06,-2.48,4.30,A,47,1<br>17.46,-7.08,0.70,A,27,11<br>13.46,-9.08,5.90,A,65,21
Bcl2	MOL005399	alexandrin_qt	105.659	16.06,-1.68,3.10,A,40,18<br>12.86,-7.88,3.10,A,39,27<br>11.86,-8.68,6.10,A,69,28
Bcl2	MOL006331	4',5-Dihydroxyflavone	71.597	5.06,-10.08,-8.90,A,3,6<br>4.06,-3.88,-7.70,A,4,14<br>4.86,-1.88,-6.70,P,23,17
Bcl2	MOL008647	N-Trans-Feruloyltyramine	113.617	16.86,-1.88,10.70,P,89,2<br>12.86,-9.28,2.50,A,36,22<br>11.06,-8.48,6.70,A,77,23
Bcl2	MOL009763	(+)-Syringaresinol-O-beta-D-glucoside	106.571	15.26,-8.08,6.70,P,77,9<br>14.06,-7.48,1.70,A,34,37<br>17.46,-7.08,0.70,A,27,39
